# Supplementary material for: Route of oxytocin administration for preventing blood loss at caesarean section: a systematic review with meta-analysis
Source: BMJ Open. 2021 Sep 16;11(9):e051793. doi: 10.1136/bmjopen-2021-051793 (PMC8449971; doi:10.1136/bmjopen-2021-051793)
Supplement: Supplementary data [file bmjopen-2021-051793supp002.pdf]

Supplementary file 2. Search strategy for systematic review on oxytocin route of delivery

Database	Search strategy	Results
BVS	#1 MH:Cesárea OR MH:"Cesarean Section" OR Cesárea OR (Cesarean Section) OR (Parto Abdominal) OR (Abdominal Deliveries) OR (Abdominal Delivery) OR (C Section (OB)) OR (C-Section (OB)) OR (C-Sections (OB)) OR (Caesarean Section) OR (Caesarean Sections) OR (Cesarean Sections) OR (Postcesarean Section) OR MH:E04.520.252.500$ OR MH:"Cesárea Repetida" OR MH:"Cesarean Section, Repeat" OR MH:Recesariana OR (Cesárea Repetida) OR Recesariana OR (Repeat Cesarean Section) OR (Repeat Cesarean Sections) OR (Operación Cesárea Repetida) OR MH:E04.520.252.500.150$ OR MH:"Parto Obstétrico" OR MH:"Delivery, Obstetric" OR (Parto Obstétrico) OR (Liberação Obstétrica) OR (Obstetric Deliveries) OR (Obstetric Delivery) OR (Liberación Obstétrica) OR MH:E04.520.252$ OR MH:"Terceira Fase do Trabalho de Parto" OR MH:"Labor Stage, Third" OR MH:"Tercer Periodo del Trabajo de Parto" OR (Terceira Fase do Trabalho de Parto) OR (Tercer Periodo del Trabajo de Parto) OR (Terceiro Estágio do Trabalho de Parto) OR (Terceiro Período do Trabalho de Parto) OR (Third Labor Stage) OR (Third Labor Stages) OR (Third Stage Labor) OR MH:G08.686.784.769.326.500.110$<br><br>#2 MH:Oxitocina OR MH:Oxytocin OR MH:Ocitocina OR Oxitocina OR Oxytocin OR Ocitocina OR Ocytocin OR Pitocin OR Syntocinon OR MH:D06.472.699.631.692.433$ OR MH:D12.644.548.691.692.433$ OR MH:"Receptores de Ocitocina" OR MH:"Receptors, Oxytocin" OR MH:"Receptores de Oxitocina" OR (Receptores de Ocitocina) OR (Receptores de Oxitocina) OR (Oxytocin Receptor) OR (Oxytocin Receptors) OR MH:D12.776.543.750.695.630$ OR MH:D12.776.543.750.720.600.630$ OR MH:D12.776.543.750.750.555.630$ OR MH:D12.776.543.750.750.660.600$ OR MH:Ocitócicos OR MH:Oxytocics OR MH:Oxitócicos OR Ocitócicos OR Oxytocics OR Oxitócicos OR (Efeito Ocitócico) OR (Estimulantes Uterinos) OR (Oxytocic Agents) OR (Oxytocic Drugs) OR (Oxytocic Effect) OR (Oxytocic Effects) OR (Uterine Stimulants) OR (Efecto Oxitócico) OR MH:D27.505.696.875.737$ OR MH:D27.505.954.705.737$<br><br>#3 #1 AND #2	376
CINAHL (via EBSCO)	S1 MH Cesarean Section OR ((Cesarean Sections) OR (Cesarean Section) OR (Abdominal Deliveries) OR (Abdominal Deliver) OR (Abdominal Delivery) OR (C-Section (OB)) OR (C Section (OB)) OR (C-Sections (OB)) OR (Postcesarean Section) OR (cesarean delivery) OR (Cesarean Section) OR (Elective cesarean section) OR (Elective cesarean delivery) OR Cesarean OR (Emergency cesarean section) OR (Emergency cesarean delivery) OR (Intrapartum cesarean section) OR (Intrapartum cesarean delivery) OR (Intra-partum cesarean section) OR (Intra-partum cesarean delivery) OR (Intra partum cesarean section) OR (Intra partum cesarean delivery) OR (Caesarean Section) OR (Caesarean Sections) OR (Postcaesarean Section) OR (caesarean delivery) OR (Elective caesarean section) OR (Elective caesarean delivery) OR Caesarean OR (Emergency caesarean section) OR (Emergency caesarean delivery) OR (Intrapartum caesarean section) OR (Intrapartum caesarean delivery) OR (Intra-partum caesarean section) OR (Intra-partum caesarean delivery) OR (Intra partum caesarean section) OR (Intra partum caesarean delivery) OR (Prelabor cesarean section) OR (Prelabor cesarean delivery) OR (Pre-labor cesarean delivery) OR (Pre-labor cesarean delivery) OR (Pre labor cesarean section) OR (Pre labor cesarean delivery) OR (Prelabour cesarean section) OR (Prelabour cesarean delivery) OR (Pre-labour cesarean delivery) OR (Pre-labour cesarean delivery) OR (Pre labour cesarean section) OR (Pre labour cesarean delivery) OR (Prelabor caesarean section) OR (Prelabor caesarean delivery) OR (Pre-labor caesarean delivery) OR (Pre-labor caesarean delivery) OR (Pre labor caesarean section) OR (Pre labor caesarean delivery) OR (Prelabour caesarean section) OR (Prelabour caesarean delivery) OR (Pre-labour caesarean delivery) OR (Pre-labour caesarean delivery) OR (Pre labour caesarean section) OR (Pre labour caesarean delivery) OR (Repeat Cesarean Section) OR (Repeat Cesarean Sections) OR (Repeat Caesarean Section) OR (Repeat Caesarean Sections) OR (Obstetric anesthesia) OR (Obstetric Deliveries) OR (Obstetric Delivery) OR (Third Labor Stage) OR (Third Labor Stages) OR (Third Stage Labor) OR (Third Stage of Labor) OR (Third-stage labor) OR (Third Labour Stage) OR (Third Labour Stages) OR (Third Stage Labour) OR (Third Stage of Labour) OR (Third-stage labour)) OR MH Cesarean Section, Repeat OR MH Delivery, Obstetric OR MH Labor Stage, Third<br><br>S2 MH Oxytocin OR MH Receptors, Oxytocin OR MH Oxytocics OR (Oxytocin OR Ocytocin OR Syntocinon OR Pitocin OR (Oxytocin Receptor) OR (Oxytocin Receptors) OR (alpha hypophamine) OR (atonin o) OR (beta methyl beta mercaptobutyric acid oxytocin) OR (di sipidin) OR disipidin OR endopituitrin OR fetusin OR hypophysin OR hypophysine OR (intravenous oxytocin) OR mipareton OR neoxyn OR ocytocin OR orasthin OR orastina OR oxitocin OR (oxiton inj) OR oxitone OR oxystin OR oxytan OR (oxytocin 10 usp units in dextrose 5%) OR (oxytocin 20 usp units in dextrose 5%) OR (oxytocin 5 usp units in dextrose 5%) OR (oxytocin dimer) OR (oxytocin pentapeptide) OR (oxytocin s inj) OR oxytocine OR pareton OR partocon OR (partocon inj) OR partolact OR partoxin OR physormon OR pitocin OR (pitocin inj) OR piton OR (piton s) OR (piton s inj) OR pituilobine OR pitupartin OR solvoxine OR synpitan OR (synthetic oxytocin inj) OR syntocinon OR (syntocinon inj) OR (syntocinon spray) OR tranoxy OR utedrin OR uteracon OR uterason OR (utron inj) OR xitocin OR Oxytocics OR (Oxytocic Drugs) OR (Uterine Stimulants) OR (Oxytocic Agents) OR (Oxytocic Effect) OR (Oxytocic Effects)<br><br>S3 S1 AND S2	1,563

ClinicalTrials.gov	Condition: (Cesarean Sections) OR (Cesarean Section) OR (Delivery, Abdominal) OR (Abdominal Deliveries) OR (Abdominal Deliver) OR (Deliveries, Abdominal) OR (Abdominal Delivery) OR "C-Section (OB)" OR "C Section (OB)" OR "C-Sections (OB)"<br><br>Intervention: Oxytocin OR Ocytocin OR Syntocinon OR Pitocin OR (Oxytocin Receptor) OR (Receptor, Oxytocin) OR (Oxytocin Receptors) OR (alpha hypophamine) OR "atonin o" OR "beta methyl beta mercaptobutyric acid oxytocin" OR "di sipidin" OR disipidin	90
Cochrane Library	#1 MeSH descriptor: [Cesarean Section] explode all trees<br>#2 (Cesarean Sections) OR (Cesarean Section) OR (Delivery, Abdominal) OR (Abdominal Deliveries) OR (Abdominal Deliver) OR (Deliveries, Abdominal) OR (Abdominal Delivery) OR "C-Section (OB)" OR "C Section (OB)" OR "C-Sections (OB)" OR (Postcesarean Section) OR (cesarean delivery) OR (Cesarean Section) OR (Elective cesarean section) OR (Elective cesarean delivery) OR Cesarean OR (Emergency cesarean section) OR (Emergency cesarean delivery) OR (Intrapartum cesarean section) OR (Intrapartum cesarean delivery) OR (Intra-partum cesarean section) OR (Intra-partum cesarean delivery) OR (Intra partum cesarean section) OR (Intra partum cesarean delivery)<br>#3 (Caesarean Section) OR (Caesarean Sections) OR (Postcaesarean Section) OR (caesarean delivery) OR (Elective caesarean section) OR (Elective caesarean delivery) OR Caesarean OR (Emergency caesarean section) OR (Emergency caesarean delivery) OR (Intrapartum caesarean section) OR (Intrapartum caesarean delivery) OR (Intra-partum caesarean section) OR (Intra-partum caesarean delivery) OR (Intra partum caesarean section) OR (Intra partum caesarean delivery)<br>#4 (Prelabor cesarean section) OR (Prelabor cesarean delivery) OR (Pre-labor cesarean delivery) OR (Pre-labor cesarean delivery) OR (Pre labor cesarean section) OR (Pre labor cesarean delivery)<br>#5 (Prelabour cesarean section) OR (Prelabour cesarean delivery) OR (Pre-labour cesarean delivery) OR (Pre-labour cesarean delivery) OR (Pre labour cesarean section) OR (Pre labour cesarean delivery)<br>#6 (Prelabor caesarean section) OR (Prelabor caesarean delivery) OR (Pre-labor caesarean delivery) OR (Pre-labor caesarean delivery) OR (Pre labor caesarean section) OR (Pre labor caesarean delivery)<br>#7 (Prelabour caesarean section) OR (Prelabour caesarean delivery) OR (Pre-labour caesarean delivery) OR (Pre-labour caesarean delivery) OR (Pre labour caesarean section) OR (Pre labour caesarean delivery)<br>#8 MeSH descriptor: [Cesarean Section, Repeat] explode all trees<br>#9 (Repeat Cesarean Section) OR (Cesarean Sections, Repeat) OR (Repeat Cesarean Sections) OR (Section, Repeat Cesarean) OR (Sections, Repeat Cesarean)<br>#10 (Repeat Caesarean Section) OR (Caesarean Sections, Repeat) OR (Repeat Caesarean Sections) OR (Section, Repeat Caesarean) OR (Sections, Repeat Caesarean) OR (Obstetric anesthesia)<br>#11 MeSH descriptor: [Delivery, Obstetric] explode all trees<br>#12 (Deliveries, Obstetric) OR (Obstetric Deliveries) OR (Obstetric Delivery)<br>#13 MeSH descriptor: [Labor Stage, Third] explode all trees<br>#14 (Labor Stages, Third) OR (Stage, Third Labor) OR (Stages, Third Labor) OR (Third Labor Stage) OR (Third Labor Stages) OR (Labor, Third Stage) OR (Third Stage Labor) OR "Third Stage of Labor" OR "Third-stage labor"<br>#15 (Labour Stage, Third) OR (Labour Stages, Third) OR (Stage, Third Labour) OR (Stages, Third Labour) OR (Third Labour Stage) OR (Third Labour Stages) OR (Labour, Third Stage) OR (Third Stage Labour) OR "Third Stage of Labour" OR "Third-stage labour"<br>#16 #1 OR #2 OR #3 OR #4 OR #5 OR #6 OR #7 OR #8 OR #9 OR #10 OR #11 OR #12 OR #13 OR #14 OR #15<br>#17 MeSH descriptor: [Oxytocin] explode all trees<br>#18 Oxytocin OR Ocytocin OR Syntocinon OR Pitocin<br>#19 MeSH descriptor: [Receptors, Oxytocin] explode all trees<br>#20 (Oxytocin Receptor) OR (Receptor, Oxytocin) OR (Oxytocin Receptors) OR (alpha hypophamine) OR "atonin o" OR "beta methyl beta mercaptobutyric acid oxytocin" OR "di sipidin" OR disipidin OR endopituitrin OR fetusin OR hypophysin OR hypophysine OR (intravenous oxytocin) OR mipareton OR neoxyn OR ocytocin OR orasthin OR orastina OR oxitocin OR "oxiton inj" OR oxitone OR oxystin OR oxytan OR (oxytocin 10 usp units in dextrose 5%) OR (oxytocin 20 usp units in dextrose 5%) OR (oxytocin 5 usp units in dextrose 5%) OR (oxytocin dimer) OR (oxytocin pentapeptide) OR "oxytocin s inj" OR oxytocine OR pareton OR partacon OR partocon OR "partocon inj" OR partolact OR partoxin OR physormon OR pitocin OR "pitocin inj" OR piton OR "piton s" OR "piton s inj" OR pituilobine OR pitupartin OR solvoxine OR synpitan OR (synthetic oxytocin inj) OR syntocinon OR "syntocinon inj" OR (syntocinon spray) OR tranoxy OR utedrin OR uteracon OR uterason OR "utron inj" OR xitocin<br>#21 MeSH descriptor: [Oxytocics] explode all trees<br>#22 Oxytocics OR (Oxytocic Drugs) OR (Drugs, Oxytocic) OR (Uterine Stimulants) OR (Stimulants, Uterine) OR (Oxytocic Agents) OR (Agents, Oxytocic) OR (Oxytocic Effect) OR (Effect, Oxytocic) OR (Oxytocic Effects) OR (Effects, Oxytocic)<br>#23 #17 OR #18 OR #19 OR #20 OR #21 OR #22<br>#24 #16 AND #23<br>#25 #24 in Trials	2,375
Embase	#1 'cesarean section'/exp OR 'cesarean section' OR 'birth, abdominal operation'/exp OR 'birth, abdominal operation' OR 'birth, caesarean'/exp OR 'birth, caesarean' OR 'caesarean birth'/exp OR 'caesarean birth' OR 'caesarean section'/exp OR 'caesarean section' OR 'caesarian birth'/exp OR 'caesarian birth' OR 'caesarian section'/exp OR 'caesarian section' OR 'cesarean delivery'/exp OR 'cesarean delivery' OR 'cesarian section'/exp OR 'cesarian section' OR 'elective repeat cesarean	3,804

	section'/exp OR 'elective repeat cesarean section' OR 'fetectomy'/exp OR 'fetectomy' OR 'repeated cesarotomy'/exp OR 'repeated cesarotomy' OR 'sectio caesarea'/exp OR 'sectio caesarea'<br><br>#2 'repeat cesarean section'/exp OR 'repeat cesarean section' OR 'cesarean section, repeat'/exp OR 'cesarean section, repeat' OR 'repeat cesarean section, elective'/exp OR 'repeat cesarean section, elective' OR 'repeat cesarotomy'/exp OR 'repeat cesarotomy' OR 'repeat section, cesarean'/exp OR 'repeat section, cesarean' OR 'repeated cesarean section'/exp OR 'repeated cesarean section' OR 'section, elective repeat cesarean'/exp OR 'section, elective repeat cesarean' OR 'section, repeat cesarean'/exp OR 'section, repeat cesarean'<br><br>#3 'obstetric delivery'/exp OR 'obstetric delivery' OR 'delivery'/exp OR delivery OR 'delivery pattern'/exp OR 'delivery pattern' OR 'delivery term'/exp OR 'delivery term' OR 'delivery, obstetric'/exp OR 'delivery, obstetric'<br><br>#4 'labor stage 3'/exp OR 'labor stage 3' OR 'delivery stage 3'/exp OR 'delivery stage 3' OR 'delivery stage iii'/exp OR 'delivery stage iii' OR 'labor stage iii'/exp OR 'labor stage iii' OR 'labor stage, third'/exp OR 'labor stage, third' OR 'labour stage 3'/exp OR 'labour stage 3' OR 'labour stage iii'/exp OR 'labour stage iii' OR 'labour stage, third'/exp OR 'labour stage, third' OR 'third delivery stage'/exp OR 'third delivery stage' OR 'third labor stage'/exp OR 'third labor stage' OR 'third labour stage'/exp OR 'third labour stage' OR 'third stage of delivery'/exp OR 'third stage of delivery' OR 'third stage of labor'/exp OR 'third stage of labor' OR 'third stage of labour'/exp OR 'third stage of labour'<br><br>#5 #1 OR #2 OR #3 OR #4<br><br>#6 'oxytocin' OR 'oxytocin'/exp OR oxytocin OR 'alpha hypophamine'/exp OR 'alpha hypophamine' OR 'atonin o'/exp OR 'atonin o' OR 'beta methyl beta mercaptobutyric acid oxytocin'/exp OR 'beta methyl beta mercaptobutyric acid oxytocin' OR 'di sipidin'/exp OR 'di sipidin' OR 'disipidin'/exp OR disipidin OR 'endopituitrin'/exp OR endopituitrin OR 'fetusin'/exp OR fetusin OR 'hypophysin'/exp OR hypophysin OR 'hypophysine'/exp OR hypophysine OR 'intravenous oxytocin'/exp OR 'intravenous oxytocin' OR 'mipareton'/exp OR mipareton OR 'neoxyn'/exp OR neoxyn OR 'ocytocin'/exp OR ocytocin OR 'opn 300'/exp OR 'opn 300' OR 'opn300'/exp OR opn300 OR 'orasthin'/exp OR orasthin OR 'orastina'/exp OR orastina OR 'oxitocin'/exp OR oxitocin OR 'oxiton inj'/exp OR 'oxiton inj' OR 'oxitone'/exp OR oxitone OR 'oxystin'/exp OR oxystin OR 'oxytan'/exp OR oxytan OR 'oxytocin 10 usp units in dextrose 5%'/exp OR 'oxytocin 10 usp units in dextrose 5%' OR 'oxytocin 20 usp units in dextrose 5%'/exp OR 'oxytocin 20 usp units in dextrose 5%' OR 'oxytocin 5 usp units in dextrose 5%'/exp OR 'oxytocin 5 usp units in dextrose 5%' OR 'oxytocin dimer'/exp OR 'oxytocin dimer' OR 'oxytocin pentapeptide'/exp OR 'oxytocin pentapeptide' OR 'oxytocin s inj'/exp OR 'oxytocin s inj' OR 'oxytocina'/exp OR oxytocina OR 'oxytocine'/exp OR oxytocine OR 'pareton'/exp OR pareton OR 'partacon'/exp OR partacon OR 'partocon'/exp OR partocon OR 'partocon inj'/exp OR 'partocon inj' OR 'partolact'/exp OR partolact OR 'partoxin'/exp OR partoxin OR 'physormon'/exp OR physormon OR 'pitocin'/exp OR pitocin OR 'pitocin inj'/exp OR 'pitocin inj' OR 'piton'/exp OR piton OR 'piton s'/exp OR 'piton s' OR 'piton s inj'/exp OR 'piton s inj' OR 'pituilobine'/exp OR pituilobine OR 'pitupartin'/exp OR pitupartin OR 'solvoxine'/exp OR solvoxine OR 'synpitan'/exp OR synpitan OR 'synthetic oxytocin inj'/exp OR 'synthetic oxytocin inj' OR 'syntocinon'/exp OR syntocinon OR 'syntocinon inj'/exp OR 'syntocinon inj' OR 'syntocinon spray'/exp OR 'syntocinon spray' OR 'ti 001'/exp OR 'ti 001' OR 'ti 114'/exp OR 'ti 114' OR 'ti001'/exp OR 'ti001' OR 'ti114'/exp OR 'ti114' OR 'tranoxy'/exp OR tranoxy OR 'tur 001'/exp OR 'tur 001' OR 'tur001'/exp OR 'tur001' OR 'utedrin'/exp OR utedrin OR 'uteracon'/exp OR uteracon OR 'uterason'/exp OR uterason OR 'utron inj'/exp OR 'utron inj' OR 'vagitocin'/exp OR vagitocin OR 'xitocin'/exp OR xitocin<br><br>#7 'oxytocin receptor'/exp OR 'oxytocin receptor' OR 'neuron, oxytocinergic'/exp OR 'neuron, oxytocinergic' OR 'oxytocinergic nerve cell'/exp OR 'oxytocinergic nerve cell' OR 'oxytocinergic neuron'/exp OR 'oxytocinergic neuron' OR 'receptor, oxytocin'/exp OR 'receptor, oxytocin' OR 'receptors, oxytocin'/exp OR 'receptors, oxytocin'<br><br>#8 'oxytocic agent'/exp OR 'oxytocic agent' OR 'labor inducing drug'/exp OR 'labor inducing drug' OR 'labour inducing drug'/exp OR 'labour inducing drug' OR 'ocytocic agent'/exp OR 'ocytocic agent' OR 'oxytocic activity'/exp OR 'oxytocic activity' OR 'oxytocic substance'/exp OR 'oxytocic substance' OR 'oxytocics'/exp OR oxytocics<br><br>#9 #6 OR #7 OR #8<br>#10 #5 AND #9<br>#11 #10 AND [embase]/lim NOT ([embase]/lim AND [medline]/lim)	
Global Index Medicus	MH:Cesárea OR MH:"Cesarean Section" OR Cesárea OR (Cesarean Section) OR (Parto Abdominal) OR (Abdominal Deliveries) OR (Abdominal Delivery) OR (C Section (OB)) OR (C-Section (OB)) OR (C-Sections (OB)) OR (Caesarean Section) OR (Caesarean Sections) OR (Cesarean Sections) OR (Postcesarean Section) OR MH:E04.520.252.500$ OR MH:"Cesárea Repetida" OR MH:"Cesarean Section, Repeat" OR MH:Recesariana OR (Cesárea Repetida) OR Recesariana OR (Repeat Cesarean Section) OR (Repeat Cesarean Sections) OR (Operación Cesárea Repetida) OR MH:E04.520.252.500.150$ OR MH:"Parto Obstétrico" OR MH:"Delivery, Obstetric" OR (Parto Obstétrico) OR (Liberação Obstétrica) OR (Obstetric Deliveries) OR (Obstetric Delivery) OR (Liberación Obstétrica) OR MH:E04.520.252$ OR MH:"Terceira Fase do Trabalho de Parto" OR MH:"Labor Stage, Third" OR MH:"Tercer Periodo del Trabajo de Parto" OR (Terceira Fase do Trabalho de Parto) OR (Tercer Periodo del Trabajo de Parto) OR (Terceiro Estágio do Trabalho de	421

	Parto) OR (Terceiro Período do Trabalho de Parto) OR (Third Labor Stage) OR (Third Labor Stages) OR (Third Stage Labor) OR MH:G08.686.784.769.326.500.110$<br>AND<br>MH:Oxitocina OR MH:Oxytocin OR MH:Ocitocina OR Oxitocina OR Oxytocin OR Ocitocina OR Ocytocin OR Pitocin OR Syntocinon OR MH:D06.472.699.631.692.433$ OR MH:D12.644.548.691.692.433$ OR MH:"Receptores de Ocitocina" OR MH:"Receptors, Oxytocin" OR MH:"Receptores de Oxitocina" OR (Receptores de Ocitocina) OR (Receptores de Oxitocina) OR (Oxytocin Receptor) OR (Oxytocin Receptors) OR MH:D12.776.543.750.695.630$ OR MH:D12.776.543.750.720.600.630$ OR MH:D12.776.543.750.750.555.630$ OR MH:D12.776.543.750.750.660.600$ OR MH:Ocitócicos OR MH:Oxytocics OR MH:Oxitócicos OR Ocitócicos OR Oxytocics OR Oxitócicos OR (Efeito Ocitócico) OR (Estimulantes Uterinos) OR (Oxytocic Agents) OR (Oxytocic Drugs) OR (Oxytocic Effect) OR (Oxytocic Effects) OR (Uterine Stimulants) OR (Efecto Oxitócico) OR MH:D27.505.696.875.737$ OR MH:D27.505.954.705.737$	
MEDLINE (via PubMed)	#1 "Cesarean Section"[Mesh] OR (Cesarean Sections) OR (Cesarean Section) OR (Delivery, Abdominal) OR (Abdominal Deliveries) OR (Abdominal Deliver) OR (Deliveries, Abdominal) OR (Abdominal Delivery) OR "C-Section (OB)" OR "C Section (OB)" OR "C-Sections (OB)" OR (Postcesarean Section) OR (cesarean delivery) OR (Cesarean Section) OR (Elective cesarean section) OR (Elective cesarean delivery) OR Cesarean OR (Emergency cesarean section) OR (Emergency cesarean delivery) OR (Intrapartum cesarean section) OR (Intrapartum cesarean delivery) OR (Intra-partum cesarean section) OR (Intra-partum cesarean delivery) OR (Intra partum cesarean section) OR (Intra partum cesarean delivery)<br><br>#2 (Caesarean Section) OR (Caesarean Sections) OR (Postcaesarean Section) OR (caesarean delivery) OR (Elective caesarean section) OR (Elective caesarean delivery) OR Caesarean OR (Emergency caesarean section) OR (Emergency caesarean delivery) OR (Intrapartum caesarean section) OR (Intrapartum caesarean delivery) OR (Intra-partum caesarean section) OR (Intra-partum caesarean delivery) OR (Intra partum caesarean section) OR (Intra partum caesarean delivery)<br><br>#3 (Prelabor cesarean section) OR (Prelabor cesarean delivery) OR (Pre-labor cesarean delivery) OR (Pre-labor cesarean delivery) OR (Pre labor cesarean section) OR (Pre labor cesarean delivery)<br><br>#4 (Prelabour cesarean section) OR (Prelabour cesarean delivery) OR (Pre-labour cesarean delivery) OR (Pre-labour cesarean delivery) OR (Pre labour cesarean section) OR (Pre labour cesarean delivery)<br><br>#5 (Prelabor caesarean section) OR (Prelabor caesarean delivery) OR (Pre-labor caesarean delivery) OR (Pre-labor caesarean delivery) OR (Pre labor caesarean section) OR (Pre labor caesarean delivery)<br>#6 (Prelabour caesarean section) OR (Prelabour caesarean delivery) OR (Pre-labour caesarean delivery) OR (Pre-labour caesarean delivery) OR (Pre labour caesarean section) OR (Pre labour caesarean delivery)<br><br>#7 "Cesarean Section, Repeat'[Mesh] OR (Repeat Cesarean Section) OR (Cesarean Sections, Repeat) OR (Repeat Cesarean Sections) OR (Section, Repeat Cesarean) OR (Sections, Repeat Cesarean)<br><br>#8 (Repeat Caesarean Section) OR (Caesarean Sections, Repeat) OR (Repeat Caesarean Sections) OR (Section, Repeat Caesarean) OR (Sections, Repeat Caesarean) OR (Obstetric anesthesia)<br><br>#9 "Delivery, Obstetric"[Mesh] OR (Deliveries, Obstetric) OR (Obstetric Deliveries) OR (Obstetric Delivery)<br><br>#10 "Labor Stage, Third"[Mesh] OR (Labor Stages, Third) OR (Stage, Third Labor) OR (Stages, Third Labor) OR (Third Labor Stage) OR (Third Labor Stages) OR (Labor, Third Stage) OR (Third Stage Labor) OR "Third Stage of Labor" OR "Third-stage labor"<br><br>#11 (Labour Stage, Third) OR (Labour Stages, Third) OR (Stage, Third Labour) OR (Stages, Third Labour) OR (Third Labour Stage) OR (Third Labour Stages) OR (Labour, Third Stage) OR (Third Stage Labour) OR "Third Stage of Labour" OR "Third-stage labour"<br><br>#12 #1 OR #2 OR #3 OR #4 OR #5 OR #6 OR #7 OR #8 OR #9 OR #10 OR #11<br><br>#13 "Oxytocin"[Mesh] OR Oxytocin OR Ocytocin OR Syntocinon OR Pitocin<br><br>#14 "Receptors, Oxytocin"[Mesh] OR (Oxytocin Receptor) OR (Receptor, Oxytocin) OR (Oxytocin Receptors) OR (alpha hypophamine) OR "atonin o" OR "beta methyl beta mercaptobutyric acid oxytocin" OR "di sipidin" OR disipidin OR endopituitrin OR fetusin OR hypophysin OR hypophysine OR (intravenous oxytocin) OR mipareton OR neoxyn OR ocytocin OR orasthin OR orastina OR oxitocin OR "oxiton inj" OR oxitone OR oxystin OR oxytan OR (oxytocin 10 usp units in dextrose 5%) OR (oxytocin 20 usp units in dextrose 5%) OR (oxytocin 5 usp units in dextrose 5%) OR (oxytocin dimer) OR (oxytocin pentapeptide) OR "oxytocin s inj" OR oxytocine OR pareton OR partacon OR partocon OR "partocon inj" OR partolact OR partoxin OR physormon OR pitocin OR "pitocin inj" OR piton OR "piton s" OR "piton s inj" OR pituilobine OR pitupartin OR solvoxine OR synpitan OR	7,944

	(synthetic oxytocin inj) OR syntocinon OR "syntocinon inj" OR (syntocinon spray) OR tranoxy OR utedrin OR uteracon OR uterason OR "utron inj" OR xitocin<br>#15 "Oxytocics"[Mesh] OR Oxytocics OR (Oxytocic Drugs) OR (Drugs, Oxytocic) OR (Uterine Stimulants) OR (Stimulants, Uterine) OR (Oxytocic Agents) OR (Agents, Oxytocic) OR (Oxytocic Effect) OR (Effect, Oxytocic) OR (Oxytocic Effects) OR (Effects, Oxytocic)<br><br>#16 "Oxytocics" [Pharmacological Action]<br>#17 #13 OR #14 OR #15 OR #16<br>#18 #12 AND #17<br>#19 ((clinical[Title/Abstract] AND trial[Title/Abstract]) OR clinical trials as topic[MeSH Terms] OR clinical trial[Publication Type] OR random*[Title/Abstract] OR random allocation[MeSH Terms] OR therapeutic use[MeSH Subheading])<br>#20 #18 AND #19	
Opengrey	Cesarean Sections) OR (Cesarean Section) OR (Delivery, Abdominal) OR (Abdominal Deliveries) OR (Abdominal Deliver) OR (Deliveries, Abdominal) OR (Abdominal Delivery) OR "C-Section (OB)" OR "C Section (OB)" OR "C-Sections (OB)" OR (Postcesarean Section) OR (cesarean delivery) OR (Cesarean Section) OR (Elective cesarean section) OR (Elective cesarean delivery) OR Cesarean OR (Emergency cesarean section) OR (Emergency cesarean delivery) OR (Intrapartum cesarean section) OR (Intrapartum cesarean delivery) OR (Intra-partum cesarean section) OR (Intra-partum cesarean delivery) OR (Intra partum cesarean section) OR (Intra partum cesarean delivery) OR (Caesarean Section) OR (Caesarean Sections) OR (Postcaesarean Section) OR (caesarean delivery) OR (Elective caesarean section) OR (Elective caesarean delivery) OR Caesarean OR (Emergency caesarean section) OR (Emergency caesarean delivery) OR (Intrapartum caesarean section) OR (Intrapartum caesarean delivery) OR (Intra-partum caesarean section) OR (Intra-partum caesarean delivery) OR (Intra partum caesarean section) OR (Intra partum caesarean delivery) OR (Prelabor cesarean section) OR (Prelabor cesarean delivery) OR (Pre-labor cesarean delivery) OR (Pre-labor cesarean delivery) OR (Pre labor cesarean section) OR (Pre labor cesarean delivery) OR (Prelabour cesarean section) OR (Prelabour cesarean delivery) OR (Pre-labour cesarean delivery) OR (Pre-labour cesarean delivery) OR (Pre labour cesarean section) OR (Pre labour cesarean delivery) OR (Prelabor caesarean section) OR (Prelabor caesarean delivery) OR (Pre-labor caesarean delivery) OR (Pre-labor caesarean delivery) OR (Pre labor caesarean section) OR (Pre labor caesarean delivery) OR (Prelabour caesarean section) OR (Prelabour caesarean delivery) OR (Pre-labour caesarean delivery) OR (Pre-labour caesarean delivery) OR (Pre labour caesarean section) OR (Pre labour caesarean delivery) OR (Repeat Cesarean Section) OR (Cesarean Sections, Repeat) OR (Repeat Cesarean Sections) OR (Section, Repeat Cesarean) OR (Sections, Repeat Cesarean) OR (Repeat Caesarean Section) OR (Caesarean Sections, Repeat) OR (Repeat Caesarean Sections) OR (Section, Repeat Caesarean) OR (Sections, Repeat Caesarean) OR (Obstetric anesthesia) OR (Deliveries, Obstetric) OR (Obstetric Deliveries) OR (Obstetric Delivery) OR (Labor Stages, Third) OR (Stage, Third Labor) OR (Stages, Third Labor) OR (Third Labor Stage) OR (Third Labor Stages) OR (Labor, Third Stage) OR (Third Stage Labor) OR "Third Stage of Labor" OR "Third-stage labor" OR (Labour Stage, Third) OR (Labour Stages, Third) OR (Stage, Third Labour) OR (Stages, Third Labour) OR (Third Labour Stage) OR (Third Labour Stages) OR (Labour, Third Stage) OR (Third Stage Labour) OR "Third Stage of Labour" OR "Third-stage labour"	126
SciELO	((Cesarean Sections) OR (Cesarean Section) OR (Abdominal Deliveries) OR (Abdominal Deliver) OR (Abdominal Delivery) OR (C-Section (OB)) OR (C Section (OB)) OR (C-Sections (OB)) OR (Postcesarean Section) OR (cesarean delivery) OR (Cesarean Section) OR (Elective cesarean section) OR (Elective cesarean delivery) OR Cesarean OR (Emergency cesarean section) OR (Emergency cesarean delivery) OR (Intrapartum cesarean section) OR (Intrapartum cesarean delivery) OR (Intra-partum cesarean section) OR (Intra-partum cesarean delivery) OR (Intra partum cesarean section) OR (Intra partum cesarean delivery) OR (Caesarean Section) OR (Caesarean Sections) OR (Postcaesarean Section) OR (caesarean delivery) OR (Elective caesarean section) OR (Elective caesarean delivery) OR Caesarean OR (Emergency caesarean section) OR (Emergency caesarean delivery) OR (Intrapartum caesarean section) OR (Intrapartum caesarean delivery) OR (Intra-partum caesarean section) OR (Intra-partum caesarean delivery) OR (Intra partum caesarean section) OR (Intra partum caesarean delivery) OR (Prelabor cesarean section) OR (Prelabor cesarean delivery) OR (Pre-labor cesarean delivery) OR (Pre-labor cesarean delivery) OR (Pre labor cesarean section) OR (Pre labor cesarean delivery) OR (Prelabour cesarean section) OR (Prelabour cesarean delivery) OR (Pre-labour cesarean delivery) OR (Pre-labour cesarean delivery) OR (Pre labour cesarean section) OR (Pre labour cesarean delivery) OR (Prelabor caesarean section) OR (Prelabor caesarean delivery) OR (Pre-labor caesarean delivery) OR (Pre-labor caesarean delivery) OR (Pre labor caesarean section) OR (Pre labor caesarean delivery) OR (Prelabour caesarean section) OR (Prelabour caesarean delivery) OR (Pre-labour caesarean delivery) OR (Pre-labour caesarean delivery) OR (Pre labour caesarean section) OR (Pre labour caesarean delivery) OR (Repeat Cesarean Section) OR (Repeat Cesarean Sections) OR (Repeat Caesarean Section) OR (Repeat Caesarean Sections) OR (Obstetric anesthesia) OR (Obstetric Deliveries) OR (Obstetric Delivery) OR (Third Labor Stage) OR (Third Labor Stages) OR (Third Stage Labor) OR (Third Stage of Labor) OR (Third-stage labor) OR (Third Labour Stage) OR (Third Labour Stages) OR (Third Stage Labour) OR (Third Stage of Labour) OR (Third-stage labour))<br>AND<br>(Oxytocin OR Ocytocin OR Syntocinon OR Pitocin OR (Oxytocin Receptor) OR (Oxytocin Receptors) OR (alpha hypophamine) OR (atonin o) OR (beta methyl beta mercaptobutyric acid oxytocin) OR (di sipidin) OR disipidin OR endopituitrin OR fetusin OR hypophysin OR hypophysine OR (intravenous oxytocin) OR mipareton OR neoxyn OR ocytocin OR orasthin OR orastina OR oxitocin OR (oxiton inj) OR oxitone OR oxystin OR oxytan OR (oxytocin 10 usp units in dextrose 5%) OR (oxytocin 20 usp	91

	units in dextrose 5%) OR (oxytocin 5 usp units in dextrose 5%) OR (oxytocin dimer) OR (oxytocin pentapeptide) OR (oxytocin s inj) OR oxytocine OR pareton OR partacon OR partocon OR (partocon inj) OR partolact OR partoxin OR physormon OR pitocin OR (pitocin inj) OR piton OR (piton s) OR (piton s inj) OR pituilobine OR pitupartin OR solvoxine OR synpitan OR (synthetic oxytocin inj) OR syntocinon OR (syntocinon inj) OR (syntocinon spray) OR tranoxy OR utedrin OR uteracon OR uterason OR (utron inj) OR xitocin OR Oxytocics OR (Oxytocic Drugs) OR (Uterine Stimulants) OR (Oxytocic Agents) OR (Oxytocic Effect) OR (Oxytocic Effects))	
WHO - ICTRP	Condition: (Cesarean Sections) OR (Cesarean Section) OR (Delivery, Abdominal) OR (Abdominal Deliveries) OR (Abdominal Deliver) OR (Deliveries, Abdominal) OR (Abdominal Delivery) OR "C-Section (OB)" OR "C Section (OB)" OR "C-Sections (OB)"<br><br>Intervention: Oxytocin OR Ocytocin OR Syntocinon OR Pitocin OR (Oxytocin Receptor) OR (Receptor, Oxytocin) OR (Oxytocin Receptors) OR (alpha hypophamine) OR "atonin o" OR "beta methyl beta mercaptobutyric acid oxytocin" OR "di sipidin" OR disipidin	95
Total		16,883
