# Supplementary material for: Route of oxytocin administration for preventing blood loss at caesarean section: a systematic review with meta-analysis
Source: BMJ Open. 2021 Sep 16;11(9):e051793. doi: 10.1136/bmjopen-2021-051793 (PMC8449971; doi:10.1136/bmjopen-2021-051793)
Supplement: Supplementary data [file bmjopen-2021-051793supp003.pdf]

Supplementary file 3. Excluded studies in systematic review on route of oxytocin administration

Excluded studies with reasons

Study	*Reason for exclusion*
Zarzur E. A Ocitocina e a Operação Cesariana. Rev Bras Anest. 1992;42(4):293-5.	Did not compare different routes
Garza-Hinojosa A, González-Cordero G. Cambios hemodinámicos y electrocardiográfi cos con el uso de oxitocina en bolo durante la cesárea. Ver Mex Anest. 2015;38(S1): S257-62.	Did not compare different routes
Singh KP, Kameshore N, Kamei H. Prophylactic intramuscular injection of oxytocin vs intravenous infusion of oxytocin to minimize blood loss at caesarean section. Int J Gynecol Obstet. 2015;131(Suppl. 5): E290.	Insufficient information (Abstract- authors did not reply to contact)
