# Supplementary material for: Route of oxytocin administration for preventing blood loss at caesarean section: a systematic review with meta-analysis
Source: BMJ Open. 2021 Sep 16;11(9):e051793. doi: 10.1136/bmjopen-2021-051793 (PMC8449971; doi:10.1136/bmjopen-2021-051793)
Supplement: Supplementary data [file bmjopen-2021-051793supp004.pdf]

Supplementary file 4. Details of trials on different routes of oxytocin administration at CS

Akinaga 2016	
**Method**	Double blind, placebo controlled RCT<br>Study protocol UMIN000007577 (https://upload.umin.ac.jp/cgi-open-bin/ctr_e/ctr_view.cgi?recptno=R000008709)
**Participants**	**Setting**: women managed at Hamamatsu University hospital, Japan<br>**Dates of recruitment**: April to October 2012<br>**Inclusion criteria**: 40 women, ASA status I or II with a single fetus at ≥36 weeks scheduled for elective CS under spinal anesthesia<br>**Exclusion criteria**: conditions predisposing to uterine atony and postpartum hemorrhage, such as placenta previa, multiple gestations, polyhydramnios, previous uterine atony and post-partum bleeding, macrosomia, uterine fibroids, bleeding diathesis, and pre-eclampsia
**Interventions**	After clamping of umbilical cord<br>**Group 1** (intramyometrial): bolus injection of oxytocin (0.07 IU/kg) diluted to 2 mL saline was slowly (over 30 seconds) injected into the myometrium of the uterine fundus and an IV saline infusion (10 mL) was also given.<br>(n=20 randomized)<br>**Group 2**: (intravenous): 2 mL bolus saline injection was injected into the myometrium and an IV oxytocin injection (0.07 IU/kg) diluted to 10 mL with saline was also given.<br>(n=20 randomized)<br>**Both groups:** After bolus injections, both groups received oxytocin at 0.1 IU/kg in 500 mL of lactated Ringer's solution and continuously administered IV at a rate of 100 mL/h.
**Outcomes**	Volume of blood loss during CS (volume in suction bottle). Additional uterotonics. Adverse effects (hypotension, nausea/vomiting, headache, flushing)
**Notes**	Funding: not reported<br>Conflict of interest: none

Dennehy 1998	
**Method**	Double blind, placebo controlled RCT<br>no protocol
**Participants**	**Setting**: women managed in hospital (no other information) in Canada<br>**Dates of recruitment**: No information<br>**Inclusion criteria**: 40 ASA I or II women with singleton fetus scheduled for elective CS at term (> 37 wks).<br>**Exclusion criteria**: age <18 yr, contraindication to spinal anaesthesia, polyhydramnios, placenta praevia, uterine fibroids, hypertension or cardiac disease.
**Interventions**	Immediately after placental removal:<br>**Group 1** (IV bolus): 0.5 ml of 10 IU/ml oxytocin in unknown time and an intramyometrial 2 ml saline injection. Total bolus injection dose: 5 IU, Total bolus injection time: NI.<br>(n=20 randomized)<br>**Group 2** (Intramyometrial bolus): 2 ml of 10 IU/ml oxytocin into the uterine fundus and 0.5 mL saline IV bolus. Total bolus injection dose: 20 IU, Total bolus injection time: NI.<br>(n=20 randomized)
**Outcomes**	Need for additional uterotonics, hypotension
**Notes**	Funding: not reported<br>Conflict of interest: none

Mangla 2012	
**Method**	Double blind RCT<br>No protocol.
**Participants**	**Setting**: Guru Gobind Singh Hospital, Jamnagar, Gujarat, India<br>**Dates of recruitment**: No information<br>**Inclusion criteria:** "women operated by lower segment cesarean section". No other information<br>**Exclusion criteria:** No information
**Interventions**	**Group 1**: IV infusion 20 IU/500 ml Ringer after placental detachment, no information on rate or duration of infusion<br>(n=50 randomized).<br>**Group 2**: Intra-myometrial 5 IU in 10 cc normal saline; 5 cc injected in each cornu of the uterus after separation of placenta.<br>(n=50 randomized)<br>**Group 3**: Intra-myometrial 5 IU in 10 cc normal saline; 5 cc injected in each cornu of the uterus before separation of placenta.<br>(n=50 randomized) This group was not included in the SR because it was identical to Group 2 but given at a different timing
**Outcomes**	Volume of blood loss during CS and 1st hour after (measured by adding volume in graduated suction jar and number of soaked sponges; no information on how blood loss was measured in the 1st hour after).<br>Additional uterotonic. Nausea/vomiting requiring antiemetic.
**Notes**	Funding: none<br>Conflict of interest: none

CS: cesarean section, IU: international units, IV: intravenous, RCT: randomized controlled trial
