# Supplementary material for: Route of oxytocin administration for preventing blood loss at caesarean section: a systematic review with meta-analysis
Source: BMJ Open. 2021 Sep 16;11(9):e051793. doi: 10.1136/bmjopen-2021-051793 (PMC8449971; doi:10.1136/bmjopen-2021-051793)
Supplement: Supplementary data [file bmjopen-2021-051793supp005.pdf]

Supplementary file 5: Postpartum hemorrhage prevention core outcome sets[1] in trials on different routes of oxytocin administration at cesarean section

Outcome		Measure (in each group)	Study		
			Akinaga 2016	Dennehy 1998	Mangla 2012
1	Blood loss (from birth up to cessation of active bleeding)				
	≥ 1000 ml	N women with this loss	no	no	no
	total volume	mean or median volume	yes	no	yes
2	Shock (defined by trialists)	N women	no	no	no
3	Maternal death				
	PPH-related maternal deaths	N women	no	no	no
	all cause mortality	N women	no	no	no
4	Blood transfusion				
	Wm receiving any RBC product	N women	no	no	no
	total volume	mean or median RBC units	no	no	no
5	Transfer to higher level of care	N women	no	no	no
6	Use of additional uterotonics	N women	yes	yes	yes
7	Adverse effects (defined by trialists)	N women	yes (headache, nausea/vomiting, flushing, hypotension)	yes (hypotension)	yes (nausea/ vomiting)
8	Breastfeeding	N women	no	no	no
9	Patient reported outcomes	N women	no	no	no

N: number, PPH: post-partum hemorrhage, RBC: red blood cell

1.Source: Meher S, Cuthbert A, Kirkham JJ, et al. Core outcome sets for prevention and treatment of postpartum haemorrhage: an international Delphi consensus study. BJOG. 2019 Jan;126(1):83-93.
