# Supplementary material for: Route of oxytocin administration for preventing blood loss at caesarean section: a systematic review with meta-analysis
Source: BMJ Open. 2021 Sep 16;11(9):e051793. doi: 10.1136/bmjopen-2021-051793 (PMC8449971; doi:10.1136/bmjopen-2021-051793)

Supplementary file 6. Risk of bias details

Judgments and justifications for risk of bias assessments in trials on different routes of oxytocin administration at cesarean section

Domain	Akinaga 2016		
1	Sequence generation	UNCLEAR	No information on random sequence generation
2	Allocation concealment	LOW	*'An anesthesiologist unrelated to this research selected one of two opaque envelopes containing a letter indicating the group name'*
3	Blinding participants/personnel (objective outcomes)	LOW	*'The subject, care providers, and investigators were blinded to patient allocation until all data collection was completed.'*
3	Blinding participants/personnel (subjective outcomes)	LOW	*'The subject, care providers, and investigators were blinded to patient allocation until all data collection was completed.'*
4	Blinding outcome assessors (objective outcomes)	LOW	*'The i.m.y. group received a bolus injection of oxytocin (0.07 IU/kg) diluted to 2 mL with saline into the myometrium of the uterine fundus during slow (30-s) i.v. infusion of saline (10 mL). Conversely, the i.v. group received a 2-mL bolus injection of saline into the myometrium during slow (30-s) i.v. infusion of oxytocin (0.07 IU/kg) diluted to 10mL with saline.'*
4	Blinding outcome assessors (subjective outcomes)	LOW	*The subject, care providers, and investigators were blinded to patient allocation until all data collection was completed.'*
5	Incomplete outcome	HIGH	*´Nine patients were excluded from the study: eight for protocol deviation and one for adenomyosis.'* High rate of loss (20%), with no information about which was the original group that the participants had been randomized to. ITT was not used.
6	Selective reporting	LOW	All outcomes described in protocol were reported
7	Other bias	LOW	There are probably no other sources of bias

Domain	Dennehy 1998		
1	Sequence generation	LOW	*´Participants were assigned to one of two study groups, according to a computer generated series of random numbers.'*
2	Allocation concealment	UNCLEAR	No information on allocation concealment
3	Blinding participants/personnel (objective outcomes)	LOW	*'The hospital pharmacy prepared the syringes containing the study solutions trader aseptic conditions according to the random table and delivered them to the delivery room in a plastic bag.'*
3	Blinding participants/personnel (subjective outcomes)	does not apply	No subjective outcomes
4	Blinding outcome assessors (objective outcomes)	LOW	*´The injections were conducted in a double blind fashion where neither the attending anaesthetist or obstetrician had knowledge of the content of the syringes.*'
4	Blinding outcome assessors (subjective outcomes)	does not apply	No subjective outcomes
5	Incomplete outcome	LOW	*One patient did not complete the study protocol because the attending anaesthetist was unable to locate the subarachnoid space and she, therefore, received general anaesthesia for Caesarean section*'. Low rate of losses (2%).
6	Selective reporting	UNCLEAR	No study protocol
7	Other bias	LOW	There are probably no other sources of bias

Domain	Mangla 2012		
1	Sequence generation	UNCLEAR	No information on random sequence generation
2	Allocation concealment	UNCLEAR	No information on allocation concealment
3	Blinding participants/personnel (objective outcomes)	LOW	Blinding of participants was unconfirmed. Objective outcomes are unlikely to be influenced by this fact.
3	Blinding participants/personnel (subjective outcomes)	HIGH	Blinding of participants and personnel was unconfirmed. Subjective outcomes are likely to be influenced by this fact.
4	Blinding outcome assessors (objective outcomes)	LOW	Blinding of outcome assessors was unconfirmed. The judgment of objective outcomes is unlikely to be influenced by this fact.
4	Blinding outcome assessors (subjective outcomes)	HIGH	Blinding of participants and personnel was unconfirmed. Subjective outcomes are likely to be influenced by this fact.
5	Incomplete outcome	LOW	The outcome data of all randomized participants were presented
6	Selective reporting	UNCLEAR	No study protocol
7	Other bias	UNCLEAR	No information on baseline characteristics

Supplementary file 6, continuation

Figure S6. Risk of bias graph: review authors' judgements about each risk of bias item presented as percentages across all included studies.

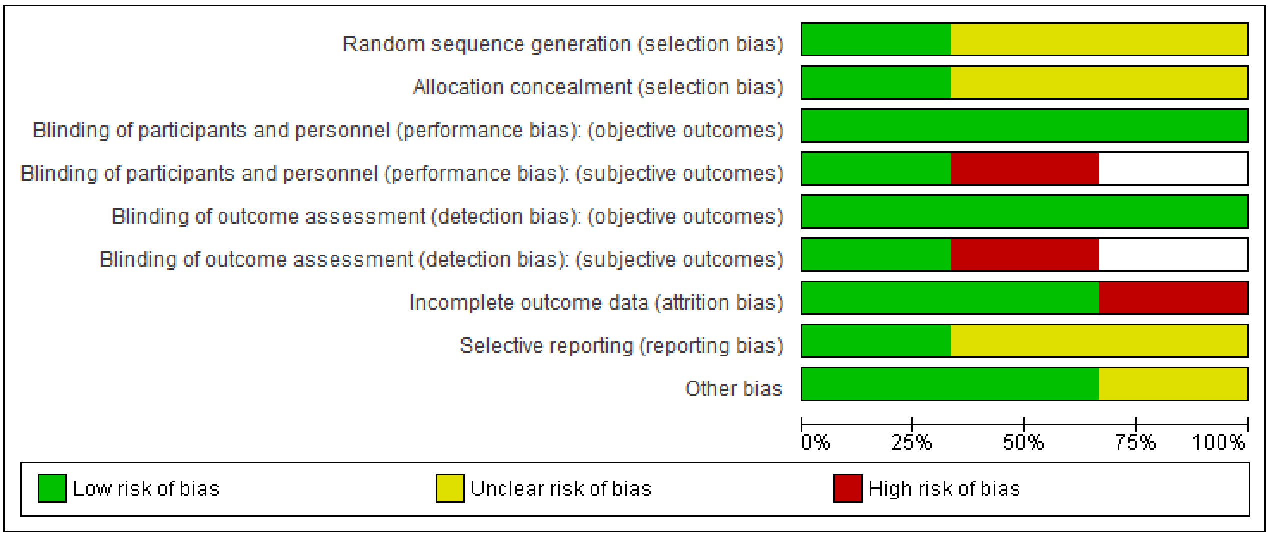

Supplement: Supplementary data [file bmjopen-2021-051793supp006.pdf]
