# Supplementary material for: Route of oxytocin administration for preventing blood loss at caesarean section: a systematic review with meta-analysis
Source: BMJ Open. 2021 Sep 16;11(9):e051793. doi: 10.1136/bmjopen-2021-051793 (PMC8449971; doi:10.1136/bmjopen-2021-051793)
Supplement: Supplementary data [file bmjopen-2021-051793supp007.pdf]

Supplementary file 7. Summary of findings table and GRADE: intramyometrial versus intravenous oxytocin at cesarean section

Intramyometrial compared to Intravenous prophylactic oxytocin for women giving birth by cesarean

Patient or population: women giving birth by cesarean
Setting: hospital
Intervention: intramyometrial oxytocin
Comparison: intravenous oxytocin

Outcomes	Anticipated absolute effects* (95% CI)		Relative effect (95% CI)	N of participants (studies)	Certainty of the evidence (GRADE)	Comments
	Risk with Intravenous prophylactic oxytocin	Risk with Intramyometrial				
Post-Partum Hemorrhage	60 per 1000	**8 per 1000** (1 to 162)	**RR 0.14** (0.01 to 2.70)	100 (1 RCT)	⨁⨁◯◯ LOW [a]	Mangla 2012: Blood loss > 900 mL.
Need for additional uterotonic	71 per 1000	**59 per 1000** (18 to 192)	**RR 0.82** (0.25 to 2.69)	140 (2 RCTs)	⨁◯◯◯ VERY LOW [a,b]	
Hypotension not requiring epinephrine	200 per 1000	**200 per 1000** (58 to 690)	**RR 1.00** (0.29 to 3.45)	40 (1 RCT)	⨁⨁◯◯ LOW [a]	
Hypotension requiring ephedrine	See comment	See comment	Not estimable	79 (2 RCTs)	⨁⨁◯◯ LOW [c]	The 2 trials had no events.
Nausea and/or Vomiting	157 per 1000	**20 per 1000** (3 to 108)	**RR 0.13** (0.02 to 0.69)	140 (2 RCTs)	⨁⨁◯◯ LOW [d,e]	
Headache	0 per 1000	**0 per 1000** (0 to 0)	**RR 3.00** (0.13 to 69.52)	40 (1 RCT)	⨁⨁◯◯ LOW [a]	
Facial flushing	100 per 1000	**50 per 1000** (5 to 508)	**RR 0.50** (0.05 to 5.08)	40 (1 RCT)	⨁⨁◯◯ LOW [a]	
Volume of blood loss	The mean volume of blood loss was **664.2** mL	MD **57.40 mL lower** (101.71 lower to 13.09 lower)	-	40 (1 RCT)	⨁⨁◯◯ LOW [f]	

***The risk in the intervention group** (and its 95% confidence interval) is based on the assumed risk in the comparison group and the **relative effect** of the intervention (and its 95% CI).
CI: Confidence interval; **RR:** Risk ratio; **MD:** Mean difference

GRADE Working Group grades of evidence
High certainty: We are very confident that the true effect lies close to that of the estimate of the effect
Moderate certainty: We are moderately confident in the effect estimate: The true effect is likely to be close to the estimate of the effect, but there is a possibility that it is substantially different
Low certainty: Our confidence in the effect estimate is limited: The true effect may be substantially different from the estimate of the effect
Very low certainty: We have very little confidence in the effect estimate: The true effect is likely to be substantially different from the estimate of effect

Explanations

a. Evidence certainty downgraded -2 due to very serious imprecision (very small number of events, and wide 95% CI crossing the line of no effect).
b. Evidence certainty downgraded -1 due to inconsistency (I2 61%)
c. Evidence certainty downgraded -2 due to very serious imprecision (lack of events).
d. Evidence certainty downgraded -1 due to study limitation (lack of blinding of participants, subjective outcome)
e. Evidence certainty downgraded -1 due to serious imprecision (small sample size)
f. Evidence certainty downgraded -2 due to very serious imprecision (small sample size and wide 95% confidence interval).
