# Supplementary material for: Route of oxytocin administration for preventing blood loss at caesarean section: a systematic review with meta-analysis
Source: BMJ Open. 2021 Sep 16;11(9):e051793. doi: 10.1136/bmjopen-2021-051793 (PMC8449971; doi:10.1136/bmjopen-2021-051793)
Supplement: Author's manuscript [file bmjopen-2021-051793.draft_revisions.pdf]

BMJ Open is committed to open peer review. As part of this commitment we make the peer review history of every article we publish publicly available.

When an article is published we post the peer reviewers' comments and the authors' responses online. We also post the versions of the paper that were used during peer review. These are the versions that the peer review comments apply to.

The versions of the paper that follow are the versions that were submitted during the peer review process. They are not the versions of record or the final published versions. They should not be cited or distributed as the published version of this manuscript.

BMJ Open is an open access journal and the full, final, typeset and author-corrected version of record of the manuscript is available on our site with no access controls, subscription charges or pay-per-view fees (http://bmjopen.bmj.com).

If you have any questions on BMJ Open's open peer review process please email info.bmjopen@bmj.com

Route of oxytocin administration to prevent blood loss at caesarean section: a systematic review with metanalysis

Journal:	*BMJ Open*
Manuscript ID	bmjopen-2021-051793
Article Type:	Original research
Date Submitted by the Author:	28-Mar-2021
Complete List of Authors:	Torloni, Maria Regina; Hospital e Maternidade Santa Joana, Obstetrics Department; Universidade Federal de Sao Paulo Escola Paulista de Medicina, Evidence Based Healthcare Post-Graduation Program<br>Siaulys, Monica; Hospital e Maternidade Santa Joana, Anaesthesiology Department<br>Riera, Rachel; Hospital Sirio-Libanes, Centre of Health Technology Assessment; Universidade Federal de Sao Paulo Escola Paulista de Medicina, Evidence Based Healthcare Post-Graduation Program<br>Martimbianco, Ana ; Universidade Metropolitana de Santos; Universidade Federal de Sao Paulo Escola Paulista de Medicina, Evidence Based Healthcare Post-Graduate program<br>Pacheco, Rafael; Centro Universitário São Camilo; Universidade Federal de Sao Paulo Escola Paulista de Medicina, Evidence Based Healthcare Post-Graduation Program<br>Latorraca, Carolina ; Universidade Federal de Sao Paulo Escola Paulista de Medicina, Evidence Based Healthcare Post-Graduate program<br>Widmer, Mariana; World Health Organization, Reproductive Health and Research<br>Betran, Ana Pilar; World Health Organization, Reproductive Health and Research
Keywords:	Maternal medicine < OBSTETRICS, THERAPEUTICS, Adverse events < THERAPEUTICS

SCHOLARONE™
Manuscripts

I, the Submitting Author has the right to grant and does grant on behalf of all authors of the Work (as defined in the below author licence), an exclusive licence and/or a non-exclusive licence for contributions from authors who are: i) UK Crown employees; ii) where BMJ has agreed a CC-BY licence shall apply, and/or iii) in accordance with the terms applicable for US Federal Government officers or employees acting as part of their official duties; on a worldwide, perpetual, irrevocable, royalty-free basis to BMJ Publishing Group Ltd ("BMJ") its licensees and where the relevant Journal is co-owned by BMJ to the co-owners of the Journal, to publish the Work in this journal and any other BMJ products and to exploit all rights, as set out in our licence.

The Submitting Author accepts and understands that any supply made under these terms is made by BMJ to the Submitting Author unless you are acting as an employee on behalf of your employer or a postgraduate student of an affiliated institution which is paying any applicable article publishing charge ("APC") for Open Access articles. Where the Submitting Author wishes to make the Work available on an Open Access basis (and intends to pay the relevant APC), the terms of reuse of such Open Access shall be governed by a Creative Commons licence – details of these licences and which Creative Commons licence will apply to this Work are set out in our licence referred to above.

Other than as permitted in any relevant BMJ Author's Self Archiving Policies, I confirm this Work has not been accepted for publication elsewhere, is not being considered for publication elsewhere and does not duplicate material already published. I confirm all authors consent to publication of this Work and authorise the granting of this licence.

For peer review only

Route of oxytocin administration to prevent blood loss at caesarean section: a systematic review with metanalysis

Maria Regina Torloni[1,2], Monica Siaulys[1], Rachel Riera[2,3], Ana Luiza Cabrera Martimbianco[2,4], Rafael Leite Pacheco[2,5], Carolina de Oliveira Cruz Latorraca[2], Mariana Widmer[6], Ana Pilar Betrán[6].

1. Hospital e Maternidade Santa Joana
Rua Dr. Eduardo Amaro 225, São Paulo, SP, CEP 04104-080, Brazil.

2. Evidence Based Healthcare Post-Graduation Program, São Paulo Federal University-UNIFESP, Rua Botucatu 740, 5º andar, São Paulo, SP, CEP 04023-900, Brazil

3. Centre of Health Technology Assessment, Hospital Sirio-Libanês,
Rua Dona Adma Jafet, 91, São Paulo, SP, CEP 01308-050, Brazil

4. Universidade Metropolitana de Santos (UNIMES)
Av. Gen. Francisco Glicério 8, Santos, SP, CEP 11045-002, Brazil

5. Centro Universitário São Camilo
Av. Nazaré, 1501, São Paulo, SP, CEP 04263-200

6. Department of Reproductive Health and Research, World Health Organization, UNDP/UNFPA/UNICEF/WHO/World Bank Special Programme of Research, Development and Research Training in Human Reproduction (HRP),
Avenue Appia 20, 1201 Geneva, Switzerland.

Corresponding author:
Maria Regina Torloni, email: ginecologia@terra.com.br

Word count: 3797

For peer review only

Abstract

Objectives: Assess the effects of different routes of prophylactic oxytocin administration in women having a caesarean section (CS). **Design:** Systematic review and metanalysis. **Methods.** Medline, EMBASE, CINAHL, Cochrane Library, BVS, SciELO, and Global Index Medicus were searched through 24/05/2020 for randomised controlled trials (RCT) comparing different routes of prophylactic oxytocin administration during CS. Study selection, data extraction, and quality assessment were conducted by two investigators independently. We pooled results in random effects metanalyses and calculated average risk ratio (RR), mean difference (MD), and 95% confidence intervals (CI). We used GRADE to assess the overall quality of evidence for each outcome. **Results:** Three trials (180 women) were included in the review. All studies compared intramyometrial (IMY) versus intravenous (IV) oxytocin in women having pre-labour CS. IMY compared to IV oxytocin administration may result in little or no difference in the incidence of post-partum haemorrhage (RR 0.14, 95% CI 0.01 to 2.70; N=100 participants; 1 RCT), hypotension (RR 1.00, 95% CI 0.29 to 3.45; N=40; 1 RCT), headache (RR 3.00, 95% CI 0.13 to 69.52; N=40; 1 RCT) or facial flushing (RR 0.50, 95% CI 0.05 to 5.08; N=40; 1 RCT); IMY oxytocin may reduce nausea/vomiting (RR 0.14, 95% CI 0.03 to 0.78; N=140; 2 RCTs). We are very uncertain about the effect IMY versus IV oxytocin on the need for additional uterotonics (RR 0.71; 95% CI 0.05 to 9.73; N=179; 3 RCTs). IMY oxytocin may reduce blood loss slightly (MD -57.40 mL, 95% CI -13.09 to -101.71; N=40; 1 RCT). **Conclusions:** There is limited, low to very low certainty evidence on the effects of two routes of prophylactic oxytocin administration at CS. At present, there is insufficient evidence to recommend IMY or IV oxytocin administration at CS to prevent blood loss. More trials are needed on this relevant question.

Prospero: CRD186797

Keywords: Postpartum Hemorrhage, Cesarean section, Oxytocin, Drug Administration Routes, Systematic Review, Meta-Analysis

Article summary: Strengths and Limitations

- This is the first systematic review to assess the effects of different routes of oxytocin administration to prevent bleeding in women giving birth by caesarean.
- We performed an extensive search without language restrictions in 10 databases to identify relevant trials, and followed strict methodology throughout the systematic review process.
- We found few trials, involving a small number of participants, and addressing a limited number of outcomes.
- The trials did not test all possible routes of oxytocin administration, and only included pre-labour caesareans at term.
- The overall certainty of the evidence was low or very low for all outcomes due mainly to imprecision.

For peer review only

INTRODUCTION

Deliveries by caesarean section (CS) have increased worldwide over the last three decades, including in low- and middle-income countries (LMIC)[1]. Even in optimal conditions, women who have a CS lose more blood, and have a higher risk for post-partum haemorrhage (PPH) than those who give birth by vaginal delivery (VD)[2 3]. PPH is an important cause of maternal morbidity, including prolonged hospital stay, blood transfusion and hysterectomy, and it is the leading cause of maternal mortality worldwide[4-6]. Uterine atony is responsible for 50% to 80% of all cases of PPH[4 6].

Routine administration of prophylactic uterotonics soon after birth reduces the incidence of PPH and its associated complications[6]. Oxytocin remains the first-line agent to prevent PPH recommended by the World Health Organization (WHO) because of its lack of major contraindications, similar efficacy, and lower cost and incidence of side effects, compared to other uterotonics[6]. For women having a VD, a systematic review in 2020 showed that intravenous (IV) oxytocin is more effective than intramuscular (IM) administration to reduce PPH, severe PPH, blood transfusion, and severe maternal morbidity, with no clear differences in adverse events[7]. Based on these findings, WHO updated its guideline in 2020 and now recommends that women giving birth by VD who already have endovenous access should receive a slow IV injection of 10 IU of oxytocin, in preference to IM administration[8]. For women giving birth by CS, due to the lack of specific evidence, WHO currently recommends 10 IU of oxytocin without preference for the IV or IM routes[8]. Other guidelines provide little, unclear, or no recommendations on the route of administration of prophylactic oxytocin at CS[9-11].

Since all women having a CS will have an endovenous access, IV administration of oxytocin seems logical. However, due to concerns about the cardiovascular side-effects of the drug in women undergoing a major abdominal surgery, many studies have investigated different dosages and regimens of IV oxytocin administration at CS in search of the optimal method to safely prescribe this uterotonic[12-17]. After IV injection in late pregnancy, oxytocin has an almost immediate onset of action but a half-life of only 5-12 minutes due to its rapid hepatic and renal degradation[17-19]. With continued infusion, oxytocin reaches a steady state plateau in the plasma in approximately 40 minutes[17-19]. Activation of oxytocin receptors in the myometrium stimulates prostaglandin synthesis and increases intracellular calcium in myocytes which leads to strong uterine contractions thus reducing blood loss from the placental site[16-19]. However, the simultaneous activation of oxytocin receptors in the cardiovascular system causes decreased systemic vascular resistance, hypotension, tachycardia, and coronary vasoconstriction with the attendant symptoms of skin flushing, headache, nausea, vomiting, and chest pain[17 20 21]. These changes are usually transient, depend on the dose and speed of IV oxytocin administration, and are tolerated by most healthy young women[16 22]. However, in some women undergoing a CS, these haemodynamic changes can be potentially dangerous and even life-threatening because of the sympathetic nervous blockade secondary to regional anaesthesia, the concomitant use of other vasoactive drugs, excessive blood loss, and the underlying clinical and obstetric disorders that may have led to the CS indication (e.g. cardiac disease, hypertensive disorders, placenta

For peer review only

previa)[21 23-25]. Due to its antidiuretic properties, oxytocin administration can also lead to water intoxication resulting in pulmonary oedema, seizures, coma and even death, especially in women who receive large volumes of fluid[13 17-19]. The routine use of large IV fluid preloads during CS under regional anaesthesia can potentially increase the risk of this complication[26].

After IM oxytocin injection, uterine response occurs within 3-5 minutes and can last for up to 2-3 hours[8 18 19 27]. Direct intramyometrial (IMY) injection of uterotonics can also promote uterine contraction[28 29]. IMY oxytocin injection is believed to promote immediate uterine contraction by local effect and by drug absorption from the myometrium into the systemic circulation[28 30-32]. Although oxytocin is not licensed for IMY use[18 19], this route of administration has been tested to prevent PPH at CS since 1990[28 30-32].

The route of administration of prophylactic oxytocin at CS can potentially affect the volume of maternal blood loss as well as the incidence of drug-related adverse effects. Since the side-effects of oxytocin are directly related to dose and rate of administration[13 17-19 27], in theory, the IM or IMY routes could lead to fewer cardiovascular side-effects than the IV route. If the efficacy of IM and IMY prophylactic oxytocin during CS is similar to IV administration, and if these routes have fewer side-effects than the endovenous route, this could challenge the preference for IV administration. Several randomised trials have compared different routes of prophylactic oxytocin administration at CS, but there are no systematic reviews on this topic. It is important to assess the balance between the effectiveness and adverse effects of the various routes of prophylactic oxytocin administration at CS because this information is essential to underpin evidence-based recommendations on the prevention of PPH in women given birth by CS.

OBJECTIVES

To assess the effects of administrating prophylactic oxytocin using different routes in women undergoing a CS.

METHODS

We conducted this review following Cochrane methods[33] and reported it according to the PRISMA statement[34]. We registered the protocol prospectively (CRD 186797- Supplementary file 1).

Patient and public involvement

There was no patient or public involvement in this study.

Types of studies

We included randomised clinical trials with a parallel design. Abstracts were eligible if they provided sufficient outcome data and methodological details for quality assessment.

Types of participants

For peer review only

Study participants were women having a primary or repeat CS, before or during labour, at any gestational age and for any indication, regardless of previous exposure to oxytocin (for labour induction or augmentation). Studies with women at high or low baseline risk for PPH, with or without comorbidities, and with singleton or multiple pregnancies were eligible for inclusion. We included studies involving women with both routes of delivery only if authors provided separate data for those giving birth by CS.

Types of interventions

We included studies that compared different routes of oxytocin administration (IV, IM or IMY) given alone, at any moment during CS (pre-delivery, after delivery, or after placental extraction). We excluded studies that administered oxytocin associated with other drugs to prevent PPH.

Outcomes

Studies that reported at least one of our outcomes of interest were included. Our outcomes were based on the PPH prevention core outcome set created by the CROWN initiative[35]. The primary outcomes were PPH $\geq$ 1000 mL (measured objectively or subjectively), use of additional uterotonics, and any immediate adverse effects possibly related to oxytocin (e.g. nausea/vomiting, facial flushing, headache, hypotension, cardiac arrhythmia). The secondary outcomes were total volume of blood loss at CS, need for transfusion, maternal transfer to higher level of care, shock, severe maternal morbidity (admission to ICU, hysterectomy, coma or organ failure), maternal mortality related to PPH, breastfeeding at hospital discharge, and maternal satisfaction.

Search strategy

The search strategy was developed by an experienced information specialist. It was adapted and run in seven electronic databases (MEDLINE, Embase, Cochrane Library, CINAHL, BVS, Global Index Medicus, SciELO) and two trial registry platforms (ClinicalTrials.gov and WHO – ICTRP) (Supplementary file 2). We searched for unpublished studies (grey literature) in Opengrey (https://opengrey.eu). We screened the reference lists of all retrieved studies and relevant systematic reviews to identify additional potentially eligible trials not captured by the electronic searches. There were no language or publication status restrictions. Searches were run on May 24, 2020.

Process of study selection, data extraction and risk of bias assessment

All references were uploaded in the Rayyan platform[36] and duplicates were excluded. All titles and abstracts were screened independently by two review authors. Potentially eligible studies were selected for full text reading; those that fulfilled the selection criteria were included in the review. Two review authors extracted data independently and a third author check data for accuracy. Two review authors independently assessed the quality of included studies using the Cochrane Risk of Bias tool[33]. In each study, authors assessed and graded seven domains as being at high, low, or unclear risk of bias. Four domains (random sequence generation, allocation concealment, selective reporting, and other source of bias) were

For peer review only

assessed at study level; three domains (blinding of participants and personnel, blinding of outcome assessment, and incomplete outcome data) were assessed at outcome-level. Any disagreements in the process of study selection, data extraction, or risk of bias assessment were solved by a third senior review author.

Data analyses and assessment of the certainty of the evidence

We planned to conduct three comparisons between routes of oxytocin administration: IV vs IM, IV vs IMY, and IM vs IMY. All analyses were conducted on an intention-to-treat basis. When data were missing, we contacted trial authors. Where metanalyses were not deemed suitable, we report results descriptively. Similar study data were pooled using the software Review Manager 5.4 (The Cochrane Collaboration, 2020). For dichotomous outcomes, we calculated risk ratio (RR) and 95% confidence intervals (CI); for continuous data, we calculated mean differences (MD), or standardized mean differences (SMD), as appropriate, and 95% CI. We pooled data using random-effects metanalyses; $I^2 \geq 50\%$ was considered an indication of high statistical heterogeneity. We planned to conduct pre-specified subgroup analyses for the primary outcomes (when data were available) according to parity (nullipara vs multipara, and multipara with CS vs multipara without previous CS), type of CS (pre-labour vs intrapartum), previous use of oxytocin in labour, and baseline risk for PPH. We could not conduct these analyses because of the small number of trials included and lack of information. We planned to conduct sensitivity analyses for the main comparisons restricted only to high-quality studies (at low risk of bias for random sequence generation and allocation concealment), and restricted only to studies that assessed blood loss using an objective measure. These analyses were not done because all studies measured blood loss objectively and none were high-quality. Due to the small number of trials, we could not assess publication bias.

We assessed the certainty (quality) of the body of evidence for selected outcomes using the GRADE approach[37]. Evidence was categorized as being of high, moderate, low, or very low quality. We downgraded the quality of the evidence one or two levels because of trial limitations, inconsistency, indirectness, imprecision, and publication bias[37]. This process was conducted by two independent investigators; disagreements were solved by a third senior investigator.

RESULTS

A total of 13,389 unique references were identified. After title and abstract screening, six citations were selected for full text reading. Two studies and one abstract were excluded (Supplementary file 3) and three trials[30-32] were included in the review (Figure 1).

The three trials included a total of 180 women and were conducted between 1998 and 2012, in Japan[30], Canada[31] , and India[32] (Table 1 and Supplementary file 4). None of the trials provided clear information on the proportion of nulliparas or multiparas (with and without previous CS). One trial[32] did not report participants´ baseline risk for PPH, gestational age, or type of CS; the other two trials[30 31] included participants with regular/low baseline risk for PPH, undergoing a pre-labour CS with at least 36 weeks´ gestation, under spinal anaesthesia

For peer review only - http://bmjopen.bmj.com/site/about/guidelines.xhtml

(Table 1). All three trials compared IV vs IMY oxytocin administration after foetal delivery. There was heterogeneity between studies on the oxytocin doses used in each route; the IMY dose ranged from 4.3 IU[30] to 20 IU[31], and the total IV dose ranged from 4.3 IU[30] to 20 IU[32]. One trial[30] administered oxytocin with the placenta still in place, immediately after umbilical cord clamping, while the other two studies waited for placental detachment or removal before giving the oxytocin[31 32]. Akinaga et al randomised 40 Japanese women to receive either an IMY oxytocin injection and an IV saline bolus injection, or an IV oxytocin bolus and an IMY saline injection immediately after umbilical cord clamping; all women also received a slow oxytocin maintenance infusion for the next five hours[30]. Dennehy et al randomised 40 women in Canada to receive an IMY oxytocin bolus injection into the uterine fundus and a saline IV bolus injection, or an IV oxytocin bolus injection and a saline IMY injection after placental removal[31]. Mangla et al randomised 100 women in India to receive either an IMY oxytocin bolus injection (half in each uterine cornus) or to start a 500 mL infusion containing oxytocin after placental separation[32]. The two trials that reported the volume of blood loss [30 32] used objective methods of assessment, but only one[32] assessed the incidence of blood loss > 900 mL. All trials assessed the need for additional uterotonics and at least one adverse effect of oxytocin. No trial reported any of our other secondary outcomes (Table 1 and Supplementary file 5).

For peer review only

Table 1. Main characteristics of trials on routes of oxytocin administration at caesarean section

Characteristics	Akinaga 2016 (ref 30)	Dennehy 1998 (ref 31)	Mangla 2012 (ref 32)
Setting	Japan, one university hospital	Canada, no information on type of hospital(s)	India, one hospital
Design	Double blind, placebo controlled RCT	Double blind, placebo controlled RCT	RCT
Sample size	40	40	100
Gestational age	all ≥ 36 weeks	all ≥ 37 weeks	no information
Baseline risk for PPH	all low risk	all low risk	no information
Parity	unclear	no information	no information
Participants with previous CS	unclear	no information	no information
Type of CS	Pre-labour, scheduled	Pre-labour, scheduled	no information
Previous exposure to oxytocin	No	No	no information
Anaesthesia	spinal	spinal	spinal or general
Timing of administration	After umbilical cord clamping	After placental removal	After placental separation
Oxytocin routes and regimen	IMY bolus vs IV bolus followed by IV maintenance infusion	IMY bolus vs IV bolus	IMY bolus vs IV infusion
Oxytocin dose, diluent, speed of administration, (dose/min), Total dose	IMY bolus: 4.3 IU[1] in 2 mL 0.9% saline over 30 sec in uterine fundus. Total dose: 4.3 IU<br><br>IV bolus: 4.3 IU[1] in 10 ml 0.9% saline over 30 sec (8.5 IU/min) Total dose: 4.3 IU<br><br>Maintenance infusion (both groups): 6.1 IU[2] in 500 ml Ringer at 100 ml/h for 5 h (0.02 IU/min) Total dose: 6.1 IU<br><br>Total oxytocin regimen dose[3]: 10.4 IU	IMY bolus: 2 ml of 10 IU/ml in uterine fundus. Total dose: 20 IU<br><br>IV bolus: 0.5 ml of 10 IU/ml over unknown time Total dose: 5 IU	IMY bolus: 5 IU in 10 ml 0.9% saline (5 ml in each uterine cornu). Total dose: 5 IU<br><br>IV infusion 20 IU in 500 ml Ringer, no information on speed or duration Total dose: 20 IU
Outcomes reported	Volume of blood loss, Additional uterotonics, Adverse effects	Additional uterotonics, Adverse effects	Volume of blood loss, Additional uterotonics, Adverse effects

CS: caesarean section, IU: international units, IV: intravenous, IMY: Intramyometrial, min: minutes, ml: millilitre, PPH: post-partum haemorrhage, RCT: randomised controlled trial, Ref: reference, Sec: seconds.
1. Bolus dose was calculated based on the mean weight of participants (0.07 IU/Kg x 61 Kg), 2. Maintenance infusion, dose was calculated based on the mean weight of participants (0.01 IU/Kg x 61 Kg).
3. Total oxytocin dose received by all participants (bolus + maintenance infusion)

Figure 2 presents the risk of bias of the three included studies (see Supplementary file 6 for details and summary graph). All studies had at least one domain with unclear or high risk of

For peer review only

bias. Akinaga 2016[30] and Mangla 2012[32] did not describe the method used to generate the randomization sequence; Dennehy 1998[31] and Mangla 2012[32] did not describe how allocation concealment was achieved. Overall, the three trials had an unclear risk of bias for random sequence generation or allocation concealment, or both domains. We contacted the authors of the three trials to obtain additional details, but none replied.

Table 2 presents a summary of all outcomes for the comparison between prophylactic IMY versus IV oxytocin in women having a CS. See supplementary file 7 for the assessment of the certainty of the evidence for each outcome.

Table 2. Comparison of prophylactic intramyometrial versus intravenous oxytocin to reduce bleeding in women giving birth by caesarean

**Outcome or subgroup title**	**N studies**	**N women**	**Statistical method**	**Effect size**
1. PPH > 1000 mL	1	100	Risk Ratio (IMY, Random, 95% CI)	0.14 (0.01, 2.70)
2. Use of additional uterotonics	3	179	Risk Ratio (IMY, Random, 95% CI)	0.71 (0.05, 9.73)
3. Hypotension not requiring ephedrine	1	40	Risk Ratio (IMY, Random, 95% CI)	1.00 (0.29, 3.45)
4. Hypotension requiring ephedrine	2	79	Risk Ratio (IMY, Random, 95% CI)	Not estimable
5. Nausea and/or vomiting	2	140	Risk Ratio (IMY, Random, 95% CI)	0.14 (0.03, 0.78)
6. Headache	1	40	Risk Ratio (IMY, Random, 95% CI)	3.00 (0.13, 69.52)
7. Facial flushing	1	40	Risk Ratio (IMY, Random, 95% CI)	0.50 (0.05, 5.08)
8. Mean blood loss (mL)	1	40	Mean Difference (IMY, Random, 95% CI)	-57.40 (-13.09, -101.71)

CI: Confidence interval, IMY: Intramyometrial, PPH: Post-partum haemorrhage,

Incidence of PPH

None of the studies assessed PPH according to the standard definition ($\geq$ 1000 ml). However, Mangla 2012[32] reported that none of the women who received IMY oxytocin had a blood loss > 900 ml compared to 6% of those who received IV oxytocin (0/50 versus 3/50, RR 0.14, 95% CI 0.01 to 2.70; 100 participants; 1 RCT, low certainty evidence). (Table 2, Supplementary file 8).

Need for additional uterotonics

The pooled estimate from the three trials was very imprecise and compatible with an important risk reduction or increase in the need for additional uterotonic with IMY compared to IV oxytocin injection (4/90 versus 5/89, respectively; RR 0.71; 95% CI 0.05 to 9.73; 179 participants; 3 RCTs; I^2=61%, very low certainty evidence) (Figure 3, Table 2)

Adverse effects of oxytocin

Hypotension: Data from one trial[30] indicates that IMY compared to IV oxytocin administration may have no effect on the incidence of isolated hypotension episodes that do not require ephedrine (RR: 1.00, 95% CI 0.29 to 3.45; 40 participants; 1 RCT; low certainty

evidence). Two trials[30 31] (79 participants) assessed the incidence of persistent hypotension requiring treatment with epinephrine, but there were no events among the women who received IMY or IV oxytocin (Table 2, Supplementary file 8).

Changes in heart rate: We could not perform a metanalysis for this outcome. Akinaga[30] reported no apparent changes in the heart rate (HR) of the 20 women who received IMY oxytocin while the HR of the IV group started to increase soon after the bolus injection, reaching a maximum value 75 seconds after. The mean maximum HR in the IV group was significantly higher in the IMY group (92.9 $\pm$ 10.3 vs 83.2 $\pm$ 7.2 beats per minute, p=0.003). On the other hand, Dennehy reported a significantly higher increase in HR one minute after IMY than after IV oxytocin administration, but the authors did not provide numerical data for this outcome[31].It should be noted that while Akinaga[30] gave the same dose of oxytocin (4.3 IU) in both routes, the IMY oxytocin dose given by Dennehy[31] was four times higher than the IV dose (20 IU vs 5 IU, respectively).

Nausea and/or vomiting: The pooled estimate of two trials including 140 women showed a lower average risk of nausea and/or vomiting in the group that received IMY oxytocin (RR 0.14; 95% CI 0.03 to 0.78; 140 participants; 2 RCTs; I^2=0%, low certainty evidence) (Figure 4, Table 2).

Headache and facial flushing: Only Akinaga[30] reported the incidence of headache and facial flushing. One woman in the IMY group complained of headache versus none in the IV group (1/20 versus 0/20, RR 3.00, 95% CI 0.13 to 69.52; 40 participants; 1 RCT; low certainty evidence). One participant in the IMY and two in the IV oxytocin group had facial flushing (1/20 versus 2/20, RR 0.50, 95% CI 0.05 to 5.08; 40 participants; 1 RCT; low certainty evidence). (Table 2, Supplementary file 8).

Volume of blood loss

Mangla[32] reported that the women in the IMY group had a lower mean volume of blood loss than those in the IV group (460 mL versus 606 mL, respectively), but did not provide the standard deviations (SD) for this measure. Akinaga[30] also reported a slightly lower mean volume of blood loss in the IMY than in the IV group (606.8 $\pm$ 68.8 versus 664.2 $\pm$ 74.1 mL; MD -57.40 mL; 95% CI -13.09 to -101.71; 40 participants; 1 RCT; low certainty evidence) (Table 2, Supplementary file 8). We could not pool the results of the two trials because one of them[32] did not provide information on SD and authors did not reply to our email.

DISCUSSION

Our extensive search identified only three trials on the effects of different routes of administration of prophylactic oxytocin in women giving birth by CS. The existing evidence suggests that IMY compared to IV oxytocin administration may result in little to no difference on the incidence of PPH ($\geq$ 1000 mL) and most side effects (hypotension, headache, or facial flushing), but IMY oxytocin may reduce the incidence of nausea/vomiting. The evidence also suggests that IMY oxytocin may reduce the volume of blood loss slightly. We are very uncertain about the effects of IMY versus IV oxytocin on the need for additional uterotonics.

The overall certainty of the evidence was low or very low for all outcomes due mainly to imprecision.

Where information was available, most trials recruited only healthy women with a low baseline risk for PPH, who were having an elective pre-labour CS under spinal anaesthesia to deliver a single foetus at term. Information about parity, including the percentage of participants with one or more previous CS, was unclear or missing in all studies. Therefore, we cannot infer that the results of this systematic review apply to women with other characteristics, such as preterm or multiple pregnancies, women having intra-partum CS, or those with previous exposure to oxytocin for labour induction or augmentation. The different doses and regimen of oxytocin used in the trials can also have affected the results because oxytocin plasma levels are dose dependent.[38 39]

It is surprising that we found no studies comparing IM versus IV prophylactic oxytocin at CS, since these are the more frequently used routes of administration, and there are many trials on this comparison for women giving birth vaginally[7]. While the slower onset of action of oxytocin after IM administration[18 19] can raise concerns about its capacity to control bleeding at CS, this could, in theory, be circumvented by injecting it earlier, for example at uterine incision instead of after foetal delivery. Potential advantages of IM compared to IV administration include fewer side effects due to the slower increase in oxytocin plasma concentration, and a longer lasting effect[18 19] which could preclude the need for IV oxytocin maintenance infusions. Intravenous oxytocin is a high-alert medication[40] (a drug with increased risk of causing significant patient harm when used in error) which was listed in third place in the list of the top medication errors in 2020[41]. Therefore, in theory, an additional safety advantage of the IM route is that it could potentially reduce medication errors associated with IV oxytocin. Finally, for women having a CS, prophylactic IM oxytocin injection would be painless because the patient would be under anaesthesia. It was also surprising to find three trials that assessed IMY oxytocin administration[30-32] because the drug is not licenced for IMY use, and there are no pharmacokinetic studies on this route of oxytocin administration.

Previous reviews have compared various uterotonics (including oxytocin) versus placebo or other agents in different doses, regimens and timing of administration to prevent PPH at CS [14 42]. However, to the best of our knowledge, this is the first systematic review to assess different routes of oxytocin administration to prevent PPH in women giving birth by CS. Strong points of our review include its comprehensive literature search without language restrictions and strict adherence to standard Cochrane methods[33]. Limitations of the review include the small number of trials identified, the lack of response from authors to clarify important methodological and clinical aspects of their studies, and the fact that none of the trials addressed additional core clinical outcomes.

At present, there is insufficient evidence to recommend IMY or IV oxytocin administration at CS to prevent PPH. Our review findings indicate the need for more high quality trials comparing the effectiveness and safety of different routes of prophylactic oxytocin administration, including the intramuscular route, in women having a CS. Future trials should

For peer review only

have adequate sample size, be of high methodological and reporting quality, and recruit participants who represent the general obstetric population, including women at high risk for PPH due to clinical or obstetric disorders, and women undergoing prelabour as well as intrapartum CS, with or without previous exposure to oxytocin for labour induction or augmentation. These trials also need to report all relevant PPH prevention core outcomes [35]. Given the increasing numbers of births by CS worldwide including in LMIC, and the high rates of mortality following CS in these countries[43], research to identify the most beneficial route and regimen for prophylactic oxytocin to prevent PPH in women giving birth by CS should be prioritized to avoid preventable mortality and morbidity. Particularly disturbing is the lack of evidence for women having intrapartum CS. These women are particularly vulnerable to uterine atony and increased post-partum bleeding because of prolonged labours, often with previous exposure to oxytocin for induction or augmentation which may desensitize oxytocin receptors and impair myometrial response to prophylactic oxytocin after delivery[44 45]. These evidence gaps are likely to affect more dramatically women in LMIC where skills and resources to provide safe and timely CS, and to deal with its complications, are sub-optimal. This lack of evidence is thus likely to increase maternal and perinatal inequalities between and within countries.

CONCLUSIONS

There is limited, low to very low certainty evidence on the effects of two different routes of prophylactic oxytocin administration at CS. The limited evidence suggests that IMY compared to IV oxytocin may result in little to no difference in the incidence of PPH and most side effects, but may reduce nausea/vomiting and the volume of blood loss slightly. More trials are needed on this relevant question, especially on the effects of intramuscular oxytocin and involving women with intrapartum CS.

Author Contributions

MRT, MS, APB and MW conceptualized the study. MRT wrote the study protocol with the contributions of MS, APB, MW and RR. The search strategy was developed by COCL, MRT and RR, with the contribution of MS and APB. Citation screening and study selection were performed by MS, APB, ALCM, RLP, and COCL under the supervision of RR and MRT. Data extraction and study quality assessment were performed by MRT, ALCM, RR and RLP. Data analysis was conducted by MRT, RR, ALCM and RLP. The report was drafted by MRT with input from MS, APB and MW. All authors reviewed and approved the final manuscript. MRT is the guarantor and takes responsibility for the content of this article

Acknowledgements: none

Funding This work was supported by UNDP/UNFPA/UNICEF/WHO/World Bank Special Programme of Research, Development and Research Training in Human Reproduction (HRP), Department of Sexual and Reproductive Health and Research, World Health Organization, Geneva, Switzerland.

For peer review only

Disclaimer: The funders had no role in conducting or writing the study

Competing interests: None declared.

Patient consent: Not required

Ethics approval: This systematic review used published studies. Ethical approval was waived.

Provenance and peer review Not commissioned; externally peer reviewed.

Data sharing statement No additional data are available.

Figure legends:

Figure 1. Flowchart of the process of study selection
Figure 2. Risk of bias summary of trials on oxytocin route of delivery at caesarean
Figure 3. Forest plot of intramyometrial versus intravenous oxytocin at caesarean. Outcome: need for additional uterotonic
Figure 4. Forest plot of intramyometrial versus intravenous oxytocin at caesarean. Outcome: nausea and/or vomiting

For peer review only

References

1. Betran AP, Ye J, Moller AB, et al. The Increasing Trend in Caesarean Section Rates: Global, Regional and National Estimates: 1990-2014. *PLoS One* 2016;11(2):e0148343. doi: 10.1371/journal.pone.0148343
2. Fawcus S, Moodley J. Postpartum haemorrhage associated with caesarean section and caesarean hysterectomy. *Best Pract Res Clin Obstet Gynaecol* 2013;27(2):233-49. doi: 10.1016/j.bpobgyn.2012.08.018
3. Parant O, Guerby P, Bayoumeu F. [Obstetric and anesthetic specificities in the management of a postpartum hemorrhage (PPH) associated with cesarean section]. *J Gynecol Obstet Biol Reprod (Paris)* 2014;43(10):1104-22. doi: 10.1016/j.jgyn.2014.10.004
4. Deneux-Tharaux C, Bonnet MP, Tort J. [Epidemiology of post-partum haemorrhage]. *J Gynecol Obstet Biol Reprod (Paris)* 2014;43(10):936-50. doi: 10.1016/j.jgyn.2014.09.023
5. Say L, Chou D, Gemmill A, et al. Global causes of maternal death: a WHO systematic analysis. *Lancet Glob Health* 2014;2(6):e323-33. doi: 10.1016/S2214-109X(14)70227-X
6. World Health Organization. WHO recommendations: uterotonics for the prevention of postpartum haemorrhage. Geneva: World Health Organization; 2018. Licence: CC BY-NC-SA 3.0 IGO.
7. Oladapo OT, Okusanya BO, Abalos E, et al. Intravenous versus intramuscular prophylactic oxytocin for the third stage of labour. *Cochrane Database Syst Rev* 2020;11:CD009332. doi: 10.1002/14651858.CD009332.pub4
8. World Health Organization. WHO recommendation on routes of oxytocin administration for the prevention of postpartum haemorrhage after vaginal birth. Geneva: World Health Organization; 2020. Licence: CC BY-NC-SA 3.0 IGO.
9. Dahlke JD, Mendez-Figueroa H, Maggio L, et al. Prevention and management of postpartum hemorrhage: a comparison of 4 national guidelines. *Am J Obstet Gynecol* 2015;213(1):76 e1-76 e10. doi: 10.1016/j.ajog.2015.02.023
10. Sentilhes L, Goffinet F, Vayssiere C, et al. Comparison of postpartum haemorrhage guidelines: discrepancies underline our lack of knowledge. *BJOG* 2017;124(5):718-22. doi: 10.1111/1471-0528.14305
11. Weale N, Laxton C. Prophylactic use of oxytocin at caesarean section: where are the guidelines? *Anaesthesia* 2013;68(10):1006-9. doi: 10.1111/anae.12337
12. Carvalho JC, Balki M, Kingdom J, et al. Oxytocin requirements at elective cesarean delivery: a dose-finding study. *Obstet Gynecol* 2004;104(5 Pt 1):1005-10. doi: 10.1097/01.AOG.0000142709.04450.bd
13. Dyer RA, van Dyk D, Dresner A. The use of uterotonic drugs during caesarean section. *Int J Obstet Anesth* 2010;19(3):313-9. doi: 10.1016/j.ijoa.2010.04.011
14. Stephens LC, Bruessel T. Systematic review of oxytocin dosing at caesarean section. *Anaesth Intensive Care* 2012;40(2):247-52. doi: 10.1177/0310057X1204000206
15. Roach MK, Abramovici A, Tita AT. Dose and duration of oxytocin to prevent postpartum hemorrhage: a review. *Am J Perinatol* 2013;30(7):523-8. doi: 10.1055/s-0032-1329184
16. Yamaguchi ET, Siaulys MM, Torres ML. Oxytocin in cesarean-sections. What's new? *Braz J Anesthesiol* 2016;66(4):402-7. doi: 10.1016/j.bjane.2014.11.015
17. Balki M, Tsen L. Oxytocin protocols for cesarean delivery. *Int Anesthesiol Clin* 2014;52(2):48-66. doi: 10.1097/AIA.0000000000000016
18. FDA Approved Drug Products: Pitocin (oxytocin) injection. Available at: https://www.accessdata.fda.gov/drugsatfda_docs/label/2014/018261s031lbl.pdf. Accessed on December 20, 2020.

19. Prescribers Digital Reference (PDR): Oxytocin - Drug Summary. Available at: https://www.pdr.net/drug-summary/Pitocin-oxytocin-1666. Accessed on December 20, 2020.
20. Pinder AJ, Dresner M, Calow C, et al. Haemodynamic changes caused by oxytocin during caesarean section under spinal anaesthesia. *Int J Obstet Anesth* 2002;11(3):156-9. doi: 10.1054/ijoa.2002.0970
21. Thomas JS, Koh SH, Cooper GM. Haemodynamic effects of oxytocin given as i.v. bolus or infusion on women undergoing Caesarean section. *Br J Anaesth* 2007;98(1):116-9. doi: 10.1093/bja/ael302
22. Tsen LC, Balki M. Oxytocin protocols during cesarean delivery: time to acknowledge the risk/benefit ratio? *Int J Obstet Anesth* 2010;19(3):243-5. doi: 10.1016/j.ijoa.2010.05.001
23. Jonsson M, Hanson U, Lidell C, et al. ST depression at caesarean section and the relation to oxytocin dose. A randomised controlled trial. *BJOG* 2010;117(1):76-83. doi: 10.1111/j.1471-0528.2009.02356.x
24. Svanstrom MC, Biber B, Hanes M, et al. Signs of myocardial ischaemia after injection of oxytocin: a randomized double-blind comparison of oxytocin and methylergometrine during Caesarean section. *Br J Anaesth* 2008;100(5):683-9. doi: 10.1093/bja/aen071
25. Thomas TA, Cooper GM, Editorial Board of the Confidential Enquiries into Maternal Deaths in the United K. Maternal deaths from anaesthesia. An extract from Why mothers die 1997-1999, the Confidential Enquiries into Maternal Deaths in the United Kingdom. *Br J Anaesth* 2002;89(3):499-508.
26. Sarna MC, Soni AK, Gomez M, et al. Intravenous oxytocin in patients undergoing elective cesarean section. *Anesth Analg* 1997;84(4):753-6. doi: 10.1097/00000539-199704000-00010
27. Unterscheider J, Breathnach F, Geary M. Standard medical therapy. In: Arulkumaran S, Karoshi M, Keith LG, et al., eds. A comprehensive textbook of postpartum hemorrhage An essential clinical reference for effective management. 2nd ed. London: Sapiens Publishing Ltd 2012:355-60.
28. Catanzarite VA. Prophylactic intramyometrial carboprost tromethamine does not substantially reduce blood loss relative to intramyometrial oxytocin at routine cesarean section. *Am J Perinatol* 1990;7(1):39-42. doi: 10.1055/s-2007-999443
29. Hayashi RH, Castillo MS, Noah ML. Management of severe postpartum hemorrhage due to uterine atony using an analogue of prostaglandin F2 alpha. *Obstet Gynecol* 1981;58(4):426-9.
30. Akinaga C, Uchizaki S, Kurita T, et al. Randomized double-blind comparison of the effects of intramyometrial and intravenous oxytocin during elective cesarean section. *J Obstet Gynaecol Res* 2016;42(4):404-9. doi: 10.1111/jog.12926
31. Dennehy KC, Rosaeg OP, Cicutti NJ, et al. Oxytocin injection after caesarean delivery: intravenous or intramyometrial? *Can J Anaesth* 1998;45(7):635-9. doi: 10.1007/BF03012092
32. Mangla D, Goel JK, Goel R. Prophylactic Intramyometrial Oxytocin before Placenta Delivery during Cesarean Section Prevents Postpartum Hemorrhage: A Prospective Randomized Study of 150 Women. *J South Asian Feder Obst Gynae* 2012;4(2):93-96.
33. Higgins JPT, Thomas J, Chandler J, et al. Cochrane Handbook for Systematic Reviews of Interventions version 6.1 [Internet]. 2020. Available at www.training.cochrane.org/handbook. In: Cochrane, ed.
34. Moher D, Liberati A, Tetzlaff J, et al. Preferred reporting items for systematic reviews and meta-analyses: the PRISMA statement. *J Clin Epidemiol* 2009;62(10):1006-12. doi: 10.1016/j.jclinepi.2009.06.005
35. Meher S, Cuthbert A, Kirkham JJ, et al. Core outcome sets for prevention and treatment of postpartum haemorrhage: an international Delphi consensus study. *BJOG* 2019;126(1):83-93. doi: 10.1111/1471-0528.15335
36. Ouzzani M, Hammady H, Fedorowicz Z, et al. Rayyan-a web and mobile app for systematic reviews. *Syst Rev* 2016;5(1):210. doi: 10.1186/s13643-016-0384-4
37. Guyatt GH, Oxman AD, Vist GE, et al. GRADE: an emerging consensus on rating quality of evidence and strength of recommendations. *BMJ* 2008;336(7650):924-6. doi: 10.1136/bmj.39489.470347.AD

For peer review only

38. Uvnas-Moberg K, Ekstrom-Bergstrom A, Berg M, et al. Maternal plasma levels of oxytocin during physiological childbirth - a systematic review with implications for uterine contractions and central actions of oxytocin. *BMC Pregnancy Childbirth* 2019;19(1):285. doi: 10.1186/s12884-019-2365-9

39. Yamaguchi ET, Cardoso MM, Torres ML, et al. Serum oxytocin concentrations in elective caesarean delivery: a randomized comparison of three infusion regimens. *Int J Obstet Anesth* 2011;20(3):224-8. doi: 10.1016/j.ijoa.2011.03.004

40. Institute for Safe Medication Practices-ISMP 2018. High-Alert Medications in Acute Care Settings. Available at: https://www.ismp.org/recommendations/high-alert-medications-acute-list

41. Institute for Safe Medication Practices-ISMP 2021. Start the Year Off Right by Preventing These Top 10 Medication Errors and Hazards from 2020. Available at: https://www.ismp.org/resources/start-year-right-preventing-these-top-10-medication-errors-and-hazards-2020.

42. Gallos I, Williams H, Price M, et al. Uterotonic drugs to prevent postpartum haemorrhage: a network meta-analysis. *Health Technol Assess* 2019;23(9):1-356. doi: 10.3310/hta23090

43. Sobhy S, Arroyo-Manzano D, Murugesu N, et al. Maternal and perinatal mortality and complications associated with caesarean section in low-income and middle-income countries: a systematic review and meta-analysis. *Lancet* 2019;393(10184):1973-82. doi: 10.1016/S0140-6736(18)32386-9

44. Bischoff K, Nothacker M, Lehane C, et al. Lack of controlled studies investigating the risk of postpartum haemorrhage in cesarean delivery after prior use of oxytocin: a scoping review. *BMC Pregnancy Childbirth* 2017;17(1):399. doi: 10.1186/s12884-017-1584-1

45. Phaneuf S, Rodriguez Linares B, TambyRaja RL, et al. Loss of myometrial oxytocin receptors during oxytocin-induced and oxytocin-augmented labour. *J Reprod Fertil* 2000;120(1):91-7. doi: 10.1530/jrf.0.1200091

For peer review only

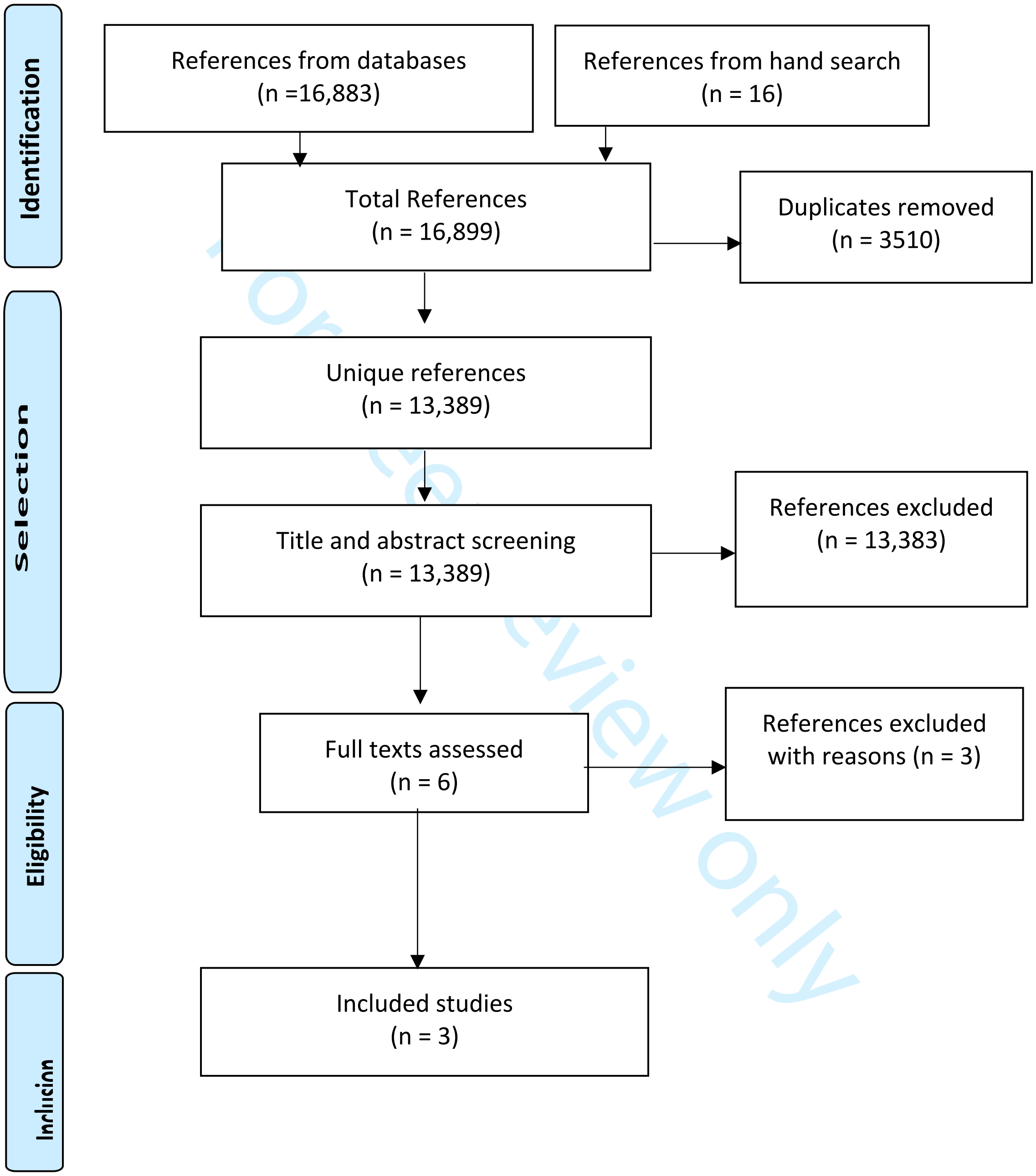

For peer review only - http://bmjopen.bmj.com/site/about/guidelines.xhtml

Figure 2. Risk of bias summary of trials on oxytocin route of delivery at caesarean section

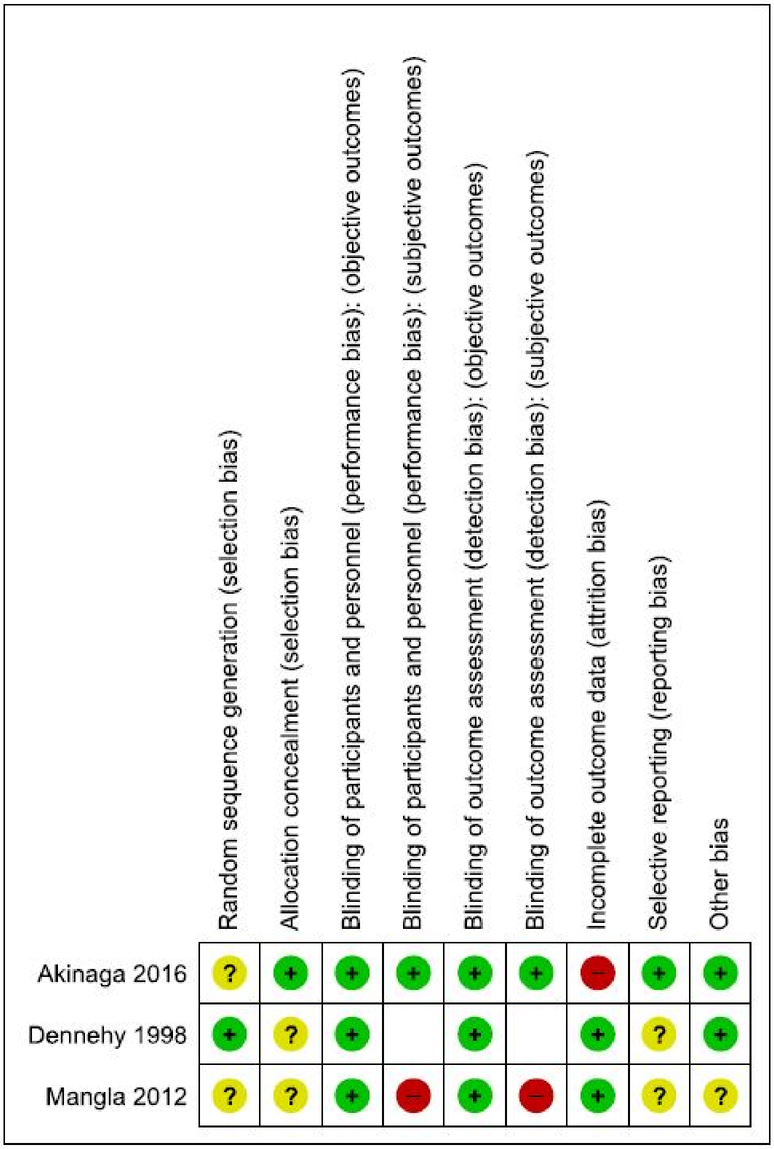

Green: Low risk of bias, Yellow: uncertain risk of bias, Red: high risk of bias
Empty: Does not apply (study did not have subjective outcomes)

For peer review only - http://bmjopen.bmj.com/site/about/guidelines.xhtml

Figure 3. Forest plot of intramyometrial versus intravenous oxytocin at caesarean section. Outcome: need for additional uterotonic.

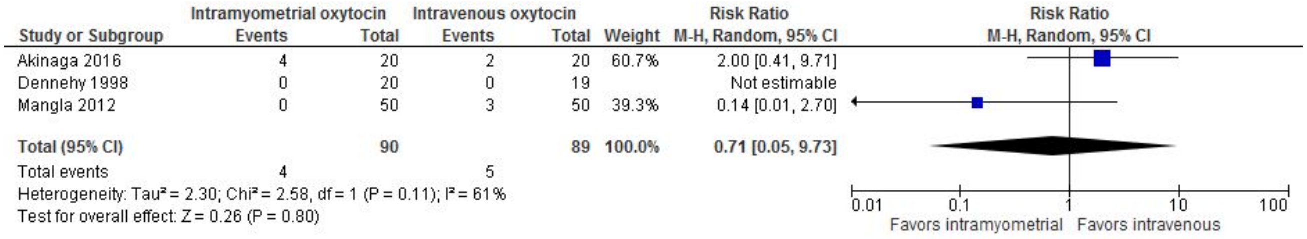

For peer review only

Figure 4. Forest plot of intramyometrial versus intravenous oxytocin at caesarean section. Outcome: nausea and/or vomiting

	Intramyometrial oxytocin		Intravenous oxytocin			Risk Ratio
Study or Subgroup	Events	Total	Events	Total	Weight	M-H, Random, 95% CI
Akinaga 2016	0	20	1	20	29.2%	0.33 [0.01, 7.72]
Mangla 2012	1	50	10	50	70.8%	0.10 [0.01, 0.75]
**Total (95% CI)**		**70**		**70**	**100.0%**	**0.14 [0.03, 0.78]**
Total events	1		11			

Heterogeneity: Tau² = 0.00; Chi² = 0.41, df = 1 (P = 0.52); I² = 0%
Test for overall effect: Z = 2.25 (P = 0.02)

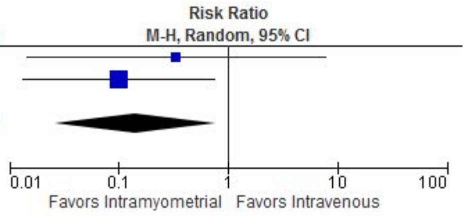

Akinaga 2016: nausea identified by questioning women immediately after oxytocin injection, every 2 minutes for 20 minutes.
Mangla 2012: use of additional antiemetic due to nausea and vomiting after initial antiemetic dose given to all women by anesthetist before induction.

For peer review only

NIHR | National Institute for Health Research

PROSPERO
International prospective register of systematic reviews

Prophylactic oxytocin to prevent postpartum hemorrhage at cesarean section

Maria Regina Torloni, Monica Siaulys, Rachel Riera, Ana Pilar Betran, Mariana Widmer

To enable PROSPERO to focus on COVID-19 registrations during the 2020 pandemic, this registration record was automatically published exactly as submitted. The PROSPERO team has not checked eligibility.

Citation

Maria Regina Torloni, Monica Siaulys, Rachel Riera, Ana Pilar Betran, Mariana Widmer. Prophylactic oxytocin to prevent postpartum hemorrhage at cesarean section. PROSPERO 2020 CRD42020186797 Available from:
https://www.crd.york.ac.uk/prospero/display_record.php?ID=CRD42020186797

Review question

In women submitted to cesarean section, what is the effectiveness and safety of prophylactic oxytocin administered in different periods, regimens, routes, and doses?

Searches

We will develop a search strategy combining key words related to oxytocin and cesarean delivery that will be adapted and run in several databases such as PubMed, Embase, CENTRAL and CINAHL. There will be no date or language limitations.

We will also create and run a search for ongoing studies in trial registry platforms.

The electronic search will be complemented by screening the reference lists of all included studies and other systematic reviews on prevention of postpartum hemorrhage (PPH).

Types of study to be included

All randomized controlled trials of effectiveness or side-effects of oxytocin versus oxytocin to prevent PPH in women submitted to CS will be included.

Condition or domain being studied

Prevention of postpartum hemorrhage. Cesarean section. Oxytocin

Participants/population

Inclusion criteria:

We will include studies that enrolled women of any age, race, socioeconomic level, nationality, with or without any health problems, at high or low risk for PPH, who delivered by cesarean section (CS) for any indication, with any number of fetuses, and at any gestational age after 26 weeks. We will include trials that enrolled women who had a primary or a repeat CS, a prelabour (planned or emergency) CS, or an intrapartum CS. We will include studies where participants received oxytocin for labor induction or augmentation before the CS. Studies that tested oxytocin to prevent PPH in participants having vaginal and cesarean deliveries will be included if the authors present data separately for the women having a CS.

Exclusion criteria:

We will exclude studies that assessed oxytocin in combination with another uterotonic drug, or that compared oxytocin versus placebo or other uterotonic drugs to prevent PPH.

Intervention(s), exposure(s)

We will compare different routes, dosages, regimen, or periods of administration of oxytocin to prevent PPH in women submitted to CS. We will include trials in which non-pharmacologic co-interventions (e.g. uterine massage) were included as a randomized intervention in all arms of the trial.

For peer review only - http://bmjopen.bmj.com/site/about/guidelines.xhtml

Multi-arm trials that compared oxytocin versus other uterotonic drugs, but that also compared different routes, dosages, regimens or periods of administration of oxytocin alone will be included.

Comparator(s)/control

Any route, dosage, regimens or periods of administration of oxytocin to prevent PPH at CS that are different from the intervention group.

Main outcome(s)

1. Postpartum hemorrhage of at least 1000 mL assessed by any method
2. Need for additional uterotonics

3. Any side effects

* Measures of effect

Incidence, Relative risk. We will use other effect measures depending on the data reported in the trials.

Additional outcome(s)

1. Volume of blood loss (mL) measured by any method

2. Blood transfusion after placental delivery

3. Shock, as defined by trialists

4. Maternal mortality or severe maternal morbidity related to PPH (organ failure, coma, hysterectomy, or as defined by the study authors)

5. Maternal transfer to a higher level of care

6. Maternal satisfaction (or sense of wellbeing) as defined by trialists

7. Breastfeeding at discharge

* Measures of effect

Incidence, Relative risk. We will use other effect measures depending on the data reported in the trials.

Data extraction (selection and coding)

The references retrieved from each database will be uploaded into a reference management software and duplicates will be excluded. Two review authors will independently screen titles and abstracts to select potentially relevant records. Studies selected at first screening will be read by two independent authors who will include those that fulfill the selection criteria. Reasons for exclusion will be listed. Disagreements in these two phases will be solved by a third review author.

Two authors will independently conduct data extraction. Disagreements will be solved by a third review author. We will contact study authors by email to obtain missing information and study details, when necessary.

We will use a form created for this review to extract data from each included study. The data will be entered in the Review Manager software and checked for accuracy. The following information will be collected from each study: country, year, trial registration, design details, number of participants, inclusion and exclusion criteria, main participant characteristics, type of CS, anesthesia, previous exposure to oxytocin, interventions compared and outcomes reported.

Risk of bias (quality) assessment

The risk of bias of each included study will be assessed independently by two review authors. Disagreements will be solved by a third reviewer. Full justifications for the assessments will be included in the report.

For peer review only - http://bmjopen.bmj.com/site/about/guidelines.xhtml

We will use the Cochrane Risk of Bias (RoB) tool to assess the quality of included trials. The following domains will be assessed and graded as being at high, low, or unclear risk of bias: 1) Random sequence generation, 2) Allocation concealment, 3) Blinding of participants and personnel, 4) Blinding of outcome assessment, 5) Incomplete outcome data, 6) Selective reporting (checking for reporting bias), 7) Other bias.

Strategy for data synthesis

We will pool data of similar trials in metanalyses using the random effect model using the Review Manager software (version 5.3). For dichotomous outcomes, we will present results as summary risk ratio (RR) with 95% confidence intervals (CIs). For continuous outcomes, we will pool the results of studies that measured the outcome in the same way and calculate mean differences (MD). In metanalyses of studies that measured the same continuous outcomes using different methods, we will use the standardized mean difference (SMD).

When metanalysis is not possible, we will present the results descriptively.

We will conduct separate comparisons for prelabor and intrapartum CS.

We plan to conduct the following comparisons, where data is available:

• different moments of administration.

• different routes of administration

• different regimens

• different doses

Analysis of subgroups or subsets

We plan to conduct the following subgroup analyses for the primary outcomes, if data are available: according to pregnancy characteristics (parity, onset of labor), according to previous use of oxytocin for induction/augmentation, and according to baseline risk for PPH. We will assess subgroup differences by interaction tests available in RevMan 5 (RevMan 2014).

We plan to conduct the following sensitivity analyses for the main outcomes, if data are available: restricted only to high-quality studies, and restricted only to studies that assessed blood loss objectively.

Contact details for further information

Maria Regina Torloni
torlonimr@gmail.com

Organisational affiliation of the review

Hospital e Maternidade Santa Joana

Review team members and their organisational affiliations

Dr Maria Regina Torloni. Hospital e Maternidade Santa Joana
Dr Monica Siaulys. Hospital e Maternidade Santa Joana
Dr Rachel Riera. Universidade Federal de Sao Paulo
Dr Ana Pilar Betran. World Health Organization
Dr Mariana Widmer. World Health Organization

Collaborators

Dr Ligia Mathias. Hospital e Maternidade Santa Joana
Dr Guilherme Haelvoet. Hospital e Maternidade Santa Joana
Dr Ana Luiza Martimbianco. Universidade Federal de Sao Paulo
Carolina Cruz Latorraca. Universidade Federal de Sao Paulo
Dr Rafael Leite Pacheco. Universidade Federal de Sao Paulo

For peer review only

Type and method of review
Intervention, Meta-analysis, Systematic review

Anticipated or actual start date
06 April 2020

Anticipated completion date
31 July 2020

Funding sources/sponsors
Department of Sexual and Reproductive Health and Research, World Health Organization

Conflicts of interest

Language
English

Country
Brazil, Switzerland

Stage of review
Review Ongoing

Subject index terms status
Subject indexing assigned by CRD

Subject index terms
MeSH headings have not been applied to this record

Date of registration in PROSPERO
05 July 2020

Date of first submission
17 May 2020

Stage of review at time of this submission

**Stage**	**Started**	**Completed**
Preliminary searches	Yes	No
Piloting of the study selection process	Yes	No
Formal screening of search results against eligibility criteria	No	No
Data extraction	No	No
Risk of bias (quality) assessment	No	No
Data analysis	No	No

The record owner confirms that the information they have supplied for this submission is accurate and complete and they understand that deliberate provision of inaccurate information or omission of data may be construed as scientific misconduct.

The record owner confirms that they will update the status of the review when it is completed and will add publication details in due course.

For peer review only

Versions

05 July 2020

PROSPERO
This information has been provided by the named contact for this review. CRD has accepted this information in good faith and registered the review in PROSPERO. The registrant confirms that the information supplied for this submission is accurate and complete. CRD bears no responsibility or liability for the content of this registration record, any associated files or external websites.

For peer review only

Supplementary file 2. Search strategy for systematic review on oxytocin route of delivery

Database	Search strategy	Results
BVS	#1 MH:Cesárea OR MH:"Cesarean Section" OR Cesárea OR (Cesarean Section) OR (Parto Abdominal) OR (Abdominal Deliveries) OR (Abdominal Delivery) OR (C Section (OB)) OR (C-Section (OB)) OR (C-Sections (OB)) OR (Caesarean Section) OR (Caesarean Sections) OR (Cesarean Sections) OR (Postcesarean Section) OR MH:E04.520.252.500$ OR MH:"Cesárea Repetida" OR MH:"Cesarean Section, Repeat" OR MH:Recesariana OR (Cesárea Repetida) OR Recesariana OR (Repeat Cesarean Section) OR (Repeat Cesarean Sections) OR (Operación Cesárea Repetida) OR MH:E04.520.252.500.150$ OR MH:"Parto Obstétrico" OR MH:"Delivery, Obstetric" OR (Parto Obstétrico) OR (Liberação Obstétrica) OR (Obstetric Deliveries) OR (Obstetric Delivery) OR (Liberación Obstétrica) OR MH:E04.520.252$ OR MH:"Terceira Fase do Trabalho de Parto" OR MH:"Labor Stage, Third" OR MH:"Tercer Periodo del Trabajo de Parto" OR (Terceira Fase do Trabalho de Parto) OR (Tercer Periodo del Trabajo de Parto) OR (Terceiro Estágio do Trabalho de Parto) OR (Terceiro Período do Trabalho de Parto) OR (Third Labor Stage) OR (Third Labor Stages) OR (Third Stage Labor) OR MH:G08.686.784.769.326.500.110$<br><br>#2 MH:Oxitocina OR MH:Oxytocin OR MH:Ocitocina OR Oxitocina OR Oxytocin OR Ocitocina OR Ocytocin OR Pitocin OR Syntocinon OR MH:D06.472.699.631.692.433$ OR MH:D12.644.548.691.692.433$ OR MH:"Receptores de Ocitocina" OR MH:"Receptors, Oxytocin" OR MH:"Receptores de Oxitocina" OR (Receptores de Ocitocina) OR (Receptores de Oxitocina) OR (Oxytocin Receptor) OR (Oxytocin Receptors) OR MH:D12.776.543.750.695.630$ OR MH:D12.776.543.750.720.600.630$ OR MH:D12.776.543.750.750.555.630$ OR MH:D12.776.543.750.750.660.600$ OR MH:Ocitócicos OR MH:Oxytocics OR MH:Oxitócicos OR Ocitócicos OR Oxytocics OR Oxitócicos OR (Efeito Ocitócico) OR (Estimulantes Uterinos) OR (Oxytocic Agents) OR (Oxytocic Drugs) OR (Oxytocic Effect) OR (Oxytocic Effects) OR (Uterine Stimulants) OR (Efecto Oxitócico) OR MH:D27.505.696.875.737$ OR MH:D27.505.954.705.737$<br><br>#3 #1 AND #2	376
CINAHL (via EBSCO)	S1 MH Cesarean Section OR ((Cesarean Sections) OR (Cesarean Section) OR (Abdominal Deliveries) OR (Abdominal Deliver) OR (Abdominal Delivery) OR (C-Section (OB)) OR (C Section (OB)) OR (C-Sections (OB)) OR (Postcesarean Section) OR (cesarean delivery) OR (Cesarean Section) OR (Elective cesarean section) OR (Elective cesarean delivery) OR Cesarean OR (Emergency cesarean section) OR (Emergency cesarean delivery) OR (Intrapartum cesarean section) OR (Intrapartum cesarean delivery) OR (Intra-partum cesarean section) OR (Intra-partum cesarean delivery) OR (Intra partum cesarean section) OR (Intra partum cesarean delivery) OR (Caesarean Section) OR (Caesarean Sections) OR (Postcaesarean Section) OR (caesarean delivery) OR (Elective caesarean section) OR (Elective caesarean delivery) OR Caesarean OR (Emergency caesarean section) OR (Emergency caesarean delivery) OR (Intrapartum caesarean section) OR (Intrapartum caesarean delivery) OR (Intra-partum caesarean section) OR (Intra-partum caesarean delivery) OR (Intra partum caesarean section) OR (Intra partum caesarean delivery) OR (Prelabor cesarean section) OR (Prelabor cesarean delivery) OR (Pre-labor cesarean delivery) OR (Pre-labor cesarean delivery) OR (Pre labor cesarean section) OR (Pre labor cesarean delivery) OR (Prelabour cesarean section) OR (Prelabour cesarean delivery) OR (Pre-labour cesarean delivery) OR (Pre-labour cesarean delivery) OR (Pre labour cesarean section) OR (Pre labour cesarean delivery) OR (Prelabor caesarean section) OR (Prelabor caesarean delivery) OR (Pre-labor caesarean delivery) OR (Pre-labor caesarean delivery) OR (Pre labor caesarean section) OR (Pre labor caesarean delivery) OR (Prelabour caesarean section) OR (Prelabour caesarean delivery) OR (Pre-labour caesarean delivery) OR (Pre-labour caesarean delivery) OR (Pre labour caesarean section) OR (Pre labour caesarean delivery) OR (Repeat Cesarean Section) OR (Repeat Cesarean Sections) OR (Repeat Caesarean Section) OR (Repeat Caesarean Sections) OR (Obstetric anesthesia) OR (Obstetric Deliveries) OR (Obstetric Delivery) OR (Third Labor Stage) OR (Third Labor Stages) OR (Third Stage Labor) OR (Third Stage of Labor) OR (Third-stage labor) OR (Third Labour Stage) OR (Third Labour Stages) OR (Third Stage Labour) OR (Third Stage of Labour) OR (Third-stage labour)) OR MH Cesarean Section, Repeat OR MH Delivery, Obstetric OR MH Labor Stage, Third<br><br>S2 MH Oxytocin OR MH Receptors, Oxytocin OR MH Oxytocics OR (Oxytocin OR Ocytocin OR Syntocinon OR Pitocin OR (Oxytocin Receptor) OR (Oxytocin Receptors) OR (alpha hypophamine) OR (atonin o) OR (beta methyl beta mercaptobutyric acid oxytocin) OR (di sipidin) OR disipidin OR endopituitrin OR fetusin OR hypophysin OR hypophysine OR (intravenous oxytocin) OR mipareton OR neoxyn OR ocytocin OR orasthin OR orastina OR oxitocin OR (oxiton inj) OR oxitone OR oxystin OR oxytan OR (oxytocin 10 usp units in dextrose 5%) OR (oxytocin 20 usp units in dextrose 5%) OR (oxytocin 5 usp units in dextrose 5%) OR (oxytocin dimer) OR (oxytocin pentapeptide) OR (oxytocin s inj) OR oxytocine OR pareton OR partacon OR partocon OR (partocon inj) OR partolact OR partoxin OR physormon OR pitocin OR (pitocin inj) OR piton OR (piton s) OR (piton s inj) OR pituilobine OR pitupartin OR solvoxine OR synpitan OR (synthetic oxytocin inj) OR syntocinon OR (syntocinon inj) OR (syntocinon spray) OR tranoxy OR utedrin OR uteracon OR uterason OR (utron inj) OR xitocin OR Oxytocics OR (Oxytocic Drugs) OR (Uterine Stimulants) OR (Oxytocic Agents) OR (Oxytocic Effect) OR (Oxytocic Effects)<br><br>S3 S1 AND S2	1,563

ClinicalTrials .gov	Condition: (Cesarean Sections) OR (Cesarean Section) OR (Delivery, Abdominal) OR (Abdominal Deliveries) OR (Abdominal Deliver) OR (Deliveries, Abdominal) OR (Abdominal Delivery) OR "C-Section (OB)" OR "C Section (OB)" OR "C-Sections (OB)"<br><br>Intervention: Oxytocin OR Ocytocin OR Syntocinon OR Pitocin OR (Oxytocin Receptor) OR (Receptor, Oxytocin) OR (Oxytocin Receptors) OR (alpha hypophamine) OR "atonin o" OR "beta methyl beta mercaptobutyric acid oxytocin" OR "di sipidin" OR disipidin	90
Cochrane Library	#1 MeSH descriptor: [Cesarean Section] explode all trees<br>#2 (Cesarean Sections) OR (Cesarean Section) OR (Delivery, Abdominal) OR (Abdominal Deliveries) OR (Abdominal Deliver) OR (Deliveries, Abdominal) OR (Abdominal Delivery) OR "C-Section (OB)" OR "C Section (OB)" OR "C-Sections (OB)" OR (Postcesarean Section) OR (cesarean delivery) OR (Cesarean Section) OR (Elective cesarean section) OR (Elective cesarean delivery) OR Cesarean OR (Emergency cesarean section) OR (Emergency cesarean delivery) OR (Intrapartum cesarean section) OR (Intrapartum cesarean delivery) OR (Intra-partum cesarean section) OR (Intra-partum cesarean delivery) OR (Intra partum cesarean section) OR (Intra partum cesarean delivery)<br>#3 (Caesarean Section) OR (Caesarean Sections) OR (Postcaesarean Section) OR (caesarean delivery) OR (Elective caesarean section) OR (Elective caesarean delivery) OR Caesarean OR (Emergency caesarean section) OR (Emergency caesarean delivery) OR (Intrapartum caesarean section) OR (Intrapartum caesarean delivery) OR (Intra-partum caesarean section) OR (Intra-partum caesarean delivery) OR (Intra partum caesarean section) OR (Intra partum caesarean delivery)<br>#4 (Prelabor cesarean section) OR (Prelabor cesarean delivery) OR (Pre-labor cesarean delivery) OR (Pre-labor cesarean delivery) OR (Pre labor cesarean section) OR (Pre labor cesarean delivery)<br>#5 (Prelabour cesarean section) OR (Prelabour cesarean delivery) OR (Pre-labour cesarean delivery) OR (Pre-labour cesarean delivery) OR (Pre labour cesarean section) OR (Pre labour cesarean delivery)<br>#6 (Prelabor caesarean section) OR (Prelabor caesarean delivery) OR (Pre-labor caesarean delivery) OR (Pre-labor caesarean delivery) OR (Pre labor caesarean section) OR (Pre labor caesarean delivery)<br>#7 (Prelabour caesarean section) OR (Prelabour caesarean delivery) OR (Pre-labour caesarean delivery) OR (Pre-labour caesarean delivery) OR (Pre labour caesarean section) OR (Pre labour caesarean delivery)<br>#8 MeSH descriptor: [Cesarean Section, Repeat] explode all trees<br>#9 (Repeat Cesarean Section) OR (Cesarean Sections, Repeat) OR (Repeat Cesarean Sections) OR (Section, Repeat Cesarean) OR (Sections, Repeat Cesarean)<br>#10 (Repeat Caesarean Section) OR (Caesarean Sections, Repeat) OR (Repeat Caesarean Sections) OR (Section, Repeat Caesarean) OR (Sections, Repeat Caesarean) OR (Obstetric anesthesia)<br>#11 MeSH descriptor: [Delivery, Obstetric] explode all trees<br>#12 (Deliveries, Obstetric) OR (Obstetric Deliveries) OR (Obstetric Delivery)<br>#13 MeSH descriptor: [Labor Stage, Third] explode all trees<br>#14 (Labor Stages, Third) OR (Stage, Third Labor) OR (Stages, Third Labor) OR (Third Labor Stage) OR (Third Labor Stages) OR (Labor, Third Stage) OR (Third Stage Labor) OR "Third Stage of Labor" OR "Third-stage labor"<br>#15 (Labour Stage, Third) OR (Labour Stages, Third) OR (Stage, Third Labour) OR (Stages, Third Labour) OR (Third Labour Stage) OR (Third Labour Stages) OR (Labour, Third Stage) OR (Third Stage Labour) OR "Third Stage of Labour" OR "Third-stage labour"<br>#16 #1 OR #2 OR #3 OR #4 OR #5 OR #6 OR #7 OR #8 OR #9 OR #10 OR #11 OR #12 OR #13 OR #14 OR #15<br>#17 MeSH descriptor: [Oxytocin] explode all trees<br>#18 Oxytocin OR Ocytocin OR Syntocinon OR Pitocin<br>#19 MeSH descriptor: [Receptors, Oxytocin] explode all trees<br>#20 (Oxytocin Receptor) OR (Receptor, Oxytocin) OR (Oxytocin Receptors) OR (alpha hypophamine) OR "atonin o" OR "beta methyl beta mercaptobutyric acid oxytocin" OR "di sipidin" OR disipidin OR endopituitrin OR fetusin OR hypophysin OR hypophysine OR (intravenous oxytocin) OR mipareton OR neoxyn OR ocytocin OR orasthin OR orastina OR oxitocin OR "oxiton inj" OR oxitone OR oxystin OR oxytan OR (oxytocin 10 usp units in dextrose 5%) OR (oxytocin 20 usp units in dextrose 5%) OR (oxytocin 5 usp units in dextrose 5%) OR (oxytocin dimer) OR (oxytocin pentapeptide) OR "oxytocin s inj" OR oxytocine OR pareton OR partacon OR partocon OR "partocon inj" OR partolact OR partoxin OR physormon OR pitocin OR "pitocin inj" OR piton OR "piton s" OR "piton s inj" OR pituilobine OR pitupartin OR solvoxine OR synpitan OR (synthetic oxytocin inj) OR syntocinon OR "syntocinon inj" OR (syntocinon spray) OR tranoxy OR utedrin OR uteracon OR uterason OR "utron inj" OR xitocin<br>#21 MeSH descriptor: [Oxytocics] explode all trees<br>#22 Oxytocics OR (Oxytocic Drugs) OR (Drugs, Oxytocic) OR (Uterine Stimulants) OR (Stimulants, Uterine) OR (Oxytocic Agents) OR (Agents, Oxytocic) OR (Oxytocic Effect) OR (Effect, Oxytocic) OR (Oxytocic Effects) OR (Effects, Oxytocic)<br>#23 #17 OR #18 OR #19 OR #20 OR #21 OR #22<br>#24 #16 AND #23<br>#25 #24 in Trials	2,375
Embase	#1 'cesarean section'/exp OR 'cesarean section' OR 'birth, abdominal operation'/exp OR 'birth, abdominal operation' OR 'birth, caesarean'/exp OR 'birth, caesarean' OR 'caesarean birth'/exp OR 'caesarean birth' OR 'caesarean section'/exp OR 'caesarean section' OR 'caesarian birth'/exp OR 'caesarian birth' OR 'caesarian section'/exp OR 'caesarian section' OR 'cesarean delivery'/exp OR 'cesarean delivery' OR 'cesarian section'/exp OR 'cesarian section' OR 'elective repeat cesarean	3,804

For peer review only - http://bmjopen.bmj.com/site/about/guidelines.xhtml

	section'/exp OR 'elective repeat cesarean section' OR 'fetectomy'/exp OR 'fetectomy' OR 'repeated cesarotomy'/exp OR 'repeated cesarotomy' OR 'sectio caesarea'/exp OR 'sectio caesarea'<br><br>#2 'repeat cesarean section'/exp OR 'repeat cesarean section' OR 'cesarean section, repeat'/exp OR 'cesarean section, repeat' OR 'repeat cesarean section, elective'/exp OR 'repeat cesarean section, elective' OR 'repeat cesarotomy'/exp OR 'repeat cesarotomy' OR 'repeat section, cesarean'/exp OR 'repeat section, cesarean' OR 'repeated cesarean section'/exp OR 'repeated cesarean section' OR 'section, elective repeat cesarean'/exp OR 'section, elective repeat cesarean' OR 'section, repeat cesarean'/exp OR 'section, repeat cesarean'<br><br>#3 'obstetric delivery'/exp OR 'obstetric delivery' OR 'delivery'/exp OR delivery OR 'delivery pattern'/exp OR 'delivery pattern' OR 'delivery term'/exp OR 'delivery term' OR 'delivery, obstetric'/exp OR 'delivery, obstetric'<br><br>#4 'labor stage 3'/exp OR 'labor stage 3' OR 'delivery stage 3'/exp OR 'delivery stage 3' OR 'delivery stage iii'/exp OR 'delivery stage iii' OR 'labor stage iii'/exp OR 'labor stage iii' OR 'labor stage, third'/exp OR 'labor stage, third' OR 'labour stage 3'/exp OR 'labour stage 3' OR 'labour stage iii'/exp OR 'labour stage iii' OR 'labour stage, third'/exp OR 'labour stage, third' OR 'third delivery stage'/exp OR 'third delivery stage' OR 'third labor stage'/exp OR 'third labor stage' OR 'third labour stage'/exp OR 'third labour stage' OR 'third stage of delivery'/exp OR 'third stage of delivery' OR 'third stage of labor'/exp OR 'third stage of labor' OR 'third stage of labour'/exp OR 'third stage of labour'<br><br>#5 #1 OR #2 OR #3 OR #4<br><br>#6 'oxytocin' OR 'oxytocin'/exp OR oxytocin OR 'alpha hypophamine'/exp OR 'alpha hypophamine' OR 'atonin o'/exp OR 'atonin o' OR 'beta methyl beta mercaptobutyric acid oxytocin'/exp OR 'beta methyl beta mercaptobutyric acid oxytocin' OR 'di sipidin'/exp OR 'di sipidin' OR 'disipidin'/exp OR disipidin OR 'endopituitrin'/exp OR endopituitrin OR 'fetusin'/exp OR fetusin OR 'hypophysin'/exp OR hypophysin OR 'hypophysine'/exp OR hypophysine OR 'intravenous oxytocin'/exp OR 'intravenous oxytocin' OR 'mipareton'/exp OR mipareton OR 'neoxyn'/exp OR neoxyn OR 'ocytocin'/exp OR ocytocin OR 'opn 300'/exp OR 'opn 300' OR 'opn300'/exp OR opn300 OR 'orasthin'/exp OR orasthin OR 'orastina'/exp OR orastina OR 'oxitocin'/exp OR oxitocin OR 'oxiton inj'/exp OR 'oxiton inj' OR 'oxitone'/exp OR oxitone OR 'oxystin'/exp OR oxystin OR 'oxytan'/exp OR oxytan OR 'oxytocin 10 usp units in dextrose 5%'/exp OR 'oxytocin 10 usp units in dextrose 5%' OR 'oxytocin 20 usp units in dextrose 5%'/exp OR 'oxytocin 20 usp units in dextrose 5%' OR 'oxytocin 5 usp units in dextrose 5%'/exp OR 'oxytocin 5 usp units in dextrose 5%' OR 'oxytocin dimer'/exp OR 'oxytocin dimer' OR 'oxytocin pentapeptide'/exp OR 'oxytocin pentapeptide' OR 'oxytocin s inj'/exp OR 'oxytocin s inj' OR 'oxytocina'/exp OR oxytocina OR 'oxytocine'/exp OR oxytocine OR 'pareton'/exp OR pareton OR 'partacon'/exp OR partacon OR 'partocon'/exp OR partocon OR 'partocon inj'/exp OR 'partocon inj' OR 'partolact'/exp OR partolact OR 'partoxin'/exp OR partoxin OR 'physormon'/exp OR physormon OR 'pitocin'/exp OR pitocin OR 'pitocin inj'/exp OR 'pitocin inj' OR 'piton'/exp OR piton OR 'piton s'/exp OR 'piton s' OR 'piton s inj'/exp OR 'piton s inj' OR 'pituilobine'/exp OR pituilobine OR 'pitupartin'/exp OR pitupartin OR 'solvoxine'/exp OR solvoxine OR 'synpitan'/exp OR synpitan OR 'synthetic oxytocin inj'/exp OR 'synthetic oxytocin inj' OR 'syntocinon'/exp OR syntocinon OR 'syntocinon inj'/exp OR 'syntocinon inj' OR 'syntocinon spray'/exp OR 'syntocinon spray' OR 'ti 001'/exp OR 'ti 001' OR 'ti 114'/exp OR 'ti 114' OR 'ti001'/exp OR 'ti001' OR 'ti114'/exp OR 'ti114' OR 'tranoxy'/exp OR tranoxy OR 'tur 001'/exp OR 'tur 001' OR 'tur001'/exp OR 'tur001' OR 'utedrin'/exp OR utedrin OR 'uteracon'/exp OR uteracon OR 'uterason'/exp OR uterason OR 'utron inj'/exp OR 'utron inj' OR 'vagitocin'/exp OR vagitocin OR 'xitocin'/exp OR xitocin<br><br>#7 'oxytocin receptor'/exp OR 'oxytocin receptor' OR 'neuron, oxytocinergic'/exp OR 'neuron, oxytocinergic' OR 'oxytocinergic nerve cell'/exp OR 'oxytocinergic nerve cell' OR 'oxytocinergic neuron'/exp OR 'oxytocinergic neuron' OR 'receptor, oxytocin'/exp OR 'receptor, oxytocin' OR 'receptors, oxytocin'/exp OR 'receptors, oxytocin'<br><br>#8 'oxytocic agent'/exp OR 'oxytocic agent' OR 'labor inducing drug'/exp OR 'labor inducing drug' OR 'labour inducing drug'/exp OR 'labour inducing drug' OR 'ocytocic agent'/exp OR 'ocytocic agent' OR 'oxytocic activity'/exp OR 'oxytocic activity' OR 'oxytocic substance'/exp OR 'oxytocic substance' OR 'oxytocics'/exp OR oxytocics<br><br>#9 #6 OR #7 OR #8<br>#10 #5 AND #9<br>#11 #10 AND [embase]/lim NOT ([embase]/lim AND [medline]/lim)	
Global Index Medicus	MH:Cesárea OR MH:"Cesarean Section" OR Cesárea OR (Cesarean Section) OR (Parto Abdominal) OR (Abdominal Deliveries) OR (Abdominal Delivery) OR (C Section (OB)) OR (C-Section (OB)) OR (C-Sections (OB)) OR (Caesarean Section) OR (Caesarean Sections) OR (Cesarean Sections) OR (Postcesarean Section) OR MH:E04.520.252.500$ OR MH:"Cesárea Repetida" OR MH:"Cesarean Section, Repeat" OR MH:Recesariana OR (Cesárea Repetida) OR Recesariana OR (Repeat Cesarean Section) OR (Repeat Cesarean Sections) OR (Operación Cesárea Repetida) OR MH:E04.520.252.500.150$ OR MH:"Parto Obstétrico" OR MH:"Delivery, Obstetric" OR (Parto Obstétrico) OR (Liberação Obstétrica) OR (Obstetric Deliveries) OR (Obstetric Delivery) OR (Liberación Obstétrica) OR MH:E04.520.252$ OR MH:"Terceira Fase do Trabalho de Parto" OR MH:"Labor Stage, Third" OR MH:"Tercer Periodo del Trabajo de Parto" OR (Terceira Fase do Trabalho de Parto) OR (Tercer Periodo del Trabajo de Parto) OR (Terceiro Estágio do Trabalho de	421

For peer review only

	Parto) OR (Terceiro Período do Trabalho de Parto) OR (Third Labor Stage) OR (Third Labor Stages) OR (Third Stage Labor) OR MH:G08.686.784.769.326.500.110$<br>AND<br>MH:Oxitocina OR MH:Oxytocin OR MH:Ocitocina OR Oxitocina OR Oxytocin OR Ocitocina OR Ocytocin OR Pitocin OR Syntocinon OR MH:D06.472.699.631.692.433$ OR MH:D12.644.548.691.692.433$ OR MH:"Receptores de Ocitocina" OR MH:"Receptors, Oxytocin" OR MH:"Receptores de Oxitocina" OR (Receptores de Ocitocina) OR (Receptores de Oxitocina) OR (Oxytocin Receptor) OR (Oxytocin Receptors) OR MH:D12.776.543.750.695.630$ OR MH:D12.776.543.750.720.600.630$ OR MH:D12.776.543.750.750.555.630$ OR MH:D12.776.543.750.750.660.600$ OR MH:Ocitócicos OR MH:Oxytocics OR MH:Oxitócicos OR Ocitócicos OR Oxytocics OR Oxitócicos OR (Efeito Ocitócico) OR (Estimulantes Uterinos) OR (Oxytocic Agents) OR (Oxytocic Drugs) OR (Oxytocic Effect) OR (Oxytocic Effects) OR (Uterine Stimulants) OR (Efecto Oxitócico) OR MH:D27.505.696.875.737$ OR MH:D27.505.954.705.737$	
MEDLINE (via PubMed)	#1 "Cesarean Section"[Mesh] OR (Cesarean Sections) OR (Cesarean Section) OR (Delivery, Abdominal) OR (Abdominal Deliveries) OR (Abdominal Deliver) OR (Deliveries, Abdominal) OR (Abdominal Delivery) OR "C-Section (OB)" OR "C Section (OB)" OR "C-Sections (OB)" OR (Postcesarean Section) OR (cesarean delivery) OR (Cesarean Section) OR (Elective cesarean section) OR (Elective cesarean delivery) OR Cesarean OR (Emergency cesarean section) OR (Emergency cesarean delivery) OR (Intrapartum cesarean section) OR (Intrapartum cesarean delivery) OR (Intra-partum cesarean section) OR (Intra-partum cesarean delivery) OR (Intra partum cesarean section) OR (Intra partum cesarean delivery)<br><br>#2 (Caesarean Section) OR (Caesarean Sections) OR (Postcaesarean Section) OR (caesarean delivery) OR (Elective caesarean section) OR (Elective caesarean delivery) OR Caesarean OR (Emergency caesarean section) OR (Emergency caesarean delivery) OR (Intrapartum caesarean section) OR (Intrapartum caesarean delivery) OR (Intra-partum caesarean section) OR (Intra-partum caesarean delivery) OR (Intra partum caesarean section) OR (Intra partum caesarean delivery)<br><br>#3 (Prelabor cesarean section) OR (Prelabor cesarean delivery) OR (Pre-labor cesarean delivery) OR (Pre-labor cesarean delivery) OR (Pre labor cesarean section) OR (Pre labor cesarean delivery)<br><br>#4 (Prelabour cesarean section) OR (Prelabour cesarean delivery) OR (Pre-labour cesarean delivery) OR (Pre-labour cesarean delivery) OR (Pre labour cesarean section) OR (Pre labour cesarean delivery)<br><br>#5 (Prelabor caesarean section) OR (Prelabor caesarean delivery) OR (Pre-labor caesarean delivery) OR (Pre-labor caesarean delivery) OR (Pre labor caesarean section) OR (Pre labor caesarean delivery)<br>#6 (Prelabour caesarean section) OR (Prelabour caesarean delivery) OR (Pre-labour caesarean delivery) OR (Pre-labour caesarean delivery) OR (Pre labour caesarean section) OR (Pre labour caesarean delivery)<br><br>#7 "Cesarean Section, Repeat’[Mesh] OR (Repeat Cesarean Section) OR (Cesarean Sections, Repeat) OR (Repeat Cesarean Sections) OR (Section, Repeat Cesarean) OR (Sections, Repeat Cesarean)<br><br>#8 (Repeat Caesarean Section) OR (Caesarean Sections, Repeat) OR (Repeat Caesarean Sections) OR (Section, Repeat Caesarean) OR (Sections, Repeat Caesarean) OR (Obstetric anesthesia)<br><br>#9 "Delivery, Obstetric"[Mesh] OR (Deliveries, Obstetric) OR (Obstetric Deliveries) OR (Obstetric Delivery)<br><br>#10 "Labor Stage, Third"[Mesh] OR (Labor Stages, Third) OR (Stage, Third Labor) OR (Stages, Third Labor) OR (Third Labor Stage) OR (Third Labor Stages) OR (Labor, Third Stage) OR (Third Stage Labor) OR "Third Stage of Labor" OR "Third-stage labor"<br><br>#11 (Labour Stage, Third) OR (Labour Stages, Third) OR (Stage, Third Labour) OR (Stages, Third Labour) OR (Third Labour Stage) OR (Third Labour Stages) OR (Labour, Third Stage) OR (Third Stage Labour) OR "Third Stage of Labour" OR "Third-stage labour"<br><br>#12 #1 OR #2 OR #3 OR #4 OR #5 OR #6 OR #7 OR #8 OR #9 OR #10 OR #11<br><br>#13 "Oxytocin"[Mesh] OR Oxytocin OR Ocytocin OR Syntocinon OR Pitocin<br><br>#14 "Receptors, Oxytocin"[Mesh] OR (Oxytocin Receptor) OR (Receptor, Oxytocin) OR (Oxytocin Receptors) OR (alpha hypophamine) OR "atonin o" OR "beta methyl beta mercaptobutyric acid oxytocin" OR "di sipidin" OR disipidin OR endopituitrin OR fetusin OR hypophysin OR hypophysine OR (intravenous oxytocin) OR mipareton OR neoxyn OR ocytocin OR orasthin OR orastina OR oxitocin OR "oxiton inj" OR oxitone OR oxystin OR oxytan OR (oxytocin 10 usp units in dextrose 5%) OR (oxytocin 20 usp units in dextrose 5%) OR (oxytocin 5 usp units in dextrose 5%) OR (oxytocin dimer) OR (oxytocin pentapeptide) OR "oxytocin s inj" OR oxytocine OR pareton OR partacon OR partocon OR "partocon inj" OR partolact OR partoxin OR physormon OR pitocin OR "pitocin inj" OR piton OR "piton s" OR "piton s inj" OR pituilobine OR pitupartin OR solvoxine OR synpitan OR	7,944

For peer review only

	(synthetic oxytocin inj) OR syntocinon OR "syntocinon inj" OR (syntocinon spray) OR tranoxy OR utedrin OR uteracon OR uterason OR "utron inj" OR xitocin<br>#15 "Oxytocics"[Mesh] OR Oxytocics OR (Oxytocic Drugs) OR (Drugs, Oxytocic) OR (Uterine Stimulants) OR (Stimulants, Uterine) OR (Oxytocic Agents) OR (Agents, Oxytocic) OR (Oxytocic Effect) OR (Effect, Oxytocic) OR (Oxytocic Effects) OR (Effects, Oxytocic)<br><br>#16 "Oxytocics" [Pharmacological Action]<br>#17 #13 OR #14 OR #15 OR #16<br>#18 #12 AND #17<br>#19 ((clinical[Title/Abstract] AND trial[Title/Abstract]) OR clinical trials as topic[MeSH Terms] OR clinical trial[Publication Type] OR random*[Title/Abstract] OR random allocation[MeSH Terms] OR therapeutic use[MeSH Subheading])<br>#20 #18 AND #19	
Opengrey	Cesarean Sections) OR (Cesarean Section) OR (Delivery, Abdominal) OR (Abdominal Deliveries) OR (Abdominal Deliver) OR (Deliveries, Abdominal) OR (Abdominal Delivery) OR "C-Section (OB)" OR "C Section (OB)" OR "C-Sections (OB)" OR (Postcesarean Section) OR (cesarean delivery) OR (Cesarean Section) OR (Elective cesarean section) OR (Elective cesarean delivery) OR Cesarean OR (Emergency cesarean section) OR (Emergency cesarean delivery) OR (Intrapartum cesarean section) OR (Intrapartum cesarean delivery) OR (Intra-partum cesarean section) OR (Intra-partum cesarean delivery) OR (Intra partum cesarean section) OR (Intra partum cesarean delivery) OR (Caesarean Section) OR (Caesarean Sections) OR (Postcaesarean Section) OR (caesarean delivery) OR (Elective caesarean section) OR (Elective caesarean delivery) OR Caesarean OR (Emergency caesarean section) OR (Emergency caesarean delivery) OR (Intrapartum caesarean section) OR (Intrapartum caesarean delivery) OR (Intra-partum caesarean section) OR (Intra-partum caesarean delivery) OR (Intra partum caesarean section) OR (Intra partum caesarean delivery) OR (Prelabor cesarean section) OR (Prelabor cesarean delivery) OR (Pre-labor cesarean delivery) OR (Pre-labor cesarean delivery) OR (Pre labor cesarean section) OR (Pre labor cesarean delivery) OR (Prelabour cesarean section) OR (Prelabour cesarean delivery) OR (Pre-labour cesarean delivery) OR (Pre-labour cesarean delivery) OR (Pre labour cesarean section) OR (Pre labour cesarean delivery) OR (Prelabor caesarean section) OR (Prelabor caesarean delivery) OR (Pre-labor caesarean delivery) OR (Pre-labor caesarean delivery) OR (Pre labor caesarean section) OR (Pre labor caesarean delivery) OR (Prelabour caesarean section) OR (Prelabour caesarean delivery) OR (Pre-labour caesarean delivery) OR (Pre-labour caesarean delivery) OR (Pre labour caesarean section) OR (Pre labour caesarean delivery) OR (Repeat Cesarean Section) OR (Cesarean Sections, Repeat) OR (Repeat Cesarean Sections) OR (Section, Repeat Cesarean) OR (Sections, Repeat Cesarean) OR (Repeat Caesarean Section) OR (Caesarean Sections, Repeat) OR (Repeat Caesarean Sections) OR (Section, Repeat Caesarean) OR (Sections, Repeat Caesarean) OR (Obstetric anesthesia) OR (Deliveries, Obstetric) OR (Obstetric Deliveries) OR (Obstetric Delivery) OR (Labor Stages, Third) OR (Stage, Third Labor) OR (Stages, Third Labor) OR (Third Labor Stage) OR (Third Labor Stages) OR (Labor, Third Stage) OR (Third Stage Labor) OR "Third Stage of Labor" OR "Third-stage labor" OR (Labour Stage, Third) OR (Labour Stages, Third) OR (Stage, Third Labour) OR (Stages, Third Labour) OR (Third Labour Stage) OR (Third Labour Stages) OR (Labour, Third Stage) OR (Third Stage Labour) OR "Third Stage of Labour" OR "Third-stage labour"	126
SciELO	((Cesarean Sections) OR (Cesarean Section) OR (Abdominal Deliveries) OR (Abdominal Deliver) OR (Abdominal Delivery) OR (C-Section (OB)) OR (C Section (OB)) OR (C-Sections (OB)) OR (Postcesarean Section) OR (cesarean delivery) OR (Cesarean Section) OR (Elective cesarean section) OR (Elective cesarean delivery) OR Cesarean OR (Emergency cesarean section) OR (Emergency cesarean delivery) OR (Intrapartum cesarean section) OR (Intrapartum cesarean delivery) OR (Intra-partum cesarean section) OR (Intra-partum cesarean delivery) OR (Intra partum cesarean section) OR (Intra partum cesarean delivery) OR (Caesarean Section) OR (Caesarean Sections) OR (Postcaesarean Section) OR (caesarean delivery) OR (Elective caesarean section) OR (Elective caesarean delivery) OR Caesarean OR (Emergency caesarean section) OR (Emergency caesarean delivery) OR (Intrapartum caesarean section) OR (Intrapartum caesarean delivery) OR (Intra-partum caesarean section) OR (Intra-partum caesarean delivery) OR (Intra partum caesarean section) OR (Intra partum caesarean delivery) OR (Prelabor cesarean section) OR (Prelabor cesarean delivery) OR (Pre-labor cesarean delivery) OR (Pre-labor cesarean delivery) OR (Pre labor cesarean section) OR (Pre labor cesarean delivery) OR (Prelabour cesarean section) OR (Prelabour cesarean delivery) OR (Pre-labour cesarean delivery) OR (Pre-labour cesarean delivery) OR (Pre labour cesarean section) OR (Pre labour cesarean delivery) OR (Prelabor caesarean section) OR (Prelabor caesarean delivery) OR (Pre-labor caesarean delivery) OR (Pre-labor caesarean delivery) OR (Pre labor caesarean section) OR (Pre labor caesarean delivery) OR (Prelabour caesarean section) OR (Prelabour caesarean delivery) OR (Pre-labour caesarean delivery) OR (Pre-labour caesarean delivery) OR (Pre labour caesarean section) OR (Pre labour caesarean delivery) OR (Repeat Cesarean Section) OR (Repeat Cesarean Sections) OR (Repeat Caesarean Section) OR (Repeat Caesarean Sections) OR (Obstetric anesthesia) OR (Obstetric Deliveries) OR (Obstetric Delivery) OR (Third Labor Stage) OR (Third Labor Stages) OR (Third Stage Labor) OR (Third Stage of Labor) OR (Third-stage labor) OR (Third Labour Stage) OR (Third Labour Stages) OR (Third Stage Labour) OR (Third Stage of Labour) OR (Third-stage labour))<br>AND<br>(Oxytocin OR Ocytocin OR Syntocinon OR Pitocin OR (Oxytocin Receptor) OR (Oxytocin Receptors) OR (alpha hypophamine) OR (atonin o) OR (beta methyl beta mercaptobutyric acid oxytocin) OR (di sipidin) OR disipidin OR endopituitrin OR fetusin OR hypophysin OR hypophysine OR (intravenous oxytocin) OR mipareton OR neoxyn OR ocytocin OR orasthin OR orastina OR oxitocin OR (oxiton inj) OR oxitone OR oxystin OR oxytan OR (oxytocin 10 usp units in dextrose 5%) OR (oxytocin 20 usp	91

	units in dextrose 5%) OR (oxytocin 5 usp units in dextrose 5%) OR (oxytocin dimer) OR (oxytocin pentapeptide) OR (oxytocin s inj) OR oxytocine OR pareton OR partacon OR partocon OR (partocon inj) OR partolact OR partoxin OR physormon OR pitocin OR (pitocin inj) OR piton OR (piton s) OR (piton s inj) OR pituilobine OR pitupartin OR solvoxine OR synpitan OR (synthetic oxytocin inj) OR syntocinon OR (syntocinon inj) OR (syntocinon spray) OR tranoxy OR utedrin OR uteracon OR uterason OR (utron inj) OR xitocin OR Oxytocics OR (Oxytocic Drugs) OR (Uterine Stimulants) OR (Oxytocic Agents) OR (Oxytocic Effect) OR (Oxytocic Effects))	
WHO - ICTRP	Condition: (Cesarean Sections) OR (Cesarean Section) OR (Delivery, Abdominal) OR (Abdominal Deliveries) OR (Abdominal Deliver) OR (Deliveries, Abdominal) OR (Abdominal Delivery) OR "C-Section (OB)" OR "C Section (OB)" OR "C-Sections (OB)"<br><br>Intervention: Oxytocin OR Ocytocin OR Syntocinon OR Pitocin OR (Oxytocin Receptor) OR (Receptor, Oxytocin) OR (Oxytocin Receptors) OR (alpha hypophamine) OR "atonin o" OR "beta methyl beta mercaptobutyric acid oxytocin" OR "di sipidin" OR disipidin	95
Total		16,883

For peer review only

Supplementary file 3. Excluded studies in systematic review on route of oxytocin administration

Excluded studies with reasons

Study	*Reason for exclusion*
Zarzur E. A Ocitocina e a Operação Cesariana. Rev Bras Anest. 1992;42(4):293-5.	Did not compare different routes
Garza-Hinojosa A, González-Cordero G. Cambios hemodinámicos y electrocardiográfi cos con el uso de oxitocina en bolo durante la cesárea. Ver Mex Anest. 2015;38(S1): S257-62.	Did not compare different routes
Singh KP, Kameshore N, Kamei H. Prophylactic intramuscular injection of oxytocin vs intravenous infusion of oxytocin to minimize blood loss at caesarean section. Int J Gynecol Obstet. 2015;131(Suppl. 5): E290.	Insufficient information (Abstract-authors did not reply to contact)

For peer review only

Supplementary file 4. Details of trials on different routes of oxytocin administration at CS

Akinaga 2016	
**Method**	Double blind, placebo controlled RCT<br>Study protocol UMIN000007577 (https://upload.umin.ac.jp/cgi-open-bin/ctr_e/ctr_view.cgi?recptno=R000008709)
**Participants**	**Setting**: women managed at Hamamatsu University hospital, Japan<br>**Dates of recruitment**: April to October 2012<br>**Inclusion criteria**: 40 women, ASA status I or II with a single fetus at ≥36 weeks scheduled for elective CS under spinal anesthesia<br>**Exclusion criteria**: conditions predisposing to uterine atony and postpartum hemorrhage, such as placenta previa, multiple gestations, polyhydramnios, previous uterine atony and post-partum bleeding, macrosomia, uterine fibroids, bleeding diathesis, and pre-eclampsia
**Interventions**	After clamping of umbilical cord<br>**Group 1** (intramyometrial): bolus injection of oxytocin (0.07 IU/kg) diluted to 2 mL saline was slowly (over 30 seconds) injected into the myometrium of the uterine fundus and an IV saline infusion (10 mL) was also given.<br>(n=20 randomized)<br>**Group 2**: (intravenous): 2 mL bolus saline injection was injected into the myometrium and an IV oxytocin injection (0.07 IU/kg) diluted to 10 mL with saline was also given.<br>(n=20 randomized)<br>**Both groups:** After bolus injections, both groups received oxytocin at 0.1 IU/kg in 500 mL of lactated Ringer's solution and continuously administered IV at a rate of 100 mL/h.
**Outcomes**	Volume of blood loss during CS (volume in suction bottle). Additional uterotonics. Adverse effects (hypotension, nausea/vomiting, headache, flushing)
**Notes**	Funding: not reported<br>Conflict of interest: none

For peer review only

Dennehy 1998	
**Method**	Double blind, placebo controlled RCT<br>no protocol
**Participants**	**Setting**: women managed in hospital (no other information) in Canada<br>**Dates of recruitment**: No information<br>**Inclusion criteria**: 40 ASA I or II women with singleton fetus scheduled for elective CS at term (> 37 wks).<br>**Exclusion criteria**: age <18 yr, contraindication to spinal anaesthesia, polyhydramnios, placenta praevia, uterine fibroids, hypertension or cardiac disease.
**Interventions**	Immediately after placental removal:<br>**Group 1** (IV bolus): 0.5 ml of 10 IU/ml oxytocin in unknown time and an intramyometrial 2 ml saline injection. Total bolus injection dose: 5 IU, Total bolus injection time: NI.<br>(n=20 randomized)<br>**Group 2** (Intramyometrial bolus): 2 ml of 10 IU/ml oxytocin into the uterine fundus and 0.5 mL saline IV bolus. Total bolus injection dose: 20 IU, Total bolus injection time: NI.<br>(n=20 randomized)
**Outcomes**	Need for additional uterotonics, hypotension
**Notes**	Funding: not reported<br>Conflict of interest: none

For peer review only

Mangla 2012	
**Method**	Double blind RCT<br>No protocol.
**Participants**	**Setting**: Guru Gobind Singh Hospital, Jamnagar, Gujarat, India<br>**Dates of recruitment**: No information<br>**Inclusion criteria:** "women operated by lower segment cesarean section". No other information<br>**Exclusion criteria:** No information
**Interventions**	**Group 1**: IV infusion 20 IU/500 ml Ringer after placental detachment, no information on rate or duration of infusion<br>(n=50 randomized).<br>**Group 2**: Intra-myometrial 5 IU in 10 cc normal saline; 5 cc injected in each cornu of the uterus after separation of placenta.<br>(n=50 randomized)<br>**Group 3**: Intra-myometrial 5 IU in 10 cc normal saline; 5 cc injected in each cornu of the uterus before separation of placenta.<br>(n=50 randomized) This group was not included in the SR because it was identical to Group 2 but given at a different timing
**Outcomes**	Volume of blood loss during CS and 1st hour after (measured by adding volume in graduated suction jar and number of soaked sponges; no information on how blood loss was measured in the 1st hour after).<br>Additional uterotonic. Nausea/vomiting requiring antiemetic.
**Notes**	Funding: none<br>Conflict of interest: none

CS: cesarean section, IU: international units, IV: intravenous, RCT: randomized controlled trial

For peer review only

Supplementary file 5: Postpartum hemorrhage prevention core outcome sets[1] in trials on different routes of oxytocin administration at cesarean section

	Outcome	Measure (in each group)	Study		
			Akinaga 2016	Dennehy 1998	Mangla 2012
1	Blood loss (from birth up to cessation of active bleeding)				
	≥ 1000 ml	N women with this loss	no	no	no
	total volume	mean or median volume	yes	no	yes
2	Shock (defined by trialists)	N women	no	no	no
3	Maternal death				
	PPH-related maternal deaths	N women	no	no	no
	all cause mortality	N women	no	no	no
4	Blood transfusion				
	Wm receiving any RBC product	N women	no	no	no
	total volume	mean or median RBC units	no	no	no
5	Transfer to higher level of care	N women	no	no	no
6	Use of additional uterotonics	N women	yes	yes	yes
7	Adverse effects (defined by trialists)	N women	yes (headache, nausea/vomiting, flushing, hypotension)	yes (hypotension)	yes (nausea/ vomiting)
8	Breastfeeding	N women	no	no	no
9	Patient reported outcomes	N women	no	no	no

N: number, PPH: post-partum hemorrhage, RBC: red blood cell

1.Source: Meher S, Cuthbert A, Kirkham JJ, et al. Core outcome sets for prevention and treatment of postpartum haemorrhage: an international Delphi consensus study. BJOG. 2019 Jan;126(1):83-93.

For peer review only

Supplementary file 6. Risk of bias details

Judgments and justifications for risk of bias assessments in trials on different routes of oxytocin administration at cesarean section

Domain	Akinaga 2016		
1	Sequence generation	UNCLEAR	No information on random sequence generation
2	Allocation concealment	LOW	*'An anesthesiologist unrelated to this research selected one of two opaque envelopes containing a letter indicating the group name'*
3	Blinding participants/personnel (objective outcomes)	LOW	*'The subject, care providers, and investigators were blinded to patient allocation until all data collection was completed.'*
3	Blinding participants/personnel (subjective outcomes)	LOW	*'The subject, care providers, and investigators were blinded to patient allocation until all data collection was completed.'*
4	Blinding outcome assessors (objective outcomes)	LOW	*'The i.m.y. group received a bolus injection of oxytocin (0.07 IU/kg) diluted to 2 mL with saline into the myometrium of the uterine fundus during slow (30-s) i.v. infusion of saline (10 mL). Conversely, the i.v. group received a 2-mL bolus injection of saline into the myometrium during slow (30-s) i.v. infusion of oxytocin (0.07 IU/kg) diluted to 10mL with saline.'*
4	Blinding outcome assessors (subjective outcomes)	LOW	*The subject, care providers, and investigators were blinded to patient allocation until all data collection was completed.'*
5	Incomplete outcome	HIGH	*´Nine patients were excluded from the study: eight for protocol deviation and one for adenomyosis.'* High rate of loss (20%), with no information about which was the original group that the participants had been randomized to. ITT was not used.
6	Selective reporting	LOW	All outcomes described in protocol were reported
7	Other bias	LOW	There are probably no other sources of bias

For peer review only

Domain	Dennehy 1998		
1	Sequence generation	LOW	*´Participants were assigned to one of two study groups, according to a computer generated series of random numbers.'*
2	Allocation concealment	UNCLEAR	No information on allocation concealment
3	Blinding participants/personnel (objective outcomes)	LOW	*'The hospital pharmacy prepared the syringes containing the study solutions trader aseptic conditions according to the random table and delivered them to the delivery room in a plastic bag.'*
3	Blinding participants/personnel (subjective outcomes)	does not apply	No subjective outcomes
4	Blinding outcome assessors (objective outcomes)	LOW	*´The injections were conducted in a double blind fashion where neither the attending anaesthetist or obstetrician had knowledge of the content of the syringes.'*
4	Blinding outcome assessors (subjective outcomes)	does not apply	No subjective outcomes
5	Incomplete outcome	LOW	*One patient did not complete the study protocol because the attending anaesthetist was unable to locate the subarachnoid space and she, therefore, received general anaesthesia for Caesarean section*'. Low rate of losses (2%).
6	Selective reporting	UNCLEAR	No study protocol
7	Other bias	LOW	There are probably no other sources of bias

Domain	Mangla 2012		
1	Sequence generation	UNCLEAR	No information on random sequence generation
2	Allocation concealment	UNCLEAR	No information on allocation concealment
3	Blinding participants/personnel (objective outcomes)	LOW	Blinding of participants was unconfirmed. Objective outcomes are unlikely to be influenced by this fact.
3	Blinding participants/personnel (subjective outcomes)	HIGH	Blinding of participants and personnel was unconfirmed. Subjective outcomes are likely to be influenced by this fact.
4	Blinding outcome assessors (objective outcomes)	LOW	Blinding of outcome assessors was unconfirmed. The judgment of objective outcomes is unlikely to be influenced by this fact.
4	Blinding outcome assessors (subjective outcomes)	HIGH	Blinding of participants and personnel was unconfirmed. Subjective outcomes are likely to be influenced by this fact.
5	Incomplete outcome	LOW	The outcome data of all randomized participants were presented
6	Selective reporting	UNCLEAR	No study protocol
7	Other bias	UNCLEAR	No information on baseline characteristics

For peer review only

Supplementary file 5, continuation

Figure S5. Risk of bias graph: review authors' judgements about each risk of bias item presented as percentages across all included studies.

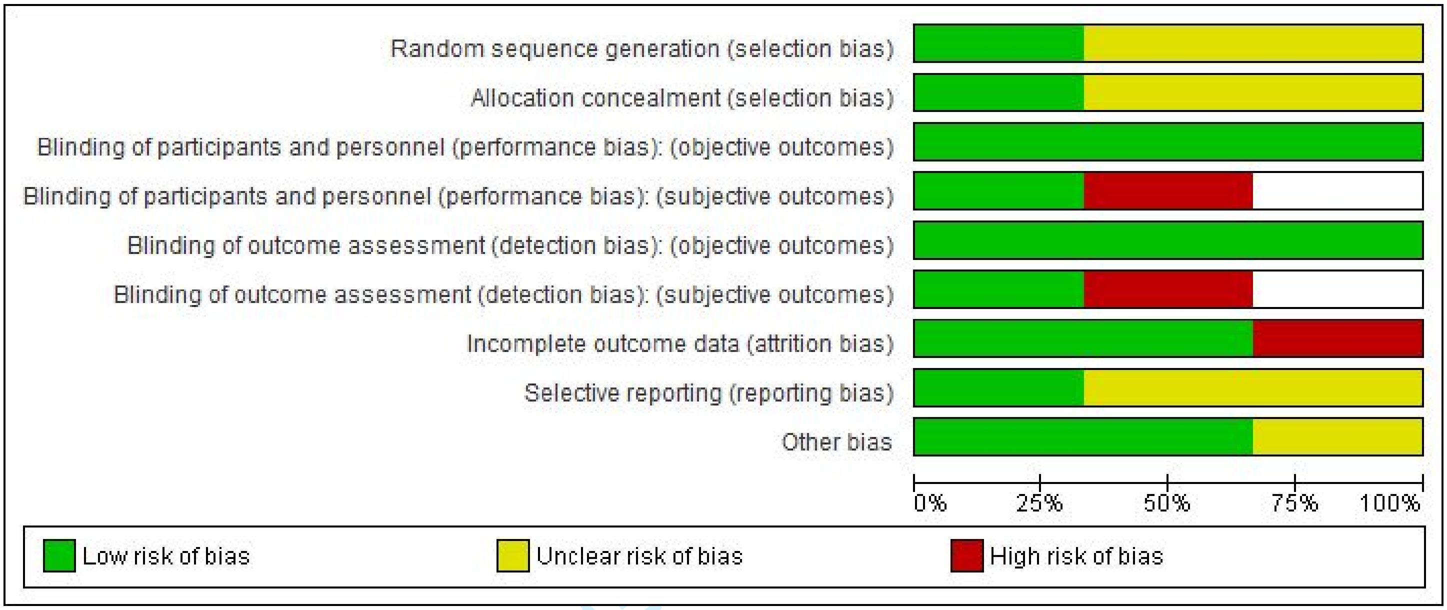

Supplementary file 7. Summary of findings table and GRADE: intramyometrial versus intravenous oxytocin at cesarean section

Intramyometrial compared to Intravenous prophylactic oxytocin for women giving birth by cesarean

Patient or population: women giving birth by cesarean
Setting: hospital
Intervention: intramyometrial oxytocin
Comparison: intravenous oxytocin

Outcomes	Anticipated absolute effects* (95% CI)		Relative effect (95% CI)	N of participants (studies)	Certainty of the evidence (GRADE)	Comments
	Risk with Intravenous prophylactic oxytocin	Risk with Intramyometrial				
Post-Partum Hemorrhage	60 per 1000	**8 per 1000** (1 to 162)	**RR 0.14** (0.01 to 2.70)	100 (1 RCT)	⊕⊕◯◯ LOW [a]	Mangla 2012: Blood loss > 900 mL.
Need for additional uterotonic	56 per 1000	**40 per 1000** (3 to 547)	**RR 0.71** (0.05 to 9.73)	179 (3 RCTs)	⊕◯◯◯ VERY LOW [a,b]	
Hypotension not requiring epinephrine	200 per 1000	**200 per 1000** (58 to 690)	**RR 1.00** (0.29 to 3.45)	40 (1 RCT)	⊕⊕◯◯ LOW [a]	
Hypotension requiring ephedrine	See comment	See comment	Not estimable	79 (2 RCTs)	⊕⊕◯◯ LOW [c]	The 2 trials had no events.
Nausea and/or Vomiting	157 per 1000	**22 per 1000** (5 to 123)	**RR 0.14** (0.03 to 0.78)	140 (2 RCTs)	⊕⊕◯◯ LOW [d,e]	
Headache	0 per 1000	**0 per 1000** (0 to 0)	**RR 3.00** (0.13 to 69.52)	40 (1 RCT)	⊕⊕◯◯ LOW [a]	
Facial flushing	100 per 1000	**50 per 1000** (5 to 508)	**RR 0.50** (0.05 to 5.08)	40 (1 RCT)	⊕⊕◯◯ LOW [a]	
Volume of blood loss	The mean volume of blood loss was **664.2** mL	MD **57.40 mL lower** (101.71 lower to 13.09 lower)	-	40 (1 RCT)	⊕⊕◯◯ LOW [f]	

***The risk in the intervention group** (and its 95% confidence interval) is based on the assumed risk in the comparison group and the **relative effect** of the intervention (and its 95% CI).
CI: Confidence interval; **RR:** Risk ratio; **MD:** Mean difference

GRADE Working Group grades of evidence
High certainty: We are very confident that the true effect lies close to that of the estimate of the effect
Moderate certainty: We are moderately confident in the effect estimate: The true effect is likely to be close to the estimate of the effect, but there is a possibility that it is substantially different
Low certainty: Our confidence in the effect estimate is limited: The true effect may be substantially different from the estimate of the effect
Very low certainty: We have very little confidence in the effect estimate: The true effect is likely to be substantially different from the estimate of effect

Explanations

a. Evidence certainty downgraded -2 due to very serious imprecision (very small number of events, and wide 95% CI crossing the line of no effect).
b. Evidence certainty downgraded -1 due to inconsistency (I2 61%)
c. Evidence certainty downgraded -2 due to very serious imprecision (lack of events).
d. Evidence certainty downgraded -1 due to study limitation (lack of blinding of participants, subjective outcome)
e. Evidence certainty downgraded -1 due to serious imprecision (small sample size)
f. Evidence certainty downgraded -2 due to very serious imprecision (small sample size and wide 95% confidence interval).

Supplementary file 8. Metanalyses of intramyometrial versus intravenous oxytocin at caesarean

The standard route used for prophylactic oxytocin at caesarean is intravenous (IV); this was the control group. Intramyometrial (IMY) oxytocin was the intervention group being tested.

A. Forest plot of comparison: Intramyometrial versus Intravenous oxytocin, Outcome: Post-Partum Hemorrhage.

Study or Subgroup	Intramyometrial oxytocin Events	Intramyometrial oxytocin Total	Intravenous oxytocin Events	Intravenous oxytocin Total	Weight	Risk Ratio M-H, Random, 95% CI
Mangla 2012	0	50	3	50	100.0%	0.14 [0.01, 2.70]
**Total (95% CI)**		**50**		**50**	**100.0%**	**0.14 [0.01, 2.70]**
Total events	0		3			
Heterogeneity: Not applicable						
Test for overall effect: Z = 1.30 (P = 0.19)						

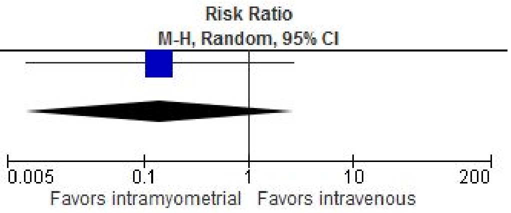

Mangla 2012: Post-partum blood loss > 900 mL

B. Forest plot of comparison: Intramyometrial versus Intravenous oxytocin, Outcome: Need for additional uterotonic.

Study or Subgroup	Intramyometrial oxytocin Events	Intramyometrial oxytocin Total	Intravenous oxytocin Events	Intravenous oxytocin Total	Weight	Risk Ratio M-H, Random, 95% CI
Akinaga 2016	4	20	2	20	60.7%	2.00 [0.41, 9.71]
Dennehy 1998	0	20	0	19		Not estimable
Mangla 2012	0	50	3	50	39.3%	0.14 [0.01, 2.70]
**Total (95% CI)**		**90**		**89**	**100.0%**	**0.71 [0.05, 9.73]**
Total events	4		5			
Heterogeneity: Tau² = 2.30; Chi² = 2.58, df = 1 (P = 0.11); I² = 61%						
Test for overall effect: Z = 0.26 (P = 0.80)						

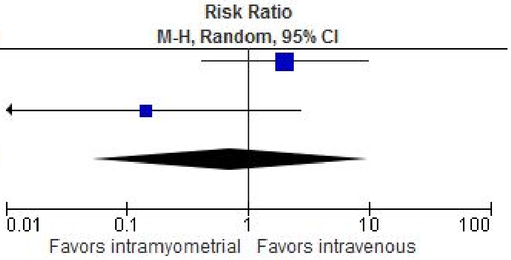

C. Forest plot of comparison: Intramyometrial versus Intravenous oxytocin, Outcome: Hypotension overall.

Study or Subgroup	Intramyometrial oxytocin Events	Intramyometrial oxytocin Total	Intravenous oxytocin Events	Intravenous oxytocin Total	Weight	Risk Ratio M-H, Random, 95% CI
**2.3.1 Hypotension not requiring epinephrine***						
Akinaga 2016	4	20	4	20	100.0%	1.00 [0.29, 3.45]
**Subtotal (95% CI)**		**20**		**20**	**100.0%**	**1.00 [0.29, 3.45]**
Total events	4		4			
Heterogeneity: Not applicable						
Test for overall effect: Z = 0.00 (P = 1.00)						
**2.3.2 Hypotension requiring ephedrine****						
Akinaga 2016	0	20	0	20		Not estimable
Dennehy 1998	0	20	0	19		Not estimable
**Subtotal (95% CI)**		**40**		**39**		**Not estimable**
Total events	0		0			
Heterogeneity: Not applicable						
Test for overall effect: Not applicable						
Test for subgroup differences: Not applicable						

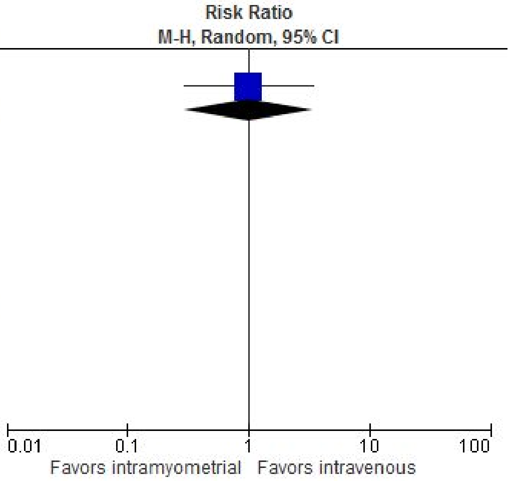

* One systolic blood pressure readings > 20% below measure at the time of uterine incision

** Akinaga 2016: two consecutive systolic blood pressure readings > 20% below measure at the time of uterine incision.

** Dennehy 1998: > 25% systolic blood pressure decrease below baseline measure (prior to uterine incision) on two subsequent one minute interval measurements

.

For peer review only - http://bmjopen.bmj.com/site/about/guidelines.xhtml

D. Forest plot of comparison: Intramyometrial versus Intravenous oxytocin, Outcome: Nausea and/or Vomiting.

	Intramyometrial oxytocin		Intravenous oxytocin			Risk Ratio
Study or Subgroup	Events	Total	Events	Total	Weight	M-H, Random, 95% CI
Akinaga 2016	0	20	1	20	29.2%	0.33 [0.01, 7.72]
Mangla 2012	1	50	10	50	70.8%	0.10 [0.01, 0.75]
**Total (95% CI)**		**70**		**70**	**100.0%**	**0.14 [0.03, 0.78]**
Total events	1		11			
Heterogeneity: Tau² = 0.00; Chi² = 0.41, df = 1 (P = 0.52); I² = 0%						
Test for overall effect: Z = 2.25 (P = 0.02)						

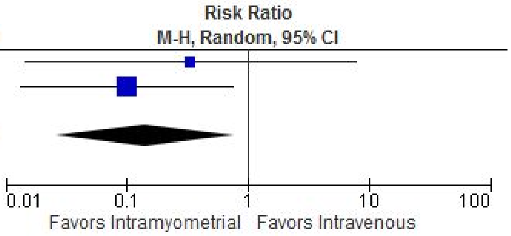

Akinaga 2016: nausea identified by questioning women immediately after oxytocin injection, every 2 minutes for 20 minutes.
Mangla 2012: use of additional antiemetic due to nausea and vomiting after initial antiemetic dose given to all women by anesthetist before induction.

E. Forest plot of comparison: Intramyometrial versus Intravenous oxytocin, Outcome: Headache.

	Intramyometrial oxytocin		Intravenous oxytocin			Risk Ratio
Study or Subgroup	Events	Total	Events	Total	Weight	M-H, Random, 95% CI
Akinaga 2016	1	20	0	20	100.0%	3.00 [0.13, 69.52]
**Total (95% CI)**		**20**		**20**	**100.0%**	**3.00 [0.13, 69.52]**
Total events	1		0			
Heterogeneity: Not applicable						
Test for overall effect: Z = 0.69 (P = 0.49)						

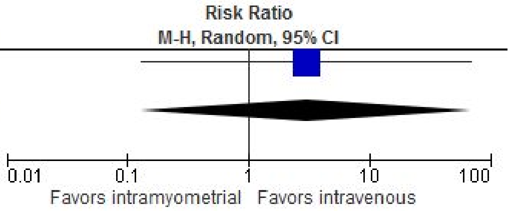

F. Forest plot of comparison: Intramyometrial versus Intravenous oxytocin, Outcome: Facial flushing.

	Intramyometrial oxytocin		Intravenous oxytocin			Risk Ratio
Study or Subgroup	Events	Total	Events	Total	Weight	M-H, Random, 95% CI
Akinaga 2016	1	20	2	20	100.0%	0.50 [0.05, 5.08]
**Total (95% CI)**		**20**		**20**	**100.0%**	**0.50 [0.05, 5.08]**
Total events	1		2			
Heterogeneity: Not applicable						
Test for overall effect: Z = 0.59 (P = 0.56)						

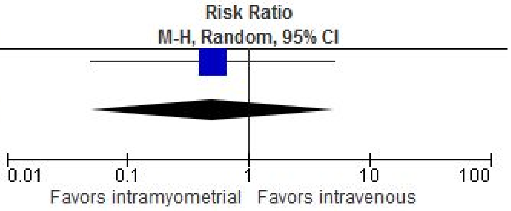

G. Forest plot of comparison: Intramyometrial versus Intravenous oxytocin, Outcome: Volume of blood loss.

	Intramyometrial oxytocin			Intravenous oxytocin				Mean Difference
Study or Subgroup	Mean	SD	Total	Mean	SD	Total	Weight	IV, Random, 95% CI
Akinaga 2016	606.8	68.8	20	664.2	74.1	20	100.0%	-57.40 [-101.71, -13.09]
**Total (95% CI)**			**20**			**20**	**100.0%**	**-57.40 [-101.71, -13.09]**
Heterogeneity: Not applicable								
Test for overall effect: Z = 2.54 (P = 0.01)								

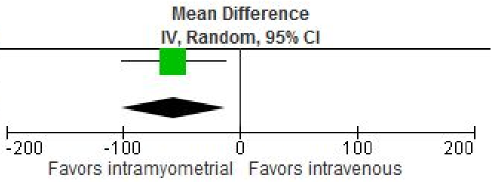

PRISMA 2009 Checklist

Section/topic	#	Checklist item	Reported on page #
**TITLE**			
Title	1	Identify the report as a systematic review, meta-analysis, or both.	1
**ABSTRACT**			
Structured summary	2	Provide a structured summary including, as applicable: background; objectives; data sources; study eligibility criteria, participants, and interventions; study appraisal and synthesis methods; results; limitations; conclusions and implications of key findings; systematic review registration number.	2
**INTRODUCTION**			
Rationale	3	Describe the rationale for the review in the context of what is already known.	3-4
Objectives	4	Provide an explicit statement of questions being addressed with reference to participants, interventions, comparisons, outcomes, and study design (PICOS).	4
**METHODS**			
Protocol and registration	5	Indicate if a review protocol exists, if and where it can be accessed (e.g., Web address), and, if available, provide registration information including registration number.	4, File S1
Eligibility criteria	6	Specify study characteristics (e.g., PICOS, length of follow-up) and report characteristics (e.g., years considered, language, publication status) used as criteria for eligibility, giving rationale.	4-5
Information sources	7	Describe all information sources (e.g., databases with dates of coverage, contact with study authors to identify additional studies) in the search and date last searched.	5
Search	8	Present full electronic search strategy for at least one database, including any limits used, such that it could be repeated.	File S2
Study selection	9	State the process for selecting studies (i.e., screening, eligibility, included in systematic review, and, if applicable, included in the meta-analysis).	5-6, Fig 1
Data collection process	10	Describe method of data extraction from reports (e.g., piloted forms, independently, in duplicate) and any processes for obtaining and confirming data from investigators.	5
Data items	11	List and define all variables for which data were sought (e.g., PICOS, funding sources) and any assumptions and simplifications made.	6, File S5
Risk of bias in individual studies	12	Describe methods used for assessing risk of bias of individual studies (including specification of whether this was done at the study or outcome level), and how this information is to be used in any data synthesis.	5-6
Summary measures	13	State the principal summary measures (e.g., risk ratio, difference in means).	6
Synthesis of results	14	Describe the methods of handling data and combining results of studies, if done, including measures of consistency (e.g., I^2) for each meta-analysis.	6

For peer review only - http://bmjopen.bmj.com/site/about/guidelines.xhtml

PRISMA 2009 Checklist

Section/topic	#	Checklist item	Reported on page #
Risk of bias across studies	15	Specify any assessment of risk of bias that may affect the cumulative evidence (e.g., publication bias, selective reporting within studies).	6
Additional analyses	16	Describe methods of additional analyses (e.g., sensitivity or subgroup analyses, meta-regression), if done, indicating which were pre-specified.	6
**RESULTS**			
Study selection	17	Give numbers of studies screened, assessed for eligibility, and included in the review, with reasons for exclusions at each stage, ideally with a flow diagram.	6-7 Fig 1, File S3
Study characteristics	18	For each study, present characteristics for which data were extracted (e.g., study size, PICOS, follow-up period) and provide the citations.	6-8, Table 1, File S4
Risk of bias within studies	19	Present data on risk of bias of each study and, if available, any outcome level assessment (see item 12).	9, Fig 2, File S6
Results of individual studies	20	For all outcomes considered (benefits or harms), present, for each study: (a) simple summary data for each intervention group (b) effect estimates and confidence intervals, ideally with a forest plot.	9, File S8
Synthesis of results	21	Present results of each meta-analysis done, including confidence intervals and measures of consistency.	9-10, Table 2,, Figs 3,4. File S8
Risk of bias across studies	22	Present results of any assessment of risk of bias across studies (see Item 15).	File S6
Additional analysis	23	Give results of additional analyses, if done (e.g., sensitivity or subgroup analyses, meta-regression [see Item 16]).	Not applicable
**DISCUSSION**			
Summary of evidence	24	Summarize the main findings including the strength of evidence for each main outcome; consider their relevance to key groups (e.g., healthcare providers, users, and policy makers).	10-11
Limitations	25	Discuss limitations at study and outcome level (e.g., risk of bias), and at review-level (e.g., incomplete retrieval of identified research, reporting bias).	11
Conclusions	26	Provide a general interpretation of the results in the context of other evidence, and implications for future research.	12
**FUNDING**			
Funding	27	Describe sources of funding for the systematic review and other support (e.g., supply of data); role of funders for the systematic review.	12

From: Moher D, Liberati A, Tetzlaff J, Altman DG, The PRISMA Group (2009). Preferred Reporting Items for Systematic Reviews and Meta-Analyses: The PRISMA Statement. PLoS Med 6(7): e1000097. doi:10.1371/journal.pmed1000097

For peer review only - http://bmjopen.bmj.com/site/about/guidelines.xhtml

BMJ Open

Route of oxytocin administration to prevent blood loss at caesarean section: a systematic review with meta-analysis

Journal:	*BMJ Open*
Manuscript ID	bmjopen-2021-051793.R1
Article Type:	Original research
Date Submitted by the Author:	21-Jun-2021

Complete List of Authors:	Torloni, Maria Regina; Hospital e Maternidade Santa Joana, ; Universidade Federal de Sao Paulo Escola Paulista de Medicina, Evidence-Based Healthcare Post-Graduate Program<br>Siaulys, Monica; Hospital e Maternidade Santa Joana, Anaesthesiology Department<br>Riera, Rachel; Hospital Sirio-Libanes, Centre of Health Technology Assessment; Universidade Federal de Sao Paulo Escola Paulista de Medicina, Evidence Based Healthcare Post-Graduation Program<br>Martimbianco, Ana ; Universidade Metropolitana de Santos; Universidade Federal de Sao Paulo Escola Paulista de Medicina, Evidence Based Healthcare Post-Graduate program<br>Pacheco, Rafael; Centro Universitário São Camilo; Universidade Federal de Sao Paulo Escola Paulista de Medicina, Evidence Based Healthcare Post-Graduation Program<br>Latorraca, Carolina ; Universidade Federal de Sao Paulo Escola Paulista de Medicina, Evidence Based Healthcare Post-Graduate program<br>Widmer, Mariana; World Health Organization, Reproductive Health and Research<br>Betran, Ana Pilar; World Health Organization, Reproductive Health and Research

**Primary Subject Heading**:	Obstetrics and gynaecology
Secondary Subject Heading:	Pharmacology and therapeutics
Keywords:	Maternal medicine < OBSTETRICS, THERAPEUTICS, Adverse events < THERAPEUTICS, Adult surgery < SURGERY

SCHOLARONE™
Manuscripts

I, the Submitting Author has the right to grant and does grant on behalf of all authors of the Work (as defined in the below author licence), an exclusive licence and/or a non-exclusive licence for contributions from authors who are: i) UK Crown employees; ii) where BMJ has agreed a CC-BY licence shall apply, and/or iii) in accordance with the terms applicable for US Federal Government officers or employees acting as part of their official duties; on a worldwide, perpetual, irrevocable, royalty-free basis to BMJ Publishing Group Ltd ("BMJ") its licensees and where the relevant Journal is co-owned by BMJ to the co-owners of the Journal, to publish the Work in this journal and any other BMJ products and to exploit all rights, as set out in our licence.

The Submitting Author accepts and understands that any supply made under these terms is made by BMJ to the Submitting Author unless you are acting as an employee on behalf of your employer or a postgraduate student of an affiliated institution which is paying any applicable article publishing charge ("APC") for Open Access articles. Where the Submitting Author wishes to make the Work available on an Open Access basis (and intends to pay the relevant APC), the terms of reuse of such Open Access shall be governed by a Creative Commons licence – details of these licences and which Creative Commons licence will apply to this Work are set out in our licence referred to above.

Other than as permitted in any relevant BMJ Author's Self Archiving Policies, I confirm this Work has not been accepted for publication elsewhere, is not being considered for publication elsewhere and does not duplicate material already published. I confirm all authors consent to publication of this Work and authorise the granting of this licence.

For peer review only

Route of oxytocin administration to prevent blood loss at caesarean section: a systematic review with meta-analysis

Maria Regina Torloni[1,2], Monica Siaulys[1], Rachel Riera[2,3], Ana Luiza Cabrera Martimbianco[2,4], Rafael Leite Pacheco[2,5], Carolina de Oliveira Cruz Latorraca[2], Mariana Widmer[6], Ana Pilar Betrán[6].

1. Hospital e Maternidade Santa Joana
Rua Dr. Eduardo Amaro 225, São Paulo, SP, CEP 04104-080, Brazil.

2. Evidence Based Healthcare Post-Graduation Program, São Paulo Federal University-UNIFESP, Rua Botucatu 740, 5º andar, São Paulo, SP, CEP 04023-900, Brazil

3. Centre of Health Technology Assessment, Hospital Sirio-Libanês,
Rua Dona Adma Jafet, 91, São Paulo, SP, CEP 01308-050, Brazil

4. Universidade Metropolitana de Santos (UNIMES)
Av. Gen. Francisco Glicério 8, Santos, SP, CEP 11045-002, Brazil

5. Centro Universitário São Camilo
Av. Nazaré, 1501, São Paulo, SP, CEP 04263-200, Brazil

6. Department of Reproductive Health and Research, World Health Organization, UNDP/UNFPA/UNICEF/WHO/World Bank Special Programme of Research, Development and Research Training in Human Reproduction (HRP),
Avenue Appia 20, 1201 Geneva, Switzerland.

Corresponding author:
Maria Regina Torloni, email: torlonimr@gmail.com

Word count: 4066

For peer review only

Abstract

Objectives: Assess the effects of different routes of prophylactic oxytocin administration to prevent blood loss at caesarean section (CS). **Design:** Systematic review and meta-analysis. **Methods.** Medline, EMBASE, CINAHL, Cochrane Library, BVS, SciELO, and Global Index Medicus were searched through 24/05/2020 for randomised controlled trials (RCT) comparing different routes of prophylactic oxytocin administration during CS. Study selection, data extraction, and quality assessment were conducted by two investigators independently. We pooled results in fixed effects metanalyses and calculated average risk ratio (RR), mean difference (MD), and 95% confidence intervals (CI). We used GRADE to assess the overall quality of evidence for each outcome. **Results:** Three trials (180 women) were included in the review. All studies compared intramyometrial (IMY) versus intravenous (IV) oxytocin in women having pre-labour CS. IMY compared to IV oxytocin administration may result in little or no difference in the incidence of post-partum haemorrhage (RR 0.14, 95% CI 0.01 to 2.70; N=100 participants; 1 RCT), hypotension (RR 1.00, 95% CI 0.29 to 3.45; N=40; 1 RCT), headache (RR 3.00, 95% CI 0.13 to 69.52; N=40; 1 RCT) or facial flushing (RR 0.50, 95% CI 0.05 to 5.08; N=40; 1 RCT); IMY oxytocin may reduce nausea/vomiting (RR 0.13, 95% CI 0.02 to 0.69; N=140; 2 RCTs). We are very uncertain about the effect IMY versus IV oxytocin on the need for additional uterotonics (RR 0.82; 95% CI 0.25 to 2.69; N=140; 2 RCTs). IMY oxytocin may reduce blood loss slightly (MD -57.40 mL, 95% CI -101.71 to -13.09; N=40; 1 RCT). **Conclusions:** There is limited, low to very low certainty evidence on the effects of two routes of prophylactic oxytocin administration at CS. At present, there is insufficient evidence to recommend IMY or IV oxytocin administration at CS to prevent blood loss. More trials are needed on this relevant question.

Prospero: CRD42020186797

Keywords: Postpartum Hemorrhage, Cesarean section, Oxytocin, Drug Administration Routes, Systematic Review, Meta-Analysis

For peer review only

Article summary: Strengths and Limitations

- This is the first systematic review to assess the effects of different routes of oxytocin administration to prevent bleeding in women giving birth by caesarean.
- We performed an extensive search without language restrictions in 10 databases to identify relevant trials, and followed strict methodology throughout the systematic review process.
- We found few trials, involving a small number of participants, and addressing a limited number of outcomes.
- The trials did not test all possible routes of oxytocin administration, and only included pre-labour caesareans at term.
- The overall certainty of the evidence was low or very low for all outcomes due mainly to imprecision.

For peer review only

INTRODUCTION

Deliveries by caesarean section (CS) have increased worldwide over the last three decades, including in low- and middle-income countries (LMIC)[1]. Even in optimal conditions, women who have a CS lose more blood, and have a higher risk for post-partum haemorrhage (PPH) than those who give birth by vaginal delivery (VD)[2 3]. PPH is an important cause of maternal morbidity, including prolonged hospital stay, blood transfusion and hysterectomy, and it is the leading cause of maternal mortality worldwide[4-6]. Uterine atony is responsible for 50% to 80% of all cases of PPH[4 6].

Routine administration of prophylactic uterotonics soon after birth reduces the incidence of PPH and its associated complications[6]. Oxytocin remains the first-line agent to prevent PPH recommended by the World Health Organization (WHO) because of its lack of major contraindications, similar efficacy, and lower cost and incidence of side effects, compared to other uterotonics[6]. For women having a VD, a systematic review in 2020 showed that intravenous (IV) oxytocin is more effective than intramuscular (IM) administration to reduce PPH, severe PPH, blood transfusion, and severe maternal morbidity, with no clear differences in adverse events[7]. Based on these findings, WHO updated its guideline in 2020 and now recommends that women giving birth by VD who already have endovenous access should receive a slow IV injection of 10 IU of oxytocin, in preference to IM administration[8]. For women giving birth by CS, due to the lack of specific evidence, WHO currently recommends 10 IU of oxytocin without preference for the IV or IM routes[8]. Other guidelines provide little, unclear, or no recommendations on the route of administration of prophylactic oxytocin at CS[9-11].

Since all women having a CS will have an endovenous access, IV administration of oxytocin seems logical. However, due to concerns about the cardiovascular side-effects of the drug in women undergoing a major abdominal surgery, many studies have investigated different dosages and regimens of IV oxytocin administration at CS in search of the optimal method to safely prescribe this uterotonic[12-17]. After IV injection in late pregnancy, oxytocin has an almost immediate onset of action but a half-life of only 5-12 minutes due to its rapid hepatic and renal degradation[17-19]. With continued infusion, oxytocin reaches a steady state plateau in the plasma in approximately 40 minutes[17-19]. Activation of oxytocin receptors in the myometrium stimulates prostaglandin synthesis and increases intracellular calcium in myocytes which leads to strong uterine contractions thus reducing blood loss from the placental site[16-19]. However, the simultaneous activation of oxytocin receptors in the cardiovascular system causes decreased systemic vascular resistance, hypotension, tachycardia, and coronary vasoconstriction with the attendant symptoms of skin flushing, headache, nausea, vomiting, and chest pain[17 20 21]. These changes are usually transient, depend on the dose and speed of IV oxytocin

For peer review only

administration, and are tolerated by most healthy young women[16 22]. However, in some women undergoing a CS, these haemodynamic changes can be potentially dangerous and even life-threatening because of the sympathetic nervous blockade secondary to regional anaesthesia, the concomitant use of other vasoactive drugs, excessive blood loss, and the underlying clinical and obstetric disorders that may have led to the CS indication (e.g. cardiac disease, hypertensive disorders, placenta previa)[21 23-25]. Due to its antidiuretic properties, oxytocin administration can also lead to water intoxication resulting in pulmonary oedema, seizures, coma and even death, especially in women who receive large volumes of fluid[13 17-19]. The routine use of large IV fluid preloads during CS under regional anaesthesia can potentially increase the risk of this complication[26].

After IM oxytocin injection, uterine response occurs within 3-5 minutes and can last for up to 2-3 hours[8 18 19 27]. Direct intramyometrial (IMY) injection of uterotonics can also promote uterine contraction[28 29]. IMY oxytocin injection is believed to promote immediate uterine contraction by local effect and by drug absorption from the myometrium into the systemic circulation[28 30-32]. Although oxytocin is not licensed for IMY use[18 19], this route of administration has been tested to prevent PPH at CS since 1990[28 30-32].

The route of administration of prophylactic oxytocin at CS can potentially affect the volume of maternal blood loss as well as the incidence of drug-related adverse effects. Since the side-effects of oxytocin are directly related to dose and rate of administration[13 17-19 27], in theory, the IM or IMY routes could lead to fewer cardiovascular side-effects than the IV route. If the efficacy of IM and IMY prophylactic oxytocin during CS is similar to IV administration, and if these routes have fewer side-effects than the endovenous route, this could challenge the preference for IV administration. Several randomised trials have compared different routes of prophylactic oxytocin administration at CS, but there are no systematic reviews on this topic. It is important to assess the balance between the effectiveness and adverse effects of the various routes of prophylactic oxytocin administration at CS because this information is essential to underpin evidence-based recommendations on the prevention of PPH in women given birth by CS.

OBJECTIVES

To assess the effects of different routes of prophylactic oxytocin administration to prevent blood loss at CS.

METHODS

For peer review only

We conducted this review following Cochrane methods[33] and reported it according to the PRISMA statement[34]. We registered the protocol prospectively (CRD42020186797- Supplementary file 1).

Patient and public involvement

There was no patient or public involvement in this study.

Types of studies

We included randomised clinical trials with a parallel design. Abstracts were eligible if they provided sufficient outcome data and methodological details for quality assessment.

Types of participants

Study participants were women having a primary or repeat CS, before or during labour, at any gestational age and for any indication, regardless of previous exposure to oxytocin (for labour induction or augmentation). Studies with women at high or low baseline risk for PPH, with or without comorbidities, and with singleton or multiple pregnancies were eligible for inclusion. We included studies involving women with both routes of delivery only if authors provided separate data for those giving birth by CS.

Types of interventions

We included studies that compared different routes of oxytocin administration (IV, IM or IMY) given alone, at any moment during CS (pre-delivery, after delivery, or after placental extraction). We excluded studies that administered oxytocin associated with other drugs to prevent PPH.

Outcomes

Studies that reported at least one of our outcomes of interest were included. Our outcomes were based on the PPH prevention core outcome set created by the CROWN initiative[35]. The primary outcomes were PPH $\geq$ 1000 mL (measured objectively or subjectively), use of additional uterotonics, and any immediate adverse effects possibly related to oxytocin (e.g. nausea/vomiting, facial flushing, headache, hypotension, cardiac arrhythmia). The secondary outcomes were total volume of blood loss at CS, need for transfusion, maternal transfer to higher level of care, shock, severe maternal morbidity (admission to ICU, hysterectomy, coma or organ failure), maternal mortality related to PPH, breastfeeding at hospital discharge, and maternal satisfaction.

Search strategy

For peer review only

We created a search strategy with the following general terms and synonyms: “caesarean section” or “C-section” or “abdominal delivery” and “oxytocin” (details in Supplementary file 2). The search strategy was developed by an experienced information specialist. It was adapted and run in seven electronic databases (MEDLINE, Embase, Cochrane Library, CINAHL, BVS, Global Index Medicus, SciELO) and two trial registry platforms (ClinicalTrials.gov and WHO – ICTRP) (Supplementary file 2). We searched for unpublished studies (grey literature) in Opengrey (https://opengrey.eu). We screened the reference lists of all retrieved studies and relevant systematic reviews to identify additional potentially eligible trials not captured by the electronic searches. There were no language or publication status restrictions. Searches were run from database inception until May 24, 2020.

Process of study selection, data extraction and risk of bias assessment

All references were uploaded in the Rayyan platform[36] and duplicates were excluded. All titles and abstracts were screened independently by two review authors. Potentially eligible studies were selected for full text reading; those that fulfilled the selection criteria were included in the review. Two review authors extracted data independently and a third author check data for accuracy. Two review authors independently assessed the quality of included studies using the Cochrane Risk of Bias tool[33]. In each study, authors assessed and graded seven domains as being at high, low, or unclear risk of bias. Four domains (random sequence generation, allocation concealment, selective reporting, and other source of bias) were assessed at study level; three domains (blinding of participants and personnel, blinding of outcome assessment, and incomplete outcome data) were assessed at outcome-level. Any disagreements in the process of study selection, data extraction, or risk of bias assessment were solved by a third senior review author.

Data analyses and assessment of the certainty of the evidence

We planned to conduct three comparisons between routes of oxytocin administration: IV vs IM, IV vs IMY, and IM vs IMY. All analyses were conducted on an intention-to-treat basis. When data were missing, we contacted trial authors. Where metanalyses were not deemed suitable, we report results descriptively. Similar study data were pooled using the software Review Manager 5.4 (The Cochrane Collaboration, 2020). For dichotomous outcomes, we calculated risk ratio (RR) and 95% confidence intervals (CI); for continuous data, we calculated mean differences (MD), or standardized mean differences (SMD), as appropriate, and 95% CI. We pooled data using the Mantel-Haenszel fixed-effect method; $I^2 \geq 50\%$ was considered an indication of high statistical heterogeneity. We planned to conduct pre-specified subgroup analyses for the primary outcomes (when data were available) according to parity (nullipara vs multipara, and multipara with CS vs

For peer review only - http://bmjopen.bmj.com/site/about/guidelines.xhtml

multipara without previous CS), type of CS (pre-labour vs intrapartum), previous use of oxytocin in labour, and baseline risk for PPH. We could not conduct these analyses because of the small number of trials included and lack of information. We planned to conduct sensitivity analyses for the main comparisons restricted only to high-quality studies (at low risk of bias for random sequence generation and allocation concealment), and restricted only to studies that assessed blood loss using an objective measure. These analyses were not done because all studies measured blood loss objectively and none were high-quality. Due to the small number of trials, we could not assess publication bias.

We assessed the certainty (quality) of the body of evidence for selected outcomes using the GRADE approach[37]. Evidence was categorized as being of high, moderate, low, or very low quality. We downgraded the quality of the evidence one or two levels because of trial limitations, inconsistency, indirectness, imprecision, and publication bias[37]. This process was conducted by two independent investigators; disagreements were solved by a third senior investigator.

RESULTS

A total of 13,389 unique references were identified. After title and abstract screening, six citations were selected for full text reading. Two studies and one abstract were excluded (Supplementary file 3) and three trials[30-32] were included in the review (Figure 1).

The three trials included a total of 180 women and were conducted between 1998 and 2012, in Japan[30], Canada[31] , and India[32] (Table 1 and Supplementary file 4). None of the trials provided clear information on the proportion of nulliparas or multiparas (with and without previous CS). One trial[32] did not report participants´ baseline risk for PPH, gestational age, or type of CS; the other two trials[30 31] included participants with regular/low baseline risk for PPH, undergoing a pre-labour CS with at least 36 weeks´ gestation, under spinal anaesthesia (Table 1). All three trials compared IV vs IMY oxytocin administration after foetal delivery. There was heterogeneity between studies on the oxytocin doses used in each route; the IMY dose ranged from 4.3 IU[30] to 20 IU[31], and the total IV dose ranged from 4.3 IU[30] to 20 IU[32]. One trial[30] administered oxytocin with the placenta still in place, immediately after umbilical cord clamping, while the other two studies waited for placental detachment or removal before giving the oxytocin[31 32]. Akinaga et al randomised 40 Japanese women to receive either an IMY oxytocin injection and an IV saline bolus injection, or an IV oxytocin bolus and an IMY saline injection immediately after umbilical cord clamping; all women also received a slow oxytocin maintenance

For peer review only

infusion for the next five hours[30]. Dennehy et al randomised 40 women in Canada to receive an IMY oxytocin bolus injection into the uterine fundus and a saline IV bolus injection, or an IV oxytocin bolus injection and a saline IMY injection after placental removal[31]. Mangla et al randomised 150 women in India to start a 500 mL infusion containing oxytocin after placental separation (Group 1, N=50), or to receive an IMY oxytocin bolus injection (half in each uterine cornus) either after placental separation (Group 2, N=50) or before placental separation (Group 3, N=50) [32]. We did not include in this systematic review the participants in the third group because it compared a different route and a different timing of oxytocin administration (Supplementary file 4). The two trials that reported the volume of blood loss [30 32] used objective methods of assessment, but only one[32] assessed the incidence of blood loss > 900 mL. All trials assessed the need for additional uterotonics and at least one adverse effect of oxytocin. No trial reported any of our other secondary outcomes (Table 1 and Supplementary file 5).

For peer review only

Table 1. Main characteristics of trials on routes of oxytocin administration at caesarean section

Characteristics	Akinaga 2016 (ref 30)	Dennehy 1998 (ref 31)	Mangla 2012 (ref 32)
Setting	Japan, one university hospital	Canada, no information on type of hospital(s)	India, one hospital
Design	Double blind, placebo controlled RCT	Double blind, placebo controlled RCT	RCT
Sample size	40	40	100[1]
Gestational age	all $\geq$ 36 weeks	all $\geq$ 37 weeks	no information
Baseline risk for PPH	all low risk	all low risk	no information
Parity	unclear	no information	no information
Participants with previous CS	unclear	no information	no information
Type of CS	Pre-labour, scheduled	Pre-labour, scheduled	no information
Previous exposure to oxytocin	No	No	no information
Anaesthesia	spinal	spinal	spinal or general
Timing of administration	After umbilical cord clamping	After placental removal	After placental separation
Oxytocin routes and regimen	IMY bolus vs IV bolus followed by IV maintenance infusion	IMY bolus vs IV bolus	IMY bolus vs IV infusion
Oxytocin dose, diluent, speed of administration, (dose/min), Total dose	IMY bolus: 4.3 IU[2] in 2 mL 0.9% saline over 30 sec in uterine fundus.<br>Total dose: 4.3 IU<br><br>IV bolus: 4.3 IU[2] in 10 ml 0.9% saline over 30 sec (8.5 IU/min)<br>Total dose: 4.3 IU<br><br>Maintenance infusion (both groups): 6.1 IU[3] in 500 ml Ringer at 100 ml/h for 5 h (0.02 IU/min)<br>Total dose: 6.1 IU<br><br>Total oxytocin regimen dose[4]: 10.4 IU	IMY bolus: 2 ml of 10 IU/ml in uterine fundus.<br>Total dose: 20 IU<br><br>IV bolus: 0.5 ml of 10 IU/ml over unknown time<br>Total dose: 5 IU	IMY bolus: 5 IU in 10 ml 0.9% saline (5 ml in each uterine cornu).<br>Total dose: 5 IU<br><br>IV infusion 20 IU in 500 ml Ringer, no information on speed or duration<br>Total dose: 20 IU
Outcomes reported	Volume of blood loss, Additional uterotonics, Adverse effects	Additional uterotonics, Adverse effects	Volume of blood loss, Additional uterotonics, Adverse effects

CS: caesarean section, IU: international units, IV: intravenous, IMY: Intramyometrial, min: minutes, ml: millilitre, PPH: post-partum haemorrhage, RCT: randomised controlled trial, Ref: reference, Sec: seconds.
1. Mangla 2012 had a third group with 50 women (total sample: 150) that was not included in this review because it administered IMY before placental separation (i.e. different timing and route from IV group).
2. Bolus dose was calculated based on the mean weight of participants (0.07 IU/Kg x 61 Kg)

For peer review only

3. Maintenance infusion dose was calculated based on the mean weight of participants (0.01 IU/Kg x 61 Kg).
4. Total oxytocin dose received by all participants (bolus + maintenance infusion)

Figure 2 presents the risk of bias of the three included studies (see Supplementary file 6 for details and summary graph). All studies had at least one domain with unclear or high risk of bias. Akinaga 2016[30] and Mangla 2012[32] did not describe the method used to generate the randomization sequence; Dennehy 1998[31] and Mangla 2012[32] did not describe how allocation concealment was achieved. Overall, the three trials had an unclear risk of bias for random sequence generation or allocation concealment, or both domains. We contacted the authors of the three trials to obtain additional details, but none replied.

Table 2 presents a summary of all outcomes for the comparison between prophylactic IMY versus IV oxytocin in women having a CS. See supplementary file 7 for the assessment of the certainty of the evidence (GRADE) for each outcome.

For peer review only

Table 2. Comparison of prophylactic intramyometrial versus intravenous oxytocin to prevent blood loss in women giving birth by caesarean

Outcome or subgroup title	N studies	N women	Effect measure (IMY vs IV)	Effect size (95% CI)	Certainty of the evidence (GRADE)[3]
1. PPH > 1000 mL	1	100	Risk Ratio	0.14 (0.01, 2.70)	Low
2. Use of additional uterotonics	2	140	Risk Ratio	0.82 (0.25, 2.69)[1]	Very low
3. Hypotension not requiring ephedrine	1	40	Risk Ratio	1.00 (0.29, 3.45)	Low
4. Hypotension requiring ephedrine	2	79	Risk Ratio	Not estimable	Low
5. Nausea and/or vomiting	2	140	Risk Ratio	0.13 (0.02, 0.69) [1]	Low
6. Headache	1	40	Risk Ratio	3.00 (0.13, 69.52)	Low
7. Facial flushing	1	40	Risk Ratio	0.50 (0.05, 5.08)	Low
8. Mean blood loss (mL)	1[2]	40	Mean Difference	-57.40 (-101.71, -13.09)	Low

CI: Confidence interval, IMY: Intramyometrial, N=number, PPH: Post-partum haemorrhage, vs: versus.
1. Mantel-Haenszel fixed-effect meta-analysis.
2. Two trials reported mean blood loss, but one (Mangla 2012) could not be included in effect size estimates because it did not provide standard deviation or CI.
3. See Supplementary file 7 for details.

For peer review only

Incidence of PPH

None of the studies assessed PPH according to the standard definition (≥ 1000 ml). However, Mangla 2012[32] reported that none of the women who received IMY oxytocin had a blood loss > 900 ml compared to 6% of those who received IV oxytocin (0/50 versus 3/50, RR 0.14, 95% CI 0.01 to 2.70; 100 participants; 1 RCT, low certainty evidence). (Table 2).

Need for additional uterotonics

The pooled estimate from the three trials was very imprecise and compatible with an important risk reduction or increase in the need for additional uterotonic with IMY compared to IV oxytocin injection (4/70 versus 5/70, respectively; RR 0.82; 95% CI 0.25 to 2.69; 140 participants; 2 RCTs; I^2=61%, very low certainty evidence) (Figure 3, Table 2)

Adverse effects of oxytocin

Hypotension: Data from one trial[30] indicates that IMY compared to IV oxytocin administration may have no effect on the incidence of isolated hypotension episodes that do not require ephedrine (RR: 1.00, 95% CI 0.29 to 3.45; 40 participants; 1 RCT; low certainty evidence). Two trials[30 31] (79 participants) assessed the incidence of persistent hypotension requiring treatment with epinephrine, but there were no events among the women who received IMY or IV oxytocin. Although the lack of events did not allow estimation of the risk ratio, the evidence from these trials is consistent with absence of a difference between IMY and IV for this outcome. (Table 2).

Changes in heart rate: We could not perform a meta-analysis for this outcome. Akinaga[30] reported no apparent changes in the heart rate (HR) of the 20 women who received IMY oxytocin while the HR of the IV group started to increase soon after the bolus injection, reaching a maximum value 75 seconds after. The maximum HR in the IV group was significantly higher than in the IMY group (92.9 ± 10.3 vs 83.2 ± 7.2 beats per minute, mean difference: 9.7, 95% CI 4.0 to 15.4, p=0.002). On the other hand, Dennehy reported a significantly higher increase in HR one minute after IMY than after IV oxytocin administration, but the authors did not provide numerical data for this outcome[31].It should be noted that while Akinaga[30] gave the same dose of oxytocin (4.3 IU) in both routes, the IMY oxytocin dose given by Dennehy[31] was four times higher than the IV dose (20 IU vs 5 IU, respectively).

Nausea and/or vomiting: The pooled estimate of two trials including 140 women showed a lower average risk of nausea and/or vomiting in the group that received

For peer review only - http://bmjopen.bmj.com/site/about/guidelines.xhtml

IMY oxytocin (RR 0.14; 95% CI 0.03 to 0.78; 140 participants; 2 RCTs; I^2=0%, low certainty evidence) (Figure 4, Table 2).

Headache and facial flushing: Only Akinaga[30] reported the incidence of headache and facial flushing**.** One woman in the IMY group complained of headache versus none in the IV group (1/20 versus 0/20, RR 3.00, 95% CI 0.13 to 69.52; 40 participants; 1 RCT; low certainty evidence). One participant in the IMY and two in the IV oxytocin group had facial flushing (1/20 versus 2/20, RR 0.50, 95% CI 0.05 to 5.08; 40 participants; 1 RCT; low certainty evidence). (Table 2).

Volume of blood loss

Mangla[32] reported that the women in the IMY group had a lower mean volume of blood loss than those in the IV group (460 mL versus 606 mL, respectively), but did not provide the standard deviations (SD) or confidence intervals for this measure. Akinaga[30] also reported a slightly lower mean volume of blood loss in the IMY than in the IV group (606.8 ± 68.8 versus 664.2 ± 74.1 mL; MD -57.40 mL; 95% CI -101.71 to -13.09; 40 participants; 1 RCT; low certainty evidence) (Table 2). We could not pool the results of the two trials because one of them[32] did not provide information on SD and the authors did not reply to our emails.

DISCUSSION

Main Findings and Interpretation

Our extensive search identified only three trials on the effects of different routes of administration of prophylactic oxytocin in women giving birth by CS. The existing evidence suggests that IMY compared to IV oxytocin administration may result in little to no difference on the incidence of PPH (≥ 1000 mL) and most side effects (hypotension, headache, or facial flushing), but IMY oxytocin may reduce the incidence of nausea/vomiting. The evidence also suggests that IMY oxytocin may reduce the volume of blood loss slightly. We are very uncertain about the effects of IMY versus IV oxytocin on the need for additional uterotonics. The overall certainty of the evidence was low or very low for all outcomes due mainly to imprecision. According to the GRADE approach, this means that we have limited confidence in the effect estimates and that the true effect may be substantially different from the estimates presented.[37]

Where information was available, most trials recruited only healthy women with a low baseline risk for PPH, who were having an elective pre-labour CS under spinal anaesthesia to deliver a single foetus at term. Information about parity, including the percentage of participants with one or more previous CS, was unclear or missing in

For peer review only - http://bmjopen.bmj.com/site/about/guidelines.xhtml

all studies. Therefore, we cannot infer that the results of this systematic review apply to women with other characteristics, such as preterm or multiple pregnancies, women having intra-partum CS, or those with previous exposure to oxytocin for labour induction or augmentation. The different doses and regimen of oxytocin used in the trials can also have affected the results because oxytocin plasma levels are dose dependent.[38 39]

It is surprising that we did not find any studies comparing IM versus IV prophylactic oxytocin at CS, since these are the more frequently used routes of administration, and there are many trials on this comparison for women giving birth vaginally[7]. While the slower onset of action of oxytocin after IM administration[18 19] can raise concerns about its capacity to control bleeding at CS, this could, in theory, be circumvented by injecting it earlier, for example at uterine incision instead of after foetal delivery. Potential advantages of IM compared to IV administration include fewer side effects due to the slower increase in oxytocin plasma concentration, and a longer lasting effect[18 19] which could preclude the need for IV oxytocin maintenance infusions. Intravenous oxytocin is a high-alert medication[40] (a drug with increased risk of causing significant patient harm when used in error) which was listed in third place in the list of the top medication errors in 2020[41]. Therefore, in theory, an additional safety advantage of the IM route is that it could potentially reduce medication errors associated with IV oxytocin. Finally, for women having a CS, prophylactic IM oxytocin injection would be painless because the patient would be under anaesthesia. It was also surprising to find three trials that assessed IMY oxytocin administration[30-32] because the drug is not licenced for IMY use, and there are no pharmacokinetic studies on this route of oxytocin administration. In order to license IMY oxytocin administration, the first step would be to conduct pharmacokinetic/pharmacodynamic (PK/PD) as well as safety studies to ensure that the drug administered in the new route is effective and safe. However, since PK/PD and safety studies are expensive and complex, it may be difficult to find institutions willing to conduct this type of study.[42]

Strengths and Limitations of the review

Previous reviews have compared various uterotonics (including oxytocin) versus placebo or other agents in different doses, regimens, and timing of administration to prevent PPH at CS [14 43]. However, to the best of our knowledge, this is the first systematic review to assess different routes of oxytocin administration to prevent PPH in women giving birth by CS. Strong points of our review include its comprehensive literature search without language restrictions and strict adherence to standard Cochrane methods[33]. Limitations of the review include the small number of trials identified, the lack of response from authors to clarify important

For peer review only

methodological and clinical aspects of their studies, and the fact that none of the trials addressed additional core clinical outcomes.

Implications for Practice and Research

At present, there is insufficient evidence to recommend IMY or IV oxytocin administration at CS to prevent PPH. Our review findings indicate the need for more high quality trials comparing the effectiveness and safety of different routes of prophylactic oxytocin administration, including the intramuscular route, in women having a CS. Future trials should have adequate sample size, be of high methodological and reporting quality, and recruit participants who represent the general obstetric population, including women at high risk for PPH due to clinical or obstetric disorders, and women undergoing prelabour as well as intrapartum CS, with or without previous exposure to oxytocin for labour induction or augmentation. Future studies should consider recent evidence about IV dosing regimens to reduce blood loss at CS[44]. These trials also need to report all relevant PPH prevention core outcomes [35]. The results of these future trials are essential for updates of our systematic review and meta-analyses, as well as for individual patient data metanalyses that will provide more robust evidence on this topic. Given the increasing numbers of births by CS worldwide including in LMIC, and the high rates of maternal mortality following CS in these countries[45], research to identify the most beneficial route and regimen for prophylactic oxytocin to prevent PPH in women giving birth by CS should be prioritized to avoid preventable mortality and morbidity. Particularly disturbing is the lack of evidence for women having intrapartum CS. These women are particularly vulnerable to uterine atony and increased post-partum bleeding because of prolonged labours, often with previous exposure to oxytocin for induction or augmentation which may desensitize oxytocin receptors and impair myometrial response to prophylactic oxytocin after delivery[46 47]. These evidence gaps are likely to affect more dramatically women in LMIC where skills and resources to provide safe and timely CS, and to deal with its complications, are sub-optimal. This lack of evidence is thus likely to increase maternal and perinatal inequalities between and within countries.

CONCLUSIONS

There is limited, low to very low certainty, evidence on the effects of different routes of prophylactic oxytocin administration at CS to prevent blood loss. The limited evidence suggests that IMY compared to IV oxytocin may result in little to no difference in the incidence of PPH and most side effects, but may reduce nausea/vomiting and the volume of blood loss slightly. More trials are needed on this relevant question, especially on the effects of intramuscular oxytocin and involving women with intrapartum CS.

For peer review only

Author Contributions

MRT, MS, APB and MW conceptualized the study. MRT wrote the study protocol with the contributions of MS, APB, MW and RR. The search strategy was developed by COCL, MRT and RR, with the contribution of MS and APB. Citation screening and study selection were performed by MS, APB, ALCM, RLP, and COCL under the supervision of RR and MRT. Data extraction and study quality assessment were performed by MRT, ALCM, RR and RLP. Data analysis was conducted by MRT, RR, ALCM and RLP. The report was drafted by MRT with input from MS, APB and MW. All authors reviewed and approved the final manuscript. MRT is the guarantor and takes responsibility for the content of this article

Acknowledgements: none

Funding This work was supported by UNDP/UNFPA/UNICEF/WHO/World Bank Special Programme of Research, Development and Research Training in Human Reproduction (HRP), Department of Sexual and Reproductive Health and Research, World Health Organization, Geneva, Switzerland.

Disclaimer: The funders had no role in conducting or writing the study

Competing interests: None declared.

Patient consent: Not required

Provenance and peer review: Not commissioned; externally peer reviewed.

Data sharing statement: All data relevant to the study are included in the article or uploaded as supplementary information.

Figure legends:

Figure 1. Flowchart of the process of study selection
Figure 2. Risk of bias summary of trials on oxytocin route of delivery at caesarean
Figure 3. Forest plot of intramyometrial versus intravenous oxytocin at caesarean. Outcome: need for additional uterotonic
Figure 4. Forest plot of intramyometrial versus intravenous oxytocin at caesarean. Outcome: nausea and/or vomiting

Ethics statement: This study does not involve human participants.

For peer review only

References

1. Betran AP, Ye J, Moller AB, et al. The Increasing Trend in Caesarean Section Rates: Global, Regional and National Estimates: 1990-2014. *PLoS One* 2016;11(2):e0148343. doi: 10.1371/journal.pone.0148343
2. Fawcus S, Moodley J. Postpartum haemorrhage associated with caesarean section and caesarean hysterectomy. *Best Pract Res Clin Obstet Gynaecol* 2013;27(2):233-49. doi: 10.1016/j.bpobgyn.2012.08.018
3. Parant O, Guerby P, Bayoumeu F. [Obstetric and anesthetic specificities in the management of a postpartum hemorrhage (PPH) associated with cesarean section]. *J Gynecol Obstet Biol Reprod (Paris)* 2014;43(10):1104-22. doi: 10.1016/j.jgyn.2014.10.004
4. Deneux-Tharaux C, Bonnet MP, Tort J. [Epidemiology of post-partum haemorrhage]. *J Gynecol Obstet Biol Reprod (Paris)* 2014;43(10):936-50. doi: 10.1016/j.jgyn.2014.09.023
5. Say L, Chou D, Gemmill A, et al. Global causes of maternal death: a WHO systematic analysis. *Lancet Glob Health* 2014;2(6):e323-33. doi: 10.1016/S2214-109X(14)70227-X
6. World Health Organization. WHO recommendations: uterotonics for the prevention of postpartum haemorrhage. Geneva: World Health Organization; 2018. Licence: CC BY-NC-SA 3.0 IGO.
7. Oladapo OT, Okusanya BO, Abalos E, et al. Intravenous versus intramuscular prophylactic oxytocin for the third stage of labour. *Cochrane Database Syst Rev* 2020;11:CD009332. doi: 10.1002/14651858.CD009332.pub4
8. World Health Organization. WHO recommendation on routes of oxytocin administration for the prevention of postpartum haemorrhage after vaginal birth. Geneva: World Health Organization; 2020. Licence: CC BY-NC-SA 3.0 IGO.
9. Dahlke JD, Mendez-Figueroa H, Maggio L, et al. Prevention and management of postpartum hemorrhage: a comparison of 4 national guidelines. *Am J Obstet Gynecol* 2015;213(1):76 e1-76 e10. doi: 10.1016/j.ajog.2015.02.023
10. Sentilhes L, Goffinet F, Vayssiere C, et al. Comparison of postpartum haemorrhage guidelines: discrepancies underline our lack of knowledge. *BJOG* 2017;124(5):718-22. doi: 10.1111/1471-0528.14305
11. Weale N, Laxton C. Prophylactic use of oxytocin at caesarean section: where are the guidelines? *Anaesthesia* 2013;68(10):1006-9. doi: 10.1111/anae.12337
12. Carvalho JC, Balki M, Kingdom J, et al. Oxytocin requirements at elective cesarean delivery: a dose-finding study. *Obstet Gynecol* 2004;104(5 Pt 1):1005-10. doi: 10.1097/01.AOG.0000142709.04450.bd
13. Dyer RA, van Dyk D, Dresner A. The use of uterotonic drugs during caesarean section. *Int J Obstet Anesth* 2010;19(3):313-9. doi: 10.1016/j.ijoa.2010.04.011
14. Stephens LC, Bruessel T. Systematic review of oxytocin dosing at caesarean section. *Anaesth Intensive Care* 2012;40(2):247-52. doi: 10.1177/0310057X1204000206
15. Roach MK, Abramovici A, Tita AT. Dose and duration of oxytocin to prevent postpartum hemorrhage: a review. *Am J Perinatol* 2013;30(7):523-8. doi: 10.1055/s-0032-1329184
16. Yamaguchi ET, Siaulys MM, Torres ML. Oxytocin in cesarean-sections. What's new? *Braz J Anesthesiol* 2016;66(4):402-7. doi: 10.1016/j.bjane.2014.11.015
17. Balki M, Tsen L. Oxytocin protocols for cesarean delivery. *Int Anesthesiol Clin* 2014;52(2):48-66. doi: 10.1097/AIA.0000000000000016
18. FDA Approved Drug Products: Pitocin (oxytocin) injection. Available at: https://www.accessdata.fda.gov/drugsatfda_docs/label/2014/018261s031lbl.pdf. Accessed on December 20, 2020.

For peer review only

19. Prescribers Digital Reference (PDR): Oxytocin - Drug Summary. Available at: https://www.pdr.net/drug-summary/Pitocin-oxytocin-1666. Accessed on December 20, 2020.
20. Pinder AJ, Dresner M, Calow C, et al. Haemodynamic changes caused by oxytocin during caesarean section under spinal anaesthesia. *Int J Obstet Anesth* 2002;11(3):156-9. doi: 10.1054/ijoa.2002.0970
21. Thomas JS, Koh SH, Cooper GM. Haemodynamic effects of oxytocin given as i.v. bolus or infusion on women undergoing Caesarean section. *Br J Anaesth* 2007;98(1):116-9. doi: 10.1093/bja/ael302
22. Tsen LC, Balki M. Oxytocin protocols during cesarean delivery: time to acknowledge the risk/benefit ratio? *Int J Obstet Anesth* 2010;19(3):243-5. doi: 10.1016/j.ijoa.2010.05.001
23. Jonsson M, Hanson U, Lidell C, et al. ST depression at caesarean section and the relation to oxytocin dose. A randomised controlled trial. *BJOG* 2010;117(1):76-83. doi: 10.1111/j.1471-0528.2009.02356.x
24. Svanstrom MC, Biber B, Hanes M, et al. Signs of myocardial ischaemia after injection of oxytocin: a randomized double-blind comparison of oxytocin and methylergometrine during Caesarean section. *Br J Anaesth* 2008;100(5):683-9. doi: 10.1093/bja/aen071
25. Thomas TA, Cooper GM, Editorial Board of the Confidential Enquiries into Maternal Deaths in the United K. Maternal deaths from anaesthesia. An extract from Why mothers die 1997-1999, the Confidential Enquiries into Maternal Deaths in the United Kingdom. *Br J Anaesth* 2002;89(3):499-508.
26. Sarna MC, Soni AK, Gomez M, et al. Intravenous oxytocin in patients undergoing elective cesarean section. *Anesth Analg* 1997;84(4):753-6. doi: 10.1097/00000539-199704000-00010
27. Unterscheider J, Breathnach F, Geary M. Standard medical therapy. In: Arulkumaran S, Karoshi M, Keith LG, et al., eds. A comprehensive textbook of postpartum hemorrhage An essential clinical reference for effective management. 2nd ed. London: Sapiens Publishing Ltd 2012:355-60.
28. Catanzarite VA. Prophylactic intramyometrial carboprost tromethamine does not substantially reduce blood loss relative to intramyometrial oxytocin at routine cesarean section. *Am J Perinatol* 1990;7(1):39-42. doi: 10.1055/s-2007-999443
29. Hayashi RH, Castillo MS, Noah ML. Management of severe postpartum hemorrhage due to uterine atony using an analogue of prostaglandin F2 alpha. *Obstet Gynecol* 1981;58(4):426-9.
30. Akinaga C, Uchizaki S, Kurita T, et al. Randomized double-blind comparison of the effects of intramyometrial and intravenous oxytocin during elective cesarean section. *J Obstet Gynaecol Res* 2016;42(4):404-9. doi: 10.1111/jog.12926
31. Dennehy KC, Rosaeg OP, Cicutti NJ, et al. Oxytocin injection after caesarean delivery: intravenous or intramyometrial? *Can J Anaesth* 1998;45(7):635-9. doi: 10.1007/BF03012092
32. Mangla D, Goel JK, Goel R. Prophylactic Intramyometrial Oxytocin before Placenta Delivery during Cesarean Section Prevents Postpartum Hemorrhage: A Prospective Randomized Study of 150 Women. *J South Asian Feder Obst Gynae* 2012;4(2):93-96.
33. Higgins JPT, Thomas J, Chandler J, et al. Cochrane Handbook for Systematic Reviews of Interventions version 6.1 [Internet]. 2020. Available at www.training.cochrane.org/handbook. In: Cochrane, ed.
34. Moher D, Liberati A, Tetzlaff J, et al. Preferred reporting items for systematic reviews and meta-analyses: the PRISMA statement. *J Clin Epidemiol* 2009;62(10):1006-12. doi: 10.1016/j.jclinepi.2009.06.005

For peer review only

35. Meher S, Cuthbert A, Kirkham JJ, et al. Core outcome sets for prevention and treatment of postpartum haemorrhage: an international Delphi consensus study. *BJOG* 2019;126(1):83-93. doi: 10.1111/1471-0528.15335
36. Ouzzani M, Hammady H, Fedorowicz Z, et al. Rayyan-a web and mobile app for systematic reviews. *Syst Rev* 2016;5(1):210. doi: 10.1186/s13643-016-0384-4
37. Guyatt GH, Oxman AD, Vist GE, et al. GRADE: an emerging consensus on rating quality of evidence and strength of recommendations. *BMJ* 2008;336(7650):924-6. doi: 10.1136/bmj.39489.470347.AD
38. Uvnas-Moberg K, Ekstrom-Bergstrom A, Berg M, et al. Maternal plasma levels of oxytocin during physiological childbirth - a systematic review with implications for uterine contractions and central actions of oxytocin. *BMC Pregnancy Childbirth* 2019;19(1):285. doi: 10.1186/s12884-019-2365-9
39. Yamaguchi ET, Cardoso MM, Torres ML, et al. Serum oxytocin concentrations in elective caesarean delivery: a randomized comparison of three infusion regimens. *Int J Obstet Anesth* 2011;20(3):224-8. doi: 10.1016/j.ijoa.2011.03.004
40. Institute for Safe Medication Practices-ISMP 2018. High-Alert Medications in Acute Care Settings. Available at: https://www.ismp.org/recommendations/high-alert-medications-acute-list
41. Institute for Safe Medication Practices-ISMP 2021. Start the Year Off Right by Preventing These Top 10 Medication Errors and Hazards from 2020. Available at: https://www.ismp.org/resources/start-year-right-preventing-these-top-10-medication-errors-and-hazards-2020.
42. U.S. Department of Health and Human Services, Food and Drug Administration, Center for Drug Evaluation and Research (CDER). Guidance for Industry: Changes to an Approved NDA or ANDA. April 2004. Available at: https://www.fda.gov/files/drugs/published/Changes-to-an-Approved-NDA-or-ANDA.pdf.
43. Gallos I, Williams H, Price M, et al. Uterotonic drugs to prevent postpartum haemorrhage: a network meta-analysis. *Health Technol Assess* 2019;23(9):1-356. doi: 10.3310/hta23090
44. Phung LC, Farrington EK, Connolly M, et al. Intravenous oxytocin dosing regimens for postpartum hemorrhage prevention at cesarean section: a systematic review and meta-analysis. *Am J Obstet Gynecol* 2021 doi: 10.1016/j.ajog.2021.04.258
45. Sobhy S, Arroyo-Manzano D, Murugesu N, et al. Maternal and perinatal mortality and complications associated with caesarean section in low-income and middle-income countries: a systematic review and meta-analysis. *Lancet* 2019;393(10184):1973-82. doi: 10.1016/S0140-6736(18)32386-9
46. Bischoff K, Nothacker M, Lehane C, et al. Lack of controlled studies investigating the risk of postpartum haemorrhage in cesarean delivery after prior use of oxytocin: a scoping review. *BMC Pregnancy Childbirth* 2017;17(1):399. doi: 10.1186/s12884-017-1584-1
47. Phaneuf S, Rodriguez Linares B, TambyRaja RL, et al. Loss of myometrial oxytocin receptors during oxytocin-induced and oxytocin-augmented labour. *J Reprod Fertil* 2000;120(1):91-7. doi: 10.1530/jrf.0.1200091

For peer review only

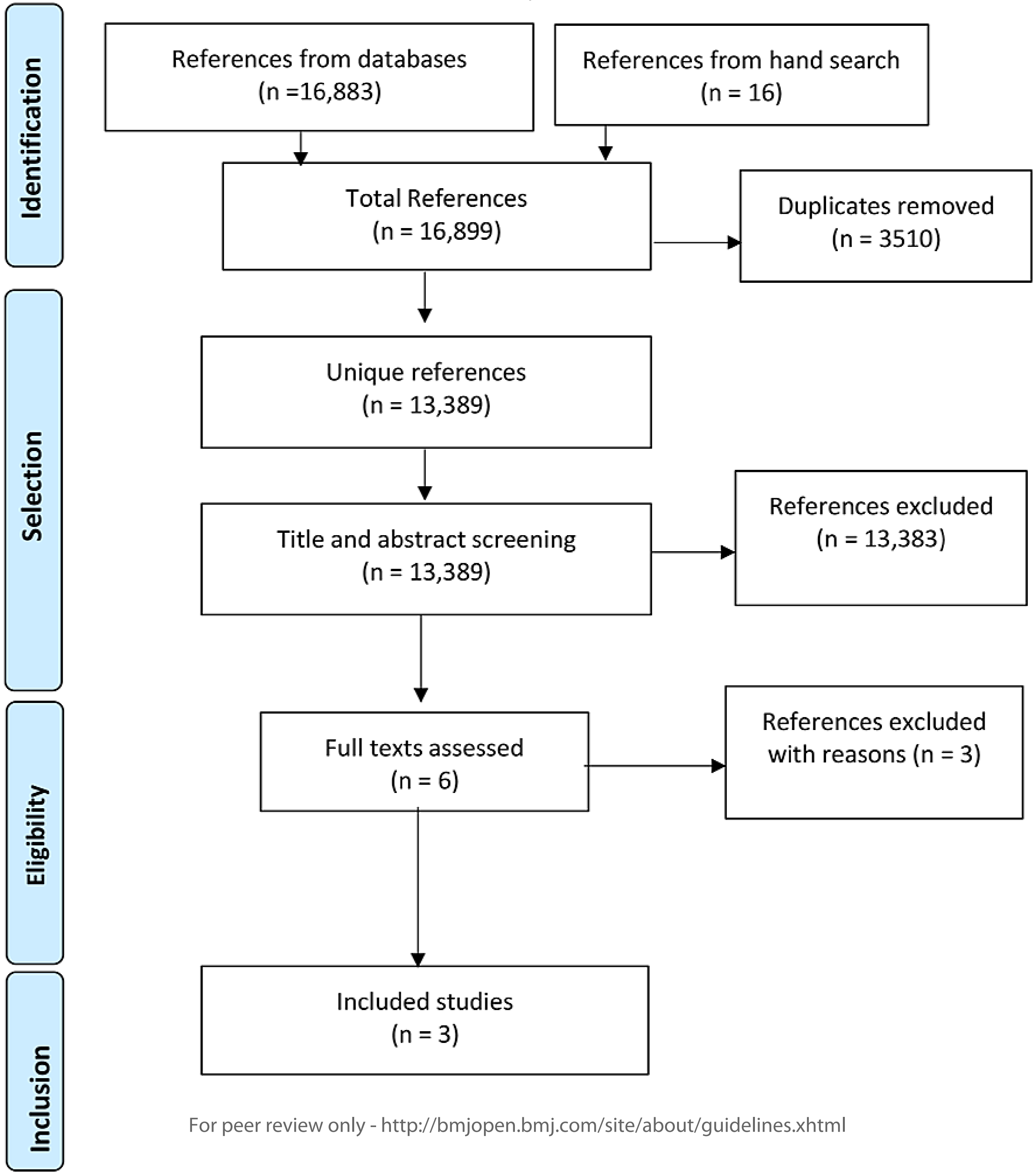
Identification
Selection
Eligibility
Inclusion
References from databases (n =16,883)
References from hand search (n = 16)
Total References (n = 16,899)
Duplicates removed (n = 3510)
Unique references (n = 13,389)
Title and abstract screening (n = 13,389)
References excluded (n = 13,383)
Full texts assessed (n = 6)
References excluded with reasons (n = 3)
Included studies (n = 3)

	Random sequence generation (selection bias)	Allocation concealment (selection bias)	Blinding of participants and personnel (performance bias): (objective outcomes)	Blinding of participants and personnel (performance bias): (subjective outcomes)	Blinding of outcome assessment (detection bias): (objective outcomes)	Blinding of outcome assessment (detection bias): (subjective outcomes)	Incomplete outcome data (attrition bias)	Selective reporting (reporting bias)	Other bias
Akinaga 2016	?	+	+	+	+	+	−	+	+
Dennehy 1998	+	?	+		+		+	?	+
Mangla 2012	?	?	+	−	+	−	+	?	?

Study or Subgroup	Intramyometrial oxytocin Events	Intramyometrial oxytocin Total	Intravenous oxytocin Events	Intravenous oxytocin Total	Weight	Risk Ratio M-H, Fixed, 95% CI
Akinaga 2016	4	20	2	20	36.4%	2.00 [0.41, 9.71]
Dennehy 1998	0	20	0	19		Not estimable
Mangla 2012	0	50	3	50	63.6%	0.14 [0.01, 2.70]
**Total (95% CI)**		**70**		**70**	**100.0%**	**0.82 [0.25, 2.69]**
Total events	4		5			

Heterogeneity: Chi² = 2.58, df = 1 (P = 0.11); I² = 61%

Test for overall effect: Z = 0.33 (P = 0.74)

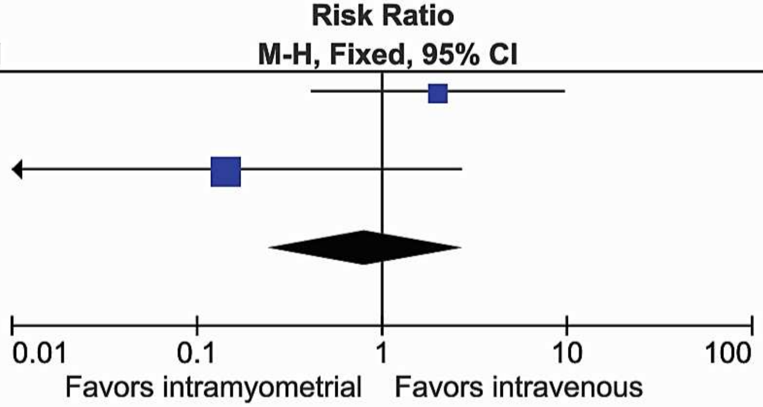

Study or Subgroup	Intramyometrial oxytocin Events	Intramyometrial oxytocin Total	Intravenous oxytocin Events	Intravenous oxytocin Total	Weight	Risk Ratio M-H, Fixed, 95% CI
Akinaga 2016	0	20	1	20	13.0%	0.33 [0.01, 7.72]
Mangla 2012	1	50	10	50	87.0%	0.10 [0.01, 0.75]
**Total (95% CI)**		**70**		**70**	**100.0%**	**0.13 [0.02, 0.69]**
Total events	1		11			

Heterogeneity: Chi² = 0.41, df = 1 (P = 0.52); I^2 = 0%

Test for overall effect: Z = 2.39 (P = 0.02)

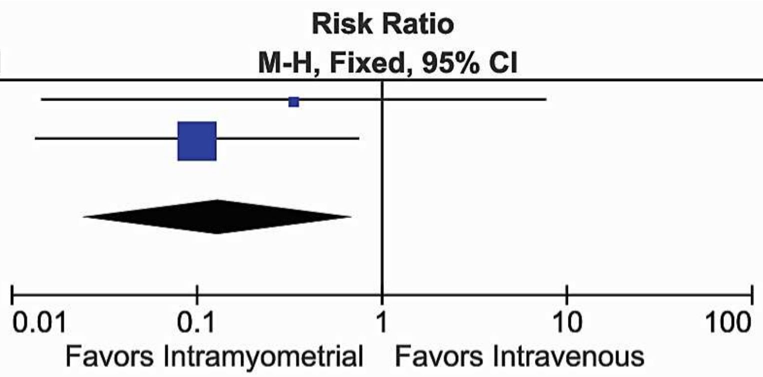

For peer review only

NIHR | National Institute for Health Research

PROSPERO
International prospective register of systematic reviews

Prophylactic oxytocin to prevent postpartum hemorrhage at cesarean section

Maria Regina Torloni, Monica Siaulys, Rachel Riera, Ana Pilar Betran, Mariana Widmer

To enable PROSPERO to focus on COVID-19 registrations during the 2020 pandemic, this registration record was automatically published exactly as submitted. The PROSPERO team has not checked eligibility.

Citation

Maria Regina Torloni, Monica Siaulys, Rachel Riera, Ana Pilar Betran, Mariana Widmer. Prophylactic oxytocin to prevent postpartum hemorrhage at cesarean section. PROSPERO 2020 CRD42020186797 Available from:
https://www.crd.york.ac.uk/prospero/display_record.php?ID=CRD42020186797

Review question

In women submitted to cesarean section, what is the effectiveness and safety of prophylactic oxytocin administered in different periods, regimens, routes, and doses?

Searches

We will develop a search strategy combining key words related to oxytocin and cesarean delivery that will be adapted and run in several databases such as PubMed, Embase, CENTRAL and CINAHL. There will be no date or language limitations.

We will also create and run a search for ongoing studies in trial registry platforms.

The electronic search will be complemented by screening the reference lists of all included studies and other systematic reviews on prevention of postpartum hemorrhage (PPH).

Types of study to be included

All randomized controlled trials of effectiveness or side-effects of oxytocin versus oxytocin to prevent PPH in women submitted to CS will be included.

Condition or domain being studied

Prevention of postpartum hemorrhage. Cesarean section. Oxytocin

Participants/population

Inclusion criteria:

We will include studies that enrolled women of any age, race, socioeconomic level, nationality, with or without any health problems, at high or low risk for PPH, who delivered by cesarean section (CS) for any indication, with any number of fetuses, and at any gestational age after 26 weeks. We will include trials that enrolled women who had a primary or a repeat CS, a prelabour (planned or emergency) CS, or an intrapartum CS. We will include studies where participants received oxytocin for labor induction or augmentation before the CS. Studies that tested oxytocin to prevent PPH in participants having vaginal and cesarean deliveries will be included if the authors present data separately for the women having a CS.

Exclusion criteria:

We will exclude studies that assessed oxytocin in combination with another uterotonic drug, or that compared oxytocin versus placebo or other uterotonic drugs to prevent PPH.

Intervention(s), exposure(s)

We will compare different routes, dosages, regimen, or periods of administration of oxytocin to prevent PPH in women submitted to CS. We will include trials in which non-pharmacologic co-interventions (e.g. uterine massage) were included as a randomized intervention in all arms of the trial.

For peer review only - http://bmjopen.bmj.com/site/about/guidelines.xhtml

Multi-arm trials that compared oxytocin versus other uterotonic drugs, but that also compared different routes, dosages, regimens or periods of administration of oxytocin alone will be included.

Comparator(s)/control

Any route, dosage, regimens or periods of administration of oxytocin to prevent PPH at CS that are different from the intervention group.

Main outcome(s)

1. Postpartum hemorrhage of at least 1000 mL assessed by any method
2. Need for additional uterotonics

3. Any side effects

* Measures of effect

Incidence, Relative risk. We will use other effect measures depending on the data reported in the trials.

Additional outcome(s)

1. Volume of blood loss (mL) measured by any method

2. Blood transfusion after placental delivery

3. Shock, as defined by trialists

4. Maternal mortality or severe maternal morbidity related to PPH (organ failure, coma, hysterectomy, or as defined by the study authors)

5. Maternal transfer to a higher level of care

6. Maternal satisfaction (or sense of wellbeing) as defined by trialists

7. Breastfeeding at discharge

* Measures of effect

Incidence, Relative risk. We will use other effect measures depending on the data reported in the trials.

Data extraction (selection and coding)

The references retrieved from each database will be uploaded into a reference management software and duplicates will be excluded. Two review authors will independently screen titles and abstracts to select potentially relevant records. Studies selected at first screening will be read by two independent authors who will include those that fulfill the selection criteria. Reasons for exclusion will be listed. Disagreements in these two phases will be solved by a third review author.

Two authors will independently conduct data extraction. Disagreements will be solved by a third review author. We will contact study authors by email to obtain missing information and study details, when necessary.

We will use a form created for this review to extract data from each included study. The data will be entered in the Review Manager software and checked for accuracy. The following information will be collected from each study: country, year, trial registration, design details, number of participants, inclusion and exclusion criteria, main participant characteristics, type of CS, anesthesia, previous exposure to oxytocin, interventions compared and outcomes reported.

Risk of bias (quality) assessment

The risk of bias of each included study will be assessed independently by two review authors. Disagreements will be solved by a third reviewer. Full justifications for the assessments will be included in the report.

For peer review only

We will use the Cochrane Risk of Bias (RoB) tool to assess the quality of included trials. The following domains will be assessed and graded as being at high, low, or unclear risk of bias: 1) Random sequence generation, 2) Allocation concealment, 3) Blinding of participants and personnel, 4) Blinding of outcome assessment, 5) Incomplete outcome data, 6) Selective reporting (checking for reporting bias), 7) Other bias.

Strategy for data synthesis

We will pool data of similar trials in metanalyses using the random effect model using the Review Manager software (version 5.3). For dichotomous outcomes, we will present results as summary risk ratio (RR) with 95% confidence intervals (CIs). For continuous outcomes, we will pool the results of studies that measured the outcome in the same way and calculate mean differences (MD). In metanalyses of studies that measured the same continuous outcomes using different methods, we will use the standardized mean difference (SMD).

When metanalysis is not possible, we will present the results descriptively.

We will conduct separate comparisons for prelabor and intrapartum CS.

We plan to conduct the following comparisons, where data is available:

• different moments of administration.

• different routes of administration

• different regimens

• different doses

Analysis of subgroups or subsets

We plan to conduct the following subgroup analyses for the primary outcomes, if data are available: according to pregnancy characteristics (parity, onset of labor), according to previous use of oxytocin for induction/augmentation, and according to baseline risk for PPH. We will assess subgroup differences by interaction tests available in RevMan 5 (RevMan 2014).

We plan to conduct the following sensitivity analyses for the main outcomes, if data are available: restricted only to high-quality studies, and restricted only to studies that assessed blood loss objectively.

Contact details for further information

Maria Regina Torloni
torlonimr@gmail.com

Organisational affiliation of the review

Hospital e Maternidade Santa Joana

Review team members and their organisational affiliations

Dr Maria Regina Torloni. Hospital e Maternidade Santa Joana
Dr Monica Siaulys. Hospital e Maternidade Santa Joana
Dr Rachel Riera. Universidade Federal de Sao Paulo
Dr Ana Pilar Betran. World Health Organization
Dr Mariana Widmer. World Health Organization

Collaborators

Dr Ligia Mathias. Hospital e Maternidade Santa Joana
Dr Guilherme Haelvoet. Hospital e Maternidade Santa Joana
Dr Ana Luiza Martimbianco. Universidade Federal de Sao Paulo
Carolina Cruz Latorraca. Universidade Federal de Sao Paulo
Dr Rafael Leite Pacheco. Universidade Federal de Sao Paulo

For peer review only

Type and method of review
Intervention, Meta-analysis, Systematic review

Anticipated or actual start date
06 April 2020

Anticipated completion date
31 July 2020

Funding sources/sponsors
Department of Sexual and Reproductive Health and Research, World Health Organization

Conflicts of interest

Language
English

Country
Brazil, Switzerland

Stage of review
Review Ongoing

Subject index terms status
Subject indexing assigned by CRD

Subject index terms
MeSH headings have not been applied to this record

Date of registration in PROSPERO
05 July 2020

Date of first submission
17 May 2020

Stage of review at time of this submission

Stage	Started	Completed
Preliminary searches	Yes	No
Piloting of the study selection process	Yes	No
Formal screening of search results against eligibility criteria	No	No
Data extraction	No	No
Risk of bias (quality) assessment	No	No
Data analysis	No	No

The record owner confirms that the information they have supplied for this submission is accurate and complete and they understand that deliberate provision of inaccurate information or omission of data may be construed as scientific misconduct.

The record owner confirms that they will update the status of the review when it is completed and will add publication details in due course.

For peer review only

NIHR | National Institute for Health Research

PROSPERO
International prospective register of systematic reviews

Versions

05 July 2020

PROSPERO
This information has been provided by the named contact for this review. CRD has accepted this information in good faith and registered the review in PROSPERO. The registrant confirms that the information supplied for this submission is accurate and complete. CRD bears no responsibility or liability for the content of this registration record, any associated files or external websites.

For peer review only

Supplementary file 2. Search strategy for systematic review on oxytocin route of delivery

Database	Search strategy	Results
BVS	#1 MH:Cesárea OR MH:"Cesarean Section" OR Cesárea OR (Cesarean Section) OR (Parto Abdominal) OR (Abdominal Deliveries) OR (Abdominal Delivery) OR (C Section (OB)) OR (C-Section (OB)) OR (C-Sections (OB)) OR (Caesarean Section) OR (Caesarean Sections) OR (Cesarean Sections) OR (Postcesarean Section) OR MH:E04.520.252.500$ OR MH:"Cesárea Repetida" OR MH:"Cesarean Section, Repeat" OR MH:Recesariana OR (Cesárea Repetida) OR Recesariana OR (Repeat Cesarean Section) OR (Repeat Cesarean Sections) OR (Operación Cesárea Repetida) OR MH:E04.520.252.500.150$ OR MH:"Parto Obstétrico" OR MH:"Delivery, Obstetric" OR (Parto Obstétrico) OR (Liberação Obstétrica) OR (Obstetric Deliveries) OR (Obstetric Delivery) OR (Liberación Obstétrica) OR MH:E04.520.252$ OR MH:"Terceira Fase do Trabalho de Parto" OR MH:"Labor Stage, Third" OR MH:"Tercer Periodo del Trabajo de Parto" OR (Terceira Fase do Trabalho de Parto) OR (Tercer Periodo del Trabajo de Parto) OR (Terceiro Estágio do Trabalho de Parto) OR (Terceiro Período do Trabalho de Parto) OR (Third Labor Stage) OR (Third Labor Stages) OR (Third Stage Labor) OR MH:G08.686.784.769.326.500.110$<br><br>#2 MH:Oxitocina OR MH:Oxytocin OR MH:Ocitocina OR Oxitocina OR Oxytocin OR Ocitocina OR Ocytocin OR Pitocin OR Syntocinon OR MH:D06.472.699.631.692.433$ OR MH:D12.644.548.691.692.433$ OR MH:"Receptores de Ocitocina" OR MH:"Receptors, Oxytocin" OR MH:"Receptores de Oxitocina" OR (Receptores de Ocitocina) OR (Receptores de Oxitocina) OR (Oxytocin Receptor) OR (Oxytocin Receptors) OR MH:D12.776.543.750.695.630$ OR MH:D12.776.543.750.720.600.630$ OR MH:D12.776.543.750.750.555.630$ OR MH:D12.776.543.750.750.660.600$ OR MH:Ocitócicos OR MH:Oxytocics OR MH:Oxitócicos OR Ocitócicos OR Oxytocics OR Oxitócicos OR (Efeito Ocitócico) OR (Estimulantes Uterinos) OR (Oxytocic Agents) OR (Oxytocic Drugs) OR (Oxytocic Effect) OR (Oxytocic Effects) OR (Uterine Stimulants) OR (Efecto Oxitócico) OR MH:D27.505.696.875.737$ OR MH:D27.505.954.705.737$<br><br>#3 #1 AND #2	376
CINAHL (via EBSCO)	S1 MH Cesarean Section OR ((Cesarean Sections) OR (Cesarean Section) OR (Abdominal Deliveries) OR (Abdominal Deliver) OR (Abdominal Delivery) OR (C-Section (OB)) OR (C Section (OB)) OR (C-Sections (OB)) OR (Postcesarean Section) OR (cesarean delivery) OR (Cesarean Section) OR (Elective cesarean section) OR (Elective cesarean delivery) OR Cesarean OR (Emergency cesarean section) OR (Emergency cesarean delivery) OR (Intrapartum cesarean section) OR (Intrapartum cesarean delivery) OR (Intra-partum cesarean section) OR (Intra-partum cesarean delivery) OR (Intra partum cesarean section) OR (Intra partum cesarean delivery) OR (Caesarean Section) OR (Caesarean Sections) OR (Postcaesarean Section) OR (caesarean delivery) OR (Elective caesarean section) OR (Elective caesarean delivery) OR Caesarean OR (Emergency caesarean section) OR (Emergency caesarean delivery) OR (Intrapartum caesarean section) OR (Intrapartum caesarean delivery) OR (Intra-partum caesarean section) OR (Intra-partum caesarean delivery) OR (Intra partum caesarean section) OR (Intra partum caesarean delivery) OR (Prelabor cesarean section) OR (Prelabor cesarean delivery) OR (Pre-labor cesarean delivery) OR (Pre-labor cesarean delivery) OR (Pre labor cesarean section) OR (Pre labor cesarean delivery) OR (Prelabour cesarean section) OR (Prelabour cesarean delivery) OR (Pre-labour cesarean delivery) OR (Pre-labour cesarean delivery) OR (Pre labour cesarean section) OR (Pre labour cesarean delivery) OR (Prelabor caesarean section) OR (Prelabor caesarean delivery) OR (Pre-labor caesarean delivery) OR (Pre-labor caesarean delivery) OR (Pre labor caesarean section) OR (Pre labor caesarean delivery) OR (Prelabour caesarean section) OR (Prelabour caesarean delivery) OR (Pre-labour caesarean delivery) OR (Pre-labour caesarean delivery) OR (Pre labour caesarean section) OR (Pre labour caesarean delivery) OR (Repeat Cesarean Section) OR (Repeat Cesarean Sections) OR (Repeat Caesarean Section) OR (Repeat Caesarean Sections) OR (Obstetric anesthesia) OR (Obstetric Deliveries) OR (Obstetric Delivery) OR (Third Labor Stage) OR (Third Labor Stages) OR (Third Stage Labor) OR (Third Stage of Labor) OR (Third-stage labor) OR (Third Labour Stage) OR (Third Labour Stages) OR (Third Stage Labour) OR (Third Stage of Labour) OR (Third-stage labour)) OR MH Cesarean Section, Repeat OR MH Delivery, Obstetric OR MH Labor Stage, Third<br><br>S2 MH Oxytocin OR MH Receptors, Oxytocin OR MH Oxytocics OR (Oxytocin OR Ocytocin OR Syntocinon OR Pitocin OR (Oxytocin Receptor) OR (Oxytocin Receptors) OR (alpha hypophamine) OR (atonin o) OR (beta methyl beta mercaptobutyric acid oxytocin) OR (di sipidin) OR disipidin OR endopituitrin OR fetusin OR hypophysin OR hypophysine OR (intravenous oxytocin) OR mipareton OR neoxyn OR ocytocin OR orasthin OR orastina OR oxitocin OR (oxiton inj) OR oxitone OR oxystin OR oxytan OR (oxytocin 10 usp units in dextrose 5%) OR (oxytocin 20 usp units in dextrose 5%) OR (oxytocin 5 usp units in dextrose 5%) OR (oxytocin dimer) OR (oxytocin pentapeptide) OR (oxytocin s inj) OR oxytocine OR pareton OR partacon OR partocon OR (partocon inj) OR partolact OR partoxin OR physormon OR pitocin OR (pitocin inj) OR piton OR (piton s) OR (piton s inj) OR pituilobine OR pitupartin OR solvoxine OR synpitan OR (synthetic oxytocin inj) OR syntocinon OR (syntocinon inj) OR (syntocinon spray) OR tranoxy OR utedrin OR uteracon OR uterason OR (utron inj) OR xitocin OR Oxytocics OR (Oxytocic Drugs) OR (Uterine Stimulants) OR (Oxytocic Agents) OR (Oxytocic Effect) OR (Oxytocic Effects)<br><br>S3 S1 AND S2	1,563

ClinicalTrials.gov	Condition: (Cesarean Sections) OR (Cesarean Section) OR (Delivery, Abdominal) OR (Abdominal Deliveries) OR (Abdominal Deliver) OR (Deliveries, Abdominal) OR (Abdominal Delivery) OR "C-Section (OB)" OR "C Section (OB)" OR "C-Sections (OB)"<br><br>Intervention: Oxytocin OR Ocytocin OR Syntocinon OR Pitocin OR (Oxytocin Receptor) OR (Receptor, Oxytocin) OR (Oxytocin Receptors) OR (alpha hypophamine) OR "atonin o" OR "beta methyl beta mercaptobutyric acid oxytocin" OR "di sipidin" OR disipidin	90
Cochrane Library	#1 MeSH descriptor: [Cesarean Section] explode all trees<br>#2 (Cesarean Sections) OR (Cesarean Section) OR (Delivery, Abdominal) OR (Abdominal Deliveries) OR (Abdominal Deliver) OR (Deliveries, Abdominal) OR (Abdominal Delivery) OR "C-Section (OB)" OR "C Section (OB)" OR "C-Sections (OB)" OR (Postcesarean Section) OR (cesarean delivery) OR (Cesarean Section) OR (Elective cesarean section) OR (Elective cesarean delivery) OR Cesarean OR (Emergency cesarean section) OR (Emergency cesarean delivery) OR (Intrapartum cesarean section) OR (Intrapartum cesarean delivery) OR (Intra-partum cesarean section) OR (Intra-partum cesarean delivery) OR (Intra partum cesarean section) OR (Intra partum cesarean delivery)<br>#3 (Caesarean Section) OR (Caesarean Sections) OR (Postcaesarean Section) OR (caesarean delivery) OR (Elective caesarean section) OR (Elective caesarean delivery) OR Caesarean OR (Emergency caesarean section) OR (Emergency caesarean delivery) OR (Intrapartum caesarean section) OR (Intrapartum caesarean delivery) OR (Intra-partum caesarean section) OR (Intra-partum caesarean delivery) OR (Intra partum caesarean section) OR (Intra partum caesarean delivery)<br>#4 (Prelabor cesarean section) OR (Prelabor cesarean delivery) OR (Pre-labor cesarean delivery) OR (Pre-labor cesarean delivery) OR (Pre labor cesarean section) OR (Pre labor cesarean delivery)<br>#5 (Prelabour cesarean section) OR (Prelabour cesarean delivery) OR (Pre-labour cesarean delivery) OR (Pre-labour cesarean delivery) OR (Pre labour cesarean section) OR (Pre labour cesarean delivery)<br>#6 (Prelabor caesarean section) OR (Prelabor caesarean delivery) OR (Pre-labor caesarean delivery) OR (Pre-labor caesarean delivery) OR (Pre labor caesarean section) OR (Pre labor caesarean delivery)<br>#7 (Prelabour caesarean section) OR (Prelabour caesarean delivery) OR (Pre-labour caesarean delivery) OR (Pre-labour caesarean delivery) OR (Pre labour caesarean section) OR (Pre labour caesarean delivery)<br>#8 MeSH descriptor: [Cesarean Section, Repeat] explode all trees<br>#9 (Repeat Cesarean Section) OR (Cesarean Sections, Repeat) OR (Repeat Cesarean Sections) OR (Section, Repeat Cesarean) OR (Sections, Repeat Cesarean)<br>#10 (Repeat Caesarean Section) OR (Caesarean Sections, Repeat) OR (Repeat Caesarean Sections) OR (Section, Repeat Caesarean) OR (Sections, Repeat Caesarean) OR (Obstetric anesthesia)<br>#11 MeSH descriptor: [Delivery, Obstetric] explode all trees<br>#12 (Deliveries, Obstetric) OR (Obstetric Deliveries) OR (Obstetric Delivery)<br>#13 MeSH descriptor: [Labor Stage, Third] explode all trees<br>#14 (Labor Stages, Third) OR (Stage, Third Labor) OR (Stages, Third Labor) OR (Third Labor Stage) OR (Third Labor Stages) OR (Labor, Third Stage) OR (Third Stage Labor) OR "Third Stage of Labor" OR "Third-stage labor"<br>#15 (Labour Stage, Third) OR (Labour Stages, Third) OR (Stage, Third Labour) OR (Stages, Third Labour) OR (Third Labour Stage) OR (Third Labour Stages) OR (Labour, Third Stage) OR (Third Stage Labour) OR "Third Stage of Labour" OR "Third-stage labour"<br>#16 #1 OR #2 OR #3 OR #4 OR #5 OR #6 OR #7 OR #8 OR #9 OR #10 OR #11 OR #12 OR #13 OR #14 OR #15<br>#17 MeSH descriptor: [Oxytocin] explode all trees<br>#18 Oxytocin OR Ocytocin OR Syntocinon OR Pitocin<br>#19 MeSH descriptor: [Receptors, Oxytocin] explode all trees<br>#20 (Oxytocin Receptor) OR (Receptor, Oxytocin) OR (Oxytocin Receptors) OR (alpha hypophamine) OR "atonin o" OR "beta methyl beta mercaptobutyric acid oxytocin" OR "di sipidin" OR disipidin OR endopituitrin OR fetusin OR hypophysin OR hypophysine OR (intravenous oxytocin) OR mipareton OR neoxyn OR ocytocin OR orasthin OR orastina OR oxitocin OR "oxiton inj" OR oxitone OR oxystin OR oxytan OR (oxytocin 10 usp units in dextrose 5%) OR (oxytocin 20 usp units in dextrose 5%) OR (oxytocin 5 usp units in dextrose 5%) OR (oxytocin dimer) OR (oxytocin pentapeptide) OR "oxytocin s inj" OR oxytocine OR pareton OR partacon OR partocon OR "partocon inj" OR partolact OR partoxin OR physormon OR pitocin OR "pitocin inj" OR piton OR "piton s" OR "piton s inj" OR pituilobine OR pitupartin OR solvoxine OR synpitan OR (synthetic oxytocin inj) OR syntocinon OR "syntocinon inj" OR (syntocinon spray) OR tranoxy OR utedrin OR uteracon OR uterason OR "utron inj" OR xitocin<br>#21 MeSH descriptor: [Oxytocics] explode all trees<br>#22 Oxytocics OR (Oxytocic Drugs) OR (Drugs, Oxytocic) OR (Uterine Stimulants) OR (Stimulants, Uterine) OR (Oxytocic Agents) OR (Agents, Oxytocic) OR (Oxytocic Effect) OR (Effect, Oxytocic) OR (Oxytocic Effects) OR (Effects, Oxytocic)<br>#23 #17 OR #18 OR #19 OR #20 OR #21 OR #22<br>#24 #16 AND #23<br>#25 #24 in Trials	2,375
Embase	#1 'cesarean section'/exp OR 'cesarean section' OR 'birth, abdominal operation'/exp OR 'birth, abdominal operation' OR 'birth, caesarean'/exp OR 'birth, caesarean' OR 'caesarean birth'/exp OR 'caesarean birth' OR 'caesarean section'/exp OR 'caesarean section' OR 'caesarian birth'/exp OR 'caesarian birth' OR 'caesarian section'/exp OR 'caesarian section' OR 'cesarean delivery'/exp OR 'cesarean delivery' OR 'cesarian section'/exp OR 'cesarian section' OR 'elective repeat cesarean	3,804

For peer review only

	section'/exp OR 'elective repeat cesarean section' OR 'fetectomy'/exp OR 'fetectomy' OR 'repeated cesarotomy'/exp OR 'repeated cesarotomy' OR 'sectio caesarea'/exp OR 'sectio caesarea'<br><br>#2 'repeat cesarean section'/exp OR 'repeat cesarean section' OR 'cesarean section, repeat'/exp OR 'cesarean section, repeat' OR 'repeat cesarean section, elective'/exp OR 'repeat cesarean section, elective' OR 'repeat cesarotomy'/exp OR 'repeat cesarotomy' OR 'repeat section, cesarean'/exp OR 'repeat section, cesarean' OR 'repeated cesarean section'/exp OR 'repeated cesarean section' OR 'section, elective repeat cesarean'/exp OR 'section, elective repeat cesarean' OR 'section, repeat cesarean'/exp OR 'section, repeat cesarean'<br><br>#3 'obstetric delivery'/exp OR 'obstetric delivery' OR 'delivery'/exp OR delivery OR 'delivery pattern'/exp OR 'delivery pattern' OR 'delivery term'/exp OR 'delivery term' OR 'delivery, obstetric'/exp OR 'delivery, obstetric'<br><br>#4 'labor stage 3'/exp OR 'labor stage 3' OR 'delivery stage 3'/exp OR 'delivery stage 3' OR 'delivery stage iii'/exp OR 'delivery stage iii' OR 'labor stage iii'/exp OR 'labor stage iii' OR 'labor stage, third'/exp OR 'labor stage, third' OR 'labour stage 3'/exp OR 'labour stage 3' OR 'labour stage iii'/exp OR 'labour stage iii' OR 'labour stage, third'/exp OR 'labour stage, third' OR 'third delivery stage'/exp OR 'third delivery stage' OR 'third labor stage'/exp OR 'third labor stage' OR 'third labour stage'/exp OR 'third labour stage' OR 'third stage of delivery'/exp OR 'third stage of delivery' OR 'third stage of labor'/exp OR 'third stage of labor' OR 'third stage of labour'/exp OR 'third stage of labour'<br><br>#5 #1 OR #2 OR #3 OR #4<br><br>#6 'oxytocin' OR 'oxytocin'/exp OR oxytocin OR 'alpha hypophamine'/exp OR 'alpha hypophamine' OR 'atonin o'/exp OR 'atonin o' OR 'beta methyl beta mercaptobutyric acid oxytocin'/exp OR 'beta methyl beta mercaptobutyric acid oxytocin' OR 'di sipidin'/exp OR 'di sipidin' OR 'disipidin'/exp OR disipidin OR 'endopituitrin'/exp OR endopituitrin OR 'fetusin'/exp OR fetusin OR 'hypophysin'/exp OR hypophysin OR 'hypophysine'/exp OR hypophysine OR 'intravenous oxytocin'/exp OR 'intravenous oxytocin' OR 'mipareton'/exp OR mipareton OR 'neoxyn'/exp OR neoxyn OR 'ocytocin'/exp OR ocytocin OR 'opn 300'/exp OR 'opn 300' OR 'opn300'/exp OR opn300 OR 'orasthin'/exp OR orasthin OR 'orastina'/exp OR orastina OR 'oxitocin'/exp OR oxitocin OR 'oxiton inj'/exp OR 'oxiton inj' OR 'oxitone'/exp OR oxitone OR 'oxystin'/exp OR oxystin OR 'oxytan'/exp OR oxytan OR 'oxytocin 10 usp units in dextrose 5%'/exp OR 'oxytocin 10 usp units in dextrose 5%' OR 'oxytocin 20 usp units in dextrose 5%'/exp OR 'oxytocin 20 usp units in dextrose 5%' OR 'oxytocin 5 usp units in dextrose 5%'/exp OR 'oxytocin 5 usp units in dextrose 5%' OR 'oxytocin dimer'/exp OR 'oxytocin dimer' OR 'oxytocin pentapeptide'/exp OR 'oxytocin pentapeptide' OR 'oxytocin s inj'/exp OR 'oxytocin s inj' OR 'oxytocina'/exp OR oxytocina OR 'oxytocine'/exp OR oxytocine OR 'pareton'/exp OR pareton OR 'partacon'/exp OR partacon OR 'partocon'/exp OR partocon OR 'partocon inj'/exp OR 'partocon inj' OR 'partolact'/exp OR partolact OR 'partoxin'/exp OR partoxin OR 'physormon'/exp OR physormon OR 'pitocin'/exp OR pitocin OR 'pitocin inj'/exp OR 'pitocin inj' OR 'piton'/exp OR piton OR 'piton s'/exp OR 'piton s' OR 'piton s inj'/exp OR 'piton s inj' OR 'pituilobine'/exp OR pituilobine OR 'pitupartin'/exp OR pitupartin OR 'solvoxine'/exp OR solvoxine OR 'synpitan'/exp OR synpitan OR 'synthetic oxytocin inj'/exp OR 'synthetic oxytocin inj' OR 'syntocinon'/exp OR syntocinon OR 'syntocinon inj'/exp OR 'syntocinon inj' OR 'syntocinon spray'/exp OR 'syntocinon spray' OR 'ti 001'/exp OR 'ti 001' OR 'ti 114'/exp OR 'ti 114' OR 'ti001'/exp OR 'ti001' OR 'ti114'/exp OR 'ti114' OR 'tranoxy'/exp OR tranoxy OR 'tur 001'/exp OR 'tur 001' OR 'tur001'/exp OR 'tur001' OR 'utedrin'/exp OR utedrin OR 'uteracon'/exp OR uteracon OR 'uterason'/exp OR uterason OR 'utron inj'/exp OR 'utron inj' OR 'vagitocin'/exp OR vagitocin OR 'xitocin'/exp OR xitocin<br><br>#7 'oxytocin receptor'/exp OR 'oxytocin receptor' OR 'neuron, oxytocinergic'/exp OR 'neuron, oxytocinergic' OR 'oxytocinergic nerve cell'/exp OR 'oxytocinergic nerve cell' OR 'oxytocinergic neuron'/exp OR 'oxytocinergic neuron' OR 'receptor, oxytocin'/exp OR 'receptor, oxytocin' OR 'receptors, oxytocin'/exp OR 'receptors, oxytocin'<br><br>#8 'oxytocic agent'/exp OR 'oxytocic agent' OR 'labor inducing drug'/exp OR 'labor inducing drug' OR 'labour inducing drug'/exp OR 'labour inducing drug' OR 'ocytocic agent'/exp OR 'ocytocic agent' OR 'oxytocic activity'/exp OR 'oxytocic activity' OR 'oxytocic substance'/exp OR 'oxytocic substance' OR 'oxytocics'/exp OR oxytocics<br><br>#9 #6 OR #7 OR #8<br>#10 #5 AND #9<br>#11 #10 AND [embase]/lim NOT ([embase]/lim AND [medline]/lim)	
Global Index Medicus	MH:Cesárea OR MH:"Cesarean Section" OR Cesárea OR (Cesarean Section) OR (Parto Abdominal) OR (Abdominal Deliveries) OR (Abdominal Delivery) OR (C Section (OB)) OR (C-Section (OB)) OR (C-Sections (OB)) OR (Caesarean Section) OR (Caesarean Sections) OR (Cesarean Sections) OR (Postcesarean Section) OR MH:E04.520.252.500$ OR MH:"Cesárea Repetida" OR MH:"Cesarean Section, Repeat" OR MH:Recesariana OR (Cesárea Repetida) OR Recesariana OR (Repeat Cesarean Section) OR (Repeat Cesarean Sections) OR (Operación Cesárea Repetida) OR MH:E04.520.252.500.150$ OR MH:"Parto Obstétrico" OR MH:"Delivery, Obstetric" OR (Parto Obstétrico) OR (Liberação Obstétrica) OR (Obstetric Deliveries) OR (Obstetric Delivery) OR (Liberación Obstétrica) OR MH:E04.520.252$ OR MH:"Terceira Fase do Trabalho de Parto" OR MH:"Labor Stage, Third" OR MH:"Tercer Periodo del Trabajo de Parto" OR (Terceira Fase do Trabalho de Parto) OR (Tercer Periodo del Trabajo de Parto) OR (Terceiro Estágio do Trabalho de	421

	Parto) OR (Terceiro Período do Trabalho de Parto) OR (Third Labor Stage) OR (Third Labor Stages) OR (Third Stage Labor) OR MH:G08.686.784.769.326.500.110$<br>AND<br>MH:Oxitocina OR MH:Oxytocin OR MH:Ocitocina OR Oxitocina OR Oxytocin OR Ocitocina OR Ocytocin OR Pitocin OR Syntocinon OR MH:D06.472.699.631.692.433$ OR MH:D12.644.548.691.692.433$ OR MH:"Receptores de Ocitocina" OR MH:"Receptors, Oxytocin" OR MH:"Receptores de Oxitocina" OR (Receptores de Ocitocina) OR (Receptores de Oxitocina) OR (Oxytocin Receptor) OR (Oxytocin Receptors) OR MH:D12.776.543.750.695.630$ OR MH:D12.776.543.750.720.600.630$ OR MH:D12.776.543.750.750.555.630$ OR MH:D12.776.543.750.750.660.600$ OR MH:Ocitócicos OR MH:Oxytocics OR MH:Oxitócicos OR Ocitócicos OR Oxytocics OR Oxitócicos OR (Efeito Ocitócico) OR (Estimulantes Uterinos) OR (Oxytocic Agents) OR (Oxytocic Drugs) OR (Oxytocic Effect) OR (Oxytocic Effects) OR (Uterine Stimulants) OR (Efecto Oxitócico) OR MH:D27.505.696.875.737$ OR MH:D27.505.954.705.737$	
MEDLINE (via PubMed)	#1 "Cesarean Section"[Mesh] OR (Cesarean Sections) OR (Cesarean Section) OR (Delivery, Abdominal) OR (Abdominal Deliveries) OR (Abdominal Deliver) OR (Deliveries, Abdominal) OR (Abdominal Delivery) OR "C-Section (OB)" OR "C Section (OB)" OR "C-Sections (OB)" OR (Postcesarean Section) OR (cesarean delivery) OR (Cesarean Section) OR (Elective cesarean section) OR (Elective cesarean delivery) OR Cesarean OR (Emergency cesarean section) OR (Emergency cesarean delivery) OR (Intrapartum cesarean section) OR (Intrapartum cesarean delivery) OR (Intra-partum cesarean section) OR (Intra-partum cesarean delivery) OR (Intra partum cesarean section) OR (Intra partum cesarean delivery)<br><br>#2 (Caesarean Section) OR (Caesarean Sections) OR (Postcaesarean Section) OR (caesarean delivery) OR (Elective caesarean section) OR (Elective caesarean delivery) OR Caesarean OR (Emergency caesarean section) OR (Emergency caesarean delivery) OR (Intrapartum caesarean section) OR (Intrapartum caesarean delivery) OR (Intra-partum caesarean section) OR (Intra-partum caesarean delivery) OR (Intra partum caesarean section) OR (Intra partum caesarean delivery)<br><br>#3 (Prelabor cesarean section) OR (Prelabor cesarean delivery) OR (Pre-labor cesarean delivery) OR (Pre-labor cesarean delivery) OR (Pre labor cesarean section) OR (Pre labor cesarean delivery)<br><br>#4 (Prelabour cesarean section) OR (Prelabour cesarean delivery) OR (Pre-labour cesarean delivery) OR (Pre-labour cesarean delivery) OR (Pre labour cesarean section) OR (Pre labour cesarean delivery)<br><br>#5 (Prelabor caesarean section) OR (Prelabor caesarean delivery) OR (Pre-labor caesarean delivery) OR (Pre-labor caesarean delivery) OR (Pre labor caesarean section) OR (Pre labor caesarean delivery)<br>#6 (Prelabour caesarean section) OR (Prelabour caesarean delivery) OR (Pre-labour caesarean delivery) OR (Pre-labour caesarean delivery) OR (Pre labour caesarean section) OR (Pre labour caesarean delivery)<br><br>#7 "Cesarean Section, Repeat'[Mesh] OR (Repeat Cesarean Section) OR (Cesarean Sections, Repeat) OR (Repeat Cesarean Sections) OR (Section, Repeat Cesarean) OR (Sections, Repeat Cesarean)<br><br>#8 (Repeat Caesarean Section) OR (Caesarean Sections, Repeat) OR (Repeat Caesarean Sections) OR (Section, Repeat Caesarean) OR (Sections, Repeat Caesarean) OR (Obstetric anesthesia)<br><br>#9 "Delivery, Obstetric"[Mesh] OR (Deliveries, Obstetric) OR (Obstetric Deliveries) OR (Obstetric Delivery)<br><br>#10 "Labor Stage, Third"[Mesh] OR (Labor Stages, Third) OR (Stage, Third Labor) OR (Stages, Third Labor) OR (Third Labor Stage) OR (Third Labor Stages) OR (Labor, Third Stage) OR (Third Stage Labor) OR "Third Stage of Labor" OR "Third-stage labor"<br><br>#11 (Labour Stage, Third) OR (Labour Stages, Third) OR (Stage, Third Labour) OR (Stages, Third Labour) OR (Third Labour Stage) OR (Third Labour Stages) OR (Labour, Third Stage) OR (Third Stage Labour) OR "Third Stage of Labour" OR "Third-stage labour"<br><br>#12 #1 OR #2 OR #3 OR #4 OR #5 OR #6 OR #7 OR #8 OR #9 OR #10 OR #11<br><br>#13 "Oxytocin"[Mesh] OR Oxytocin OR Ocytocin OR Syntocinon OR Pitocin<br><br>#14 "Receptors, Oxytocin"[Mesh] OR (Oxytocin Receptor) OR (Receptor, Oxytocin) OR (Oxytocin Receptors) OR (alpha hypophamine) OR "atonin o" OR "beta methyl beta mercaptobutyric acid oxytocin" OR "di sipidin" OR disipidin OR endopituitrin OR fetusin OR hypophysin OR hypophysine OR (intravenous oxytocin) OR mipareton OR neoxyn OR ocytocin OR orasthin OR orastina OR oxitocin OR "oxiton inj" OR oxitone OR oxystin OR oxytan OR (oxytocin 10 usp units in dextrose 5%) OR (oxytocin 20 usp units in dextrose 5%) OR (oxytocin 5 usp units in dextrose 5%) OR (oxytocin dimer) OR (oxytocin pentapeptide) OR "oxytocin s inj" OR oxytocine OR pareton OR partacon OR partocon OR "partocon inj" OR partolact OR partoxin OR physormon OR pitocin OR "pitocin inj" OR piton OR "piton s" OR "piton s inj" OR pituilobine OR pitupartin OR solvoxine OR synpitan OR	7,944

For peer review only

	(synthetic oxytocin inj) OR syntocinon OR "syntocinon inj" OR (syntocinon spray) OR tranoxy OR utedrin OR uteracon OR uterason OR "utron inj" OR xitocin<br>#15 "Oxytocics"[Mesh] OR Oxytocics OR (Oxytocic Drugs) OR (Drugs, Oxytocic) OR (Uterine Stimulants) OR (Stimulants, Uterine) OR (Oxytocic Agents) OR (Agents, Oxytocic) OR (Oxytocic Effect) OR (Effect, Oxytocic) OR (Oxytocic Effects) OR (Effects, Oxytocic)<br><br>#16 "Oxytocics" [Pharmacological Action]<br>#17 #13 OR #14 OR #15 OR #16<br>#18 #12 AND #17<br>#19 ((clinical[Title/Abstract] AND trial[Title/Abstract]) OR clinical trials as topic[MeSH Terms] OR clinical trial[Publication Type] OR random*[Title/Abstract] OR random allocation[MeSH Terms] OR therapeutic use[MeSH Subheading])<br>#20 #18 AND #19	
Opengrey	Cesarean Sections) OR (Cesarean Section) OR (Delivery, Abdominal) OR (Abdominal Deliveries) OR (Abdominal Deliver) OR (Deliveries, Abdominal) OR (Abdominal Delivery) OR "C-Section (OB)" OR "C Section (OB)" OR "C-Sections (OB)" OR (Postcesarean Section) OR (cesarean delivery) OR (Cesarean Section) OR (Elective cesarean section) OR (Elective cesarean delivery) OR Cesarean OR (Emergency cesarean section) OR (Emergency cesarean delivery) OR (Intrapartum cesarean section) OR (Intrapartum cesarean delivery) OR (Intra-partum cesarean section) OR (Intra-partum cesarean delivery) OR (Intra partum cesarean section) OR (Intra partum cesarean delivery) OR (Caesarean Section) OR (Caesarean Sections) OR (Postcaesarean Section) OR (caesarean delivery) OR (Elective caesarean section) OR (Elective caesarean delivery) OR Caesarean OR (Emergency caesarean section) OR (Emergency caesarean delivery) OR (Intrapartum caesarean section) OR (Intrapartum caesarean delivery) OR (Intra-partum caesarean section) OR (Intra-partum caesarean delivery) OR (Intra partum caesarean section) OR (Intra partum caesarean delivery) OR (Prelabor cesarean section) OR (Prelabor cesarean delivery) OR (Pre-labor cesarean delivery) OR (Pre-labor cesarean delivery) OR (Pre labor cesarean section) OR (Pre labor cesarean delivery) OR (Prelabour cesarean section) OR (Prelabour cesarean delivery) OR (Pre-labour cesarean delivery) OR (Pre-labour cesarean delivery) OR (Pre labour cesarean section) OR (Pre labour cesarean delivery) OR (Prelabor caesarean section) OR (Prelabor caesarean delivery) OR (Pre-labor caesarean delivery) OR (Pre-labor caesarean delivery) OR (Pre labor caesarean section) OR (Pre labor caesarean delivery) OR (Prelabour caesarean section) OR (Prelabour caesarean delivery) OR (Pre-labour caesarean delivery) OR (Pre-labour caesarean delivery) OR (Pre labour caesarean section) OR (Pre labour caesarean delivery) OR (Repeat Cesarean Section) OR (Cesarean Sections, Repeat) OR (Repeat Cesarean Sections) OR (Section, Repeat Cesarean) OR (Sections, Repeat Cesarean) OR (Repeat Caesarean Section) OR (Caesarean Sections, Repeat) OR (Repeat Caesarean Sections) OR (Section, Repeat Caesarean) OR (Sections, Repeat Caesarean) OR (Obstetric anesthesia) OR (Deliveries, Obstetric) OR (Obstetric Deliveries) OR (Obstetric Delivery) OR (Labor Stages, Third) OR (Stage, Third Labor) OR (Stages, Third Labor) OR (Third Labor Stage) OR (Third Labor Stages) OR (Labor, Third Stage) OR (Third Stage Labor) OR "Third Stage of Labor" OR "Third-stage labor" OR (Labour Stage, Third) OR (Labour Stages, Third) OR (Stage, Third Labour) OR (Stages, Third Labour) OR (Third Labour Stage) OR (Third Labour Stages) OR (Labour, Third Stage) OR (Third Stage Labour) OR "Third Stage of Labour" OR "Third-stage labour"	126
SciELO	((Cesarean Sections) OR (Cesarean Section) OR (Abdominal Deliveries) OR (Abdominal Deliver) OR (Abdominal Delivery) OR (C-Section (OB)) OR (C Section (OB)) OR (C-Sections (OB)) OR (Postcesarean Section) OR (cesarean delivery) OR (Cesarean Section) OR (Elective cesarean section) OR (Elective cesarean delivery) OR Cesarean OR (Emergency cesarean section) OR (Emergency cesarean delivery) OR (Intrapartum cesarean section) OR (Intrapartum cesarean delivery) OR (Intra-partum cesarean section) OR (Intra-partum cesarean delivery) OR (Intra partum cesarean section) OR (Intra partum cesarean delivery) OR (Caesarean Section) OR (Caesarean Sections) OR (Postcaesarean Section) OR (caesarean delivery) OR (Elective caesarean section) OR (Elective caesarean delivery) OR Caesarean OR (Emergency caesarean section) OR (Emergency caesarean delivery) OR (Intrapartum caesarean section) OR (Intrapartum caesarean delivery) OR (Intra-partum caesarean section) OR (Intra-partum caesarean delivery) OR (Intra partum caesarean section) OR (Intra partum caesarean delivery) OR (Prelabor cesarean section) OR (Prelabor cesarean delivery) OR (Pre-labor cesarean delivery) OR (Pre-labor cesarean delivery) OR (Pre labor cesarean section) OR (Pre labor cesarean delivery) OR (Prelabour cesarean section) OR (Prelabour cesarean delivery) OR (Pre-labour cesarean delivery) OR (Pre-labour cesarean delivery) OR (Pre labour cesarean section) OR (Pre labour cesarean delivery) OR (Prelabor caesarean section) OR (Prelabor caesarean delivery) OR (Pre-labor caesarean delivery) OR (Pre-labor caesarean delivery) OR (Pre labor caesarean section) OR (Pre labor caesarean delivery) OR (Prelabour caesarean section) OR (Prelabour caesarean delivery) OR (Pre-labour caesarean delivery) OR (Pre-labour caesarean delivery) OR (Pre labour caesarean section) OR (Pre labour caesarean delivery) OR (Repeat Cesarean Section) OR (Repeat Cesarean Sections) OR (Repeat Caesarean Section) OR (Repeat Caesarean Sections) OR (Obstetric anesthesia) OR (Obstetric Deliveries) OR (Obstetric Delivery) OR (Third Labor Stage) OR (Third Labor Stages) OR (Third Stage Labor) OR (Third Stage of Labor) OR (Third-stage labor) OR (Third Labour Stage) OR (Third Labour Stages) OR (Third Stage Labour) OR (Third Stage of Labour) OR (Third-stage labour))<br>AND<br>(Oxytocin OR Ocytocin OR Syntocinon OR Pitocin OR (Oxytocin Receptor) OR (Oxytocin Receptors) OR (alpha hypophamine) OR (atonin o) OR (beta methyl beta mercaptobutyric acid oxytocin) OR (di sipidin) OR disipidin OR endopituitrin OR fetusin OR hypophysin OR hypophysine OR (intravenous oxytocin) OR mipareton OR neoxyn OR ocytocin OR orasthin OR orastina OR oxitocin OR (oxiton inj) OR oxitone OR oxystin OR oxytan OR (oxytocin 10 usp units in dextrose 5%) OR (oxytocin 20 usp	91

For peer review only - http://bmjopen.bmj.com/site/about/guidelines.xhtml

	units in dextrose 5%) OR (oxytocin 5 usp units in dextrose 5%) OR (oxytocin dimer) OR (oxytocin pentapeptide) OR (oxytocin s inj) OR oxytocine OR pareton OR partacon OR partocon OR (partocon inj) OR partolact OR partoxin OR physormon OR pitocin OR (pitocin inj) OR piton OR (piton s) OR (piton s inj) OR pituilobine OR pitupartin OR solvoxine OR synpitan OR (synthetic oxytocin inj) OR syntocinon OR (syntocinon inj) OR (syntocinon spray) OR tranoxy OR utedrin OR uteracon OR uterason OR (utron inj) OR xitocin OR Oxytocics OR (Oxytocic Drugs) OR (Uterine Stimulants) OR (Oxytocic Agents) OR (Oxytocic Effect) OR (Oxytocic Effects))	
WHO - ICTRP	Condition: (Cesarean Sections) OR (Cesarean Section) OR (Delivery, Abdominal) OR (Abdominal Deliveries) OR (Abdominal Deliver) OR (Deliveries, Abdominal) OR (Abdominal Delivery) OR "C-Section (OB)" OR "C Section (OB)" OR "C-Sections (OB)"<br><br>Intervention: Oxytocin OR Ocytocin OR Syntocinon OR Pitocin OR (Oxytocin Receptor) OR (Receptor, Oxytocin) OR (Oxytocin Receptors) OR (alpha hypophamine) OR "atonin o" OR "beta methyl beta mercaptobutyric acid oxytocin" OR "di sipidin" OR disipidin	95
Total		16,883

For peer review only

Supplementary file 3. Excluded studies in systematic review on route of oxytocin administration

Excluded studies with reasons

Study	*Reason for exclusion*
Zarzur E. A Ocitocina e a Operação Cesariana. Rev Bras Anest. 1992;42(4):293-5.	Did not compare different routes
Garza-Hinojosa A, González-Cordero G. Cambios hemodinámicos y electrocardiográfi cos con el uso de oxitocina en bolo durante la cesárea. Ver Mex Anest. 2015;38(S1): S257-62.	Did not compare different routes
Singh KP, Kameshore N, Kamei H. Prophylactic intramuscular injection of oxytocin vs intravenous infusion of oxytocin to minimize blood loss at caesarean section. Int J Gynecol Obstet. 2015;131(Suppl. 5): E290.	Insufficient information (Abstract-authors did not reply to contact)

For peer review only

Supplementary file 4. Details of trials on different routes of oxytocin administration at CS

Akinaga 2016	
**Method**	Double blind, placebo controlled RCT<br>Study protocol UMIN000007577 (https://upload.umin.ac.jp/cgi-open-bin/ctr_e/ctr_view.cgi?recptno=R000008709)
**Participants**	**Setting**: women managed at Hamamatsu University hospital, Japan<br>**Dates of recruitment**: April to October 2012<br>**Inclusion criteria**: 40 women, ASA status I or II with a single fetus at ≥36 weeks scheduled for elective CS under spinal anesthesia<br>**Exclusion criteria**: conditions predisposing to uterine atony and postpartum hemorrhage, such as placenta previa, multiple gestations, polyhydramnios, previous uterine atony and post-partum bleeding, macrosomia, uterine fibroids, bleeding diathesis, and pre-eclampsia
**Interventions**	After clamping of umbilical cord<br>**Group 1** (intramyometrial): bolus injection of oxytocin (0.07 IU/kg) diluted to 2 mL saline was slowly (over 30 seconds) injected into the myometrium of the uterine fundus and an IV saline infusion (10 mL) was also given.<br>(n=20 randomized)<br>**Group 2**: (intravenous): 2 mL bolus saline injection was injected into the myometrium and an IV oxytocin injection (0.07 IU/kg) diluted to 10 mL with saline was also given.<br>(n=20 randomized)<br>**Both groups:** After bolus injections, both groups received oxytocin at 0.1 IU/kg in 500 mL of lactated Ringer's solution and continuously administered IV at a rate of 100 mL/h.
**Outcomes**	Volume of blood loss during CS (volume in suction bottle). Additional uterotonics. Adverse effects (hypotension, nausea/vomiting, headache, flushing)
**Notes**	Funding: not reported<br>Conflict of interest: none

For peer review only

Dennehy 1998	
**Method**	Double blind, placebo controlled RCT<br>no protocol
**Participants**	**Setting**: women managed in hospital (no other information) in Canada<br>**Dates of recruitment**: No information<br>**Inclusion criteria**: 40 ASA I or II women with singleton fetus scheduled for elective CS at term (> 37 wks).<br>**Exclusion criteria**: age <18 yr, contraindication to spinal anaesthesia, polyhydramnios, placenta praevia, uterine fibroids, hypertension or cardiac disease.
**Interventions**	Immediately after placental removal:<br>**Group 1** (IV bolus): 0.5 ml of 10 IU/ml oxytocin in unknown time and an intramyometrial 2 ml saline injection. Total bolus injection dose: 5 IU, Total bolus injection time: NI.<br>(n=20 randomized)<br>**Group 2** (Intramyometrial bolus): 2 ml of 10 IU/ml oxytocin into the uterine fundus and 0.5 mL saline IV bolus. Total bolus injection dose: 20 IU, Total bolus injection time: NI.<br>(n=20 randomized)
**Outcomes**	Need for additional uterotonics, hypotension
**Notes**	Funding: not reported<br>Conflict of interest: none

For peer review only

Mangla 2012	
**Method**	Double blind RCT<br>No protocol.
**Participants**	**Setting**: Guru Gobind Singh Hospital, Jamnagar, Gujarat, India<br>**Dates of recruitment**: No information<br>**Inclusion criteria:** "women operated by lower segment cesarean section". No other information<br>**Exclusion criteria:** No information
**Interventions**	**Group 1**: IV infusion 20 IU/500 ml Ringer after placental detachment, no information on rate or duration of infusion<br>(n=50 randomized).<br>**Group 2**: Intra-myometrial 5 IU in 10 cc normal saline; 5 cc injected in each cornu of the uterus after separation of placenta.<br>(n=50 randomized)<br>**Group 3**: Intra-myometrial 5 IU in 10 cc normal saline; 5 cc injected in each cornu of the uterus before separation of placenta.<br>(n=50 randomized) This group was not included in the SR because it was identical to Group 2 but given at a different timing
**Outcomes**	Volume of blood loss during CS and 1st hour after (measured by adding volume in graduated suction jar and number of soaked sponges; no information on how blood loss was measured in the 1st hour after).<br>Additional uterotonic. Nausea/vomiting requiring antiemetic.
**Notes**	Funding: none<br>Conflict of interest: none

CS: cesarean section, IU: international units, IV: intravenous, RCT: randomized controlled trial

For peer review only

Supplementary file 5: Postpartum hemorrhage prevention core outcome sets[1] in trials on different routes of oxytocin administration at cesarean section

Outcome		Measure (in each group)	Study		
			Akinaga 2016	Dennehy 1998	Mangla 2012
1	Blood loss (from birth up to cessation of active bleeding)				
	≥ 1000 ml	N women with this loss	no	no	no
	total volume	mean or median volume	yes	no	yes
2	Shock (defined by trialists)	N women	no	no	no
3	Maternal death				
	PPH-related maternal deaths	N women	no	no	no
	all cause mortality	N women	no	no	no
4	Blood transfusion				
	Wm receiving any RBC product	N women	no	no	no
	total volume	mean or median RBC units	no	no	no
5	Transfer to higher level of care	N women	no	no	no
6	Use of additional uterotonics	N women	yes	yes	yes
7	Adverse effects (defined by trialists)	N women	yes (headache, nausea/vomiting, flushing, hypotension)	yes (hypotension)	yes (nausea/ vomiting)
8	Breastfeeding	N women	no	no	no
9	Patient reported outcomes	N women	no	no	no

N: number, PPH: post-partum hemorrhage, RBC: red blood cell

1.Source: Meher S, Cuthbert A, Kirkham JJ, et al. Core outcome sets for prevention and treatment of postpartum haemorrhage: an international Delphi consensus study. BJOG. 2019 Jan;126(1):83-93.

For peer review only

Supplementary file 6. Risk of bias details

Judgments and justifications for risk of bias assessments in trials on different routes of oxytocin administration at cesarean section

Domain	Akinaga 2016		
1	Sequence generation	UNCLEAR	No information on random sequence generation
2	Allocation concealment	LOW	*'An anesthesiologist unrelated to this research selected one of two opaque envelopes containing a letter indicating the group name'*
3	Blinding participants/personnel (objective outcomes)	LOW	*'The subject, care providers, and investigators were blinded to patient allocation until all data collection was completed.'*
3	Blinding participants/personnel (subjective outcomes)	LOW	*'The subject, care providers, and investigators were blinded to patient allocation until all data collection was completed.'*
4	Blinding outcome assessors (objective outcomes)	LOW	*'The i.m.y. group received a bolus injection of oxytocin (0.07 IU/kg) diluted to 2 mL with saline into the myometrium of the uterine fundus during slow (30-s) i.v. infusion of saline (10 mL). Conversely, the i.v. group received a 2-mL bolus injection of saline into the myometrium during slow (30-s) i.v. infusion of oxytocin (0.07 IU/kg) diluted to 10mL with saline.'*
4	Blinding outcome assessors (subjective outcomes)	LOW	*The subject, care providers, and investigators were blinded to patient allocation until all data collection was completed.'*
5	Incomplete outcome	HIGH	*´Nine patients were excluded from the study: eight for protocol deviation and one for adenomyosis.'* High rate of loss (20%), with no information about which was the original group that the participants had been randomized to. ITT was not used.
6	Selective reporting	LOW	All outcomes described in protocol were reported
7	Other bias	LOW	There are probably no other sources of bias

For peer review only

Domain	Dennehy 1998		
1	Sequence generation	LOW	*´Participants were assigned to one of two study groups, according to a computer generated series of random numbers.'*
2	Allocation concealment	UNCLEAR	No information on allocation concealment
3	Blinding participants/personnel (objective outcomes)	LOW	*'The hospital pharmacy prepared the syringes containing the study solutions trader aseptic conditions according to the random table and delivered them to the delivery room in a plastic bag.'*
3	Blinding participants/personnel (subjective outcomes)	does not apply	No subjective outcomes
4	Blinding outcome assessors (objective outcomes)	LOW	*´The injections were conducted in a double blind fashion where neither the attending anaesthetist or obstetrician had knowledge of the content of the syringes.'*
4	Blinding outcome assessors (subjective outcomes)	does not apply	No subjective outcomes
5	Incomplete outcome	LOW	*One patient did not complete the study protocol because the attending anaesthetist was unable to locate the subarachnoid space and she, therefore, received general anaesthesia for Caesarean section*'. Low rate of losses (2%).
6	Selective reporting	UNCLEAR	No study protocol
7	Other bias	LOW	There are probably no other sources of bias

Domain	Mangla 2012		
1	Sequence generation	UNCLEAR	No information on random sequence generation
2	Allocation concealment	UNCLEAR	No information on allocation concealment
3	Blinding participants/personnel (objective outcomes)	LOW	Blinding of participants was unconfirmed. Objective outcomes are unlikely to be influenced by this fact.
3	Blinding participants/personnel (subjective outcomes)	HIGH	Blinding of participants and personnel was unconfirmed. Subjective outcomes are likely to be influenced by this fact.
4	Blinding outcome assessors (objective outcomes)	LOW	Blinding of outcome assessors was unconfirmed. The judgment of objective outcomes is unlikely to be influenced by this fact.
4	Blinding outcome assessors (subjective outcomes)	HIGH	Blinding of participants and personnel was unconfirmed. Subjective outcomes are likely to be influenced by this fact.
5	Incomplete outcome	LOW	The outcome data of all randomized participants were presented
6	Selective reporting	UNCLEAR	No study protocol
7	Other bias	UNCLEAR	No information on baseline characteristics

For peer review only

Supplementary file 6, continuation

Figure S6. Risk of bias graph: review authors' judgements about each risk of bias item presented as percentages across all included studies.

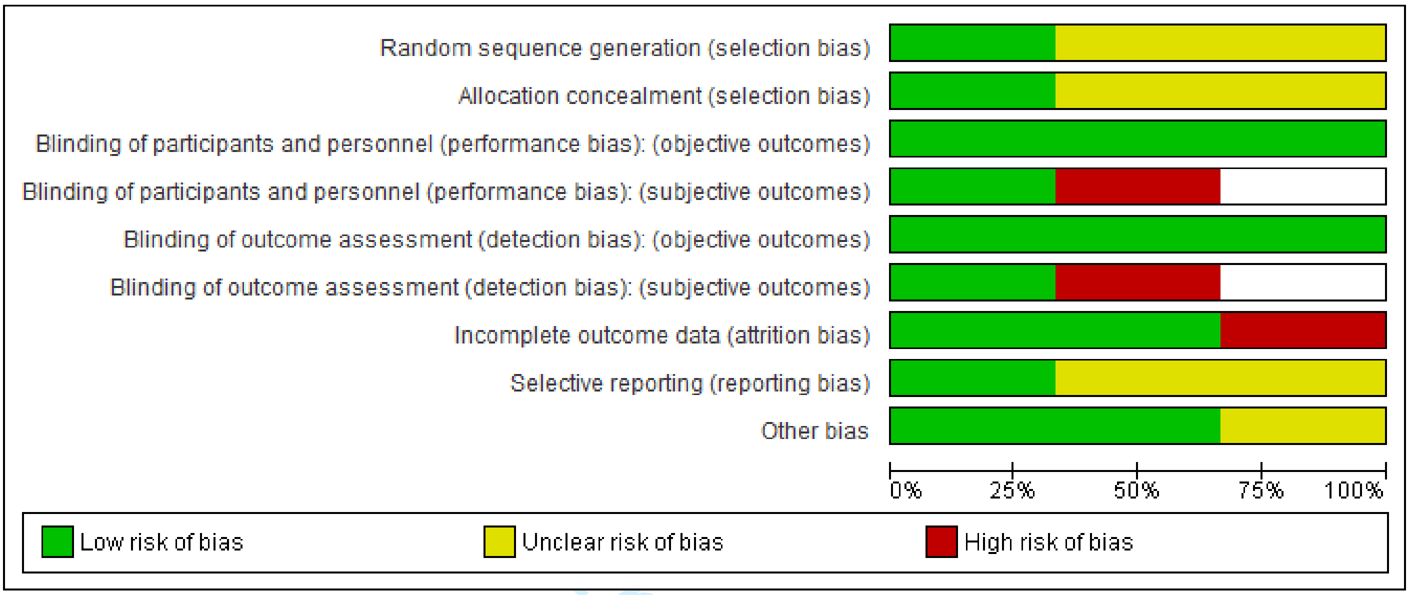

For peer review only

Supplementary file 7. Summary of findings table and GRADE: intramyometrial versus intravenous oxytocin at cesarean section

Intramyometrial compared to Intravenous prophylactic oxytocin for women giving birth by cesarean

Patient or population: women giving birth by cesarean
Setting: hospital
Intervention: intramyometrial oxytocin
Comparison: intravenous oxytocin

Outcomes	Anticipated absolute effects* (95% CI)		Relative effect (95% CI)	N of participants (studies)	Certainty of the evidence (GRADE)	Comments
	**Risk with Intravenous prophylactic oxytocin**	**Risk with Intramyometrial**				
Post-Partum Hemorrhage	60 per 1000	**8 per 1000** (1 to 162)	**RR 0.14** (0.01 to 2.70)	100 (1 RCT)	⊕⊕◯◯ LOW [a]	Mangla 2012: Blood loss > 900 mL.
Need for additional uterotonic	71 per 1000	**59 per 1000** (18 to 192)	**RR 0.82** (0.25 to 2.69)	140 (2 RCTs)	⊕◯◯◯ VERY LOW [a,b]	
Hypotension not requiring epinephrine	200 per 1000	**200 per 1000** (58 to 690)	**RR 1.00** (0.29 to 3.45)	40 (1 RCT)	⊕⊕◯◯ LOW [a]	
Hypotension requiring ephedrine	See comment	See comment	Not estimable	79 (2 RCTs)	⊕⊕◯◯ LOW [c]	The 2 trials had no events.
Nausea and/or Vomiting	157 per 1000	**20 per 1000** (3 to 108)	**RR 0.13** (0.02 to 0.69)	140 (2 RCTs)	⊕⊕◯◯ LOW [d,e]	
Headache	0 per 1000	**0 per 1000** (0 to 0)	**RR 3.00** (0.13 to 69.52)	40 (1 RCT)	⊕⊕◯◯ LOW [a]	
Facial flushing	100 per 1000	**50 per 1000** (5 to 508)	**RR 0.50** (0.05 to 5.08)	40 (1 RCT)	⊕⊕◯◯ LOW [a]	
Volume of blood loss	The mean volume of blood loss was **664.2** mL	MD **57.40 mL lower** (101.71 lower to 13.09 lower)	-	40 (1 RCT)	⊕⊕◯◯ LOW [f]	

***The risk in the intervention group** (and its 95% confidence interval) is based on the assumed risk in the comparison group and the **relative effect** of the intervention (and its 95% CI).
CI: Confidence interval; **RR:** Risk ratio; **MD:** Mean difference

GRADE Working Group grades of evidence
High certainty: We are very confident that the true effect lies close to that of the estimate of the effect
Moderate certainty: We are moderately confident in the effect estimate: The true effect is likely to be close to the estimate of the effect, but there is a possibility that it is substantially different
Low certainty: Our confidence in the effect estimate is limited: The true effect may be substantially different from the estimate of the effect
Very low certainty: We have very little confidence in the effect estimate: The true effect is likely to be substantially different from the estimate of effect

Explanations

a. Evidence certainty downgraded -2 due to very serious imprecision (very small number of events, and wide 95% CI crossing the line of no effect).
b. Evidence certainty downgraded -1 due to inconsistency (I2 61%)
c. Evidence certainty downgraded -2 due to very serious imprecision (lack of events).
d. Evidence certainty downgraded -1 due to study limitation (lack of blinding of participants, subjective outcome)
e. Evidence certainty downgraded -1 due to serious imprecision (small sample size)
f. Evidence certainty downgraded -2 due to very serious imprecision (small sample size and wide 95% confidence interval).

PRISMA 2009 Checklist

Section/topic	#	Checklist item	Reported on page #
**TITLE**			
Title	1	Identify the report as a systematic review, meta-analysis, or both.	1
**ABSTRACT**			
Structured summary	2	Provide a structured summary including, as applicable: background; objectives; data sources; study eligibility criteria, participants, and interventions; study appraisal and synthesis methods; results; limitations; conclusions and implications of key findings; systematic review registration number.	2
**INTRODUCTION**			
Rationale	3	Describe the rationale for the review in the context of what is already known.	3-4
Objectives	4	Provide an explicit statement of questions being addressed with reference to participants, interventions, comparisons, outcomes, and study design (PICOS).	4
**METHODS**			
Protocol and registration	5	Indicate if a review protocol exists, if and where it can be accessed (e.g., Web address), and, if available, provide registration information including registration number.	4, File S1
Eligibility criteria	6	Specify study characteristics (e.g., PICOS, length of follow-up) and report characteristics (e.g., years considered, language, publication status) used as criteria for eligibility, giving rationale.	4-5
Information sources	7	Describe all information sources (e.g., databases with dates of coverage, contact with study authors to identify additional studies) in the search and date last searched.	5
Search	8	Present full electronic search strategy for at least one database, including any limits used, such that it could be repeated.	File S2
Study selection	9	State the process for selecting studies (i.e., screening, eligibility, included in systematic review, and, if applicable, included in the meta-analysis).	5-6, Fig 1
Data collection process	10	Describe method of data extraction from reports (e.g., piloted forms, independently, in duplicate) and any processes for obtaining and confirming data from investigators.	5
Data items	11	List and define all variables for which data were sought (e.g., PICOS, funding sources) and any assumptions and simplifications made.	6, File S5
Risk of bias in individual studies	12	Describe methods used for assessing risk of bias of individual studies (including specification of whether this was done at the study or outcome level), and how this information is to be used in any data synthesis.	5-6
Summary measures	13	State the principal summary measures (e.g., risk ratio, difference in means).	6
Synthesis of results	14	Describe the methods of handling data and combining results of studies, if done, including measures of consistency (e.g., I^2) for each meta-analysis.	6

For peer review only - http://bmjopen.bmj.com/site/about/guidelines.xhtml

PRISMA 2009 Checklist

Section/topic	#	Checklist item	Reported on page #
Risk of bias across studies	15	Specify any assessment of risk of bias that may affect the cumulative evidence (e.g., publication bias, selective reporting within studies).	6
Additional analyses	16	Describe methods of additional analyses (e.g., sensitivity or subgroup analyses, meta-regression), if done, indicating which were pre-specified.	6
**RESULTS**			
Study selection	17	Give numbers of studies screened, assessed for eligibility, and included in the review, with reasons for exclusions at each stage, ideally with a flow diagram.	6-7 Fig 1, File S3
Study characteristics	18	For each study, present characteristics for which data were extracted (e.g., study size, PICOS, follow-up period) and provide the citations.	6-8, Table 1, File S4
Risk of bias within studies	19	Present data on risk of bias of each study and, if available, any outcome level assessment (see item 12).	9, Fig 2, File S6
Results of individual studies	20	For all outcomes considered (benefits or harms), present, for each study: (a) simple summary data for each intervention group (b) effect estimates and confidence intervals, ideally with a forest plot.	9
Synthesis of results	21	Present results of each meta-analysis done, including confidence intervals and measures of consistency.	9-10, Table 2,, Figs 3,4
Risk of bias across studies	22	Present results of any assessment of risk of bias across studies (see Item 15).	File S6
Additional analysis	23	Give results of additional analyses, if done (e.g., sensitivity or subgroup analyses, meta-regression [see Item 16]).	Not applicable
**DISCUSSION**			
Summary of evidence	24	Summarize the main findings including the strength of evidence for each main outcome; consider their relevance to key groups (e.g., healthcare providers, users, and policy makers).	10-11
Limitations	25	Discuss limitations at study and outcome level (e.g., risk of bias), and at review-level (e.g., incomplete retrieval of identified research, reporting bias).	11
Conclusions	26	Provide a general interpretation of the results in the context of other evidence, and implications for future research.	12
**FUNDING**			
Funding	27	Describe sources of funding for the systematic review and other support (e.g., supply of data); role of funders for the systematic review.	12

From: Moher D, Liberati A, Tetzlaff J, Altman DG, The PRISMA Group (2009). Preferred Reporting Items for Systematic Reviews and Meta-Analyses: The PRISMA Statement. PLoS Med 6(7): e1000097. doi:10.1371/journal.pmed1000097

For peer review only

BMJ Open

Route of oxytocin administration for preventing blood loss at caesarean section: a systematic review with meta-analysis

Journal:	*BMJ Open*
Manuscript ID	bmjopen-2021-051793.R2
Article Type:	Original research
Date Submitted by the Author:	16-Aug-2021
Complete List of Authors:	Torloni, Maria Regina; Hospital e Maternidade Santa Joana, ; Universidade Federal de Sao Paulo Escola Paulista de Medicina, Evidence-Based Healthcare Post-Graduate Program<br>Siaulys, Monica; Hospital e Maternidade Santa Joana, Anaesthesiology Department<br>Riera, Rachel; Hospital Sirio-Libanes, Centre of Health Technology Assessment; Universidade Federal de Sao Paulo Escola Paulista de Medicina, Evidence Based Healthcare Post-Graduation Program<br>Martimbianco, Ana ; Universidade Metropolitana de Santos; Universidade Federal de Sao Paulo Escola Paulista de Medicina, Evidence Based Healthcare Post-Graduate program<br>Pacheco, Rafael; Centro Universitário São Camilo; Universidade Federal de Sao Paulo Escola Paulista de Medicina, Evidence Based Healthcare Post-Graduation Program<br>Latorraca, Carolina ; Universidade Federal de Sao Paulo Escola Paulista de Medicina, Evidence Based Healthcare Post-Graduate program<br>Widmer, Mariana; World Health Organization, Reproductive Health and Research<br>Betran, Ana Pilar; World Health Organization, Reproductive Health and Research
**Primary Subject Heading**:	Obstetrics and gynaecology
Secondary Subject Heading:	Pharmacology and therapeutics, Surgery
Keywords:	Maternal medicine < OBSTETRICS, THERAPEUTICS, Adverse events < THERAPEUTICS, Adult surgery < SURGERY

SCHOLARONE™
Manuscripts

I, the Submitting Author has the right to grant and does grant on behalf of all authors of the Work (as defined in the below author licence), an exclusive licence and/or a non-exclusive licence for contributions from authors who are: i) UK Crown employees; ii) where BMJ has agreed a CC-BY licence shall apply, and/or iii) in accordance with the terms applicable for US Federal Government officers or employees acting as part of their official duties; on a worldwide, perpetual, irrevocable, royalty-free basis to BMJ Publishing Group Ltd ("BMJ") its licensees and where the relevant Journal is co-owned by BMJ to the co-owners of the Journal, to publish the Work in this journal and any other BMJ products and to exploit all rights, as set out in our licence.

The Submitting Author accepts and understands that any supply made under these terms is made by BMJ to the Submitting Author unless you are acting as an employee on behalf of your employer or a postgraduate student of an affiliated institution which is paying any applicable article publishing charge ("APC") for Open Access articles. Where the Submitting Author wishes to make the Work available on an Open Access basis (and intends to pay the relevant APC), the terms of reuse of such Open Access shall be governed by a Creative Commons licence – details of these licences and which Creative Commons licence will apply to this Work are set out in our licence referred to above.

Other than as permitted in any relevant BMJ Author's Self Archiving Policies, I confirm this Work has not been accepted for publication elsewhere, is not being considered for publication elsewhere and does not duplicate material already published. I confirm all authors consent to publication of this Work and authorise the granting of this licence.

For peer review only

Route of oxytocin administration for preventing blood loss at caesarean section: a systematic review with meta-analysis

Maria Regina Torloni[1,2], Monica Siaulys[1], Rachel Riera[2,3], Ana Luiza Cabrera Martimbianco[2,4], Rafael Leite Pacheco[2,5], Carolina de Oliveira Cruz Latorraca[2], Mariana Widmer[6], Ana Pilar Betrán[6].

1. Hospital e Maternidade Santa Joana
Rua Dr. Eduardo Amaro 225, São Paulo, SP, CEP 04104-080, Brazil.

2. Evidence Based Healthcare Post-Graduation Program, São Paulo Federal University-UNIFESP, Rua Botucatu 740, 5º andar, São Paulo, SP, CEP 04023-900, Brazil

3. Centre of Health Technology Assessment, Hospital Sirio-Libanês, Rua Dona Adma Jafet, 91, São Paulo, SP, CEP 01308-050, Brazil

4. Universidade Metropolitana de Santos (UNIMES)
Av. Gen. Francisco Glicério 8, Santos, SP, CEP 11045-002, Brazil

5. Centro Universitário São Camilo
Av. Nazaré, 1501, São Paulo, SP, CEP 04263-200, Brazil

6. Department of Reproductive Health and Research, World Health Organization, UNDP/UNFPA/UNICEF/WHO/World Bank Special Programme of Research, Development and Research Training in Human Reproduction (HRP), Avenue Appia 20, 1201 Geneva, Switzerland.

Corresponding author:
Maria Regina Torloni, email: torlonimr@gmail.com

Word count: 4073

For peer review only

Abstract

Objectives: Assess the effects of different routes of prophylactic oxytocin administration for preventing blood loss at caesarean section (CS). **Design:** Systematic review and meta-analysis. **Methods.** Medline, EMBASE, CINAHL, Cochrane Library, BVS, SciELO, and Global Index Medicus were searched through 24/05/2020 for randomised controlled trials (RCT) comparing different routes of prophylactic oxytocin administration during CS. Study selection, data extraction, and quality assessment were conducted by two investigators independently. We pooled results in fixed effects meta-analyses and calculated average risk ratio (RR), mean difference (MD), and 95% confidence intervals (CI). We used GRADE to assess the overall quality of evidence for each outcome. **Results:** Three trials (180 women) were included in the review. All studies compared intramyometrial (IMY) versus intravenous (IV) oxytocin in women having pre-labour CS. IMY compared to IV oxytocin administration may result in little or no difference in the incidence of post-partum haemorrhage (RR 0.14, 95% CI 0.01 to 2.70; N=100 participants; 1 RCT), hypotension (RR 1.00, 95% CI 0.29 to 3.45; N=40; 1 RCT), headache (RR 3.00, 95% CI 0.13 to 69.52; N=40; 1 RCT) or facial flushing (RR 0.50, 95% CI 0.05 to 5.08; N=40; 1 RCT); IMY oxytocin may reduce nausea/vomiting (RR 0.13, 95% CI 0.02 to 0.69; N=140; 2 RCTs). We are very uncertain about the effect IMY versus IV oxytocin on the need for additional uterotonics (RR 0.82; 95% CI 0.25 to 2.69; N=140; 2 RCTs). IMY oxytocin may reduce blood loss slightly (MD -57.40 mL, 95% CI -101.71 to -13.09; N=40; 1 RCT). **Conclusions:** There is limited, low to very low certainty evidence on the effects of IMY versus IV oxytocin at CS for preventing blood loss. The evidence is insufficient to support choosing one route over another. More trials, including studies that assess intramuscular oxytocin administration, are needed on this relevant question.

Prospero: CRD42020186797

Keywords: Postpartum Hemorrhage, Cesarean section, Oxytocin, Drug Administration Routes, Systematic Review, Meta-Analysis

For peer review only

Article summary: Strengths and Limitations

- This is the first systematic review to assess the effects of different routes of oxytocin administration for preventing bleeding in women giving birth by caesarean.
- We performed an extensive search without language restrictions in 10 databases to identify relevant trials, and followed strict methodology throughout the systematic review process.
- We found few trials, involving a small number of participants, and addressing a limited number of outcomes.
- The trials did not test all possible routes of oxytocin administration, and only included pre-labour caesareans at term.
- The overall certainty of the evidence was low or very low for all outcomes due mainly to imprecision.

For peer review only

INTRODUCTION

Deliveries by caesarean section (CS) have increased worldwide over the last three decades, including in low- and middle-income countries (LMIC)[1]. Even in optimal conditions, women who have a CS lose more blood, and have a higher risk for post-partum haemorrhage (PPH) than those who give birth by vaginal delivery (VD)[2 3]. PPH is an important cause of maternal morbidity, including prolonged hospital stay, blood transfusion and hysterectomy, and it is the leading cause of maternal mortality worldwide[4-6]. Uterine atony is responsible for 50% to 80% of all cases of PPH[4 6].

Routine administration of prophylactic uterotonics soon after birth reduces the incidence of PPH and its associated complications[6]. Oxytocin remains the first-line agent for preventing PPH recommended by the World Health Organization (WHO) because of its lack of major contraindications, similar efficacy, and lower cost and incidence of side effects, compared to other uterotonics[6]. For women having a VD, a systematic review in 2020 estimated that intravenous (IV) oxytocin is more effective than intramuscular (IM) administration to reduce PPH, severe PPH, blood transfusion, and severe maternal morbidity, with no clear differences in adverse events[7]. Based on these findings, WHO updated its guideline in 2020 and now recommends that women giving birth by VD who already have endovenous access should receive a slow IV injection of 10 IU of oxytocin, in preference to IM administration[8]. For women giving birth by CS, due to the lack of specific evidence, WHO currently recommends 10 IU of oxytocin without preference for the IV or IM routes[8]. Other guidelines provide little, unclear, or no recommendations on the route of administration of prophylactic oxytocin at CS[9-11].

Since all women having a CS will have an endovenous access, IV administration of oxytocin seems logical. However, due to concerns about the cardiovascular side-effects of the drug in women undergoing a major abdominal surgery, many studies have investigated different dosages and regimens of IV oxytocin administration at CS in search of the optimal method to safely prescribe this uterotonic[12-17]. After IV injection in late pregnancy, oxytocin has an almost immediate onset of action but a half-life of only 5-12 minutes due to its rapid hepatic and renal degradation[17-19]. With continued infusion, oxytocin reaches a steady state plateau in the plasma in approximately 40 minutes[17-19]. Activation of oxytocin receptors in the myometrium stimulates prostaglandin synthesis and increases intracellular calcium in myocytes which leads to strong uterine contractions thus reducing blood loss from the placental site[16-19]. However, the simultaneous activation of oxytocin receptors in the cardiovascular system causes decreased systemic vascular resistance, hypotension, tachycardia, and coronary vasoconstriction with the attendant symptoms of skin flushing, headache, nausea, vomiting, and chest pain[17 20 21]. These changes are usually transient, depend on the dose and speed of IV oxytocin

For peer review only - http://bmjopen.bmj.com/site/about/guidelines.xhtml

administration, and are tolerated by most healthy young women[16 22]. However, in some women undergoing a CS, these haemodynamic changes can be potentially dangerous and even life-threatening because of the sympathetic nervous blockade secondary to regional anaesthesia, the concomitant use of other vasoactive drugs, excessive blood loss, and the underlying clinical and obstetric disorders that may have led to the CS indication (e.g. cardiac disease, hypertensive disorders, placenta previa)[21 23-25]. Due to its antidiuretic properties, oxytocin administration can also lead to water intoxication resulting in pulmonary oedema, seizures, coma and even death, especially in women who receive large volumes of fluid[13 17-19]. The routine use of large IV fluid preloads during CS under regional anaesthesia can potentially increase the risk of this complication[26].

After IM oxytocin injection, uterine response occurs within 3-5 minutes and can last for up to 2-3 hours[8 18 19 27]. Direct intramyometrial (IMY) injection of uterotonics can also promote uterine contraction[28 29]. IMY oxytocin injection is believed to promote immediate uterine contraction by local effect and by drug absorption from the myometrium into the systemic circulation[28 30-32]. Although oxytocin is not licensed for IMY use[18 19], this route of administration has been tested for preventing PPH at CS since 1990[28 30-32].

The route of administration of prophylactic oxytocin at CS can potentially affect the volume of maternal blood loss as well as the incidence of drug-related adverse effects. Since the side-effects of oxytocin are directly related to dose and rate of administration[13 17-19 27], in theory, the IM or IMY routes could lead to fewer cardiovascular side-effects than the IV route. If the efficacy of IM and IMY prophylactic oxytocin during CS is similar to IV administration, and if these routes have fewer side-effects than the endovenous route, this could challenge the preference for IV administration. Several randomised trials have compared different routes of prophylactic oxytocin administration at CS, but there are no systematic reviews on this topic. It is important to assess the balance between the effectiveness and adverse effects of the various routes of prophylactic oxytocin administration at CS because this information is essential to underpin evidence-based recommendations on the prevention of PPH in women given birth by CS.

OBJECTIVES

To assess the effects of different routes of prophylactic oxytocin administration for preventing blood loss at CS.

METHODS

For peer review only - http://bmjopen.bmj.com/site/about/guidelines.xhtml

We conducted this review following Cochrane methods[33] and reported it according to the PRISMA statement[34]. We registered the protocol prospectively (CRD42020186797- Supplementary file 1).

Patient and public involvement

There was no patient or public involvement in this study.

Types of studies

We included randomised clinical trials with a parallel design. Abstracts were eligible if they provided sufficient outcome data and methodological details for quality assessment.

Types of participants

Study participants were women having a primary or repeat CS, before or during labour, at any gestational age and for any indication, regardless of previous exposure to oxytocin (for labour induction or augmentation). Studies with women at high or low baseline risk for PPH, with or without comorbidities, and with singleton or multiple pregnancies were eligible for inclusion. We included studies involving women with both routes of delivery only if authors provided separate data for those giving birth by CS.

Types of interventions

We included studies that compared different routes of oxytocin administration (IV, IM or IMY) given alone, at any moment during CS (pre-delivery, after delivery, or after placental extraction). We excluded studies that administered oxytocin associated with other drugs for preventing PPH.

Outcomes

Studies that reported at least one of our outcomes of interest were included. Our outcomes were based on the PPH prevention core outcome set created by the CROWN initiative[35]. The primary outcomes were PPH $\geq$ 1000 mL (measured objectively or subjectively), use of additional uterotonics, and any immediate adverse effects possibly related to oxytocin (e.g. nausea/vomiting, facial flushing, headache, hypotension, cardiac arrhythmia). The secondary outcomes were total volume of blood loss at CS, need for transfusion, maternal transfer to higher level of care, shock, severe maternal morbidity (admission to ICU, hysterectomy, coma or organ failure), maternal mortality related to PPH, breastfeeding at hospital discharge, and maternal satisfaction.

Search strategy

For peer review only

We created a search strategy with the following general terms and synonyms: “caesarean section” or “C-section” or “abdominal delivery” and “oxytocin” (details in Supplementary file 2). The search strategy was developed by an experienced information specialist. It was adapted and run in seven electronic databases (MEDLINE, Embase, Cochrane Library, CINAHL, BVS, Global Index Medicus, SciELO) and two trial registry platforms (ClinicalTrials.gov and WHO – ICTRP) (Supplementary file 2). We searched for unpublished studies (grey literature) in Opengrey (https://opengrey.eu). We screened the reference lists of all retrieved studies and relevant systematic reviews to identify additional potentially eligible trials not captured by the electronic searches. There were no language or publication status restrictions. Searches were run from database inception until May 24, 2020.

Process of study selection, data extraction and risk of bias assessment

All references were uploaded in the Rayyan platform[36] and duplicates were excluded. All titles and abstracts were screened independently by two review authors. Potentially eligible studies were selected for full text reading; those that fulfilled the selection criteria were included in the review. Two review authors extracted data independently and a third author check data for accuracy. Two review authors independently assessed the quality of included studies using the Cochrane Risk of Bias tool[33]. In each study, authors assessed and graded seven domains as being at high, low, or unclear risk of bias. Four domains (random sequence generation, allocation concealment, selective reporting, and other source of bias) were assessed at study level; three domains (blinding of participants and personnel, blinding of outcome assessment, and incomplete outcome data) were assessed at outcome-level. Any disagreements in the process of study selection, data extraction, or risk of bias assessment were solved by a third senior review author.

Data analyses and assessment of the certainty of the evidence

We planned to conduct three comparisons between routes of oxytocin administration: IV vs IM, IV vs IMY, and IM vs IMY. All analyses were conducted on an intention-to-treat basis. When data were missing, we contacted trial authors. Where meta-analyses were not deemed suitable, we report results descriptively. Similar study data were pooled using the software Review Manager 5.4 (The Cochrane Collaboration, 2020). For dichotomous outcomes, we calculated risk ratio (RR) and 95% confidence intervals (CI); for continuous data, we calculated mean differences (MD) and 95% CI. We pooled data using the Mantel-Haenszel fixed-effect method; $I^2 \geq 50\%$ was considered an indication of high statistical heterogeneity. We planned to conduct pre-specified subgroup analyses for the primary outcomes (when data were available) according to parity (nullipara vs multipara, and multipara with CS vs multipara without previous CS), type of CS (pre-

For peer review only

labour vs intrapartum), previous use of oxytocin in labour, and baseline risk for PPH. We could not conduct these analyses because of the small number of trials included and lack of information. We planned to conduct sensitivity analyses for the main comparisons restricted only to high-quality studies (at low risk of bias for random sequence generation and allocation concealment), and restricted only to studies that assessed blood loss using an objective measure. These analyses were not done because all studies measured blood loss objectively and none were high-quality. Due to the small number of trials, we could not assess publication bias.

We assessed the certainty (quality) of the body of evidence for selected outcomes using the GRADE approach[37]. Evidence was categorized as being of high, moderate, low, or very low quality. We downgraded the quality of the evidence one or two levels because of trial limitations, inconsistency, indirectness, imprecision, and publication bias[37]. This process was conducted by two independent investigators; disagreements were solved by a third senior investigator.

RESULTS

A total of 13,389 unique references were identified. After title and abstract screening, six citations were selected for full text reading. Two studies and one abstract were excluded (Supplementary file 3) and three trials[30-32] were included in the review (Figure 1).

The three trials included a total of 180 women and were conducted between 1998 and 2012, in Japan[30], Canada[31] , and India[32] (Table 1 and Supplementary file 4). None of the trials provided clear information on the proportion of nulliparas or multiparas (with and without previous CS). One trial[32] did not report participants´ baseline risk for PPH, gestational age, or type of CS; the other two trials[30 31] included participants with regular/low baseline risk for PPH, undergoing a pre-labour CS with at least 36 weeks´ gestation, under spinal anaesthesia (Table 1). All three trials compared IV vs IMY oxytocin administration after foetal delivery. There was heterogeneity between studies on the oxytocin doses used in each route; the IMY dose ranged from 4.3 IU[30] to 20 IU[31], and the total IV dose ranged from 4.3 IU[30] to 20 IU[32]. One trial[30] administered oxytocin with the placenta still in place, immediately after umbilical cord clamping, while the other two studies waited for placental detachment or removal before giving the oxytocin[31 32]. Akinaga et al randomised 40 Japanese women to receive either an IMY oxytocin injection and an IV saline bolus injection, or an IV oxytocin bolus and an IMY saline injection immediately after umbilical cord clamping; all women also received a slow oxytocin maintenance infusion for the next five hours[30]. Dennehy et al randomised 40 women in Canada to

receive an IMY oxytocin bolus injection into the uterine fundus and a saline IV bolus injection, or an IV oxytocin bolus injection and a saline IMY injection after placental removal[31]. Mangla et al randomised 150 women in India to start a 500 mL infusion containing oxytocin after placental separation (Group 1, N=50), or to receive an IMY oxytocin bolus injection (half in each uterine cornus) either after placental separation (Group 2, N=50) or before placental separation (Group 3, N=50) [32]. We did not include in this systematic review the participants in the third group because it compared a different route and a different timing of oxytocin administration (Supplementary file 4). The two trials that reported the volume of blood loss [30 32] used objective methods of assessment, but only one[32] assessed the incidence of blood loss > 900 mL. All trials assessed the need for additional uterotonics and at least one adverse effect of oxytocin. No trial reported any of our other secondary outcomes (Table 1 and Supplementary file 5).

For peer review only

Table 1. Main characteristics of trials on routes of oxytocin administration at caesarean section

Characteristics	Akinaga 2016 (ref 30)	Dennehy 1998 (ref 31)	Mangla 2012 (ref 32)
Setting	Japan, one university hospital	Canada, no information on type of hospital(s)	India, one hospital
Design	Double blind, placebo controlled RCT	Double blind, placebo controlled RCT	RCT
Sample size	40	40	100[1]
Gestational age	all $\geq$ 36 weeks	all $\geq$ 37 weeks	no information
Baseline risk for PPH	all low risk	all low risk	no information
Parity	unclear	no information	no information
Participants with previous CS	unclear	no information	no information
Type of CS	Pre-labour, scheduled	Pre-labour, scheduled	no information
Previous exposure to oxytocin	No	No	no information
Anaesthesia	spinal	spinal	spinal or general
Timing of administration	After umbilical cord clamping	After placental removal	After placental separation
Oxytocin routes and regimen	IMY bolus vs IV bolus followed by IV maintenance infusion	IMY bolus vs IV bolus	IMY bolus vs IV infusion
Oxytocin dose, diluent, speed of administration, (dose/min), Total dose	IMY bolus: 4.3 IU[2] in 2 mL 0.9% saline over 30 sec in uterine fundus.<br>Total dose: 4.3 IU<br><br>IV bolus: 4.3 IU[2] in 10 ml 0.9% saline over 30 sec (8.5 IU/min)<br>Total dose: 4.3 IU<br><br>Maintenance infusion (both groups): 6.1 IU[3] in 500 ml Ringer at 100 ml/h for 5 h (0.02 IU/min)<br>Total dose: 6.1 IU<br><br>Total oxytocin regimen dose[4]: 10.4 IU	IMY bolus: 2 ml of 10 IU/ml in uterine fundus.<br>Total dose: 20 IU<br><br>IV bolus: 0.5 ml of 10 IU/ml over unknown time<br>Total dose: 5 IU	IMY bolus: 5 IU in 10 ml 0.9% saline (5 ml in each uterine cornu).<br>Total dose: 5 IU<br><br>IV infusion 20 IU in 500 ml Ringer, no information on speed or duration<br>Total dose: 20 IU
Outcomes reported	Volume of blood loss, Additional uterotonics, Adverse effects	Additional uterotonics, Adverse effects	Volume of blood loss, Additional uterotonics, Adverse effects

CS: caesarean section, IU: international units, IV: intravenous, IMY: Intramyometrial, min: minutes, ml: millilitre, PPH: post-partum haemorrhage, RCT: randomised controlled trial, Ref: reference, Sec: seconds.
1. Mangla 2012 had a third group with 50 women (total sample: 150) that was not included in this review because it administered IMY before placental separation (i.e. different timing and route from IV group).
2. Bolus dose was calculated based on the mean weight of participants (0.07 IU/Kg x 61 Kg)

For peer review only

3. Maintenance infusion dose was calculated based on the mean weight of participants (0.01 IU/Kg x 61 Kg).
4. Total oxytocin dose received by all participants (bolus + maintenance infusion)

Figure 2 presents the risk of bias of the three included studies (see Supplementary file 6 for details and summary graph). All studies had at least one domain with unclear or high risk of bias. Akinaga 2016[30] and Mangla 2012[32] did not describe the method used to generate the randomization sequence; Dennehy 1998[31] and Mangla 2012[32] did not describe how allocation concealment was achieved. Overall, the three trials had an unclear risk of bias for random sequence generation or allocation concealment, or both domains. We contacted the authors of the three trials to obtain additional details, but none replied.

Table 2 presents a summary of all outcomes for the comparison between prophylactic IMY versus IV oxytocin in women having a CS. See supplementary file 7 for the assessment of the certainty of the evidence (GRADE) for each outcome.

For peer review only

Table 2. Comparison of prophylactic intramyometrial versus intravenous oxytocin for preventing blood loss in women giving birth by caesarean

Outcome or subgroup title	N studies	N women	Effect measure (IMY vs IV)	Effect size (95% CI)	Certainty of the evidence (GRADE)[3]
1. PPH > 1000 mL	1	100	Risk Ratio	0.14 (0.01, 2.70)	Low
2. Use of additional uterotonics	2	140	Risk Ratio	0.82 (0.25, 2.69)[1]	Very low
3. Hypotension not requiring ephedrine	1	40	Risk Ratio	1.00 (0.29, 3.45)	Low
4. Hypotension requiring ephedrine	2	79	Risk Ratio	Not estimable	Low
5. Nausea and/or vomiting	2	140	Risk Ratio	0.13 (0.02, 0.69) [1]	Low
6. Headache	1	40	Risk Ratio	3.00 (0.13, 69.52)	Low
7. Facial flushing	1	40	Risk Ratio	0.50 (0.05, 5.08)	Low
8. Mean blood loss (mL)	1[2]	40	Mean Difference	-57.40 (-101.71, -13.09)	Low

CI: Confidence interval, IMY: Intramyometrial, N=number, PPH: Post-partum haemorrhage, vs: versus.
1. Mantel-Haenszel fixed-effect meta-analysis.
2. Two trials reported mean blood loss, but one (Mangla 2012) could not be included in effect size estimates because it did not provide standard deviation or CI.
3. See Supplementary file 7 for details.

For peer review only

Incidence of PPH

None of the studies assessed PPH according to the standard definition (≥ 1000 ml). However, Mangla 2012[32] reported that none of the women who received IMY oxytocin had a blood loss > 900 ml compared to 6% of those who received IV oxytocin (0/50 versus 3/50, RR 0.14, 95% CI 0.01 to 2.70; 100 participants; 1 RCT, low certainty evidence). (Table 2).

Need for additional uterotonics

The pooled estimate from the three trials was very imprecise and compatible with an important risk reduction or increase in the need for additional uterotonic with IMY compared to IV oxytocin injection (4/70 versus 5/70, respectively; RR 0.82; 95% CI 0.25 to 2.69; 140 participants; 2 RCTs; I^2=61%, very low certainty evidence) (Figure 3, Table 2)

Adverse effects of oxytocin

Hypotension: Data from one trial[30] indicates that IMY compared to IV oxytocin administration may have no effect on the incidence of isolated hypotension episodes that do not require ephedrine (RR: 1.00, 95% CI 0.29 to 3.45; 40 participants; 1 RCT; low certainty evidence). Two trials[30 31] (79 participants) assessed the incidence of persistent hypotension requiring treatment with epinephrine, but there were no events among the women who received IMY or IV oxytocin. Although the lack of events did not allow estimation of the risk ratio, the evidence from these trials is consistent with absence of a difference between IMY and IV for this outcome. (Table 2).

Changes in heart rate: We could not perform a meta-analysis for this outcome. Akinaga[30] reported no apparent changes in the heart rate (HR) of the 20 women who received IMY oxytocin while the HR of the IV group started to increase soon after the bolus injection, reaching a maximum value 75 seconds after. The maximum HR in the IV group was significantly higher than in the IMY group (92.9 ± 10.3 vs 83.2 ± 7.2 beats per minute, mean difference: 9.7, 95% CI 4.0 to 15.4, p=0.002). On the other hand, Dennehy reported a significantly higher increase in HR one minute after IMY than after IV oxytocin administration, but the authors did not provide numerical data for this outcome[31].It should be noted that while Akinaga[30] gave the same dose of oxytocin (4.3 IU) in both routes, the IMY oxytocin dose given by Dennehy[31] was four times higher than the IV dose (20 IU vs 5 IU, respectively).

Nausea and/or vomiting: The pooled estimate of two trials including 140 women showed a lower average risk of nausea and/or vomiting in the group that received

For peer review only

IMY oxytocin (RR 0.14; 95% CI 0.03 to 0.78; 140 participants; 2 RCTs; I^2=0%, low certainty evidence) (Figure 4, Table 2).

Headache and facial flushing: Only Akinaga[30] reported the incidence of headache and facial flushing**.** One woman in the IMY group complained of headache versus none in the IV group (1/20 versus 0/20, RR 3.00, 95% CI 0.13 to 69.52; 40 participants; 1 RCT; low certainty evidence). One participant in the IMY and two in the IV oxytocin group had facial flushing (1/20 versus 2/20, RR 0.50, 95% CI 0.05 to 5.08; 40 participants; 1 RCT; low certainty evidence). (Table 2).

Volume of blood loss

Mangla[32] reported that the women in the IMY group had a lower mean volume of blood loss than those in the IV group (460 mL versus 606 mL, respectively), but did not provide the standard deviations (SD) or confidence intervals for this measure. Akinaga[30] also reported a slightly lower mean volume of blood loss in the IMY than in the IV group (606.8 ± 68.8 versus 664.2 ± 74.1 mL; MD -57.40 mL; 95% CI -101.71 to -13.09; 40 participants; 1 RCT; low certainty evidence) (Table 2). We could not pool the results of the two trials because one of them[32] did not provide information on SD and the authors did not reply to our emails.

DISCUSSION

Main Findings and Interpretation

Our extensive search identified only three trials on the effects of different routes of administration of prophylactic oxytocin in women giving birth by CS. The existing evidence suggests that IMY compared to IV oxytocin administration may result in little to no difference on the incidence of PPH (≥ 1000 mL) and most side effects (hypotension, headache, or facial flushing), but IMY oxytocin may reduce the incidence of nausea/vomiting. The evidence also suggests that IMY oxytocin may reduce the volume of blood loss slightly. We are very uncertain about the effects of IMY versus IV oxytocin on the need for additional uterotonics. The overall certainty of the evidence was low or very low for all outcomes due mainly to imprecision. According to the GRADE approach, this means that we have limited confidence in the effect estimates and that the true effect may be substantially different from the estimates presented.[37]

Where information was available, most trials recruited only healthy women with a low baseline risk for PPH, who were having an elective pre-labour CS under spinal anaesthesia to deliver a single foetus at term. Information about parity, including the percentage of participants with one or more previous CS, was unclear or missing in

For peer review only - http://bmjopen.bmj.com/site/about/guidelines.xhtml

all studies. Therefore, we cannot infer that the results of this systematic review apply to women with other characteristics, such as preterm or multiple pregnancies, women having intra-partum CS, or those with previous exposure to oxytocin for labour induction or augmentation. The different doses and regimen of oxytocin used in the trials can also have affected the results because oxytocin plasma levels are dose dependent.[38 39]

It is surprising that we did not find any studies comparing IM versus IV prophylactic oxytocin at CS, since these are the more frequently used routes of administration, and there are many trials on this comparison for women giving birth vaginally[7]. While the slower onset of action of oxytocin after IM administration[18 19] can raise concerns about its capacity to control bleeding at CS, this could, in theory, be circumvented by injecting it earlier, for example at uterine incision instead of after foetal delivery. Potential advantages of IM compared to IV administration include fewer side effects due to the slower increase in oxytocin plasma concentration, and a longer lasting effect[18 19] which could preclude the need for IV oxytocin maintenance infusions. Intravenous oxytocin is a high-alert medication[40] (a drug with increased risk of causing significant patient harm when used in error) which was listed in third place in the list of the top medication errors in 2020[41]. Therefore, in theory, an additional safety advantage of the IM route is that it could potentially reduce medication errors associated with IV oxytocin. Finally, for women having a CS, prophylactic IM oxytocin injection would be painless because the patient would be under anaesthesia. It was also surprising to find three trials that assessed IMY oxytocin administration[30-32] because the drug is not licenced for IMY use, and there are no pharmacokinetic studies on this route of oxytocin administration. In order to license IMY oxytocin administration, the first step would be to conduct pharmacokinetic/pharmacodynamic (PK/PD) as well as safety studies to ensure that the drug administered in the new route is effective and safe. However, since PK/PD and safety studies are expensive and complex, it may be difficult to find institutions willing to conduct this type of study.[42]

Strengths and Limitations of the review

Previous reviews have compared various uterotonics (including oxytocin) versus placebo or other agents in different doses, regimens, and timing of administration for preventing PPH at CS [14 43]. However, to the best of our knowledge, this is the first systematic review to assess different routes of oxytocin administration for preventing PPH in women giving birth by CS. Strong points of our review include its comprehensive literature search without language restrictions and strict adherence to standard Cochrane methods[33]. Limitations of the review include the small number of trials identified, the lack of response from authors to clarify important

methodological and clinical aspects of their studies, and the fact that none of the trials addressed additional core clinical outcomes.

Implications for Practice and Research

At present, the limited, low to very low certainty evidence on the effects of IMY versus IV oxytocin administration at CS is insufficient to support choosing one route over another for preventing PPH. Our review findings indicate the need for more high quality trials comparing the effectiveness and safety of different routes of prophylactic oxytocin administration, including the intramuscular route, in women having a CS. Future trials should have adequate sample size, be of high methodological and reporting quality, and recruit participants who represent the general obstetric population, including women at high risk for PPH due to clinical or obstetric disorders, and women undergoing prelabour as well as intrapartum CS, with or without previous exposure to oxytocin for labour induction or augmentation. Future studies should consider recent evidence about IV dosing regimens to reduce blood loss at CS[44]. These trials also need to report all relevant PPH prevention core outcomes[35]. The results of these future trials are essential for updates of our systematic review and meta-analyses, as well as for individual patient data meta-analyses that will provide more robust evidence on this topic. Given the increasing numbers of births by CS worldwide including in LMIC, and the high rates of maternal mortality following CS in these countries[45], research to identify the most beneficial route and regimen for prophylactic oxytocin for preventing PPH in women giving birth by CS should be prioritized to avoid preventable mortality and morbidity. Particularly disturbing is the lack of evidence for women having intrapartum CS. These women are particularly vulnerable to uterine atony and increased post-partum bleeding because of prolonged labours, often with previous exposure to oxytocin for induction or augmentation which may desensitize oxytocin receptors and impair myometrial response to prophylactic oxytocin after delivery[46 47]. These evidence gaps are likely to affect more dramatically women in LMIC where skills and resources to provide safe and timely CS, and to deal with its complications, are sub-optimal. This lack of evidence is thus likely to increase maternal and perinatal inequalities between and within countries.

CONCLUSIONS

There is limited, low to very low certainty, evidence on the effects of different routes of prophylactic oxytocin administration at CS for preventing blood loss. The limited evidence suggests that IMY compared to IV oxytocin may result in little to no difference in the incidence of PPH and most side effects, but may reduce nausea/vomiting and the volume of blood loss slightly. More trials are needed on this relevant question, especially on the effects of intramuscular oxytocin and involving women with intrapartum CS.

For peer review only

Author Contributions

MRT, MS, APB and MW conceptualized the study. MRT wrote the study protocol with the contributions of MS, APB, MW and RR. The search strategy was developed by COCL, MRT and RR, with the contribution of MS and APB. Citation screening and study selection were performed by MS, APB, ALCM, RLP, and COCL under the supervision of RR and MRT. Data extraction and study quality assessment were performed by MRT, ALCM, RR and RLP. Data analysis was conducted by MRT, RR, ALCM and RLP. The report was drafted by MRT with input from MS, APB and MW. All authors reviewed and approved the final manuscript. MRT is the guarantor and takes responsibility for the content of this article

Acknowledgements: none

Funding This work was supported by UNDP/UNFPA/UNICEF/WHO/World Bank Special Programme of Research, Development and Research Training in Human Reproduction (HRP), Department of Sexual and Reproductive Health and Research, World Health Organization, Geneva, Switzerland.

Disclaimer: The funders had no role in conducting or writing the study

Competing interests: None declared.

Patient consent: Not required

Ethics approval: This systematic review used published studies. Ethical approval was waived.

Provenance and peer review: Not commissioned; externally peer reviewed.

Data sharing statement: All data relevant to the study are included in the article or uploaded as supplementary information

Figure legends:

Figure 1. Flowchart of the process of study selection
Figure 2. Risk of bias summary of trials on oxytocin route of delivery at caesarean
Figure 3. Forest plot of intramyometrial versus intravenous oxytocin at caesarean. Outcome: need for additional uterotonic
Figure 4. Forest plot of intramyometrial versus intravenous oxytocin at caesarean. Outcome: nausea and/or vomiting

For peer review only

References

1. Betran AP, Ye J, Moller AB, et al. The Increasing Trend in Caesarean Section Rates: Global, Regional and National Estimates: 1990-2014. *PLoS One* 2016;11(2):e0148343. doi: 10.1371/journal.pone.0148343
2. Fawcus S, Moodley J. Postpartum haemorrhage associated with caesarean section and caesarean hysterectomy. *Best Pract Res Clin Obstet Gynaecol* 2013;27(2):233-49. doi: 10.1016/j.bpobgyn.2012.08.018
3. Parant O, Guerby P, Bayoumeu F. [Obstetric and anesthetic specificities in the management of a postpartum hemorrhage (PPH) associated with cesarean section]. *J Gynecol Obstet Biol Reprod (Paris)* 2014;43(10):1104-22. doi: 10.1016/j.jgyn.2014.10.004
4. Deneux-Tharaux C, Bonnet MP, Tort J. [Epidemiology of post-partum haemorrhage]. *J Gynecol Obstet Biol Reprod (Paris)* 2014;43(10):936-50. doi: 10.1016/j.jgyn.2014.09.023
5. Say L, Chou D, Gemmill A, et al. Global causes of maternal death: a WHO systematic analysis. *Lancet Glob Health* 2014;2(6):e323-33. doi: 10.1016/S2214-109X(14)70227-X
6. World Health Organization. WHO recommendations: uterotonics for the prevention of postpartum haemorrhage. Geneva: World Health Organization; 2018. Licence: CC BY-NC-SA 3.0 IGO.
7. Oladapo OT, Okusanya BO, Abalos E, et al. Intravenous versus intramuscular prophylactic oxytocin for the third stage of labour. *Cochrane Database Syst Rev* 2020;11:CD009332. doi: 10.1002/14651858.CD009332.pub4
8. World Health Organization. WHO recommendation on routes of oxytocin administration for the prevention of postpartum haemorrhage after vaginal birth. Geneva: World Health Organization; 2020. Licence: CC BY-NC-SA 3.0 IGO.
9. Dahlke JD, Mendez-Figueroa H, Maggio L, et al. Prevention and management of postpartum hemorrhage: a comparison of 4 national guidelines. *Am J Obstet Gynecol* 2015;213(1):76 e1-76 e10. doi: 10.1016/j.ajog.2015.02.023
10. Sentilhes L, Goffinet F, Vayssiere C, et al. Comparison of postpartum haemorrhage guidelines: discrepancies underline our lack of knowledge. *BJOG* 2017;124(5):718-22. doi: 10.1111/1471-0528.14305
11. Weale N, Laxton C. Prophylactic use of oxytocin at caesarean section: where are the guidelines? *Anaesthesia* 2013;68(10):1006-9. doi: 10.1111/anae.12337
12. Carvalho JC, Balki M, Kingdom J, et al. Oxytocin requirements at elective cesarean delivery: a dose-finding study. *Obstet Gynecol* 2004;104(5 Pt 1):1005-10. doi: 10.1097/01.AOG.0000142709.04450.bd
13. Dyer RA, van Dyk D, Dresner A. The use of uterotonic drugs during caesarean section. *Int J Obstet Anesth* 2010;19(3):313-9. doi: 10.1016/j.ijoa.2010.04.011
14. Stephens LC, Bruessel T. Systematic review of oxytocin dosing at caesarean section. *Anaesth Intensive Care* 2012;40(2):247-52. doi: 10.1177/0310057X1204000206
15. Roach MK, Abramovici A, Tita AT. Dose and duration of oxytocin to prevent postpartum hemorrhage: a review. *Am J Perinatol* 2013;30(7):523-8. doi: 10.1055/s-0032-1329184
16. Yamaguchi ET, Siaulys MM, Torres ML. Oxytocin in cesarean-sections. What's new? *Braz J Anesthesiol* 2016;66(4):402-7. doi: 10.1016/j.bjane.2014.11.015
17. Balki M, Tsen L. Oxytocin protocols for cesarean delivery. *Int Anesthesiol Clin* 2014;52(2):48-66. doi: 10.1097/AIA.0000000000000016
18. FDA Approved Drug Products: Pitocin (oxytocin) injection. Available at: https://www.accessdata.fda.gov/drugsatfda_docs/label/2014/018261s031lbl.pdf. Accessed on December 20, 2020.

For peer review only

19. Prescribers Digital Reference (PDR): Oxytocin - Drug Summary. Available at: https://www.pdr.net/drug-summary/Pitocin-oxytocin-1666. Accessed on December 20, 2020.
20. Pinder AJ, Dresner M, Calow C, et al. Haemodynamic changes caused by oxytocin during caesarean section under spinal anaesthesia. *Int J Obstet Anesth* 2002;11(3):156-9. doi: 10.1054/ijoa.2002.0970
21. Thomas JS, Koh SH, Cooper GM. Haemodynamic effects of oxytocin given as i.v. bolus or infusion on women undergoing Caesarean section. *Br J Anaesth* 2007;98(1):116-9. doi: 10.1093/bja/ael302
22. Tsen LC, Balki M. Oxytocin protocols during cesarean delivery: time to acknowledge the risk/benefit ratio? *Int J Obstet Anesth* 2010;19(3):243-5. doi: 10.1016/j.ijoa.2010.05.001
23. Jonsson M, Hanson U, Lidell C, et al. ST depression at caesarean section and the relation to oxytocin dose. A randomised controlled trial. *BJOG* 2010;117(1):76-83. doi: 10.1111/j.1471-0528.2009.02356.x
24. Svanstrom MC, Biber B, Hanes M, et al. Signs of myocardial ischaemia after injection of oxytocin: a randomized double-blind comparison of oxytocin and methylergometrine during Caesarean section. *Br J Anaesth* 2008;100(5):683-9. doi: 10.1093/bja/aen071
25. Thomas TA, Cooper GM, Editorial Board of the Confidential Enquiries into Maternal Deaths in the United K. Maternal deaths from anaesthesia. An extract from Why mothers die 1997-1999, the Confidential Enquiries into Maternal Deaths in the United Kingdom. *Br J Anaesth* 2002;89(3):499-508.
26. Sarna MC, Soni AK, Gomez M, et al. Intravenous oxytocin in patients undergoing elective cesarean section. *Anesth Analg* 1997;84(4):753-6. doi: 10.1097/00000539-199704000-00010
27. Unterscheider J, Breathnach F, Geary M. Standard medical therapy. In: Arulkumaran S, Karoshi M, Keith LG, et al., eds. A comprehensive textbook of postpartum hemorrhage An essential clinical reference for effective management. 2nd ed. London: Sapiens Publishing Ltd 2012:355-60.
28. Catanzarite VA. Prophylactic intramyometrial carboprost tromethamine does not substantially reduce blood loss relative to intramyometrial oxytocin at routine cesarean section. *Am J Perinatol* 1990;7(1):39-42. doi: 10.1055/s-2007-999443
29. Hayashi RH, Castillo MS, Noah ML. Management of severe postpartum hemorrhage due to uterine atony using an analogue of prostaglandin F2 alpha. *Obstet Gynecol* 1981;58(4):426-9.
30. Akinaga C, Uchizaki S, Kurita T, et al. Randomized double-blind comparison of the effects of intramyometrial and intravenous oxytocin during elective cesarean section. *J Obstet Gynaecol Res* 2016;42(4):404-9. doi: 10.1111/jog.12926
31. Dennehy KC, Rosaeg OP, Cicutti NJ, et al. Oxytocin injection after caesarean delivery: intravenous or intramyometrial? *Can J Anaesth* 1998;45(7):635-9. doi: 10.1007/BF03012092
32. Mangla D, Goel JK, Goel R. Prophylactic Intramyometrial Oxytocin before Placenta Delivery during Cesarean Section Prevents Postpartum Hemorrhage: A Prospective Randomized Study of 150 Women. *J South Asian Feder Obst Gynae* 2012;4(2):93-96.
33. Higgins JPT, Thomas J, Chandler J, et al. Cochrane Handbook for Systematic Reviews of Interventions version 6.1 [Internet]. 2020. Available at www.training.cochrane.org/handbook. In: Cochrane, ed.
34. Moher D, Liberati A, Tetzlaff J, et al. Preferred reporting items for systematic reviews and meta-analyses: the PRISMA statement. *J Clin Epidemiol* 2009;62(10):1006-12. doi: 10.1016/j.jclinepi.2009.06.005

For peer review only

35. Meher S, Cuthbert A, Kirkham JJ, et al. Core outcome sets for prevention and treatment of postpartum haemorrhage: an international Delphi consensus study. *BJOG* 2019;126(1):83-93. doi: 10.1111/1471-0528.15335
36. Ouzzani M, Hammady H, Fedorowicz Z, et al. Rayyan-a web and mobile app for systematic reviews. *Syst Rev* 2016;5(1):210. doi: 10.1186/s13643-016-0384-4
37. Guyatt GH, Oxman AD, Vist GE, et al. GRADE: an emerging consensus on rating quality of evidence and strength of recommendations. *BMJ* 2008;336(7650):924-6. doi: 10.1136/bmj.39489.470347.AD
38. Uvnas-Moberg K, Ekstrom-Bergstrom A, Berg M, et al. Maternal plasma levels of oxytocin during physiological childbirth - a systematic review with implications for uterine contractions and central actions of oxytocin. *BMC Pregnancy Childbirth* 2019;19(1):285. doi: 10.1186/s12884-019-2365-9
39. Yamaguchi ET, Cardoso MM, Torres ML, et al. Serum oxytocin concentrations in elective caesarean delivery: a randomized comparison of three infusion regimens. *Int J Obstet Anesth* 2011;20(3):224-8. doi: 10.1016/j.ijoa.2011.03.004
40. Institute for Safe Medication Practices-ISMP 2018. High-Alert Medications in Acute Care Settings. Available at: https://www.ismp.org/recommendations/high-alert-medications-acute-list
41. Institute for Safe Medication Practices-ISMP 2021. Start the Year Off Right by Preventing These Top 10 Medication Errors and Hazards from 2020. Available at: https://www.ismp.org/resources/start-year-right-preventing-these-top-10-medication-errors-and-hazards-2020.
42. U.S. Department of Health and Human Services, Food and Drug Administration, Center for Drug Evaluation and Research (CDER). Guidance for Industry: Changes to an Approved NDA or ANDA. April 2004. Available at: https://www.fda.gov/files/drugs/published/Changes-to-an-Approved-NDA-or-ANDA.pdf.
43. Gallos I, Williams H, Price M, et al. Uterotonic drugs to prevent postpartum haemorrhage: a network meta-analysis. *Health Technol Assess* 2019;23(9):1-356. doi: 10.3310/hta23090
44. Phung LC, Farrington EK, Connolly M, et al. Intravenous oxytocin dosing regimens for postpartum hemorrhage prevention at cesarean section: a systematic review and meta-analysis. *Am J Obstet Gynecol* 2021 doi: 10.1016/j.ajog.2021.04.258
45. Sobhy S, Arroyo-Manzano D, Murugesu N, et al. Maternal and perinatal mortality and complications associated with caesarean section in low-income and middle-income countries: a systematic review and meta-analysis. *Lancet* 2019;393(10184):1973-82. doi: 10.1016/S0140-6736(18)32386-9
46. Bischoff K, Nothacker M, Lehane C, et al. Lack of controlled studies investigating the risk of postpartum haemorrhage in cesarean delivery after prior use of oxytocin: a scoping review. *BMC Pregnancy Childbirth* 2017;17(1):399. doi: 10.1186/s12884-017-1584-1
47. Phaneuf S, Rodriguez Linares B, TambyRaja RL, et al. Loss of myometrial oxytocin receptors during oxytocin-induced and oxytocin-augmented labour. *J Reprod Fertil* 2000;120(1):91-7. doi: 10.1530/jrf.0.1200091

For peer review only

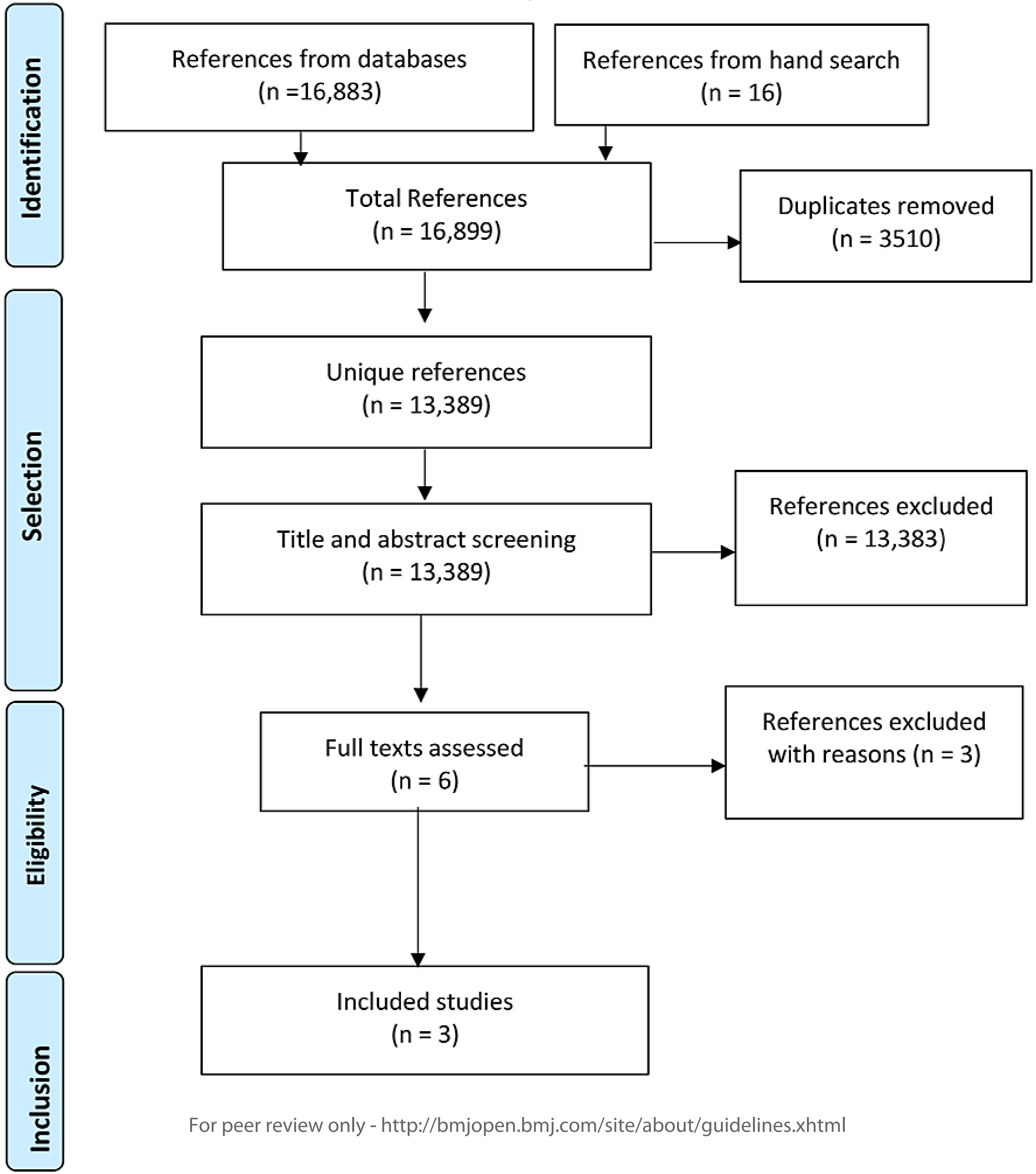
Identification
Selection
Eligibility
Inclusion
References from databases (n =16,883)
References from hand search (n = 16)
Total References (n = 16,899)
Duplicates removed (n = 3510)
Unique references (n = 13,389)
Title and abstract screening (n = 13,389)
References excluded (n = 13,383)
Full texts assessed (n = 6)
References excluded with reasons (n = 3)
Included studies (n = 3)

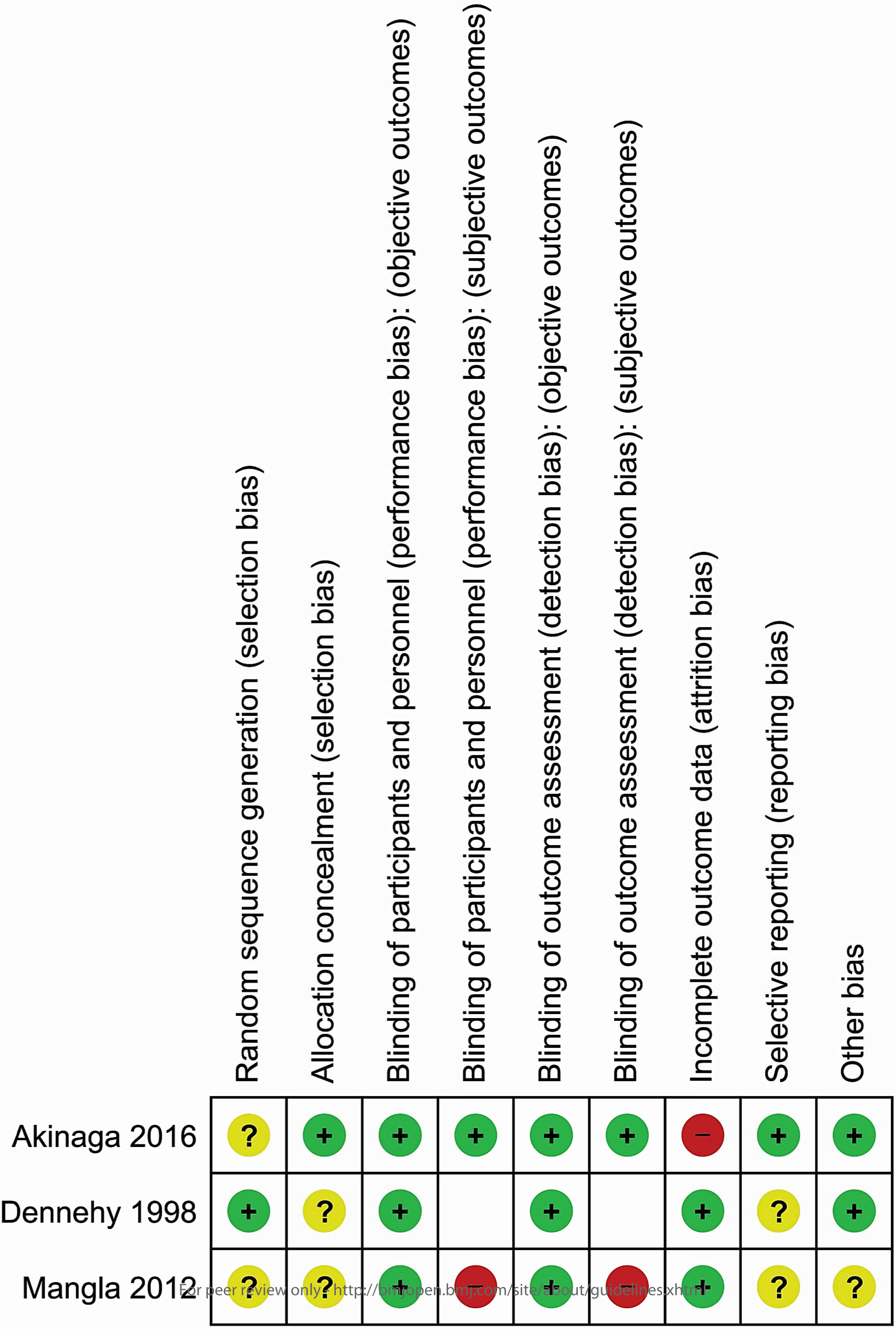
Random sequence generation (selection bias)
Allocation concealment (selection bias)
Blinding of participants and personnel (performance bias): (objective outcomes)
Blinding of participants and personnel (performance bias): (subjective outcomes)
Blinding of outcome assessment (detection bias): (objective outcomes)
Blinding of outcome assessment (detection bias): (subjective outcomes)
Incomplete outcome data (attrition bias)
Selective reporting (reporting bias)
Other bias
Akinaga 2016
Dennehy 1998
Mangla 2012

Study or Subgroup	Intramyometrial oxytocin Events	Intramyometrial oxytocin Total	Intravenous oxytocin Events	Intravenous oxytocin Total	Weight	Risk Ratio M-H, Fixed, 95% CI
Akinaga 2016	4	20	2	20	36.4%	2.00 [0.41, 9.71]
Dennehy 1998	0	20	0	19		Not estimable
Mangla 2012	0	50	3	50	63.6%	0.14 [0.01, 2.70]
**Total (95% CI)**		**70**		**70**	**100.0%**	**0.82 [0.25, 2.69]**
Total events	4		5			

Heterogeneity: Chi² = 2.58, df = 1 (P = 0.11); I^2 = 61%

Test for overall effect: Z = 0.33 (P = 0.74)

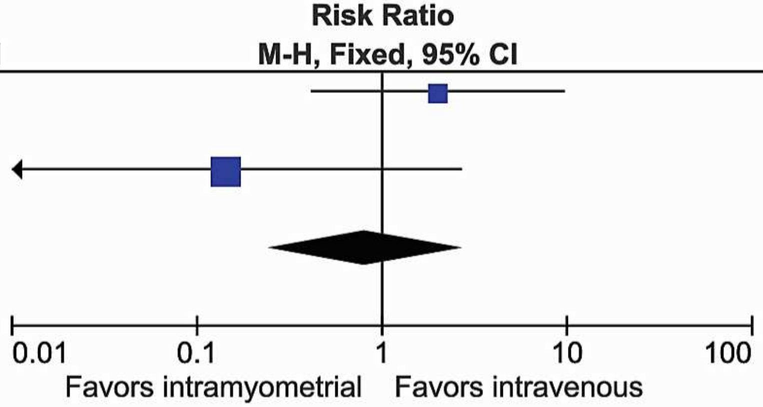

For peer review only

Study or Subgroup	Intramyometrial oxytocin Events	Intramyometrial oxytocin Total	Intravenous oxytocin Events	Intravenous oxytocin Total	Weight	Risk Ratio M-H, Fixed, 95% CI
Akinaga 2016	0	20	1	20	13.0%	0.33 [0.01, 7.72]
Mangla 2012	1	50	10	50	87.0%	0.10 [0.01, 0.75]
**Total (95% CI)**		**70**		**70**	**100.0%**	**0.13 [0.02, 0.69]**
Total events	1		11			

Heterogeneity: Chi² = 0.41, df = 1 (P = 0.52); I^2 = 0%
Test for overall effect: Z = 2.39 (P = 0.02)

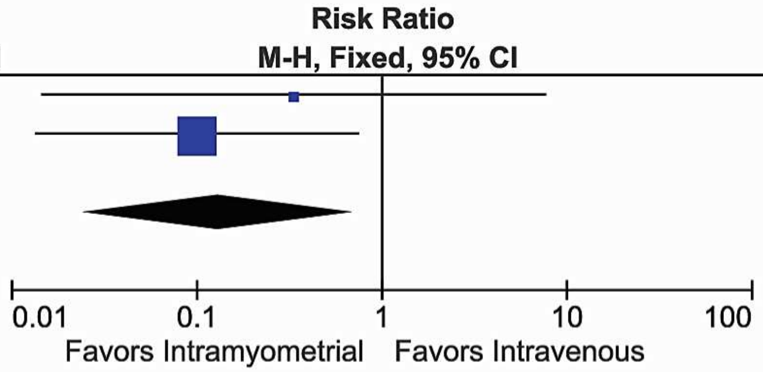

NIHR | National Institute for Health Research

PROSPERO
International prospective register of systematic reviews

Prophylactic oxytocin to prevent postpartum hemorrhage at cesarean section

Maria Regina Torloni, Monica Siaulys, Rachel Riera, Ana Pilar Betran, Mariana Widmer

To enable PROSPERO to focus on COVID-19 registrations during the 2020 pandemic, this registration record was automatically published exactly as submitted. The PROSPERO team has not checked eligibility.

Citation

Maria Regina Torloni, Monica Siaulys, Rachel Riera, Ana Pilar Betran, Mariana Widmer. Prophylactic oxytocin to prevent postpartum hemorrhage at cesarean section. PROSPERO 2020 CRD42020186797 Available from: https://www.crd.york.ac.uk/prospero/display_record.php?ID=CRD42020186797

Review question

In women submitted to cesarean section, what is the effectiveness and safety of prophylactic oxytocin administered in different periods, regimens, routes, and doses?

Searches

We will develop a search strategy combining key words related to oxytocin and cesarean delivery that will be adapted and run in several databases such as PubMed, Embase, CENTRAL and CINAHL. There will be no date or language limitations.

We will also create and run a search for ongoing studies in trial registry platforms.

The electronic search will be complemented by screening the reference lists of all included studies and other systematic reviews on prevention of postpartum hemorrhage (PPH).

Types of study to be included

All randomized controlled trials of effectiveness or side-effects of oxytocin versus oxytocin to prevent PPH in women submitted to CS will be included.

Condition or domain being studied

Prevention of postpartum hemorrhage. Cesarean section. Oxytocin

Participants/population

Inclusion criteria:

We will include studies that enrolled women of any age, race, socioeconomic level, nationality, with or without any health problems, at high or low risk for PPH, who delivered by cesarean section (CS) for any indication, with any number of fetuses, and at any gestational age after 26 weeks. We will include trials that enrolled women who had a primary or a repeat CS, a prelabour (planned or emergency) CS, or an intrapartum CS. We will include studies where participants received oxytocin for labor induction or augmentation before the CS. Studies that tested oxytocin to prevent PPH in participants having vaginal and cesarean deliveries will be included if the authors present data separately for the women having a CS.

Exclusion criteria:

We will exclude studies that assessed oxytocin in combination with another uterotonic drug, or that compared oxytocin versus placebo or other uterotonic drugs to prevent PPH.

Intervention(s), exposure(s)

We will compare different routes, dosages, regimen, or periods of administration of oxytocin to prevent PPH in women submitted to CS. We will include trials in which non-pharmacologic co-interventions (e.g. uterine massage) were included as a randomized intervention in all arms of the trial.

For peer review only - http://bmjopen.bmj.com/site/about/guidelines.xhtml

Multi-arm trials that compared oxytocin versus other uterotonic drugs, but that also compared different routes, dosages, regimens or periods of administration of oxytocin alone will be included.

Comparator(s)/control

Any route, dosage, regimens or periods of administration of oxytocin to prevent PPH at CS that are different from the intervention group.

Main outcome(s)

1. Postpartum hemorrhage of at least 1000 mL assessed by any method
2. Need for additional uterotonics

3. Any side effects

* Measures of effect

Incidence, Relative risk. We will use other effect measures depending on the data reported in the trials.

Additional outcome(s)

1. Volume of blood loss (mL) measured by any method

2. Blood transfusion after placental delivery

3. Shock, as defined by trialists

4. Maternal mortality or severe maternal morbidity related to PPH (organ failure, coma, hysterectomy, or as defined by the study authors)

5. Maternal transfer to a higher level of care

6. Maternal satisfaction (or sense of wellbeing) as defined by trialists

7. Breastfeeding at discharge

* Measures of effect

Incidence, Relative risk. We will use other effect measures depending on the data reported in the trials.

Data extraction (selection and coding)

The references retrieved from each database will be uploaded into a reference management software and duplicates will be excluded. Two review authors will independently screen titles and abstracts to select potentially relevant records. Studies selected at first screening will be read by two independent authors who will include those that fulfill the selection criteria. Reasons for exclusion will be listed. Disagreements in these two phases will be solved by a third review author.

Two authors will independently conduct data extraction. Disagreements will be solved by a third review author. We will contact study authors by email to obtain missing information and study details, when necessary.

We will use a form created for this review to extract data from each included study. The data will be entered in the Review Manager software and checked for accuracy. The following information will be collected from each study: country, year, trial registration, design details, number of participants, inclusion and exclusion criteria, main participant characteristics, type of CS, anesthesia, previous exposure to oxytocin, interventions compared and outcomes reported.

Risk of bias (quality) assessment

The risk of bias of each included study will be assessed independently by two review authors. Disagreements will be solved by a third reviewer. Full justifications for the assessments will be included in the report.

For peer review only

We will use the Cochrane Risk of Bias (RoB) tool to assess the quality of included trials. The following domains will be assessed and graded as being at high, low, or unclear risk of bias: 1) Random sequence generation, 2) Allocation concealment, 3) Blinding of participants and personnel, 4) Blinding of outcome assessment, 5) Incomplete outcome data, 6) Selective reporting (checking for reporting bias), 7) Other bias.

Strategy for data synthesis

We will pool data of similar trials in metanalyses using the random effect model using the Review Manager software (version 5.3). For dichotomous outcomes, we will present results as summary risk ratio (RR) with 95% confidence intervals (CIs). For continuous outcomes, we will pool the results of studies that measured the outcome in the same way and calculate mean differences (MD). In metanalyses of studies that measured the same continuous outcomes using different methods, we will use the standardized mean difference (SMD).

When metanalysis is not possible, we will present the results descriptively.

We will conduct separate comparisons for prelabor and intrapartum CS.

We plan to conduct the following comparisons, where data is available:

• different moments of administration.

• different routes of administration

• different regimens

• different doses

Analysis of subgroups or subsets

We plan to conduct the following subgroup analyses for the primary outcomes, if data are available: according to pregnancy characteristics (parity, onset of labor), according to previous use of oxytocin for induction/augmentation, and according to baseline risk for PPH. We will assess subgroup differences by interaction tests available in RevMan 5 (RevMan 2014).

We plan to conduct the following sensitivity analyses for the main outcomes, if data are available: restricted only to high-quality studies, and restricted only to studies that assessed blood loss objectively.

Contact details for further information

Maria Regina Torloni
torlonimr@gmail.com

Organisational affiliation of the review

Hospital e Maternidade Santa Joana

Review team members and their organisational affiliations

Dr Maria Regina Torloni. Hospital e Maternidade Santa Joana
Dr Monica Siaulys. Hospital e Maternidade Santa Joana
Dr Rachel Riera. Universidade Federal de Sao Paulo
Dr Ana Pilar Betran. World Health Organization
Dr Mariana Widmer. World Health Organization

Collaborators

Dr Ligia Mathias. Hospital e Maternidade Santa Joana
Dr Guilherme Haelvoet. Hospital e Maternidade Santa Joana
Dr Ana Luiza Martimbianco. Universidade Federal de Sao Paulo
Carolina Cruz Latorraca. Universidade Federal de Sao Paulo
Dr Rafael Leite Pacheco. Universidade Federal de Sao Paulo

For peer review only

Type and method of review
Intervention, Meta-analysis, Systematic review

Anticipated or actual start date
06 April 2020

Anticipated completion date
31 July 2020

Funding sources/sponsors
Department of Sexual and Reproductive Health and Research, World Health Organization

Conflicts of interest

Language
English

Country
Brazil, Switzerland

Stage of review
Review Ongoing

Subject index terms status
Subject indexing assigned by CRD

Subject index terms
MeSH headings have not been applied to this record

Date of registration in PROSPERO
05 July 2020

Date of first submission
17 May 2020

Stage of review at time of this submission

Stage	Started	Completed
Preliminary searches	Yes	No
Piloting of the study selection process	Yes	No
Formal screening of search results against eligibility criteria	No	No
Data extraction	No	No
Risk of bias (quality) assessment	No	No
Data analysis	No	No

The record owner confirms that the information they have supplied for this submission is accurate and complete and they understand that deliberate provision of inaccurate information or omission of data may be construed as scientific misconduct.

The record owner confirms that they will update the status of the review when it is completed and will add publication details in due course.

For peer review only

Versions

05 July 2020

PROSPERO
This information has been provided by the named contact for this review. CRD has accepted this information in good faith and registered the review in PROSPERO. The registrant confirms that the information supplied for this submission is accurate and complete. CRD bears no responsibility or liability for the content of this registration record, any associated files or external websites.

For peer review only

Supplementary file 2. Search strategy for systematic review on oxytocin route of delivery

Database	Search strategy	Results
BVS	#1 MH:Cesárea OR MH:"Cesarean Section" OR Cesárea OR (Cesarean Section) OR (Parto Abdominal) OR (Abdominal Deliveries) OR (Abdominal Delivery) OR (C Section (OB)) OR (C-Section (OB)) OR (C-Sections (OB)) OR (Caesarean Section) OR (Caesarean Sections) OR (Cesarean Sections) OR (Postcesarean Section) OR MH:E04.520.252.500$ OR MH:"Cesárea Repetida" OR MH:"Cesarean Section, Repeat" OR MH:Recesariana OR (Cesárea Repetida) OR Recesariana OR (Repeat Cesarean Section) OR (Repeat Cesarean Sections) OR (Operación Cesárea Repetida) OR MH:E04.520.252.500.150$ OR MH:"Parto Obstétrico" OR MH:"Delivery, Obstetric" OR (Parto Obstétrico) OR (Liberação Obstétrica) OR (Obstetric Deliveries) OR (Obstetric Delivery) OR (Liberación Obstétrica) OR MH:E04.520.252$ OR MH:"Terceira Fase do Trabalho de Parto" OR MH:"Labor Stage, Third" OR MH:"Tercer Periodo del Trabajo de Parto" OR (Terceira Fase do Trabalho de Parto) OR (Tercer Periodo del Trabajo de Parto) OR (Terceiro Estágio do Trabalho de Parto) OR (Terceiro Período do Trabalho de Parto) OR (Third Labor Stage) OR (Third Labor Stages) OR (Third Stage Labor) OR MH:G08.686.784.769.326.500.110$<br><br>#2 MH:Oxitocina OR MH:Oxytocin OR MH:Ocitocina OR Oxitocina OR Oxytocin OR Ocitocina OR Ocytocin OR Pitocin OR Syntocinon OR MH:D06.472.699.631.692.433$ OR MH:D12.644.548.691.692.433$ OR MH:"Receptores de Ocitocina" OR MH:"Receptors, Oxytocin" OR MH:"Receptores de Oxitocina" OR (Receptores de Ocitocina) OR (Receptores de Oxitocina) OR (Oxytocin Receptor) OR (Oxytocin Receptors) OR MH:D12.776.543.750.695.630$ OR MH:D12.776.543.750.720.600.630$ OR MH:D12.776.543.750.750.555.630$ OR MH:D12.776.543.750.750.660.600$ OR MH:Ocitócicos OR MH:Oxytocics OR MH:Oxitócicos OR Ocitócicos OR Oxytocics OR Oxitócicos OR (Efeito Ocitócico) OR (Estimulantes Uterinos) OR (Oxytocic Agents) OR (Oxytocic Drugs) OR (Oxytocic Effect) OR (Oxytocic Effects) OR (Uterine Stimulants) OR (Efecto Oxitócico) OR MH:D27.505.696.875.737$ OR MH:D27.505.954.705.737$<br><br>#3 #1 AND #2	376
CINAHL (via EBSCO)	S1 MH Cesarean Section OR ((Cesarean Sections) OR (Cesarean Section) OR (Abdominal Deliveries) OR (Abdominal Deliver) OR (Abdominal Delivery) OR (C-Section (OB)) OR (C Section (OB)) OR (C-Sections (OB)) OR (Postcesarean Section) OR (cesarean delivery) OR (Cesarean Section) OR (Elective cesarean section) OR (Elective cesarean delivery) OR Cesarean OR (Emergency cesarean section) OR (Emergency cesarean delivery) OR (Intrapartum cesarean section) OR (Intrapartum cesarean delivery) OR (Intra-partum cesarean section) OR (Intra-partum cesarean delivery) OR (Intra partum cesarean section) OR (Intra partum cesarean delivery) OR (Caesarean Section) OR (Caesarean Sections) OR (Postcaesarean Section) OR (caesarean delivery) OR (Elective caesarean section) OR (Elective caesarean delivery) OR Caesarean OR (Emergency caesarean section) OR (Emergency caesarean delivery) OR (Intrapartum caesarean section) OR (Intrapartum caesarean delivery) OR (Intra-partum caesarean section) OR (Intra-partum caesarean delivery) OR (Intra partum caesarean section) OR (Intra partum caesarean delivery) OR (Prelabor cesarean section) OR (Prelabor cesarean delivery) OR (Pre-labor cesarean delivery) OR (Pre-labor cesarean delivery) OR (Pre labor cesarean section) OR (Pre labor cesarean delivery) OR (Prelabour cesarean section) OR (Prelabour cesarean delivery) OR (Pre-labour cesarean delivery) OR (Pre-labour cesarean delivery) OR (Pre labour cesarean section) OR (Pre labour cesarean delivery) OR (Prelabor caesarean section) OR (Prelabor caesarean delivery) OR (Pre-labor caesarean delivery) OR (Pre-labor caesarean delivery) OR (Pre labor caesarean section) OR (Pre labor caesarean delivery) OR (Prelabour caesarean section) OR (Prelabour caesarean delivery) OR (Pre-labour caesarean delivery) OR (Pre-labour caesarean delivery) OR (Pre labour caesarean section) OR (Pre labour caesarean delivery) OR (Repeat Cesarean Section) OR (Repeat Cesarean Sections) OR (Repeat Caesarean Section) OR (Repeat Caesarean Sections) OR (Obstetric anesthesia) OR (Obstetric Deliveries) OR (Obstetric Delivery) OR (Third Labor Stage) OR (Third Labor Stages) OR (Third Stage Labor) OR (Third Stage of Labor) OR (Third-stage labor) OR (Third Labour Stage) OR (Third Labour Stages) OR (Third Stage Labour) OR (Third Stage of Labour) OR (Third-stage labour)) OR MH Cesarean Section, Repeat OR MH Delivery, Obstetric OR MH Labor Stage, Third<br><br>S2 MH Oxytocin OR MH Receptors, Oxytocin OR MH Oxytocics OR (Oxytocin OR Ocytocin OR Syntocinon OR Pitocin OR (Oxytocin Receptor) OR (Oxytocin Receptors) OR (alpha hypophamine) OR (atonin o) OR (beta methyl beta mercaptobutyric acid oxytocin) OR (di sipidin) OR disipidin OR endopituitrin OR fetusin OR hypophysin OR hypophysine OR (intravenous oxytocin) OR mipareton OR neoxyn OR ocytocin OR orasthin OR orastina OR oxitocin OR (oxiton inj) OR oxitone OR oxystin OR oxytan OR (oxytocin 10 usp units in dextrose 5%) OR (oxytocin 20 usp units in dextrose 5%) OR (oxytocin 5 usp units in dextrose 5%) OR (oxytocin dimer) OR (oxytocin pentapeptide) OR (oxytocin s inj) OR oxytocine OR pareton OR partacon OR partocon OR (partocon inj) OR partolact OR partoxin OR physormon OR pitocin OR (pitocin inj) OR piton OR (piton s) OR (piton s inj) OR pituilobine OR pitupartin OR solvoxine OR synpitan OR (synthetic oxytocin inj) OR syntocinon OR (syntocinon inj) OR (syntocinon spray) OR tranoxy OR utedrin OR uteracon OR uterason OR (utron inj) OR xitocin OR Oxytocics OR (Oxytocic Drugs) OR (Uterine Stimulants) OR (Oxytocic Agents) OR (Oxytocic Effect) OR (Oxytocic Effects)<br><br>S3 S1 AND S2	1,563

ClinicalTrials.gov	Condition: (Cesarean Sections) OR (Cesarean Section) OR (Delivery, Abdominal) OR (Abdominal Deliveries) OR (Abdominal Deliver) OR (Deliveries, Abdominal) OR (Abdominal Delivery) OR "C-Section (OB)" OR "C Section (OB)" OR "C-Sections (OB)"<br><br>Intervention: Oxytocin OR Ocytocin OR Syntocinon OR Pitocin OR (Oxytocin Receptor) OR (Receptor, Oxytocin) OR (Oxytocin Receptors) OR (alpha hypophamine) OR "atonin o" OR "beta methyl beta mercaptobutyric acid oxytocin" OR "di sipidin" OR disipidin	90
Cochrane Library	#1 MeSH descriptor: [Cesarean Section] explode all trees<br>#2 (Cesarean Sections) OR (Cesarean Section) OR (Delivery, Abdominal) OR (Abdominal Deliveries) OR (Abdominal Deliver) OR (Deliveries, Abdominal) OR (Abdominal Delivery) OR "C-Section (OB)" OR "C Section (OB)" OR "C-Sections (OB)" OR (Postcesarean Section) OR (cesarean delivery) OR (Cesarean Section) OR (Elective cesarean section) OR (Elective cesarean delivery) OR Cesarean OR (Emergency cesarean section) OR (Emergency cesarean delivery) OR (Intrapartum cesarean section) OR (Intrapartum cesarean delivery) OR (Intra-partum cesarean section) OR (Intra-partum cesarean delivery) OR (Intra partum cesarean section) OR (Intra partum cesarean delivery)<br>#3 (Caesarean Section) OR (Caesarean Sections) OR (Postcaesarean Section) OR (caesarean delivery) OR (Elective caesarean section) OR (Elective caesarean delivery) OR Caesarean OR (Emergency caesarean section) OR (Emergency caesarean delivery) OR (Intrapartum caesarean section) OR (Intrapartum caesarean delivery) OR (Intra-partum caesarean section) OR (Intra-partum caesarean delivery) OR (Intra partum caesarean section) OR (Intra partum caesarean delivery)<br>#4 (Prelabor cesarean section) OR (Prelabor cesarean delivery) OR (Pre-labor cesarean delivery) OR (Pre-labor cesarean delivery) OR (Pre labor cesarean section) OR (Pre labor cesarean delivery)<br>#5 (Prelabour cesarean section) OR (Prelabour cesarean delivery) OR (Pre-labour cesarean delivery) OR (Pre-labour cesarean delivery) OR (Pre labour cesarean section) OR (Pre labour cesarean delivery)<br>#6 (Prelabor caesarean section) OR (Prelabor caesarean delivery) OR (Pre-labor caesarean delivery) OR (Pre-labor caesarean delivery) OR (Pre labor caesarean section) OR (Pre labor caesarean delivery)<br>#7 (Prelabour caesarean section) OR (Prelabour caesarean delivery) OR (Pre-labour caesarean delivery) OR (Pre-labour caesarean delivery) OR (Pre labour caesarean section) OR (Pre labour caesarean delivery)<br>#8 MeSH descriptor: [Cesarean Section, Repeat] explode all trees<br>#9 (Repeat Cesarean Section) OR (Cesarean Sections, Repeat) OR (Repeat Cesarean Sections) OR (Section, Repeat Cesarean) OR (Sections, Repeat Cesarean)<br>#10 (Repeat Caesarean Section) OR (Caesarean Sections, Repeat) OR (Repeat Caesarean Sections) OR (Section, Repeat Caesarean) OR (Sections, Repeat Caesarean) OR (Obstetric anesthesia)<br>#11 MeSH descriptor: [Delivery, Obstetric] explode all trees<br>#12 (Deliveries, Obstetric) OR (Obstetric Deliveries) OR (Obstetric Delivery)<br>#13 MeSH descriptor: [Labor Stage, Third] explode all trees<br>#14 (Labor Stages, Third) OR (Stage, Third Labor) OR (Stages, Third Labor) OR (Third Labor Stage) OR (Third Labor Stages) OR (Labor, Third Stage) OR (Third Stage Labor) OR "Third Stage of Labor" OR "Third-stage labor"<br>#15 (Labour Stage, Third) OR (Labour Stages, Third) OR (Stage, Third Labour) OR (Stages, Third Labour) OR (Third Labour Stage) OR (Third Labour Stages) OR (Labour, Third Stage) OR (Third Stage Labour) OR "Third Stage of Labour" OR "Third-stage labour"<br>#16 #1 OR #2 OR #3 OR #4 OR #5 OR #6 OR #7 OR #8 OR #9 OR #10 OR #11 OR #12 OR #13 OR #14 OR #15<br>#17 MeSH descriptor: [Oxytocin] explode all trees<br>#18 Oxytocin OR Ocytocin OR Syntocinon OR Pitocin<br>#19 MeSH descriptor: [Receptors, Oxytocin] explode all trees<br>#20 (Oxytocin Receptor) OR (Receptor, Oxytocin) OR (Oxytocin Receptors) OR (alpha hypophamine) OR "atonin o" OR "beta methyl beta mercaptobutyric acid oxytocin" OR "di sipidin" OR disipidin OR endopituitrin OR fetusin OR hypophysin OR hypophysine OR (intravenous oxytocin) OR mipareton OR neoxyn OR ocytocin OR orasthin OR orastina OR oxitocin OR "oxiton inj" OR oxitone OR oxystin OR oxytan OR (oxytocin 10 usp units in dextrose 5%) OR (oxytocin 20 usp units in dextrose 5%) OR (oxytocin 5 usp units in dextrose 5%) OR (oxytocin dimer) OR (oxytocin pentapeptide) OR "oxytocin s inj" OR oxytocine OR pareton OR partacon OR partocon OR "partocon inj" OR partolact OR partoxin OR physormon OR pitocin OR "pitocin inj" OR piton OR "piton s" OR "piton s inj" OR pituilobine OR pitupartin OR solvoxine OR synpitan OR (synthetic oxytocin inj) OR syntocinon OR "syntocinon inj" OR (syntocinon spray) OR tranoxy OR utedrin OR uteracon OR uterason OR "utron inj" OR xitocin<br>#21 MeSH descriptor: [Oxytocics] explode all trees<br>#22 Oxytocics OR (Oxytocic Drugs) OR (Drugs, Oxytocic) OR (Uterine Stimulants) OR (Stimulants, Uterine) OR (Oxytocic Agents) OR (Agents, Oxytocic) OR (Oxytocic Effect) OR (Effect, Oxytocic) OR (Oxytocic Effects) OR (Effects, Oxytocic)<br>#23 #17 OR #18 OR #19 OR #20 OR #21 OR #22<br>#24 #16 AND #23<br>#25 #24 in Trials	2,375
Embase	#1 'cesarean section'/exp OR 'cesarean section' OR 'birth, abdominal operation'/exp OR 'birth, abdominal operation' OR 'birth, caesarean'/exp OR 'birth, caesarean' OR 'caesarean birth'/exp OR 'caesarean birth' OR 'caesarean section'/exp OR 'caesarean section' OR 'caesarian birth'/exp OR 'caesarian birth' OR 'caesarian section'/exp OR 'caesarian section' OR 'cesarean delivery'/exp OR 'cesarean delivery' OR 'cesarian section'/exp OR 'cesarian section' OR 'elective repeat cesarean	3,804

For peer review only

	section'/exp OR 'elective repeat cesarean section' OR 'fetectomy'/exp OR 'fetectomy' OR 'repeated cesarotomy'/exp OR 'repeated cesarotomy' OR 'sectio caesarea'/exp OR 'sectio caesarea'<br><br>#2 'repeat cesarean section'/exp OR 'repeat cesarean section' OR 'cesarean section, repeat'/exp OR 'cesarean section, repeat' OR 'repeat cesarean section, elective'/exp OR 'repeat cesarean section, elective' OR 'repeat cesarotomy'/exp OR 'repeat cesarotomy' OR 'repeat section, cesarean'/exp OR 'repeat section, cesarean' OR 'repeated cesarean section'/exp OR 'repeated cesarean section' OR 'section, elective repeat cesarean'/exp OR 'section, elective repeat cesarean' OR 'section, repeat cesarean'/exp OR 'section, repeat cesarean'<br><br>#3 'obstetric delivery'/exp OR 'obstetric delivery' OR 'delivery'/exp OR delivery OR 'delivery pattern'/exp OR 'delivery pattern' OR 'delivery term'/exp OR 'delivery term' OR 'delivery, obstetric'/exp OR 'delivery, obstetric'<br><br>#4 'labor stage 3'/exp OR 'labor stage 3' OR 'delivery stage 3'/exp OR 'delivery stage 3' OR 'delivery stage iii'/exp OR 'delivery stage iii' OR 'labor stage iii'/exp OR 'labor stage iii' OR 'labor stage, third'/exp OR 'labor stage, third' OR 'labour stage 3'/exp OR 'labour stage 3' OR 'labour stage iii'/exp OR 'labour stage iii' OR 'labour stage, third'/exp OR 'labour stage, third' OR 'third delivery stage'/exp OR 'third delivery stage' OR 'third labor stage'/exp OR 'third labor stage' OR 'third labour stage'/exp OR 'third labour stage' OR 'third stage of delivery'/exp OR 'third stage of delivery' OR 'third stage of labor'/exp OR 'third stage of labor' OR 'third stage of labour'/exp OR 'third stage of labour'<br><br>#5 #1 OR #2 OR #3 OR #4<br><br>#6 'oxytocin' OR 'oxytocin'/exp OR oxytocin OR 'alpha hypophamine'/exp OR 'alpha hypophamine' OR 'atonin o'/exp OR 'atonin o' OR 'beta methyl beta mercaptobutyric acid oxytocin'/exp OR 'beta methyl beta mercaptobutyric acid oxytocin' OR 'di sipidin'/exp OR 'di sipidin' OR 'disipidin'/exp OR disipidin OR 'endopituitrin'/exp OR endopituitrin OR 'fetusin'/exp OR fetusin OR 'hypophysin'/exp OR hypophysin OR 'hypophysine'/exp OR hypophysine OR 'intravenous oxytocin'/exp OR 'intravenous oxytocin' OR 'mipareton'/exp OR mipareton OR 'neoxyn'/exp OR neoxyn OR 'ocytocin'/exp OR ocytocin OR 'opn 300'/exp OR 'opn 300' OR 'opn300'/exp OR opn300 OR 'orasthin'/exp OR orasthin OR 'orastina'/exp OR orastina OR 'oxitocin'/exp OR oxitocin OR 'oxiton inj'/exp OR 'oxiton inj' OR 'oxitone'/exp OR oxitone OR 'oxystin'/exp OR oxystin OR 'oxytan'/exp OR oxytan OR 'oxytocin 10 usp units in dextrose 5%'/exp OR 'oxytocin 10 usp units in dextrose 5%' OR 'oxytocin 20 usp units in dextrose 5%'/exp OR 'oxytocin 20 usp units in dextrose 5%' OR 'oxytocin 5 usp units in dextrose 5%'/exp OR 'oxytocin 5 usp units in dextrose 5%' OR 'oxytocin dimer'/exp OR 'oxytocin dimer' OR 'oxytocin pentapeptide'/exp OR 'oxytocin pentapeptide' OR 'oxytocin s inj'/exp OR 'oxytocin s inj' OR 'oxytocina'/exp OR oxytocina OR 'oxytocine'/exp OR oxytocine OR 'pareton'/exp OR pareton OR 'partacon'/exp OR partacon OR 'partocon'/exp OR partocon OR 'partocon inj'/exp OR 'partocon inj' OR 'partolact'/exp OR partolact OR 'partoxin'/exp OR partoxin OR 'physormon'/exp OR physormon OR 'pitocin'/exp OR pitocin OR 'pitocin inj'/exp OR 'pitocin inj' OR 'piton'/exp OR piton OR 'piton s'/exp OR 'piton s' OR 'piton s inj'/exp OR 'piton s inj' OR 'pituilobine'/exp OR pituilobine OR 'pitupartin'/exp OR pitupartin OR 'solvoxine'/exp OR solvoxine OR 'synpitan'/exp OR synpitan OR 'synthetic oxytocin inj'/exp OR 'synthetic oxytocin inj' OR 'syntocinon'/exp OR syntocinon OR 'syntocinon inj'/exp OR 'syntocinon inj' OR 'syntocinon spray'/exp OR 'syntocinon spray' OR 'ti 001'/exp OR 'ti 001' OR 'ti 114'/exp OR 'ti 114' OR 'ti001'/exp OR 'ti001' OR 'ti114'/exp OR 'ti114' OR 'tranoxy'/exp OR tranoxy OR 'tur 001'/exp OR 'tur 001' OR 'tur001'/exp OR 'tur001' OR 'utedrin'/exp OR utedrin OR 'uteracon'/exp OR uteracon OR 'uterason'/exp OR uterason OR 'utron inj'/exp OR 'utron inj' OR 'vagitocin'/exp OR vagitocin OR 'xitocin'/exp OR xitocin<br><br>#7 'oxytocin receptor'/exp OR 'oxytocin receptor' OR 'neuron, oxytocinergic'/exp OR 'neuron, oxytocinergic' OR 'oxytocinergic nerve cell'/exp OR 'oxytocinergic nerve cell' OR 'oxytocinergic neuron'/exp OR 'oxytocinergic neuron' OR 'receptor, oxytocin'/exp OR 'receptor, oxytocin' OR 'receptors, oxytocin'/exp OR 'receptors, oxytocin'<br><br>#8 'oxytocic agent'/exp OR 'oxytocic agent' OR 'labor inducing drug'/exp OR 'labor inducing drug' OR 'labour inducing drug'/exp OR 'labour inducing drug' OR 'ocytocic agent'/exp OR 'ocytocic agent' OR 'oxytocic activity'/exp OR 'oxytocic activity' OR 'oxytocic substance'/exp OR 'oxytocic substance' OR 'oxytocics'/exp OR oxytocics<br><br>#9 #6 OR #7 OR #8<br>#10 #5 AND #9<br>#11 #10 AND [embase]/lim NOT ([embase]/lim AND [medline]/lim)	
Global Index Medicus	MH:Cesárea OR MH:"Cesarean Section" OR Cesárea OR (Cesarean Section) OR (Parto Abdominal) OR (Abdominal Deliveries) OR (Abdominal Delivery) OR (C Section (OB)) OR (C-Section (OB)) OR (C-Sections (OB)) OR (Caesarean Section) OR (Caesarean Sections) OR (Cesarean Sections) OR (Postcesarean Section) OR MH:E04.520.252.500$ OR MH:"Cesárea Repetida" OR MH:"Cesarean Section, Repeat" OR MH:Recesariana OR (Cesárea Repetida) OR Recesariana OR (Repeat Cesarean Section) OR (Repeat Cesarean Sections) OR (Operación Cesárea Repetida) OR MH:E04.520.252.500.150$ OR MH:"Parto Obstétrico" OR MH:"Delivery, Obstetric" OR (Parto Obstétrico) OR (Liberação Obstétrica) OR (Obstetric Deliveries) OR (Obstetric Delivery) OR (Liberación Obstétrica) OR MH:E04.520.252$ OR MH:"Terceira Fase do Trabalho de Parto" OR MH:"Labor Stage, Third" OR MH:"Tercer Periodo del Trabajo de Parto" OR (Terceira Fase do Trabalho de Parto) OR (Tercer Periodo del Trabajo de Parto) OR (Terceiro Estágio do Trabalho de	421

For peer review only

	Parto) OR (Terceiro Período do Trabalho de Parto) OR (Third Labor Stage) OR (Third Labor Stages) OR (Third Stage Labor) OR MH:G08.686.784.769.326.500.110$<br>AND<br>MH:Oxitocina OR MH:Oxytocin OR MH:Ocitocina OR Oxitocina OR Oxytocin OR Ocitocina OR Ocytocin OR Pitocin OR Syntocinon OR MH:D06.472.699.631.692.433$ OR MH:D12.644.548.691.692.433$ OR MH:"Receptores de Ocitocina" OR MH:"Receptors, Oxytocin" OR MH:"Receptores de Oxitocina" OR (Receptores de Ocitocina) OR (Receptores de Oxitocina) OR (Oxytocin Receptor) OR (Oxytocin Receptors) OR MH:D12.776.543.750.695.630$ OR MH:D12.776.543.750.720.600.630$ OR MH:D12.776.543.750.750.555.630$ OR MH:D12.776.543.750.750.660.600$ OR MH:Ocitócicos OR MH:Oxytocics OR MH:Oxitócicos OR Ocitócicos OR Oxytocics OR Oxitócicos OR (Efeito Ocitócico) OR (Estimulantes Uterinos) OR (Oxytocic Agents) OR (Oxytocic Drugs) OR (Oxytocic Effect) OR (Oxytocic Effects) OR (Uterine Stimulants) OR (Efecto Oxitócico) OR MH:D27.505.696.875.737$ OR MH:D27.505.954.705.737$	
MEDLINE (via PubMed)	#1 "Cesarean Section"[Mesh] OR (Cesarean Sections) OR (Cesarean Section) OR (Delivery, Abdominal) OR (Abdominal Deliveries) OR (Abdominal Deliver) OR (Deliveries, Abdominal) OR (Abdominal Delivery) OR "C-Section (OB)" OR "C Section (OB)" OR "C-Sections (OB)" OR (Postcesarean Section) OR (cesarean delivery) OR (Cesarean Section) OR (Elective cesarean section) OR (Elective cesarean delivery) OR Cesarean OR (Emergency cesarean section) OR (Emergency cesarean delivery) OR (Intrapartum cesarean section) OR (Intrapartum cesarean delivery) OR (Intra-partum cesarean section) OR (Intra-partum cesarean delivery) OR (Intra partum cesarean section) OR (Intra partum cesarean delivery)<br><br>#2 (Caesarean Section) OR (Caesarean Sections) OR (Postcaesarean Section) OR (caesarean delivery) OR (Elective caesarean section) OR (Elective caesarean delivery) OR Caesarean OR (Emergency caesarean section) OR (Emergency caesarean delivery) OR (Intrapartum caesarean section) OR (Intrapartum caesarean delivery) OR (Intra-partum caesarean section) OR (Intra-partum caesarean delivery) OR (Intra partum caesarean section) OR (Intra partum caesarean delivery)<br><br>#3 (Prelabor cesarean section) OR (Prelabor cesarean delivery) OR (Pre-labor cesarean delivery) OR (Pre-labor cesarean delivery) OR (Pre labor cesarean section) OR (Pre labor cesarean delivery)<br><br>#4 (Prelabour cesarean section) OR (Prelabour cesarean delivery) OR (Pre-labour cesarean delivery) OR (Pre-labour cesarean delivery) OR (Pre labour cesarean section) OR (Pre labour cesarean delivery)<br><br>#5 (Prelabor caesarean section) OR (Prelabor caesarean delivery) OR (Pre-labor caesarean delivery) OR (Pre-labor caesarean delivery) OR (Pre labor caesarean section) OR (Pre labor caesarean delivery)<br>#6 (Prelabour caesarean section) OR (Prelabour caesarean delivery) OR (Pre-labour caesarean delivery) OR (Pre-labour caesarean delivery) OR (Pre labour caesarean section) OR (Pre labour caesarean delivery)<br><br>#7 "Cesarean Section, Repeat'[Mesh] OR (Repeat Cesarean Section) OR (Cesarean Sections, Repeat) OR (Repeat Cesarean Sections) OR (Section, Repeat Cesarean) OR (Sections, Repeat Cesarean)<br><br>#8 (Repeat Caesarean Section) OR (Caesarean Sections, Repeat) OR (Repeat Caesarean Sections) OR (Section, Repeat Caesarean) OR (Sections, Repeat Caesarean) OR (Obstetric anesthesia)<br><br>#9 "Delivery, Obstetric"[Mesh] OR (Deliveries, Obstetric) OR (Obstetric Deliveries) OR (Obstetric Delivery)<br><br>#10 "Labor Stage, Third"[Mesh] OR (Labor Stages, Third) OR (Stage, Third Labor) OR (Stages, Third Labor) OR (Third Labor Stage) OR (Third Labor Stages) OR (Labor, Third Stage) OR (Third Stage Labor) OR "Third Stage of Labor" OR "Third-stage labor"<br><br>#11 (Labour Stage, Third) OR (Labour Stages, Third) OR (Stage, Third Labour) OR (Stages, Third Labour) OR (Third Labour Stage) OR (Third Labour Stages) OR (Labour, Third Stage) OR (Third Stage Labour) OR "Third Stage of Labour" OR "Third-stage labour"<br><br>#12 #1 OR #2 OR #3 OR #4 OR #5 OR #6 OR #7 OR #8 OR #9 OR #10 OR #11<br><br>#13 "Oxytocin"[Mesh] OR Oxytocin OR Ocytocin OR Syntocinon OR Pitocin<br><br>#14 "Receptors, Oxytocin"[Mesh] OR (Oxytocin Receptor) OR (Receptor, Oxytocin) OR (Oxytocin Receptors) OR (alpha hypophamine) OR "atonin o" OR "beta methyl beta mercaptobutyric acid oxytocin" OR "di sipidin" OR disipidin OR endopituitrin OR fetusin OR hypophysin OR hypophysine OR (intravenous oxytocin) OR mipareton OR neoxyn OR ocytocin OR orasthin OR orastina OR oxitocin OR "oxiton inj" OR oxitone OR oxystin OR oxytan OR (oxytocin 10 usp units in dextrose 5%) OR (oxytocin 20 usp units in dextrose 5%) OR (oxytocin 5 usp units in dextrose 5%) OR (oxytocin dimer) OR (oxytocin pentapeptide) OR "oxytocin s inj" OR oxytocine OR pareton OR partacon OR partocon OR "partocon inj" OR partolact OR partoxin OR physormon OR pitocin OR "pitocin inj" OR piton OR "piton s" OR "piton s inj" OR pituilobine OR pitupartin OR solvoxine OR synpitan OR	7,944

	(synthetic oxytocin inj) OR syntocinon OR "syntocinon inj" OR (syntocinon spray) OR tranoxy OR utedrin OR uteracon OR uterason OR "utron inj" OR xitocin<br>#15 "Oxytocics"[Mesh] OR Oxytocics OR (Oxytocic Drugs) OR (Drugs, Oxytocic) OR (Uterine Stimulants) OR (Stimulants, Uterine) OR (Oxytocic Agents) OR (Agents, Oxytocic) OR (Oxytocic Effect) OR (Effect, Oxytocic) OR (Oxytocic Effects) OR (Effects, Oxytocic)<br><br>#16 "Oxytocics" [Pharmacological Action]<br>#17 #13 OR #14 OR #15 OR #16<br>#18 #12 AND #17<br>#19 ((clinical[Title/Abstract] AND trial[Title/Abstract]) OR clinical trials as topic[MeSH Terms] OR clinical trial[Publication Type] OR random*[Title/Abstract] OR random allocation[MeSH Terms] OR therapeutic use[MeSH Subheading])<br>#20 #18 AND #19	
Opengrey	Cesarean Sections) OR (Cesarean Section) OR (Delivery, Abdominal) OR (Abdominal Deliveries) OR (Abdominal Deliver) OR (Deliveries, Abdominal) OR (Abdominal Delivery) OR "C-Section (OB)" OR "C Section (OB)" OR "C-Sections (OB)" OR (Postcesarean Section) OR (cesarean delivery) OR (Cesarean Section) OR (Elective cesarean section) OR (Elective cesarean delivery) OR Cesarean OR (Emergency cesarean section) OR (Emergency cesarean delivery) OR (Intrapartum cesarean section) OR (Intrapartum cesarean delivery) OR (Intra-partum cesarean section) OR (Intra-partum cesarean delivery) OR (Intra partum cesarean section) OR (Intra partum cesarean delivery) OR (Caesarean Section) OR (Caesarean Sections) OR (Postcaesarean Section) OR (caesarean delivery) OR (Elective caesarean section) OR (Elective caesarean delivery) OR Caesarean OR (Emergency caesarean section) OR (Emergency caesarean delivery) OR (Intrapartum caesarean section) OR (Intrapartum caesarean delivery) OR (Intra-partum caesarean section) OR (Intra-partum caesarean delivery) OR (Intra partum caesarean section) OR (Intra partum caesarean delivery) OR (Prelabor cesarean section) OR (Prelabor cesarean delivery) OR (Pre-labor cesarean delivery) OR (Pre-labor cesarean delivery) OR (Pre labor cesarean section) OR (Pre labor cesarean delivery) OR (Prelabour cesarean section) OR (Prelabour cesarean delivery) OR (Pre-labour cesarean delivery) OR (Pre-labour cesarean delivery) OR (Pre labour cesarean section) OR (Pre labour cesarean delivery) OR (Prelabor caesarean section) OR (Prelabor caesarean delivery) OR (Pre-labor caesarean delivery) OR (Pre-labor caesarean delivery) OR (Pre labor caesarean section) OR (Pre labor caesarean delivery) OR (Prelabour caesarean section) OR (Prelabour caesarean delivery) OR (Pre-labour caesarean delivery) OR (Pre-labour caesarean delivery) OR (Pre labour caesarean section) OR (Pre labour caesarean delivery) OR (Repeat Cesarean Section) OR (Cesarean Sections, Repeat) OR (Repeat Cesarean Sections) OR (Section, Repeat Cesarean) OR (Sections, Repeat Cesarean) OR (Repeat Caesarean Section) OR (Caesarean Sections, Repeat) OR (Repeat Caesarean Sections) OR (Section, Repeat Caesarean) OR (Sections, Repeat Caesarean) OR (Obstetric anesthesia) OR (Deliveries, Obstetric) OR (Obstetric Deliveries) OR (Obstetric Delivery) OR (Labor Stages, Third) OR (Stage, Third Labor) OR (Stages, Third Labor) OR (Third Labor Stage) OR (Third Labor Stages) OR (Labor, Third Stage) OR (Third Stage Labor) OR "Third Stage of Labor" OR "Third-stage labor" OR (Labour Stage, Third) OR (Labour Stages, Third) OR (Stage, Third Labour) OR (Stages, Third Labour) OR (Third Labour Stage) OR (Third Labour Stages) OR (Labour, Third Stage) OR (Third Stage Labour) OR "Third Stage of Labour" OR "Third-stage labour"	126
SciELO	((Cesarean Sections) OR (Cesarean Section) OR (Abdominal Deliveries) OR (Abdominal Deliver) OR (Abdominal Delivery) OR (C-Section (OB)) OR (C Section (OB)) OR (C-Sections (OB)) OR (Postcesarean Section) OR (cesarean delivery) OR (Cesarean Section) OR (Elective cesarean section) OR (Elective cesarean delivery) OR Cesarean OR (Emergency cesarean section) OR (Emergency cesarean delivery) OR (Intrapartum cesarean section) OR (Intrapartum cesarean delivery) OR (Intra-partum cesarean section) OR (Intra-partum cesarean delivery) OR (Intra partum cesarean section) OR (Intra partum cesarean delivery) OR (Caesarean Section) OR (Caesarean Sections) OR (Postcaesarean Section) OR (caesarean delivery) OR (Elective caesarean section) OR (Elective caesarean delivery) OR Caesarean OR (Emergency caesarean section) OR (Emergency caesarean delivery) OR (Intrapartum caesarean section) OR (Intrapartum caesarean delivery) OR (Intra-partum caesarean section) OR (Intra-partum caesarean delivery) OR (Intra partum caesarean section) OR (Intra partum caesarean delivery) OR (Prelabor cesarean section) OR (Prelabor cesarean delivery) OR (Pre-labor cesarean delivery) OR (Pre-labor cesarean delivery) OR (Pre labor cesarean section) OR (Pre labor cesarean delivery) OR (Prelabour cesarean section) OR (Prelabour cesarean delivery) OR (Pre-labour cesarean delivery) OR (Pre-labour cesarean delivery) OR (Pre labour cesarean section) OR (Pre labour cesarean delivery) OR (Prelabor caesarean section) OR (Prelabor caesarean delivery) OR (Pre-labor caesarean delivery) OR (Pre-labor caesarean delivery) OR (Pre labor caesarean section) OR (Pre labor caesarean delivery) OR (Prelabour caesarean section) OR (Prelabour caesarean delivery) OR (Pre-labour caesarean delivery) OR (Pre-labour caesarean delivery) OR (Pre labour caesarean section) OR (Pre labour caesarean delivery) OR (Repeat Cesarean Section) OR (Repeat Cesarean Sections) OR (Repeat Caesarean Section) OR (Repeat Caesarean Sections) OR (Obstetric anesthesia) OR (Obstetric Deliveries) OR (Obstetric Delivery) OR (Third Labor Stage) OR (Third Labor Stages) OR (Third Stage Labor) OR (Third Stage of Labor) OR (Third-stage labor) OR (Third Labour Stage) OR (Third Labour Stages) OR (Third Stage Labour) OR (Third Stage of Labour) OR (Third-stage labour))<br>AND<br>(Oxytocin OR Ocytocin OR Syntocinon OR Pitocin OR (Oxytocin Receptor) OR (Oxytocin Receptors) OR (alpha hypophamine) OR (atonin o) OR (beta methyl beta mercaptobutyric acid oxytocin) OR (di sipidin) OR disipidin OR endopituitrin OR fetusin OR hypophysin OR hypophysine OR (intravenous oxytocin) OR mipareton OR neoxyn OR ocytocin OR orasthin OR orastina OR oxitocin OR (oxiton inj) OR oxitone OR oxystin OR oxytan OR (oxytocin 10 usp units in dextrose 5%) OR (oxytocin 20 usp	91

	units in dextrose 5%) OR (oxytocin 5 usp units in dextrose 5%) OR (oxytocin dimer) OR (oxytocin pentapeptide) OR (oxytocin s inj) OR oxytocine OR pareton OR partacon OR partocon OR (partocon inj) OR partolact OR partoxin OR physormon OR pitocin OR (pitocin inj) OR piton OR (piton s) OR (piton s inj) OR pituilobine OR pitupartin OR solvoxine OR synpitan OR (synthetic oxytocin inj) OR syntocinon OR (syntocinon inj) OR (syntocinon spray) OR tranoxy OR utedrin OR uteracon OR uterason OR (utron inj) OR xitocin OR Oxytocics OR (Oxytocic Drugs) OR (Uterine Stimulants) OR (Oxytocic Agents) OR (Oxytocic Effect) OR (Oxytocic Effects))	
WHO - ICTRP	Condition: (Cesarean Sections) OR (Cesarean Section) OR (Delivery, Abdominal) OR (Abdominal Deliveries) OR (Abdominal Deliver) OR (Deliveries, Abdominal) OR (Abdominal Delivery) OR "C-Section (OB)" OR "C Section (OB)" OR "C-Sections (OB)"<br><br>Intervention: Oxytocin OR Ocytocin OR Syntocinon OR Pitocin OR (Oxytocin Receptor) OR (Receptor, Oxytocin) OR (Oxytocin Receptors) OR (alpha hypophamine) OR "atonin o" OR "beta methyl beta mercaptobutyric acid oxytocin" OR "di sipidin" OR disipidin	95
Total		16,883

For peer review only

Supplementary file 3. Excluded studies in systematic review on route of oxytocin administration

Excluded studies with reasons

Study	*Reason for exclusion*
Zarzur E. A Ocitocina e a Operação Cesariana. Rev Bras Anest. 1992;42(4):293-5.	Did not compare different routes
Garza-Hinojosa A, González-Cordero G. Cambios hemodinámicos y electrocardiográfi cos con el uso de oxitocina en bolo durante la cesárea. Ver Mex Anest. 2015;38(S1): S257-62.	Did not compare different routes
Singh KP, Kameshore N, Kamei H. Prophylactic intramuscular injection of oxytocin vs intravenous infusion of oxytocin to minimize blood loss at caesarean section. Int J Gynecol Obstet. 2015;131(Suppl. 5): E290.	Insufficient information (Abstract-authors did not reply to contact)

For peer review only

Supplementary file 4. Details of trials on different routes of oxytocin administration at CS

Akinaga 2016	
**Method**	Double blind, placebo controlled RCT<br>Study protocol UMIN000007577<br>(https://upload.umin.ac.jp/cgi-open-bin/ctr_e/ctr_view.cgi?recptno=R000008709)
**Participants**	**Setting**: women managed at Hamamatsu University hospital, Japan<br>**Dates of recruitment**: April to October 2012<br>**Inclusion criteria**: 40 women, ASA status I or II with a single fetus at ≥36 weeks scheduled for elective CS under spinal anesthesia<br>**Exclusion criteria**: conditions predisposing to uterine atony and postpartum hemorrhage, such as placenta previa, multiple gestations, polyhydramnios, previous uterine atony and post-partum bleeding, macrosomia, uterine fibroids, bleeding diathesis, and pre-eclampsia
**Interventions**	After clamping of umbilical cord<br>**Group 1** (intramyometrial): bolus injection of oxytocin (0.07 IU/kg) diluted to 2 mL saline was slowly (over 30 seconds) injected into the myometrium of the uterine fundus and an IV saline infusion (10 mL) was also given.<br>(n=20 randomized)<br>**Group 2**: (intravenous): 2 mL bolus saline injection was injected into the myometrium and an IV oxytocin injection (0.07 IU/kg) diluted to 10 mL with saline was also given.<br>(n=20 randomized)<br>**Both groups:** After bolus injections, both groups received oxytocin at 0.1 IU/kg in 500 mL of lactated Ringer's solution and continuously administered IV at a rate of 100 mL/h.
**Outcomes**	Volume of blood loss during CS (volume in suction bottle). Additional uterotonics. Adverse effects (hypotension, nausea/vomiting, headache, flushing)
**Notes**	Funding: not reported<br>Conflict of interest: none

For peer review only

Dennehy 1998	
**Method**	Double blind, placebo controlled RCT<br>no protocol
**Participants**	**Setting**: women managed in hospital (no other information) in Canada<br>**Dates of recruitment**: No information<br>**Inclusion criteria**: 40 ASA I or II women with singleton fetus scheduled for elective CS at term (> 37 wks).<br>**Exclusion criteria**: age <18 yr, contraindication to spinal anaesthesia, polyhydramnios, placenta praevia, uterine fibroids, hypertension or cardiac disease.
**Interventions**	Immediately after placental removal:<br>**Group 1** (IV bolus): 0.5 ml of 10 IU/ml oxytocin in unknown time and an intramyometrial 2 ml saline injection. Total bolus injection dose: 5 IU, Total bolus injection time: NI.<br>(n=20 randomized)<br>**Group 2** (Intramyometrial bolus): 2 ml of 10 IU/ml oxytocin into the uterine fundus and 0.5 mL saline IV bolus. Total bolus injection dose: 20 IU, Total bolus injection time: NI.<br>(n=20 randomized)
**Outcomes**	Need for additional uterotonics, hypotension
**Notes**	Funding: not reported<br>Conflict of interest: none

For peer review only

Mangla 2012	
**Method**	Double blind RCT<br>No protocol.
**Participants**	**Setting**: Guru Gobind Singh Hospital, Jamnagar, Gujarat, India<br>**Dates of recruitment**: No information<br>**Inclusion criteria:** "women operated by lower segment cesarean section". No other information<br>**Exclusion criteria:** No information
**Interventions**	**Group 1**: IV infusion 20 IU/500 ml Ringer after placental detachment, no information on rate or duration of infusion<br>(n=50 randomized).<br>**Group 2**: Intra-myometrial 5 IU in 10 cc normal saline; 5 cc injected in each cornu of the uterus after separation of placenta.<br>(n=50 randomized)<br>**Group 3**: Intra-myometrial 5 IU in 10 cc normal saline; 5 cc injected in each cornu of the uterus before separation of placenta.<br>(n=50 randomized) This group was not included in the SR because it was identical to Group 2 but given at a different timing
**Outcomes**	Volume of blood loss during CS and 1st hour after (measured by adding volume in graduated suction jar and number of soaked sponges; no information on how blood loss was measured in the 1st hour after).<br>Additional uterotonic. Nausea/vomiting requiring antiemetic.
**Notes**	Funding: none<br>Conflict of interest: none

CS: cesarean section, IU: international units, IV: intravenous, RCT: randomized controlled trial

For peer review only

Supplementary file 5: Postpartum hemorrhage prevention core outcome sets[1] in trials on different routes of oxytocin administration at cesarean section

Outcome		Measure (in each group)	Study		
			Akinaga 2016	Dennehy 1998	Mangla 2012
1	Blood loss (from birth up to cessation of active bleeding)				
	≥ 1000 ml	N women with this loss	no	no	no
	total volume	mean or median volume	yes	no	yes
2	Shock (defined by trialists)	N women	no	no	no
3	Maternal death				
	PPH-related maternal deaths	N women	no	no	no
	all cause mortality	N women	no	no	no
4	Blood transfusion				
	Wm receiving any RBC product	N women	no	no	no
	total volume	mean or median RBC units	no	no	no
5	Transfer to higher level of care	N women	no	no	no
6	Use of additional uterotonics	N women	yes	yes	yes
7	Adverse effects (defined by trialists)	N women	yes (headache, nausea/vomiting, flushing, hypotension)	yes (hypotension)	yes (nausea/ vomiting)
8	Breastfeeding	N women	no	no	no
9	Patient reported outcomes	N women	no	no	no

N: number, PPH: post-partum hemorrhage, RBC: red blood cell

1.Source: Meher S, Cuthbert A, Kirkham JJ, et al. Core outcome sets for prevention and treatment of postpartum haemorrhage: an international Delphi consensus study. BJOG. 2019 Jan;126(1):83-93.

For peer review only

Supplementary file 6. Risk of bias details

Judgments and justifications for risk of bias assessments in trials on different routes of oxytocin administration at cesarean section

Domain	Akinaga 2016		
1	Sequence generation	UNCLEAR	No information on random sequence generation
2	Allocation concealment	LOW	*'An anesthesiologist unrelated to this research selected one of two opaque envelopes containing a letter indicating the group name'*
3	Blinding participants/personnel (objective outcomes)	LOW	*'The subject, care providers, and investigators were blinded to patient allocation until all data collection was completed.'*
3	Blinding participants/personnel (subjective outcomes)	LOW	*'The subject, care providers, and investigators were blinded to patient allocation until all data collection was completed.'*
4	Blinding outcome assessors (objective outcomes)	LOW	*'The i.m.y. group received a bolus injection of oxytocin (0.07 IU/kg) diluted to 2 mL with saline into the myometrium of the uterine fundus during slow (30-s) i.v. infusion of saline (10 mL). Conversely, the i.v. group received a 2-mL bolus injection of saline into the myometrium during slow (30-s) i.v. infusion of oxytocin (0.07 IU/kg) diluted to 10mL with saline.'*
4	Blinding outcome assessors (subjective outcomes)	LOW	*The subject, care providers, and investigators were blinded to patient allocation until all data collection was completed.'*
5	Incomplete outcome	HIGH	*´Nine patients were excluded from the study: eight for protocol deviation and one for adenomyosis.'* High rate of loss (20%), with no information about which was the original group that the participants had been randomized to. ITT was not used.
6	Selective reporting	LOW	All outcomes described in protocol were reported
7	Other bias	LOW	There are probably no other sources of bias

For peer review only

Domain	Dennehy 1998		
1	Sequence generation	LOW	*´Participants were assigned to one of two study groups, according to a computer generated series of random numbers.'*
2	Allocation concealment	UNCLEAR	No information on allocation concealment
3	Blinding participants/personnel (objective outcomes)	LOW	*'The hospital pharmacy prepared the syringes containing the study solutions trader aseptic conditions according to the random table and delivered them to the delivery room in a plastic bag.'*
3	Blinding participants/personnel (subjective outcomes)	does not apply	No subjective outcomes
4	Blinding outcome assessors (objective outcomes)	LOW	*´The injections were conducted in a double blind fashion where neither the attending anaesthetist or obstetrician had knowledge of the content of the syringes.'*
4	Blinding outcome assessors (subjective outcomes)	does not apply	No subjective outcomes
5	Incomplete outcome	LOW	*One patient did not complete the study protocol because the attending anaesthetist was unable to locate the subarachnoid space and she, therefore, received general anaesthesia for Caesarean section*'. Low rate of losses (2%).
6	Selective reporting	UNCLEAR	No study protocol
7	Other bias	LOW	There are probably no other sources of bias

Domain	Mangla 2012		
1	Sequence generation	UNCLEAR	No information on random sequence generation
2	Allocation concealment	UNCLEAR	No information on allocation concealment
3	Blinding participants/personnel (objective outcomes)	LOW	Blinding of participants was unconfirmed. Objective outcomes are unlikely to be influenced by this fact.
3	Blinding participants/personnel (subjective outcomes)	HIGH	Blinding of participants and personnel was unconfirmed. Subjective outcomes are likely to be influenced by this fact.
4	Blinding outcome assessors (objective outcomes)	LOW	Blinding of outcome assessors was unconfirmed. The judgment of objective outcomes is unlikely to be influenced by this fact.
4	Blinding outcome assessors (subjective outcomes)	HIGH	Blinding of participants and personnel was unconfirmed. Subjective outcomes are likely to be influenced by this fact.
5	Incomplete outcome	LOW	The outcome data of all randomized participants were presented
6	Selective reporting	UNCLEAR	No study protocol
7	Other bias	UNCLEAR	No information on baseline characteristics

For peer review only

Supplementary file 6, continuation

Figure S6. Risk of bias graph: review authors' judgements about each risk of bias item presented as percentages across all included studies.

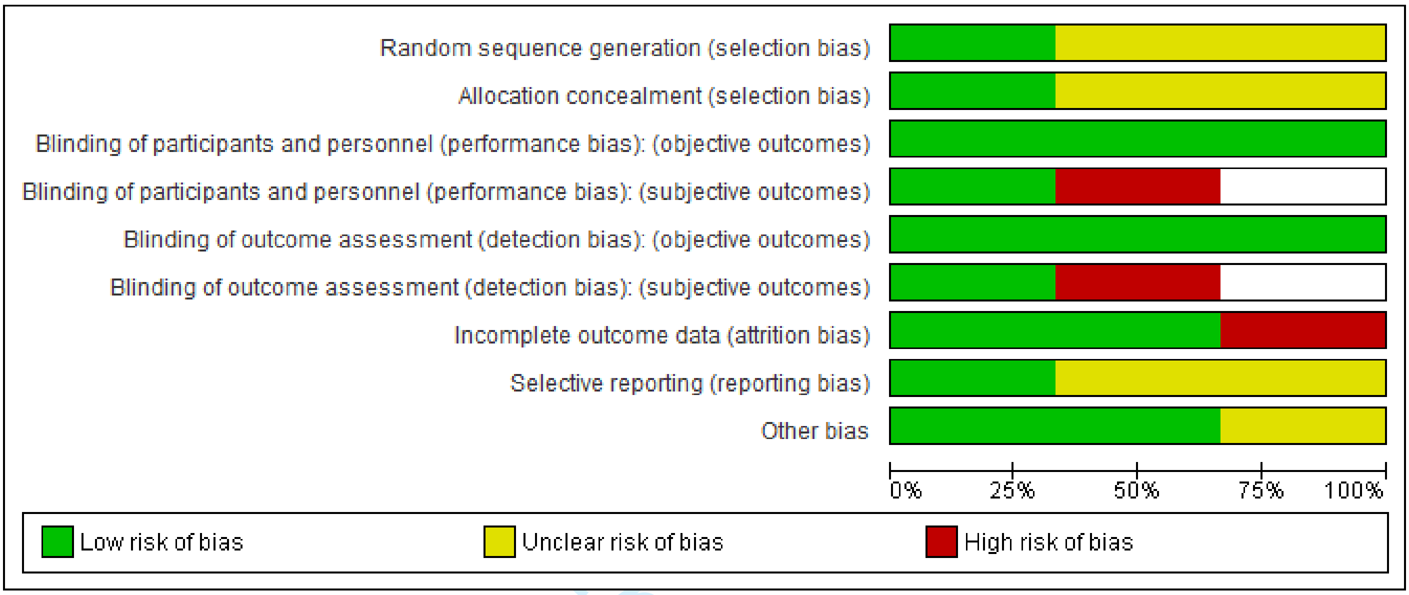

Supplementary file 7. Summary of findings table and GRADE: intramyometrial versus intravenous oxytocin at cesarean section

Intramyometrial compared to Intravenous prophylactic oxytocin for women giving birth by cesarean

Patient or population: women giving birth by cesarean
Setting: hospital
Intervention: intramyometrial oxytocin
Comparison: intravenous oxytocin

Outcomes	Anticipated absolute effects* (95% CI)		Relative effect (95% CI)	N of participants (studies)	Certainty of the evidence (GRADE)	Comments
	**Risk with Intravenous prophylactic oxytocin**	**Risk with Intramyometrial**				
Post-Partum Hemorrhage	60 per 1000	**8 per 1000** (1 to 162)	**RR 0.14** (0.01 to 2.70)	100 (1 RCT)	⊕⊕◯◯ LOW [a]	Mangla 2012: Blood loss > 900 mL.
Need for additional uterotonic	71 per 1000	**59 per 1000** (18 to 192)	**RR 0.82** (0.25 to 2.69)	140 (2 RCTs)	⊕◯◯◯ VERY LOW [a,b]	
Hypotension not requiring epinephrine	200 per 1000	**200 per 1000** (58 to 690)	**RR 1.00** (0.29 to 3.45)	40 (1 RCT)	⊕⊕◯◯ LOW [a]	
Hypotension requiring ephedrine	See comment	See comment	Not estimable	79 (2 RCTs)	⊕⊕◯◯ LOW [c]	The 2 trials had no events.
Nausea and/or Vomiting	157 per 1000	**20 per 1000** (3 to 108)	**RR 0.13** (0.02 to 0.69)	140 (2 RCTs)	⊕⊕◯◯ LOW [d,e]	
Headache	0 per 1000	**0 per 1000** (0 to 0)	**RR 3.00** (0.13 to 69.52)	40 (1 RCT)	⊕⊕◯◯ LOW [a]	
Facial flushing	100 per 1000	**50 per 1000** (5 to 508)	**RR 0.50** (0.05 to 5.08)	40 (1 RCT)	⊕⊕◯◯ LOW [a]	
Volume of blood loss	The mean volume of blood loss was **664.2** mL	MD **57.40 mL lower** (101.71 lower to 13.09 lower)	-	40 (1 RCT)	⊕⊕◯◯ LOW [f]	

***The risk in the intervention group** (and its 95% confidence interval) is based on the assumed risk in the comparison group and the **relative effect** of the intervention (and its 95% CI).
CI: Confidence interval; **RR:** Risk ratio; **MD:** Mean difference

GRADE Working Group grades of evidence
High certainty: We are very confident that the true effect lies close to that of the estimate of the effect
Moderate certainty: We are moderately confident in the effect estimate: The true effect is likely to be close to the estimate of the effect, but there is a possibility that it is substantially different
Low certainty: Our confidence in the effect estimate is limited: The true effect may be substantially different from the estimate of the effect
Very low certainty: We have very little confidence in the effect estimate: The true effect is likely to be substantially different from the estimate of effect

Explanations

a. Evidence certainty downgraded -2 due to very serious imprecision (very small number of events, and wide 95% CI crossing the line of no effect).
b. Evidence certainty downgraded -1 due to inconsistency (I2 61%)
c. Evidence certainty downgraded -2 due to very serious imprecision (lack of events).
d. Evidence certainty downgraded -1 due to study limitation (lack of blinding of participants, subjective outcome)
e. Evidence certainty downgraded -1 due to serious imprecision (small sample size)
f. Evidence certainty downgraded -2 due to very serious imprecision (small sample size and wide 95% confidence interval).

PRISMA 2009 Checklist

Section/topic	#	Checklist item	Reported on page #
**TITLE**			
Title	1	Identify the report as a systematic review, meta-analysis, or both.	1
**ABSTRACT**			
Structured summary	2	Provide a structured summary including, as applicable: background; objectives; data sources; study eligibility criteria, participants, and interventions; study appraisal and synthesis methods; results; limitations; conclusions and implications of key findings; systematic review registration number.	2
**INTRODUCTION**			
Rationale	3	Describe the rationale for the review in the context of what is already known.	3-4
Objectives	4	Provide an explicit statement of questions being addressed with reference to participants, interventions, comparisons, outcomes, and study design (PICOS).	4
**METHODS**			
Protocol and registration	5	Indicate if a review protocol exists, if and where it can be accessed (e.g., Web address), and, if available, provide registration information including registration number.	4, File S1
Eligibility criteria	6	Specify study characteristics (e.g., PICOS, length of follow-up) and report characteristics (e.g., years considered, language, publication status) used as criteria for eligibility, giving rationale.	4-5
Information sources	7	Describe all information sources (e.g., databases with dates of coverage, contact with study authors to identify additional studies) in the search and date last searched.	5
Search	8	Present full electronic search strategy for at least one database, including any limits used, such that it could be repeated.	File S2
Study selection	9	State the process for selecting studies (i.e., screening, eligibility, included in systematic review, and, if applicable, included in the meta-analysis).	5-6, Fig 1
Data collection process	10	Describe method of data extraction from reports (e.g., piloted forms, independently, in duplicate) and any processes for obtaining and confirming data from investigators.	5
Data items	11	List and define all variables for which data were sought (e.g., PICOS, funding sources) and any assumptions and simplifications made.	6, File S5
Risk of bias in individual studies	12	Describe methods used for assessing risk of bias of individual studies (including specification of whether this was done at the study or outcome level), and how this information is to be used in any data synthesis.	5-6
Summary measures	13	State the principal summary measures (e.g., risk ratio, difference in means).	6
Synthesis of results	14	Describe the methods of handling data and combining results of studies, if done, including measures of consistency (e.g., I^2) for each meta-analysis.	6

For peer review only - http://bmjopen.bmj.com/site/about/guidelines.xhtml

PRISMA 2009 Checklist

Section/topic	#	Checklist item	Reported on page #
Risk of bias across studies	15	Specify any assessment of risk of bias that may affect the cumulative evidence (e.g., publication bias, selective reporting within studies).	6
Additional analyses	16	Describe methods of additional analyses (e.g., sensitivity or subgroup analyses, meta-regression), if done, indicating which were pre-specified.	6
**RESULTS**			
Study selection	17	Give numbers of studies screened, assessed for eligibility, and included in the review, with reasons for exclusions at each stage, ideally with a flow diagram.	6-7 Fig 1, File S3
Study characteristics	18	For each study, present characteristics for which data were extracted (e.g., study size, PICOS, follow-up period) and provide the citations.	6-8, Table 1, File S4
Risk of bias within studies	19	Present data on risk of bias of each study and, if available, any outcome level assessment (see item 12).	9, Fig 2, File S6
Results of individual studies	20	For all outcomes considered (benefits or harms), present, for each study: (a) simple summary data for each intervention group (b) effect estimates and confidence intervals, ideally with a forest plot.	9
Synthesis of results	21	Present results of each meta-analysis done, including confidence intervals and measures of consistency.	9-10, Table 2,, Figs 3,4
Risk of bias across studies	22	Present results of any assessment of risk of bias across studies (see Item 15).	File S6
Additional analysis	23	Give results of additional analyses, if done (e.g., sensitivity or subgroup analyses, meta-regression [see Item 16]).	Not applicable
**DISCUSSION**			
Summary of evidence	24	Summarize the main findings including the strength of evidence for each main outcome; consider their relevance to key groups (e.g., healthcare providers, users, and policy makers).	10-11
Limitations	25	Discuss limitations at study and outcome level (e.g., risk of bias), and at review-level (e.g., incomplete retrieval of identified research, reporting bias).	11
Conclusions	26	Provide a general interpretation of the results in the context of other evidence, and implications for future research.	12
**FUNDING**			
Funding	27	Describe sources of funding for the systematic review and other support (e.g., supply of data); role of funders for the systematic review.	12

From: Moher D, Liberati A, Tetzlaff J, Altman DG, The PRISMA Group (2009). Preferred Reporting Items for Systematic Reviews and Meta-Analyses: The PRISMA Statement. PLoS Med 6(7): e1000097. doi:10.1371/journal.pmed1000097

For peer review only
